# Anion-Controlled Synthesis of Novel Guanidine-Substituted Oxanorbornanes

**DOI:** 10.3390/ijms232416036

**Published:** 2022-12-16

**Authors:** Luka Barešić, Davor Margetić, Zoran Glasovac

**Affiliations:** Laboratory for Physical Organic Chemistry, Division of Organic Chemistry and Biochemistry, Ruđer Bošković Institute, Bijenička cesta 54, 10000 Zagreb, Croatia

**Keywords:** cycloaddition, guanidines, oxanorbornane, hexafluorophosphate, halide anion, acidity, aza-Michael reaction

## Abstract

The cycloaddition of simple alkyl-substituted guanidine derivatives is an interesting approach toward polycyclic superbases and guanidine-based organocatalysts. Due to the high nucleophilicity of guanidines, an aza-Michael reaction with dienophiles is more common and presents a huge obstacle in achieving the desired synthetic goal. Our preliminary investigations indicated that the proton could act as a suitable protecting group to regulate the directionality of the reaction. To investigate the role of the protonation state and type of anion, the reactivity of furfuryl guanidines with dimethyl acetylenedicarboxylate was explored. Furfuryl guanidines showed a strong reaction dependence on the nucleophilicity of the counterion and the structure of guanidine. While the reaction of **DMAD** with the guanidinium halides provided products of an aza-Michael addition, Diels–Alder cycloaddition occurred if non-nucleophilic hexafluorophosphate salts were used. Depending on the structure and the reaction conditions, oxanorbornadiene products underwent subsequent intramolecular cyclization. A tendency toward intramolecular cyclization was interpreted in terms of the p*K*_a_ of different positions of the guanidine functionality in oxanorbornadienes. New polycyclic guanidines had a slightly decreased p*K*_a_ in acetonitrile and well-defined geometry suitable for the buildup of selective sensors.

## 1. Introduction

Oxanorbornane is a well-recognized rigid bicyclic moiety present in many naturally occurring compounds [1]. It is often used as the rigid subunit in the construction of biologically and technologically interesting molecules—glycomimetics [2], β-turn inducers [3], amphiphilic systems for drug delivery [4], anion transporting polymers [5], self-healing polymers [6,7], etc. Substituted oxanorbornanes are readily prepared by a variety of methods, of which the cycloaddition/hydrogenation sequence starting from (un)substituted furans is the most common [1]. This synthetic approach could be considered ecofriendly due to the combination of two reactions with perfect atom economy and the usage of bioavailable furan derivatives.

The rigidity of oxanorbornanes [8] ensures a well-defined orientation of the substituents that were used in designing novel molecular systems with enhanced recognition capabilities [9,10]. In that sense, guanidines are promising subunits that are well-known for their high basicity and binding capabilities regardless of whether they were used as organocatalysts [11,12] or as substrate-binding moieties within a larger system [13,14]. In both cases, guanidine activity primarily originates from their high tendency to create a functional group pairing through the formation of multiple charge-assisted hydrogen bonds [15].

Although the chemistry of oxanorbornanes is well developed, guanidine-containing derivatives are rather scarce in the literature. Their preparation is limited to a postfunctionalization of the cycloadducts and encompasses at least one protection/deprotection step [5]. Employing the guanidine-substituted dienes or dienophiles directly in the cycloaddition reaction renders these steps unnecessary. The main obstacle to this approach is the well-known tendency of guanidines to undergo aza-Michael addition to activated olefins. Namely, less substituted derivatives usually undergo aza-Michael addition to acrylates [16], maleimides [17], or dimethyl acetylenedicarboxylate (**DMAD**) [18,19], and it is often associated with cyclization to imidazolidin-4-one derivatives [16,20]. The addition of persubstituted guanidines to the dienophiles was also confirmed to be a reversible process [21], and in some instances, the guanidines were even used as catalysts for the cycloaddition reactions [22]. Furthermore, the mechanism of formal Diels–Alder reactions of guanidine-derived heterodienes with various dienophiles has been interpreted as a tandem nucleophilic addition/cyclization process [23]. Our interest in guanidine chemistry is focused on derivatives with at least two NH bonds available for the recognition and substrate binding, and investigating this requires a proper approach to highly selective guanidine/dienophile cycloaddition chemistry.

**Figure 1:**
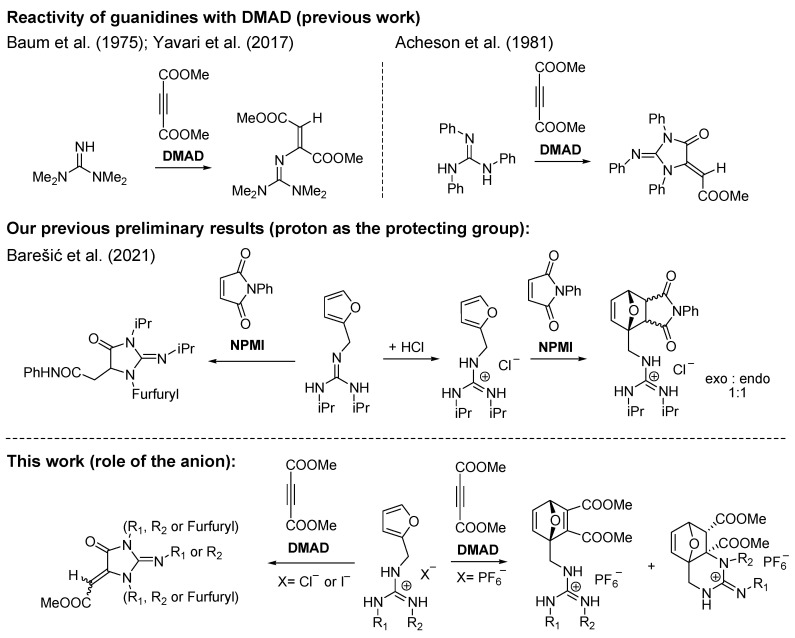
Typical reactivity of guanidines and their salts with typical dienophiles **DMAD** and **NPMI** [16,18,19,24].

We recently conducted preliminary experiments on the cycloaddition of trisubstituted guanidines to *N*-phenylmaleimide (**NPMI**) and obtained creatinine derivatives as a consequence of a tandem Michael addition/imide ring-opening reaction (Figure 1) [24]. On the other hand, when the reaction was conducted with guanidinium salts, cycloaddition occurred smoothly without any indication of a significant side reaction. Apparently, using a proton as the protecting group works well with maleimide derivatives to prevent Michael additions. While being highly encouraging, a lack of selectivity toward *exo* or *endo* cycloaddition products turned our attention to **DMAD** as the dienophile. Namely, the hydrogenation of oxanorbornadienes obtained by the cycloaddition of furans with **DMAD** proceeds in a diastereospecific manner producing *endo*-oxanorbornane products only [25]. Diastereospecific synthesis is essential for the future construction of the target organocatalysts and receptors based on guanidine-substituted oxanorbornanes.

In this work, the impact of the anion selection on the discrimination between the cycloaddition and aza-Michael addition as a typical concurrent reaction is described (Figure 1). Starting from furfuryl guanidinium hexafluorophosphates and **DMAD**, the successful synthesis of several guanidine-substituted oxanorbornadienes was achieved. Depending on the structure of the diene, polycyclic (condensed) guanidinooxanorbornenes were formed.

## 2. Results and Discussion

**Scheme 1:**
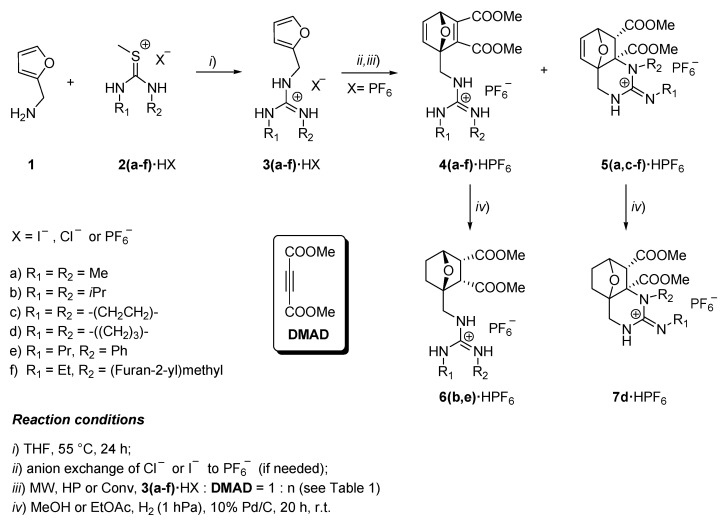
Cycloaddition of furfurylguanidine hexafluorophosphates (**3(a**−**f)·**HPF_6_) with **DMAD**. Hydrogenation was conducted only for the selected derivatives.

The synthesis of oxanorbornanes containing a guanidine moiety was performed starting with furfuryl amine as a bioavailable starting molecule (Scheme 1) [26]. Furfurylamine was first converted to the furfuryl guanidinium salt [27], which could be considered the “proton protected” [28] guanidine. Our initial attempts of cycloadditions of **DMAD** with guanidinium chlorides or iodides did not provide any trace of target oxanorbornadienes. Instead, different products of aza-Michael addition/cyclization (**AMA/CYC**) reactions were formed (see Section 2.2).

The usage of guanidinium salts with a weakly nucleophilic hexafluorophosphate anion resulted in the formation of the target oxanorbornadienes **4(a**–**f)·**HPF_6_ without any trace of the imidazolidinone-type products. The best results were obtained with MW heating the reaction mixture at 100 °C for 1 h. To achieve the complete conversion of the starting guanidine **3(a**–**f)·**HPF_6_ to **4(a**−**f)·**HPF_6_, the reactant ratio of guanidine **DMAD** = 1:6 was used. Lower ratios resulted in incomplete conversion after 1 h of reaction time. Prolonged heating or a higher temperature led to an increased amount of polymeric material. The reaction conditions, product ratios, and unoptimized yields are given in Table 1. In certain cases, products were isolated in the hydrogenated form either to reveal the isolation as the origin of the relatively low yields (entries 14 and 16) or to avoid product decomposition (entry 17). The hydrogenation of **4b·**HPF_6_ proceeded smoothly providing similar overall yields of oxanorbornadiene or oxanorbornane products (entries 5 and 6).

Together with the expected formation of oxanorbornadienes **4x·**HPF_6_, cyclization to the novel three or tetracyclic derivatives **5x·**HPF_6_ was also identified (Scheme 1), leading to the interesting cycloaddition/intramolecular cyclization (**CA/IMC**) tandem approach to the novel polycyclic guanidines. The formation of the polycyclic derivatives **5x·**HPF_6_ was attributed to the classical Diels–Alder [4+2] reaction followed by intramolecular aza-Michael addition. The latter reaction took place via the nucleophilic attack of the guanidine nitrogen atom even though it was protonated. Generally, oxanorbornadienes are known to react with nucleophiles, which can result in the promoted retro-DA process [29]. In our case, no appearance of new furan signals that would indicate a retro-DA process was observed.

Of all target oxanorbornadienes, diisopropyl derivative **4b·**HPF_6_ did not show any tendency toward intramolecular cyclization. For other derivatives, the ratio of **4x**/**5x** was found to be highly sensitive toward the amount of moisture, usage of protic solvents, and aging of the starting material (presumably due to partial hydrolysis of the hexafluorophosphate anion). Upon using freshly prepared and carefully dried **3d·**HPF_6_, only a cycloadduct was formed as indicated by the NMR spectrum, while the amount of the polycyclic product **5d·**HPF_6_ was below the detection limit. In the case of **3c·**HPF_6_, even freshly prepared guanidine produced approximately 10% of the tetracyclic derivative **5c·**HPF_6_. If guanidine salt **3c·**HPF_6_ aged for 1 month was used, the ratio of the product **5c·**HPF_6_ increased to 22.5% (Table 1, entries 8 and 9). An even more pronounced difference was observed in the cycloaddition of **3a·**HPF_6_, where an almost 1: 1 ratio was obtained (Table 1, entry 2). In addition to promoting intramolecular cyclization, the usage of the aged starting materials also led to a somewhat increased amount of polymeric materials.

A huge effect on the product ratio was observed when the reaction was conducted under high pressure (5–7 kbar) [30]. As expected, applying high pressure conditions promoted cyclization leading to **5x·**HPF_6_ as the strongly preferred product (Table 1, entries 10, 11, 18, and 19). However, the conversions were moderate to poor unless the duration of the reaction was significantly prolonged (Table 1, entries 11 and 19) presumably due to a lower reaction temperature.

The purification of target oxanorbornadienes by chromatography was problematic. Passing the reaction mixture containing less than 10% of the cyclization product **5x·**HPF_6_ over the short column of silica gel led to a complete loss of oxanorbornadiene while the amount of cyclic product **5x·**HPF_6_ increased (exemplified by the loss of **4a·**HPF_6_ upon chromatography, Figure S1, Supplementary Materials). The latter was concluded from the results given in entries 7, 12, 14, and 15, where the amount of isolated cyclic products was significantly higher than what should be expected from the product ratios. The low isolated yields of **5a·**HPF_6_ could be ascribed to the pronounced decomposition of oxanorbornadiene that was faster than the **IMC** step. The hydrogenation of the reaction mixture in the EtOAc prevented **IMC** and the oxanorbornanes could be isolated in moderate to good yields (Table 1, Entries 16 and 17).

In view of other results, the aforementioned absence of the **IMC** process in the case of isopropyl derivative **4b·**HPF_6_ was somewhat surprising. While the sterics could be thought of as the most likely reason, the results for **4e·**HPF_6_ indicate that we should take a more detailed insight into the structure and properties of starting guanidines. Namely, the polycyclic derivative **5e·**HPF_6_ was produced by a nucleophilic attack of the guanidine nitrogen atom bearing a phenyl substituent, which is sterically more crowded. We should emphasize that the HN(Ph) position also had a higher acidity than the HN(Pr) one (vide infra), and, expectedly, it was more active in hydrogen bonding and more susceptible to partial deprotonation. With that in mind, the NMR experiment was conducted in which the oxanorbornadiene **4b·**HPF_6_ (crude product) was effectively cyclized upon the addition of the phosphazene base P1*tBu (Figure S2, Supplementary Materials), confirming deprotonation as the trigger of the **IMC** process. Therefore, we concluded that the acid/base properties of the functional groups play a more important role in cyclization than the steric demands.

We could also note that despite the expected higher nucleophilicity of guanidine **3d** than of **3c** [31], the results indicate a higher tendency of **3c·**HPF_6_ toward **CA/IMC** products. It should be noted that **3c**H^+^ is expected to be more acidic than **3d**H^+^ [32]. Based on these results, we assume that the partial deprotonation of the guanidine subunit takes place during the reaction. The tentative mechanism is briefly discussed in the next section.

### 2.1. Mechanism of the CA/IMC Tandem Process

**Figure 2:**
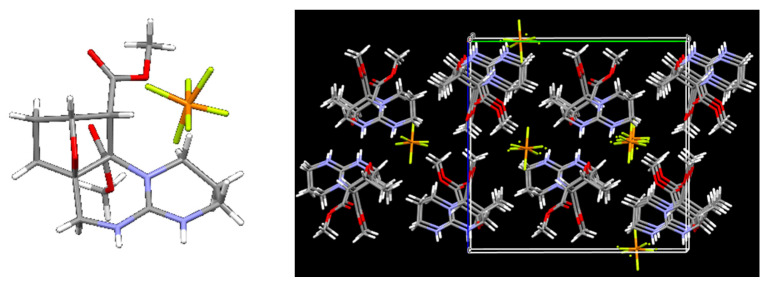
X-ray structure of the derivative **7d·**HPF_6_.

Before the discussion on the possible mechanism, we shall briefly analyze the main structural characteristics of the polycyclic derivative **7d·**HPF_6_ as the representative of all structures formed upon **CA/IMC**. The crystal structure (Figure 2) revealed the *syn/exo* stereochemistry of cyclization, which was also supported by the 1D and 2D NMR spectra. The main indicator of the *syn/exo* addition was the multiplicity of the hydrogen atom at the bridgehead position (doublet) and the coupling with the hydrogen located at the oxa-bridge (^3^*J*_HH_ > 4 Hz). The data obtained were typical for *exo* position of the bridgehead hydrogen atom. When asymmetric guanidine derivatives were used, ^1^H,^13^C-HMBC, and NOESY spectra were used to confirm which nitrogen atom acted as a nucleophile. The spectroscopic data were in agreement with the proposed attack from the *exo* side (see Section S2 in Supplementary Materials) and showed that the phenyl and furfuryl groups activated the nucleophilicity of the nitrogen atom. This geometrical feature contrasts the previous results on the intermolecular addition of amines to the oxanorbornadiene, where the assigned configuration of the *Z*-β-enamine retro-DA product implied the *anti*-addition of amine and a proton [26]. The crystal structure also revealed a layered arrangement of the guanidine pairs bound together by two guanidinium oxa-bridge hydrogen bonds. Hexafluorophosphate anions filled the pockets between layers below the guanidine plane and only weakly participated in the coordination of one of the guanidinium NH bonds. Ester groups were positioned perpendicularly to the guanidine moiety with one of them located at approximately 3 Å to one of the guanidinium nitrogen atoms while the other one stacked with the π-system of the guanidinium cation moiety from the same layer along axis a.

To reiterate, the main noncovalent interaction in the structure was between the guanidine subunit and the oxa-bridge. A similar finding was also expected in oxanorbornadiene providing a preorganized structure for the *exo* attack of the nucleophilic nitrogen atom.

**Scheme 2:**
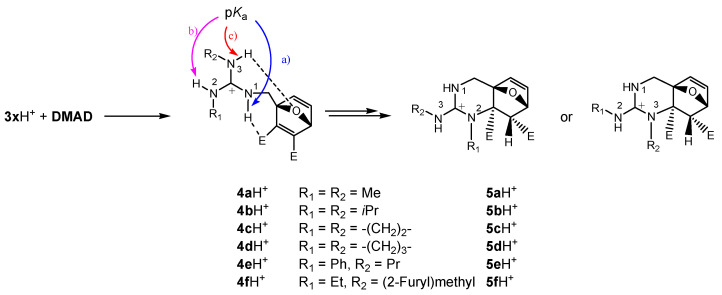
Cyclization of guanidinium-substituted oxanorbornadienes (**4x**H^+^) with indicated different positions for the hydrogen bonding/partial deprotonation. Anions were deliberately neglected from the calculations.

Taking into account the fact that *syn*—*exo* addition was experimentally proven (Figure 2), that deprotonation triggers the **IMC** process (Figure S2, Supplementary Materials), and that the extent of **IMC** is higher if the aged reactant was used (Table 1), we assumed several key points of the mechanism. They are as follows: (a) the process is acid catalyzed, (b) the acid is either moisture or HF liberated in the aged samples, (c) the proton-donating component is bound to the guanidine subunit by the noncovalent interactions, and (d) internal proton transfer from the guanidine moiety to one of the basic functional groups occurs just before or during C-N bond formation. The last two key points are directly related to the p*K*_a_ of the guanidine NH bond involved in hydrogen bonding. To test these assumptions, we calculated the p*K*_a_ values of all the positions of the guanidine moiety in derivatives **4a**H^+^–**4f**H^+^. The structures of the guanidinium cations with marked hydrogen bonding interactions are schematically presented in Scheme 2. Their calculated p*K*_a_s together with Gibbs energies of **CA**/**IMC** reactions are given in Table 2.

Our calculations indicated that the most acidic NH bond in the considered guanidinium cations was generally the one closest to the oxanorbornadiene fragment, with **4e**H^+^ being an exemption. Deprotonation at that position would not lead to cyclization due to the formation of a highly unfavorable four-membered ring. In the case of **4e**H^+^, the most acidic site was the NH(Ph) one, which was expected given the known trends in p*K*_a_ values of tetramethylguanidines and P1 phosphazenes [33,34,35]. By comparing it with the second p*K*_a_ values of other derivatives (usually the N_R1_ position), we obtained the trend **4e**H^+^ < **4c**H^+^ < **4f**H^+^ < **4a**H^+^ < **4d**H^+^ < **4b**H^+^. The results were consistent with two qualitative observations: the absence of the **IMC** process during the cycloaddition of **3b·**HPF_6_ and a somewhat higher tendency toward the **IMC** process of **4c·**HPF_6_ over **4d·**HPF_6_ as visible from Table 1. This result is somewhat in opposition to the conclusion that could be drawn from the literature about the nucleophilicity of the cyclic guanidines **TBD** and **TBO** [31]. Additionally, the trend in p*K*_a_s calculated for **4e**H^+^ and **4f**H^+^ was in full agreement with the structures of the isolated products and supports our proposition on internal deprotonation as the trigger for cyclization.

The thermodynamics of the **CA** and **CA/IMC** reactions were calculated using the M11/aug-cc-pVTZ//M11/6-31+G(d,p) method. Both optimization and single-point calculations were conducted in acetonitrile treated as a dielectric continuum using the SMD approach. The employed method was shown to provide very good agreement with CCSD(T) benchmark calculations for the Diels–Alder reaction [36]. For the sake of comparison, the results obtained by the B3LYP model were added, for which the pronounced shift of the reaction energies toward the endothermic region is known [36,37]. The M11 results showed a strong overall thermodynamical stabilization of the products over the reactants. From the thermodynamic point of view, the most reactive derivative was expected to be **3d**H^+^ while the p*K*_a_ analysis predicted its relatively low reactivity within the series. The smallest stabilization was obtained for **4b**H^+^ but it was still sufficient to rule out the thermodynamics of the reaction as the origin of the absence of the **IMC** process. As expected, the B3LYP approach predicted an unrealistically strong endergonicity of the reactions and, opposite to M11, predicted destabilization going from **4b**H^+^ to **5b**H^+^. While this result is consistent with the absence of an **IMC** product in this particular case (Table 1, entries 3–6), unfavorable thermodynamics estimated for the **CA** step calculated for all derivatives render these results unreliable. Going to the larger basis set has an additional negative impact on the results.

### 2.2. Halides as Nucleophiles. Aza-Michael/Cyclization

As already mentioned, the reaction of **DMAD** with guanidinium chlorides or iodides (**3(b**–**d)·**HX) produced 2-aminoimidazolidinone derivatives **9**–**12** together with 2-halofumarate **8X** (**8Cl** or **8I** depending on the starting guanidinium salt, Scheme 2) as confirmed by the NMR and GC-MS measurements. The obtained imidazolidinone derivatives closely resembled the expected products that would be obtained by the aza-Michael addition (**AMA**) of the neutral guanidine to **DMAD** as described in the literature [19,20]. In the case of guanidine salt **3a·**HI, a complex reaction mixture was obtained, and we were unable to identify the products formed.

**Scheme 3:**
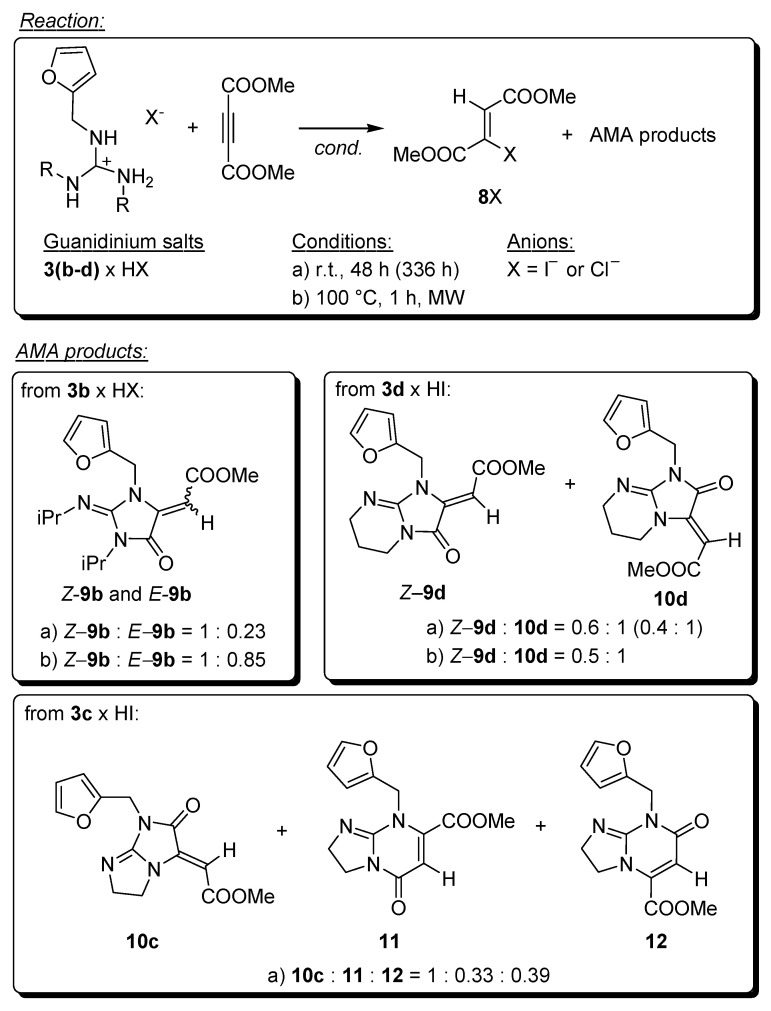
The products obtained from the aza-Michael reaction of hydrohalide salts of **3b**–**3d** with **DMAD** (**AMA/CYC** products).

To explain the results, we need to take a closer look at the reactivity of **DMAD**. Its sensitivity toward nucleophiles is well known [20,38]. The formation of 2-halofumarates was already described by Billetter and coworkers who noticed their formation from alkali halides and acetylene dicarboxylic acid [39,40]. From the reactions of hexafluorophosphate salts described above, we can conclude that a guanidinium cation will not act as a nucleophile toward **DMAD** unless deprotonation takes place to a significant extent. Based on this, we are proposing the initial addition of a halide anion to **DMAD** as the trigger that generates the halofumaryl anion, which is sufficiently basic to deprotonate the guanidinium cation. Thus, the formed neutral guanidines underwent an **AMA**/**CYC** tandem reaction, which provided the observed 2-aminoimidazolidinone derivatives **9**–**12** (Scheme 3). The structural assignment of the isomers was performed with X-ray analysis, 1D and 2D spectra, and DFT calculations of the NMR chemical shifts (details are given in Section S2, Supplementary Material). The appearance of the deep dark red color serves as additional evidence of the neutral guanidine **DMAD** reaction [41].

Somewhat surprising is the structural diversity of the isolated products. Starting from **3b·**HX, the formation of both *Z* and *E* isomers of **9b** was observed while no other possible products were isolated. Their ratio was dependent on temperature, with the *E* isomer being less abundant going from approximately 11% (r.t.) to approximately 47% (100 °C). In the case of **3c·**HI, we did not observe the presence of the (*Z*/*E*)–**9c** isomer, but rather the derivatives **10c**–**12**. The cyclization of the aminoimidazole derivatives to the remote carboxylic group from **DMAD** to avoid the bicyclo[3.3.0]octane structure was already described by Acheson and Willis [16], and in that sense, our results are consistent with the literature. Finally, the aza-Michael addition of the guanidinium salt **3d·**HI furnished *Z*–**9d** and **10d** as the main products, while no presence of 1,5,7-triazabicyclo[4.4.0]decene derivatives was isolated and confirmed. Apparently, cyclization to the proximate carboxylic group is highly preferred. Another qualitative observation showed that the reactivity of the endocyclic nitrogen atoms in **3c** and **3d** was comparable to or higher than the exocyclic one. On the other hand, a nitrogen atom bearing an isopropyl substituent was quite unreactive toward double and triple C-C bonds, which can be deduced from the absence of the **IMC** process in **4b·**HPF_6_ and of the **10b** -type **AMA**/**CYC** product. A tentative explanation of the latter observation includes the increased basicity of the guanidine nitrogen atom attached to the isopropyl group, which disfavors the *i*Pr-N=C tautomeric form, and more pronounced steric congestion with respect to the alkyl, cycloalkyl, and furfuryl groups. A more detailed computational investigation of these results is underway and will be published separately.

## 3. Materials and Methods

General remarks on synthesis and reactivity of furfuryl guanidines: All furfuryl guanidinium salts (**3x**) were prepared via the guanidinylation of furfurylamines with isothiouronium iodides according to the general procedure described by Aoyagi and Endo [27]. Hexafluorophosphates were obtained using the following reaction sequence: (*i*) deprotonation of iodides, (*ii*) reprotonation of the crude neutral guanidine with 1M aqueous HCl, and (*iii*) anion exchange with ammonium hexafluorophosphate. Guanidinium hexafluorophosphates were isolated by the extraction of the heterogeneous aqueous mixture.

Cycloaddition reactions were conducted using the following experimental procedures (Table 1):

**MW**: 100 °C, 1 h, in acetonitrile, 1 cm^3^ per 1 mmol of reactant **3(a–f)·**HPF_6_, MW heating.

**HP**: high pressure (5–7 kbar), 0.5 mmol of guanidine in 1 cm^3^ of DCM.

**Conv**: 120 °C (temperature of oil bath), closed microwave vessel, 1 h, in acetonitrile, 1 cm^3^ per 1 mmol of reactant **3c·**HPF_6._

Microwave reactions were performed in a Single mode CEM Discover reactor using 150 W of the initial power and 60 min of the hold time at the temperature of 100 °C. HP reactions were conducted in a high-pressure piston–cylinder apparatus, Institute of Physical Chemistry, Polish Academy of Sciences, Warsaw, Poland, in Teflon cells, and pentane was used as a piezotransmitter liquid.

Aza-Michael reactions were conducted at room temperature for 48 or 366 h or under MW heating for 1 h as described above. The reactant ratios were **3(a**–**d)·**HI : **DMAD** = 1:2 in all cases. Detailed descriptions of all instrumentation, experimental procedures, and spectroscopic data of the isolated products are given in the Supplementary Materials.

X-ray structure determination: Crystal structures were determined with a Rigaku XtaLAB Synergy S diffractometer using ShellXL for the structure refinement [42]. The structural data were deposited in the Cambridge Crystallographic Data Centre (CCDC) under the following deposit numbers: 2,224,646 (**5c·**HPF_6_), 2,224,362 (**7d·**HPF_6_), 2,224,384 (**5f·**HPF_6_), 2,224,367 (*E*–**9b**), 2,224,371 (*Z*–**9b**), and 2,224,372 (**10c**). Details about the crystallization conditions, solvents, ORTEP images, and the selected data are given in the Supplementary Materials.

Computational details: All the structures were fully optimized and the nature of the stationary points was confirmed with vibrational analysis (NImag = 0). Calculations were performed using the Gaussian09 [43] (p*K*_a_ calculations) or Gaussian16 [44] (thermodynamics of the reactions) program packages. The p*K*_a_ calculations were conducted using the computational scheme developed earlier and improved recently [45,46]. For this purpose, a reduced Gibbs energy of the protonation (Δ*G*′_a_(BH^+^)) was calculated using either the B3LYP//B3LYP or MP2/B3LYP approach and was converted to p*K*_a_ by using empirical correlations as given in Equations (1) (B3LYP//B3LYP) and (2) (MP2//B3LYP) [45,46]:

p*K*_a_(BH^+^) = 0.545 × Δ*G*′_a_(BH^+^) − 133.5 (1)

p*K*_a_(BH^+^) = 0.601 × Δ*G*′_a_(BH^+^) − 146.8 (2)

The thermochemistry of the reactions was calculated using M11/aug-cc-pVTZ//M11/6-31+G(d,p) (M11 model). B3LYP calculations were conducted at the B3LYP/6-31G(d,p) level of theory without (B3-S) or with additional correction for the electronic energies calculated from B3LYP/6-31+G(2df,p) single point calculations (B3-L). For the sake of identification of the structures, NMR shieldings were calculated using the GIAO/6-311+G(d,p)//B3LYP/6-31G(d) approach. Except for the p*K*_a_ calculations, optimizations and single point calculations were performed in either acetonitrile (thermochemistry) or chloroform (NMR) using predefined parameters (*ε*_0_(CH_3_CN) = 35.688; *ε*_0_(CHCl_3_) = 4.7113). The solvents were treated implicitly by employing the SMD continuum solvation approach [47].

## 4. Conclusions

In this work, we described the reactions of several guanidinium salts with **DMAD** as the typical dienophile in the Diels–Alder reaction and electrophile in the aza-Michael addition reaction. By proper choice of the counterion and structural features, one can direct the reaction to the desired products. Starting from the salts with non-nucleophilic anions, cycloaddition products could be obtained in moderate to high yields in a relatively short reaction sequence. Most of the tested guanidines underwent intramolecular aza-Michael cyclization despite their protonation state. Such a tandem reaction (cycloaddition/aza-Michael intramolecular cyclization) can be considered an elegant approach to the novel, condensed, and rigid polycyclic guanidines. Due to the well-defined position of the guanidine and carboxylic groups, we envision these types of compounds as the building blocks for the future synthesis of novel catalysts and anion sensors. Our results also confirm that the proton is a sufficiently good protecting group for the cycloaddition, as it prevents the nucleophilic attack of guanidine on **DMAD**. Based on the DFT calculations, observed intramolecular cyclization was ascribed to the formation of thermodynamically significantly more stable oxanorbornene derivatives and the high mobility of the proton. The latter is strongly related to the p*K*_a_ value of the guanidinium position that is responsible for the nucleophilic attack.

Cycloaddition is not possible when nucleophilic anions are present in the reaction mixture. The halofumarate anion formed in situ deprotonated the guanidinium ion, leading to the neutral guanidines resulting in the aza-Michael reaction instead of cycloaddition. Somewhat surprising is the diversity of the products isolated from the reaction mixtures, and it is tentatively ascribed to the combination of the stability of guanidine tautomers, nucleophilicity, and thermodynamic stability of the products. An investigation of the mechanism is underway.

## Figures and Tables

**Table 1:** Employed reaction conditions, product ratios, conversion, and isolated yields in the reaction of furfurylguanidinium hexafluorophosphates with **DMAD**.

Entry	Reactant	Method ^a^	Equiv./Time ^b^	4x·HPF_6_/ 5x·HPF_6_	Conv./% (Yield/%) ^c,d^
1	**3a**·HPF_6_	MW	1:6/1 h	85/15	90 (36 as **5a**·HPF_6_)
2	**3a**·HPF_6_ ^e^	MW	1:6/1 h	57/43	98 (13 as **5a**·HPF_6_)
3	**3b·**HPF_6_	MW	1:3/1 h	100/0	79 [57 as **4b**·HPF_6_] ^f^
4	**3b**·HPF_6_	MW	1:3/4 h	100/0	98 (70 as **4b**·HPF_6_)
5	**3b**·HPF_6_	MW	1:6/1h	100/0	98 (72 as **4b**·HPF_6_)
6	**3b**·HPF_6_ ^e^	MW	1:6/1 h	100/0	98 (71 as **6b**·HPF_6_)
7	**3c**·HPF_6_	MW	1:3/1 h	91/9	83 (50 as **5c**·HPF_6_)
8	**3c**·HPF_6_	Conv.	1:6/1 h	88/12	94 (30 as **5c**·HPF_6_) [56 as **5c**·HPF_6_] ^f^
9	**3c**·HPF_6_ ^e^	MW	1:6/1 h	77/23	94 (70 as **5c**·HPF_6_)
10	**3c**·HPF_6_	HP	1:1.1/24 h	5/95	20 (n/d)
11	**3c**·HPF_6_	HP	1:1.1/30 days	4/96	71 (n/d)
12	**3d**·HPF_6_	MW	1:3/1 h	88/12	73 (50 as **5d**·HPF_6_)
13	**3d**·HPF_6_	MW	1:6/1 h	100/0	98 [61 as **4d**·HPF_6_ ] ^f^
14	**3d**·HPF_6_	MW	1:6/1 h	100/0	98 (65 as **7d**·HPF_6_)
15	**3e**·HPF_6_	MW	1:6/1 h	93/7	94 (33 as **5e**·HPF_6_)
16	**3e**·HPF_6_	MW	1:6/1 h	95/5	94 (64 as **6e**·HPF_6_)
17	**3f**·HPF_6_	MW	1:6/1 h	96/4	97 (57 as **6f·**HPF_6_)
18	**3f**·HPF_6_	HP	1:1.1/24 h	13/87	23 (n/d)
19	**3f**·HPF_6_	HP	1:1.1/144 h	13/87	82 (75 as **5f**·HPF_6_)

^a^ Reaction conditions are given in the Section 3. ^b^ Equiv. relates to the starting molar ratio of the guanidines salts **3(a**–**f)·**HPF_6_ and **DMAD** used. The amount of **DMAD** corresponds to the n used in Scheme 1. ^c^ Conversion **·**s determined from the ^1^H NMR spectra of the crude reaction mixture. ^d^ Isolated, nonoptimized yield, n/d = not determined. ^e^ Aged sample of **3a·**HPF_6_, **3b·**HPF_6_, or **3c·**HPF_6_ stored over two months in a closed flask and not dried. ^f^ NMR yields calculated using *p*-dimethoxybenzene or *N*^1^,*N*^3^-dimethylthiourea as the internal standard are given in square brackets.

**Table 2:** Calculated ^ACN^p*K*_a_s for all three positions in the guanidine subunit and the Gibbs energies of the reaction for the cycloaddition and cyclization of the oxanorbornadiene ions **4a**H^+^–**4f**H^+^.

Oxanorb.	N_X_	^ACN^p*K*_a_ ^a^	Model ^b^	⚜_r_*G*(CA) ^c^	⚜_r_*G*(tot) ^c,d^
**4a**H^+^	N1	22.6 (22.4)	M11	−10	−96
	N2,3	23.0 (22.7)	B3-S (B3-L)	16 (52)	−26 (23)
**4b**H^+^	N1	23.3 (22.1)	M11	−16	−78
	N2,3	24.0 (23.1)	B3-S (B3-L)	16 (52)	6 (60)
**4c**H^+^	N1	20.5 (20.5)	M11	−3	−98
	N2,3	21.4 (21.0)	B3-S (B3-L)	16 (56)	−37 (16)
**4d**H^+^	N1	22.3 (22.5)	M11	−18	−102
	N2,3	23.5 (22.8)	B3-S (B3-L)	19 (55)	−22 (27)
**4e**H^+^	N_1_ N_2_ N_3_	21.6 (21.5) 19.6 (18.8) 21.8 (21.9)	M11 B3-S (B3-L)	−18 18 (55)	−91 −14 (35)
**4f**H^+^	N_1_ N_2_ N_3_	21.8 (21.0) 23.4 (22.8) 22.6 (21.4)	M11 B3-S (B3-L)	−14 14 (46)	−92 −14 (36)

^a^ p*K*_a_ was calculated using two linear correlation approaches, see Computational details. Values in parentheses refer to the MP2//B3LYP model. ^b^ For the description of the computational models, see the Computational details section. ^c^ In kJ mol^−1^, respective to the indefinitely separated reactants **DMAD** and **3x**H^+^. Values in parentheses relate to the values obtained using B3LYP/6-311+G(2df,p) single point energy calculations, see Computational details. ^d^ Δ_r_*G*(tot) relates to the Gibbs energy of the tandem **CA/IMC** process.

## Data Availability

The data are available in this publication and Supplementary Materials.

## Supplementary Materials

The following supporting information can be downloaded at https://www.mdpi.com/article/10.3390/ijms232416036/s1: Detailed experimental procedures, spectroscopic data, details on the structural analysis of the obtained products by 2D NMR techniques, DFT calculations and X-ray structural analysis, calculations of the p*K*_a_ and **CA/IMC** thermochemistry data, and cartesian coordinates of all the optimized structures.

## References

[1] Moreno-Vargas A.J., Vogel P., Cossy J. (2014). Synthesis of 7-Oxabicyclo[2.2.1]heptane and Derivatives. Topics in Heterocyclic Chemistry 35. Synthesis of Saturated Oxygenated Heterocycles I.

[2] Vogel P. (2008). Total Asymmetric Synthesis of Glycomimetics and Polypropionates of Biological Interest. Chimia.

[3] Hackenberger C.P.R., Schiffers I., Runsink J., Bolm C. (2004). General Synthesis of Unsymmetrical Norbornane Scaffolds as Inducers for Hydrogen Bond Interactions in Peptides. J. Org. Chem..

[4] Saroj S., Janni D.S., Ummadi C.R., Manheri M.K. (2020). Functionalizable oxanorbornane-based head-group in the design of new Non-ionic amphiphiles and their drug delivery properties. Mat. Sci. Eng. C.

[5] Hennig A., Gabriel G.J., Tew G.N., Matile S. (2008). Stimuli-Responsive Polyguanidino-Oxanorbornene Membrane Transporters as Multicomponent Sensors in Complex Matrices. J. Am. Chem. Soc..

[6] Chen X., Dam M.A., Ono K., Mal A., Shen H., Nutt S.R., Sheran K., Wudl F. (2002). A thermally re-mendable cross-linked polymeric material. Science.

[7] Khan N.I., Halder S., Gunjan S.B., Prasad T. (2018). A review on Diels-Alder based self-healing polymer composites. IOP Conf. Ser. Mater. Sci. Eng..

[8] Warrener R.N., Margetić D., Sun G., Amarasekara A.S., Foley P., Butler D.N., Russell R.A. (1999). Molecular topology: The synthesis of a new class of rigid arc-shaped spacer molecules based on *syn*-facially fused norbornanes and 7-heteronorbornanes in which heterobridges are used to govern backbone curvature. Tetrahedron Lett..

[9] Briš A., Trošelj P., Margetić D. (2017). Cooperativity in Binding of Aliphatic Diamines by Bis(Zn(II)porphyrin) Receptor with Moderately Flexible Linkers. Croat. Chem. Acta.

[10] Robson R.N., Hay B.P., Pfeffer F.M. (2019). To Cooperate or Not: The Role of Central Functionality in Bisthiourea [6]polynorbornane Hosts. Eur. J. Org. Chem..

[11] Selig P. (2013). Guanidine Organocatalysis. Synthesis.

[12] Chou H.-C., Leow D., Tan C.-H. (2019). Recent Advances in Chiral Guanidine-Catalyzed Enantioselective Reactions. Chem. Asian J..

[13] Schug K.A., Lindner W. (2005). Noncovalent Binding between Guanidinium and Anionic Groups:  Focus on Biological- and Synthetic-Based Arginine/Guanidinium Interactions with Phosph[on]ate and Sulf[on]ate Residues. Chem. Rev..

[14] Schmuck C. (2006). How to improve guanidinium cations for oxoanion binding in aqueous solution?: The design of artificial peptide receptors. Coord. Chem. Rev..

[15] Hargrove A.E., Nieto S., Zhang T., Sessler J.L., Anslyn E.V. (2011). Artificial Receptors for the Recognition of Phosphorylated Molecules. Chem. Rev..

[16] Acheson R.M., Wallis J.D. (1981). Addition reactions of heterocyclic compounds. Part 74. Products from dimethyl acetylenedicarboxylate with thiourea, thioamide, and guanidine derivatives. J. Chem. Soc. Perkin Trans..

[17] Shikhaliev K.S., Kovygin Y.A., Potapov A.Y., Sabynin A.L., Kosheleva E.A. (2017). Recyclization of maleimides with N-carboximide amides. Russ. Chem. Bull. Int. Ed..

[18] Baum J., Scholz D., Tataruch F., Viehe H.G. (1975). New syntheses of amino-substituted 2-azabutadienes. Chimia.

[19] Yavari I., Amirahmadia A., Halvagar M.R. (2017). A Synthesis of Functionalized Thiazoles and Pyrimidine-4(3H)-thiones from 1,1,3,3-Tetramethylguanidine, Acetylenic Esters, and Aryl Isothiocyanates. Synlett.

[20] Neochoritis C.G., Zarganes–Tzitzikas T., Stephanidou-Stephanatou J. (2014). Dimethyl Acetylenedicarboxylate: A Versatile Tool in Organic Synthesis. Synthesis.

[21] Horvath A. (1996). Catalysis and regioselectivity in the Michael addition of azoles. Kinetic vs. thermodynamic control. Tetrahedron Lett..

[22] Shen J., Nguyen T.T., Goh Y.-P., Ye W., Fu X., Xu J., Tan C.-H. (2006). Chiral Bicyclic Guanidine-Catalyzed Enantioselective Reactions of Anthrones. J. Am. Chem. Soc..

[23] Li H., Zhao J., Zeng L., Hu W. (2011). Organocatalytic Asymmetric Domino Aza-Michael–Mannich Reaction: Synthesis of Tetrahydroimidazopyrimidine Derivatives. J. Org. Chem..

[24] Barešić L., Margetić D., Glasovac Z. (2021). Cycloaddition of Thiourea- and Guanidine-Substituted Furans to Dienophiles: A Comparison of the Environmentally-Friendly Methods. Chem. Proc..

[25] Barlow M.G., Suliman N.N.E., Tipping A.E. (1995). A high-yield synthesis of 3-carboethoxy-4-trifluoromethylfuran and some Diels-Alder reactions of this furoate with acetylenic dienophiles. J. Fluor. Chem..

[26] Zhang P., Liao X., Ma C., Li Q., Li A., He Y. (2019). Chemoenzymatic Conversion of Corncob to Furfurylamine via Tandem Catalysis with Tin-Based Solid Acid and Transaminase Biocatalyst. ACS Sustain. Chem. Eng..

[27] Aoyagi N., Endo T. (2017). Synthesis of five- and six-membered cyclic guanidines by guanylation with isothiouronium iodides and amines under mild conditions. Synth. Commun..

[28] Oganesyan A., Cruz I.A., Amador R.B., Sorto N.A., Lozano J., Godinez C.E., Anguiano J., Pace H., Sabih G., Gutierrez C.G. (2007). High Yield Selective Acylation of Polyamines:  Proton as Protecting Group. Org. Lett..

[29] Huang C., Yin Y., Guo J., Wang J., Fan B., Yang L. (2014). A facile synthesis of β-amino carbonyl compounds through an aza-Michael addition reaction under solvent-free conditions. RSC Adv..

[30] Margetić D. (2019). High Pressure Organic Synthesis.

[31] Kiesewetter M.K., Scholten M.D., Kirn N., Weber R.L., Hedrick J.L., Waymouth R.M. (2009). Cyclic Guanidine Organic Catalysts: What Is Magic About Triazabicyclodecene?. J. Org. Chem..

[32] Ghobril C., Hammar P., Kodepelly S., Spiess B., Wagner A., Himo F., Baati R. (2010). Structure–Reactivity Relationship Studies for Guanidine-Organocatalyzed Direct Intramolecular Aldolization of Ketoaldehydes. ChemCatChem.

[33] Kaljurand I., Rodima T., Pihl A., Mäemets V., Leito I., Koppel I.A., Mishima M. (2003). Acid-Base Equilibria in Nonpolar Media. 4. Extension of the Self-Consistent Basicity Scale in THF Medium. Gas-Phase Basicities of Phosphazenes. J. Org. Chem..

[34] Kaljurand I., Kütt A., Sooväli L., Rodima T., Mäemets V., Leito I., Koppel I.A. (2005). Extension of the Self-Consistent Spectrophotometric Basicity Scale in Acetonitrile to a Full Span of 28 p*K*_a_ Units: Unification of Different Basicity Scales. J. Org. Chem..

[35] Zall C.M., Linehan J.C., Appel A.M. (2015). A Molecular Copper Catalyst for Hydrogenation of CO_2_ to Formate. ACS Catal..

[36] Yepes D., Valenzuela J., Martínez-Araya J.I., Pérez P., Jaque P. (2019). Effect of Exchange-Correlation Functional on the Synchronicity/Nonsynchronicity in Bond Formation in Diels-Alder Reactions: A Reaction Force Constant Analysis. Phys. Chem. Chem. Phys..

[37] Mezei P.D., Csonka G.L., Kallay M. (2015). Accurate Diels–Alder Reaction Energies from Efficient Density Functional Calculations. J. Chem. Theory Comput..

[38] Fan M.-J., Lia G.-Q., Liang Y.-M. (2006). DABCO catalyzed reaction of various nucleophiles with activated alkynes leading to the formation of alkenoic acid esters, 1,4-dioxane, morpholine, and piperazinone derivatives. Tetrahedron.

[39] Billetter H., Pantenburg I., Ruschewitz U. (2005). Lithium hydrogen iodo-fumarate monohydrate. Acta Cryst..

[40] Billetter H., Pantenburg I., Ruschewitz U. (2006). Copper(II) chloro-fumarate trihydrate. Acta Cryst..

[41] Shestakov A.S., Sidorenko O.E., Shikhaliev K.S. (2007). Synthesis of 2-iminoimidazolidin-4-one derivatives by cyclization of 2-aryl-1-(4,6-dimethylpyrimidin-2-yl)guanidines with ethyl bromoacetate, dimethyl acetylenedicarboxylate, and maleic anhydride. Russ. Chem. Bull. Int. Ed..

[42] Sheldrick G.M. (2015). Crystal structure and refinement with SHELXL. Acta Cryst..

[43] Frisch M.J., Trucks G.W., Schlegel H.B., Scuseria G.E., Robb M.A., Cheeseman J.R., Scalmani G., Barone V., Mennucci B., Petersson G.A. (2009). Gaussian09. Rev D. 01.

[44] Frisch M.J., Trucks G.W., Schlegel H.B., Scuseria G.E., Robb M.A., Cheeseman J.R., Scalmani G., Barone V., Petersson G.A., Nakatsuji H. (2019). Gaussian16. Rev C. 01.

[45] Glasovac Z., Eckert-Maksić M., Maksić Z.B. (2009). Basicity of organic bases and superbases in acetonitrile by the polarized continuum model and DFT calculations. New J. Chem..

[46] Glasovac Z., Kovačević B. (2022). Modeling p*K*_a_ of the Brønsted Bases as an Approach to the Gibbs Energy of the Proton in Acetonitrile. Int. J. Mol. Sci..

[47] Marenich A.V., Cramer C.J., Truhlar D.G. (2009). Universal Solvation Model Based on Solute Electron Density and on a Continuum Model of the Solvent Defined by the Bulk Dielectric Constant and Atomic Surface Tensions. J. Phys. Chem. B.

