# Supplementary material for: Anion-Controlled Synthesis of Novel Guanidine-Substituted Oxanorbornanes
Source: Int J Mol Sci. 2022 Dec 16;23(24):16036. doi: 10.3390/ijms232416036 (PMC10375892; doi:10.3390/ijms232416036)
Supplement: Supplementary file 1 [file ijms-23-16036-s001.zip › ijms-2057906-supplementary.pdf]

Supplemental Material

for

Anion Controlled Synthesis of Novel Guanidine Substituted Oxanorbornanes

Luka Barešić, Davor Margetić, Zoran Glasovac.

S1. Experimental part: ... 2

S1.1. Synthesis of precursors ... 2

S1.2. Cycloaddition reactions ... 7

S1.3. Aza-Michael reactions ... 13

S2. NMR analysis of structures of products ... 18

a) 2D NMR Analysis of the structure of **CA/IMC** products ... 18

b) 2D NMR Structural assignation of **AMA/CYC** products ... 19

b) NMR calculations of the possible isomers obtained by the reaction of **3c** HI with **DMAD**: ... 22

c) GC-MS data for the **AMA/CYC** products obtained from **3c**·HI ... 25

d) X-ray structures ... 27

S3. NMR and IR spectra ... 31

S4. HRMS intrumentation and spectra ... 69

S5. pK_a calculations ... 84

S6. Cartesian coordinates ... 89

a). Optimized geometries used in pK_a calculations ... 89

b). Optimized structures used for calculations of $\Delta_r G$ ... 110

c) Optimized structures used in NMR calculations ... 123

S7. References ... 128

S1. Experimental part:

General remarks: Petroleum ether (bp 65-90 °C) and dichloromethane were purchased from Gram-Mol (Croatia) and distilled before use. THF, ethyl acetate, acetonitrile, and methanol were purchased from Riedel-de-Haën and used without prior purification unless stated otherwise. Furfuryl amine, dimethyl thiourea, diisopropyl thiourea, and imidazolidine-2-thione (ethylene thiourea) were purchased from Alfa Æsar. Propylene thiourea [1] was synthesized as described elsewhere. Microwave-assisted reactions were conducted on CEM Discover single-mode instrument in a closed vessel equipped with a magnetic stirrer. The temperature was controlled by the external IR sensor located below the vessel. NMR spectra were recorded on Bruker Avance AV600 and AV300 using TMS as the reference compound. X-ray structure determination was conducted on Rigaku XtaLAB Synergy S diffractometer. High–resolution mass spectra (HRMS) were recorded on a 4800 Plus Maldi TOF/TOF Analyser and Agilent 6550 Series Accurate-Mass-Quadrupole Time-of-Flight (Q-TOF). Infrared spectra (FTIR-ATR) were recorded on the Fourier Transform Infrared Attenuated Total Reflection PerkinElmer UATR Two Spectrometer (range 400-4000 cm^{-1}).

Guanidinium iodides **3a**·HI – **3f**·HI and their conjugate bases **3a** – **3f** were synthesized by guanidylation of furfuryl amine with isothouronium iodide followed by deprotonation with 40 % NaOH as described by Aoyagi and Endo [1] (Method A). NMR spectra are consistent with the literature data. Hexafluorophosphate salts were prepared by reprotonation of the neutral guanidines with equimolar amounts of 1M aqueous HCl, adding a slight excess of NH_4PF_6 and extraction of the formed heterogeneous mixture with CH_2Cl_2 or EtOAc.

S1.1. Synthesis of precursors

General procedure for the synthesis of the neutral guanidines 3a – 3f (Method A) [1]**:**

a) *Guanidylation of amines:* Furfuryl amine and corresponding isothiouronium halide or hexafluorophosphate were stirred in THF for 16 hours at 40–50°C. The crude reaction mixture was cooled and evaporated to dryness. The crude product was treated with diethyl ether. The precipitate was filtered with suction and washed thoroughly with an additional amount of ether. The solid product was dried at a high vacuum for 1 h. ^{1}H NMR spectra of the crude mixture showed that the product is pure enough for further usage.

b) Deprotonation of hydroiodides: Crude hydroiodide salts were added to 40 % aqueous NaOH and stirred for 30 min. The mixture is then extracted with CH_2Cl_2, organic layers were collected, dried on anhydrous $MgSO_4$, filtered, and evaporated to dryness. So obtained crude neutral guanidine was used for the preparation of the hexafluorophosphate salts.

N^1,N^3-diisopropyl-N^2-furfurylguanidinium chloride (3b·HCl, Method B):

N,*N'*-Diisopropyl carbodiimide (**DIC**, 1.515 g, 12.0 mmol), and furfuryl amine (1.110 g, 11.4 mmol) were mixed in a microwave vessel, capped, and heated with stirring at 90 °C for 6 hours under MW irradiation (P = 150 W). After cooling, the reaction mixture was transferred to the round-bottomed flask and distilled under high vacuum conditions. The faintly yellowish fraction distilling at 60-80 °C *p* = 0.01-0.04 Pa) was collected providing 1.781 g (8.0 mmol, 70 %) of crude guanidine which was used in the preparation of the chloride and hexafluorophosphate salts without further purification.

Crude product **3b**, obtained by distillation (1.781 g) was transferred to the 20 cm^3 of methanol containing 5.8 cm^3 of 1M HCl in water. The mixture was evaporated to dryness giving viscous oil. After treatment with a small amount of acetonitrile (3 cm^3), a white precipitate of the desired hydrochloride **3b**·HCl is formed upon cooling to 4 °C overnight. The product was separated by suction and dried yielding 1.074 g (4.1 mmol, 36 % of overall yield starting from **DIC** and furfuryl amine).

^{1}H-NMR (DMSO-d_6, 600 MHz) δ/ppm: 1.12 (d, $^3J_{H-H}$ = 6.4 Hz, 12H, -CH(C$\underline{H}_3$)$_2$); 3.94 (m, 2H, -C$\underline{H}$(CH$_3$)$_2$); 4.56 (d, $^3J_{H-H}$ = 5.6 Hz, 2H, -C$\underline{H}_2$-N); 6.36 (d, $^3J_{H-H}$ = 3.1 Hz, 1H, Furan C(3)$\underline{H}$); 6.41 (dd, $^3J_{H-H}$ = 3.1 Hz, 1.8 Hz, 1H, Furan C(4)$\underline{H}$); 7.37 (d, 2H, $^3J_{H-H}$ = 8.2 Hz, =CHN$\underline{H}$-); 7.62 (d, $^3J_{H-H}$ = 1.8 Hz, 1H, Furan C(5)$\underline{H}$); 8.14 (t, $^3J_{H-H}$ = 5.7 Hz, 1H, CH$_2$N$\underline{H}$-).

^{13}C NMR (DMSO-d_6, 150.9 MHz) δ/ppm: 22.7, 38.2, 44.3, 108.4, 110.9, 143.1, 151.1, 153.2.

General procedure for the synthesis of hexafluorophosphate salts from chlorides: Neutral guanidine was dissolved in water and acidified with an equimolar amount of 1M HCl. To the formed homogenous solution, a slight excess (1.05–1.1 mol equivalent) of NH_4PF_6 was added. The solution became turbid and a water-immiscible layer appeared. The mixture was extracted with DCM, dried, and evaporated to dryness. Obtained hexafluorophosphates were used in cycloaddition reactions without further purification.

***N*^{1},*N*^{3}-dimethyl-*N*^{2}-furfurylguanidinium hexafluorophosphate (3a**·HPF_6**):** Crude neutral guanidine **3a** obtained by deprotonation of the corresponding iodide (1.606 g, 9.6 mmol) was dissolved in 25 cm^3 of water acidified with 10 cm^3 of 1M aqueous HCl. To this solution, 1.630 g (10.0 mmol) of ammonium hexafluorophosphate was added in one portion. The mixture was shaken until turbidity turns into the water-immiscible layer. A heterogeneous mixture was then extracted with 3 x 30 cm^3 of DCM. After evaporation of the solvent, 1.600 g (5.1 mol, 53 %) of the colorless viscous oil that solidifies upon overnight refrigeration at +4°C was obtained.

^{1}H-NMR (DMSO-d_6, 600 MHz) δ/ppm: 2.76 (d, $^3J_{H-H}$ = 4.6 Hz, 6H, -NC$\underline{H}_3$); 4.39 (d, $^3J_{H-H}$ = 6.0 Hz, 2H, -NC$\underline{H}_2$Furyl); 6.38 (dd, $^3J_{H-H}$ = 3.2 Hz, $^3J_{H-H}$ = 0.6 Hz, 1H, Furan C(3)*H*); 6.44 (dd, $^3J_{H-H}$ = 3.2 Hz, $^3J_{H-H}$ = 1.8 Hz, 1H, Furan C(4)*H*); 7.57 (br d, $^3J_{H-H}$ = 3.3 Hz, 2H,-N*H*CH$_3$); 7.65 (dd, $^3J_{H-H}$ = 1.8 Hz, $^3J_{H-H}$ = 0.7 Hz, 1H, Furan C(5)*H*); 7.92 (t, $^3J_{H-H}$ = 6.0 Hz, 1H, -N*H*CH$_2$Furyl).

^{13}C NMR (DMSO-d_6, 150.9 MHz) δ/ppm: 28.1, 37.6, 107.8, 110.6, 142.9, 150.2, 155.3.

FTIR-ATR ν_{max}/cm^{-1}: 3443 (N-H stretch), 1636 (C=N asymm. stretch), 825 (P-F stretch).

Melting point/ °C: 75-76.

HRMS- Q-TOF found: [MH]$^+$: 168.1137, calculated for [$C_8H_{14}N_3O$]$^+$, [MH]$^+$: 168.1131

***N*^{1},*N*^{3}-diisopropyl-*N*^{2}-furfurylguanidinium hexafluorophosphate (3b**·HPF_6**):** Crude neutral guanidine **3b** (0.523 g, 2.34 mmol) was dissolved in 10 cm^3 of water acidified with 2.3 cm^3 of 1M aqueous HCl. To this solution, 0.408 g (2.47 mmol) of ammonium hexafluorophosphate was added in one portion. The mixture was shaken until turbidity turns into the water-immiscible layer. A heterogeneous mixture was then extracted with 3 x 30 cm^3 of DCM. After evaporation of the volatile material, 0.854 g (2.31 mol, 97 %) of the colorless viscous oil that solidifies upon overnight refrigeration at +4°C was obtained.

^{1}H-NMR (DMSO-d$_6$, 300 MHz) δ/ppm: 1.12 (d, $^{3}J_{H-H}$ = 6.4 Hz, 12H, -CH(C$\underline{H}_3$)$_2$); 3.83 (m, 2H, -C$\underline{H}$(CH$_3$)$_2$); 4.48 (s, 2H, -C$\underline{H}_2$-N); 6.35 (d, $^{3}J_{H-H}$ = 2.9 Hz, 1H, Furan C(3)$\underline{H}$); 6.44 (dd, $^{3}J_{H-H}$ = 2.9 Hz, 1.8 Hz, 1H, Furan C(4)$\underline{H}$); 7.10 (br d, 2H, $^{3}J_{H-H}$ = 6.0 Hz, =CHN$\underline{H}$-); 7.65 (d, $^{3}J_{H-H}$ = 0.8 Hz, 1H, Furan C(5)$\underline{H}$); 7.86 (br s, 1H, CH$_2$N$\underline{H}$-).

^{13}C NMR (DMSO-d$_6$, 75.5 MHz) δ/ppm: 22.2, 37.8, 43.7, 108.0, 110.5, 142.9, 150.3, 152.7.

FTIR-ATR ν_{max}/cm^{-1}: 3407 (N-H stretch), 1616 (C=N asymm. stretch), 820 (P-F stretch).

Melting point/ °C: 67-69.

HRMS- Q-TOF found: [MH]$^+$: 224.1766, calculated for [$C_{12}H_{22}N_3O$]$^+$, [MH]$^+$: 224.1757

(Furan-2-yl)-*N*-(imidazolidin-2-ylidene)methanammonium hexafluorophosphate (3c·HPF$_6$):

Crude **3c** (1.631 g, 10.0 mmol), obtained by deprotonation as described above (Method A), was dissolved in 10 cm^3 of the 1M aqueous HCl and stirred until the mixture became homogenous. Afterward, 1.721 g (10.6 mmol) of ammonium hexafluorophosphate was added with stirring upon which a white precipitate was formed. The mixture was diluted with additional 15 cm^3 of water and extracted with EtOAc (3 x 25 cm^3). Organic layers were collected, dried, and evaporated providing 2.793 g (9.0 mmol, 90 %) of the target **3c**·HPF$_6$.

^{1}H-NMR (DMSO-d$_6$, 600 MHz) δ/ppm: 3.61 (s, 4H, -CH$_2$CH$_2$-); 4.38 (d, $^{3}J_{H-H}$ = 6.0 Hz, 2H, -C$\underline{H}_2$-N); 6.40 (d, $^{3}J_{H-H}$ = 3.2 Hz, 1H, Furan C(3)$\underline{H}$); 6.44 (dd, $^{3}J_{H-H}$ = 3.2 Hz, 1.8 Hz, 1H, Furan C(4)$\underline{H}$); 7.66 (m, 1H, Furan C(5)$\underline{H}$); 8.69 (t, $^{3}J_{H-H}$ = 5.9 Hz, 1H, CH$_2$N$\underline{H}$-). The other two NH signals are strongly broadened between 7 and 9 ppm as confirmed by integration of the whole interval (4H, includes three NH and one furan signal).

^{13}C NMR (DMSO-d$_6$, 150.9 MHz) δ/ppm: 38.8, 42.5, 108.2, 110.6, 143.1, 149.9, 159.2.

FTIR-ATR ν_{max}/cm^{-1}: 3408 (N-H stretch), 1664 (C=N asymm. stretch), 816 (P-F stretch).

Melting point/ °C: 88-90.

(Furan-2-yl)-*N*-(tetrahydropyrimidin-2(1H)-ylidene)methanammonium hexafluorophosphate (3d·HPF$_6$)

Due to the low reactivity of the corresponding thiouronium salt, a modification of the guanidylation procedure was employed. The round-bottom flask was charged with furfuryl amine (1.62 g, 19.89 mmol) and methyl(tetrahydropyrimidin-2(1H)-ylidene)sulfonium iodide (1.93 g, 6.27 mmol) and the reaction mixture was stirred at 110°C for 1 h. The reaction mixture was cooled to room temperature and washed with 2 x 10 cm^3 of Et$_2$O. The crude reaction mixture was transferred to a separatory funnel, by washing the reaction vessel with 25 cm^3 of water divided into two portions. The water layer was extracted with 2 x 10 cm^3 of EtOAc, and then 2 x 15 cm^3 of DCM. To the remaining colorless water layer ammonium hexafluorophosphate (1.35 g, 8.27 mmol) was added, and the water layer was extracted again with 2 x 15 cm^3 of EtOAc. The organic layer obtained from the third extraction was dried over anhydrous sodium sulfate and filtered. EtOAc was evaporated under reduced pressure and from remained yellow oil product slowly crystalized upon standing or upon seeding with few crystals from the previous batch. The product was isolated as pale yellow solid (1.86 g, 6.1 mmol, 97 % yield).

^{1}H-NMR (DMSO-d$_6$, 300 MHz) δ/ppm: 1.81 (quint, $^{3}J_{H-H}$ = 5.7 Hz, 2H, -CH$_2$CH_2CH$_2$-); 3.26 (t, 4H, $^{3}J_{H-H}$ = 5.7 Hz, -CH_2CH$_2$CH_2-); 4.33 (s, 2H, -C$\underline{H}_2$-N); 6.37 (d, $^{3}J_{H-H}$ = 3.0 Hz, 1H, Furan C(3)$\underline{H}$); 6.44 (dd, $^{3}J_{H-H}$ = 3.0 Hz, 1.8

Hz, 1H, Furan C(4)*H*); 7.65 (br s, 1H, Furan C(5)*H*, overlapped with NH); 7.79 (br s, 3H, N*H*-, confirmed by integration of whole interval (4H), includes three NH and one furane signal).

^{13}C NMR (DMSO-d$_6$, 75.5 MHz) δ/ppm: 19.6, 37.1, 38.1, 107.9, 110.6, 142.9, 150.2, 152.5.

FTIR-ATR ν_{max}/cm^{-1}: 3426 (N-H stretch), 1629 (C=N asymm. stretch), 812 (P-F stretch).

Melting point/ °C: 65-67.

HRMS- Q-TOF found: $[MH]^+$: 180.1137, calculated for $[C_9H_{14}N_3O]^+$, $[MH]^+$: 180.1137

N^1–Furfuryl–N^2–phenyl–N^3–propylguanidinium hexafluorophosphate (3e·HPF_6)

Conversion of the corresponding isothiouronium salt to the target guanidinium hexafluorophosphate **3e**·HPF_6 was conducted without isolation of the hydroiodide. The mixture of 1.606 g (4.3 mmol) of isothiouronium salt **1e**·HI and 1.0 cm^3 (0.719 g, 12.2 mmol) of $PrNH_2$ in 10 cm^3 of acetonitrile was stirred for 24 hours at 50–60 °C (temperature of the oil bath). Volatile components were removed under reduced pressure upon which viscous oily material remained. The crude impure product mixture contained also some remaining unreacted amine and the starting isothiouronium salt. The impure product was further subjected to deprotonation with 40 % NaOH as described above (Method A) except that the extraction was conducted with diethyl ether. Deprotonated crude guanidine (1.261 g) was dissolved in MeOH, 4.0 cm^3 of 1M HCl was added and the mixture was evaporated to dryness. The obtained material was redissolved in water (20 cm^3) and the solution was extracted with Et_2O (3 x 15 cm^3). The organic layer was discarded. To the aqueous layer, 0.751 g (4.61 mmol) of NH_4PF_6 and 20 cm^3 of Et_2O were added and the mixture was stirred for 30 min. The organic layer was separated and the aqueous layer was additionally extracted with 2 x 20 cm^3 of Et_2O. Combined organic extracts were dried on $MgSO_4$, filtered, and evaporated to dryness. In this way, we obtained 1.374 g (3.4 mmol, 79 %) of product in a form of a tan/white solid of acceptable NMR purity.

^{1}H-NMR (DMSO-d$_6$, 300 MHz) δ/ppm: 0.84 (t, $^3J_{H-H}$ = 7.4 Hz, 3H, -$CH_2CH_2C\underline{H}_3$); 1.52 (m, 2H, -$CH_2CH_2C\underline{H}_3$); 3.20 (m, 2H, -$CH_2C\underline{H}_2C\underline{H}_3$); 4.50 (s, 2H, -Furyl–C*H*$_2$–N); 6.39 (d, $^3J_{H-H}$ = 3.0 Hz, 1H, Furan C(3)*H*); 6.46 (dd, $^3J_{H-H}$ = 3.0 Hz, 1.9 Hz, 1H, Furan C(4)*H*); 7.16 (d, 2H, $^3J_{H-H}$ = 7.5 Hz, *ortho*- Phenyl); 7.30 (t, 1H, $^3J_{H-H}$ = 7.4 Hz, *para*-Phenyl); 7.46 (t, 2H, $^3J_{H-H}$ = 7.7 Hz, *meta*- Phenyl); 7.69 (d, $^3J_{H-H}$ = 0.9 Hz, 1H, Furan C(5)*H*); 7.94 (br s, 1H, N*H*-); 8.20 (br s, 1H, N*H*-); 9.47 (br s, 1H, N*H*-).

^{13}C NMR (DMSO-d$_6$, 150.9 MHz) δ/ppm: 10.9, 21.7, 38.2, 43.3, 108.1, 110.6, 124.4, 126.3, 129.7, 135.9, 143.0, 149.8, 153.8.

FTIR-ATR ν_{max}/cm^{-1}: 3395 (N-H stretch), 1619 (C=N asymm. stretch), 801 (P-F stretch).
Melting point/ °C: 95-97.

HRMS- Q-TOF found: $[MH]^+$: 258.1617, calculated for $[C_{15}H_{20}N_3O]^+$, $[MH]^+$: 258.1601

N^2–Ethyl–N^1,N^3–furfurylguanidinium hexafluorophosphate (3f·HPF_6):

Ethylfurfuryl guanidinium iodide **3f·**HI was prepared as described above under Method A.

To a stirring solution of furfuryl amine (1.589 g, 16.36 mmol) in 46 cm^3 of tetrahydrofuran *N*-furfuryl-*N'*-ethyl isothiuronium iodide (4.641 g, 14.3 mmol) was added and the reaction mixture was heated on 55 °C for 20 h. The solvent was evaporated under reduced pressure, and the remaining yellow solid was washed

with ethyl acetate until the white solid remained. Product (**3f**·HI) was isolated as a white solid (5.019 g, 13.4 mmol, 94%).

^{1}H–NMR (DMSO-d$_6$, 300 MHz) δ/ppm: 1.06 (t, 3H, , $^3J_{HH}$ = 7.1 Hz, -CH$_2$C*H*$_3$); 3.18–3.23 (m, 2H, -C*H*$_2$CH$_3$); 4.45 (d, 4H, , $^3J_{HH}$ = 5.7 Hz, furyl-C*H*$_2$N); 6.30 (d, 2H, , $^3J_{HH}$ = 2.8 Hz, Furan C(3)*H*); 6.43 (dd, 2H, , $^3J_{HH}$ = 3.1 Hz, , $^3J_{HH}$ = 1.9, Furan C(4)*H*); 7.64 (d, 2H, , $^3J_{HH}$ = 0.9 Hz, Furan C(5)*H*); 7.68 (t, 1H, , $^3J_{HH}$ = 5.5 Hz, NH); 8.08 (t, 2H, , $^3J_{HH}$ = 5.7 Hz, 2 x NH)

Anion exchange: In contrast to other derivatives, a somewhat different approach to iodide/hexafluorophosphate exchange was employed. In 220 cm^3 of water *N*, *N'*-bis-furfuryl-*N''*-ethyl guanidinium iodide (2.700 g, 8.3 mmol) was dissolved and to the solution was added ammonium hexafluorophosphate (2.721 g, 16.7 mmol). After 1h at 4 °C white solid crystallizes. The solid was filtered and washed 2x with 5 cm^3 of water. Product (**3f**·HPF$_6$) was isolated as a white solid (2.510 g, 6.4 mmol, 77%) of satisfactory purity for the cycloadditions.

^{1}H-NMR (DMSO-d$_6$, 300 MHz) δ/ppm: 1.07 (t, $^3J_{H\text{-}H}$ = 7.1 Hz, 3H, -CH$_2$C*H*$_3$); 3.20–3.24 (m, 2H, -C*H*$_2$CH$_3$); 4.45 (d, $^3J_{H\text{-}H}$ = 5.0 Hz, 4H, -C*H*$_2$Furyl); 6.30 (d, $^3J_{H\text{-}H}$ = 2.8 Hz, 2H, Furan C(3)*H*); 6.42 (m, 2H, Furan C(4)*H*); 7.63 (br s, 2H, Furan C(5)*H*); 7.69 (t, $^3J_{H\text{-}H}$ = 5.1 Hz, 1H, -N*H*CH$_2$Et); 8.08 (t, $^3J_{H\text{-}H}$ = 5.0 Hz, 2H, -N*H*CH$_2$Furyl);

^{13}C NMR (DMSO-d$_6$, 75.5 MHz) δ/ppm: 14.2, 36.2, 37.8, 107.8, 110.6, 142.9, 150.0, 154.0.

FTIR-ATR ν_{max}/cm^{-1}: 3417 (N-H stretch), 1627 (C=N asymm. stretch), 817 (P-F stretch).

Melting point/ °C: 96-97.

HRMS- Q-TOF found: [MH]$^+$: 248.1401, calculated for [C$_{13}$H$_{18}$N$_3$O$_2$]$^+$, [MH]$^+$: 248.1399

S1.2. Cycloaddition reactions

General procedure for cycloaddition reaction to DMAD: 1 mol-eq of the vacuum-dried guanidinium hexafluorophosphate was weighed in a microwave vessel equipped with a magnetic stirrer and 1 cm^3 of acetonitrile per 1 mmol of guanidinium salt was added and briefly stirred. To this mixture, 6 mol-eq of **DMAD** was added and the vessel was capped. The reaction mixture was heated at 100 °C for 1h. The cooled reaction mixture was transferred to a round-bottomed flask and the solvent and the excess of **DMAD** were removed by vacuum distillation.

***N*-[(2,3-dicarbomethoxy-7-oxabicyclo[2.2.1]hepta-2,5-dien-1-yl)methyl]-*N'*,*N''*-dimethylguanidinium hexafluorophosphate (4a·HPF_6)**
and
6,7-dicarbomethoxy-5-methyl-4-methylamino-11-oxa-3,5-diazatricyclo[6.2.1.0^{1,6}]undec-9-en-4-ylium hexafluorophosphate (5a·HPF_6)

The reaction of freshly prepared **3a**·HPF_6 (313 mg, 1.0 mmol), **DMAD** (740 µl, 856 mg, 6.0 mmol), and 1 cm^3 of acetonitrile in a capped microwave vessel under microwave heating (100 °C, 60 min) formed a light yellowish-brown mixture. After evaporation of volatiles under high vacuum conditions (p = 1-5 Pa), the remaining viscous material was mulled in diethyl ether (4 x 2 cm^3); the ether layer was discarded, and the remaining material was evaporated to dryness. The crude product (485 mg) is predominantly desired oxanorbornadiene containing ca 18 % of its cyclic derivative **5a**·HPF_6 and ca 20 % of **DMAD**, which remained in the viscous mixture. The crude reaction mixture was filtered over 7.5 g. of SiO_2 and eluted firstly with petrolether : EtOAc = 1 : 1 (50 cm^3) followed by EtOAc (50 cm^3). Ethyl acetate fraction was evaporated providing 175 mg of crude reddish-yellow product which was then treated with 1 cm^3 of CH_2Cl_2 Precipitated off-white salt was identified as pure **5a**·HPF_6 (64 mg, 0.14, 14 %).

4a·HPF_6: (from the crude reaction mixture) ^{1}H–NMR (DMSO-d$_6$, 300 MHz) δ/ppm: 2.72 (d, 6H, $^3J_{HH}$ = 4.5 Hz, NCH_3,); 3.73 (s, 3H, -OC$\underline{H}_3$); 3.76 (s, 3H, -OCH_3); 4.04 (d, 2H, $^3J_{HH}$ = 6.0 Hz, -CH_2N$_{gu}$); 5.75 (s, 1H, $^3J_{HH}$ = 1.9 Hz, =C*H*-); 7.23 (d, 1H, $^3J_{HH}$ = 5.2 Hz, -HC=CH-), 7.35–7.37 (dd, 1H, $^3J_{HH}$ = 5.2 Hz, $^3J_{HH}$ =1.8 Hz, -HC=CH-), 7.45-7.60 (m, 3H, NH).

5a·HPF_6: ^{1}H–NMR (DMSO-d$_6$, 600 MHz) δ/ppm: 2.80 (s, 3H, -NCH_3,); 3.08 (s, 3H, -NC$\underline{H}_3$); 3.60 (s, 3H, -OCH_3); 3.68 (s, 3H, -OCH_3); 3.70 (d, 1H, $^3J_{HH}$ = 14.7 Hz, -CH_2N); 3.73 (d, 1H, $^3J_{HH}$ = 4.4 Hz, -C*H*COOMe); 3.87 (d, 1H, $^3J_{HH}$ = 14.7 Hz, -CH_2N); 5.43 (dd, 1H, $^3J_{HH}$ = 4.4 Hz $^3J_{HH}$ = 1.7 Hz, C*H* (oxa-bridge)); 6.25 (d, 1H, $^3J_{HH}$ = 5.6 Hz, -HC=CH-); 6.94 (dd, 1H, $^3J_{HH}$ = 5.6 Hz, $^3J_{HH}$ =1.7 Hz, -HC=CH-); 7.45-7.60 (m, 3H, NH).

5a·HPF_6: ^{13}C–NMR (DMSO-d$_6$, 150.9 MHz) δ/ppm: 28.4, 35.4, 37.8, 52.0, 53.8, 57.7, 74.2, 79.1, 86.2, 131.7, 140.9, 151.9, 167.6, 168.7.

FTIR-ATR ν_{max}/cm^{-1}: 3423 (N-H stretch), 1745 (C=O stretch), 1614 (C=N asymm. stretch), 828 (P-F stretch).

Melting point/ °C: 188-189 (decomp.).

5a·HPF_6: HRMS- Q-TOF found: [MH]$^+$: 310.1404, calculated for $[C_{14}H_{20}N_3O_5]^+$, [MH]$^+$: 310.1397

***N*-[(2,3-dicarbomethoxy-7-oxabicyclo[2.2.1]hepta-2,5-dien-1-yl)methyl]-*N'*,*N''*-diisopropylguanidinium hexafluorophosphate (4b·HPF_6)**

The reaction of **3b**·HPF_6 (228 mg, 0.62 mmol), **DMAD** (455 µl, 626 mg, 3.7 mmol), and 0.62 cm^3 of acetonitrile in a capped microwave vessel under microwave heating (100 °C, 60 min) formed a light reddish-brown mixture. After the removal of the volatile material, a glassy-like reddish-brown solid remained. According to ^1H and ^{13}C NMR data, the crude product mixture was identified as target oxanorbornadiene **4b** containing ca 10 % of the unreacted **DMAD**.

4b·HPF_6: (contaminated with **DMAD**; d = 3.81 ppm)

^1H–NMR (DMSO-d$_6$, 600 MHz) δ/ppm: 1.13 (pseudo triplet, $^3J_{HH}$ = 5.9 Hz, 12H, -CH(CH_3)$_2$); 3.73 (s, 3H, COOCH$_3$); 3.76 (s, 3H, COOCH$_3$); 4.00 – 4.10 (dd, $^3J_{HH}$ = 15.8 Hz, $^3J_{HH}$ = 5.4 Hz, 1H, -N$_{Gu}$CH_aH$_b$–); 4.10 – 4.21 (dd, $^3J_{HH}$ = 15.8 Hz, $^3J_{HH}$ = 6.9 Hz, 1H, -N$_{Gu}$CH$_a$$H_b$–); 5.77 (d, $^3J_{HH}$ = 1.6 Hz, 1H, C*H* oxa-bridge); 7.04 (d, $^3J_{HH}$ = 8.1 Hz, 2H, –NH*i*Pr); 7.23 (d, $^3J_{HH}$ = 5.2 Hz, 1H,=C*H*-C); 7.36 (dd, $^3J_{HH}$ = 5.2 Hz, $^3J_{HH}$ = 1.6 Hz, 1H,=C*H*-C); 7.50 (t, $^3J_{HH}$ = 5.9 Hz, 1H,=C*H*-C). Isopropyl –C*H*(CH$_3$)$_2$ signals are located at 3.70–3.85 ppm and overlap with methyl ester singlets from **4b**·HPF_6 and from **DMAD** contamination.

^{13}C–NMR (DMSO-d$_6$, 150.9 MHz) δ/ppm: 22.2, 43.8, 52.4, 52.5, 54.9, 83.5, 96.6, 143.0, 145.4, 151.3, 154.0, 162.6, 163.5. Signals for CH_3 groups of **DMAD** are located at 53.9 while signals for two quaternary C atoms are hidden in the background noise.

FTIR-ATR ν_{max}/cm^{-1}: 3402 (N-H stretch), 1715 (C=O stretch), 1622 (C=N asymm. stretch), 827 (P-F stretch).

HRMS- Q-TOF found: $[MH]^+$: 366.2031, calculated for $[C_{18}H_{28}N_3O_5]^+$, $[MH]^+$: 366.2023

***N*-[(2,3-dicarbomethoxy-7-oxabicyclo[2.2.1]heptan-1-yl)methyl]-*N'*,*N''*-diisopropylguanidinium hexafluorophosphate** (**6b**·HPF_6)

The crude reaction product of **4b**·HPF_6 obtained from 369 mg of **3d**·HPF_6 as described above (491 mg) was hydrogenated over 203 mg of Pd/C (10 %) in 15 cm^3 of EtOAc under a balloon of H_2.(1 hPa) at the room temperature. After stirring for 24 h, the mixture was filtered over a short column of Celite and washed with additional 20 cm^3 of MeOH. The filtrate was evaporated and the crude product containing ca 10 % of dimethyl succinate was chromatographed. The first fraction was eluted with EtOAc : petrol ether = 1 : 1 (succinate). The hydrogenated product **7b**·HPF_6 was collected as the second fraction eluted with EtOAc. Target oxanorbornane salt was isolated as the white greasy material (366 mg, 0.71 mmol, 71 % over two steps, after correction for the EtOAc and H_2O present in the sample).

6b·HPF_6: ^1H–NMR (CD$_3$CN, 300 MHz) δ/ppm: 1.21 (d, 12H, $^3J_{HH}$ = 6.4 Hz, -CH(CH_3)$_2$); 1.50–1.64 (m, 1H, -CH$_2$CH$_2$-); 1.68-1.84 (m, 1H, -CH$_2$CH$_2$-), 1.90-2.15 (m, 2H, -CH$_2$CH$_2$-); 3.11 (dd, 1H, $^3J_{HH}$ = 11.8 Hz, $^3J_{HH}$ = 2.0 Hz, -CH(exo)); 3.41 (dd, 1H, $^3J_{HH}$ = 11.8 Hz, $^3J_{HH}$ = 5.2 Hz, -CH(exo)); 3.52-3.78 (m, 10H, OCH$_3$ (6H), -CH(CH$_3$)$_2$ (2H), -CH$_2$N$_{gu}$ (2H)); 4.76 (t, 1H, $^3J_{HH}$ = 5.2 Hz, C*H* (oxa-bridge)); 5.85-6.40 (overlapped 2 NH signals: 6.11 (br s, 2H) and 6.20 (t, 1H, $^3J_{HH}$ = 5.6 Hz)). Integration of the NH peaks was done on the spectrum recorded in DMSO-d$_6$ where these signals are baseline separated.

^{13}C–NMR (CD$_3$CN, 75.5 MHz) δ/ppm: 22.6, 22.7, 28.0, 28.1, 45.0, 45.5 (2C overlapped), 49.5, 49.9, 52.5, 52.6, 80.1, 89.8, 155.2 (C_{gv}), 171.7, 171.8.

FTIR-ATR ν_{max}/cm^{-1}: 3407 (N-H stretch), 1738 (C=O stretch), 1624 (C=N asymm. stretch), 826 (P-F stretch).

HRMS- Q-TOF found: $[MH]^+$: 370.2345, calculated for $[C_{18}H_{32}N_3O_5]^+$, $[MH]^+$: 370.2336

***N*-[(2,3-dicarbomethoxy-7-oxabicyclo[2.2.1]hepta-2,5-dien-1-yl)methyl]-*N'*,*N''*-(ethan-1,2-diyl)guanidinium hexafluorophosphate (4c**·HPF_6**)** (crude reaction mixture containing ca 10 % of **5c**·HPF_6)

The reaction of **3c**·HPF_6 (156 mg, 0.5 mmol), **DMAD** (427 mg, 3.0 mmol), and 0.5 cm^3 of acetonitrile in a capped microwave vessel under microwave heating (100 °C, 60 min) formed a brown mixture. Acetonitrile was evaporated under reduced pressure and the crude reaction mixture was washed with 3x 5 cm^3 Et_2O, and then 2x 5 cm^3 EtOAc. After adding 3 cm^3 of MeOH to the remaining material white solid precipitates and the MeOH layer were separated yielding 57 mg of precipitated **5c**·HPF_6. From MeOH mother liquor 100 mg of **5c**·HPF_6 was additionally precipitated upon cooling at -20 °C over 24 h. The total isolated mass was 157 mg, 0.35 mmol, and 70 % yield. ^{1}H NMR spectrum of **4c**·HPF_6 was assigned in the crude reaction mixture before precipitation with methanol and contaminated primarily with **5c**·HPF_6.

4c·HPF_6: ^{1}H–NMR (DMSO-d$_6$, 600 MHz) δ/ppm: 3.59 (s, 4H, -CH_2CH_2-); 3.74 (s, 3H, COOC*H*$_3$); 3.75 (s, 3H, COOC*H*$_3$); 4.00 (dd, 1H, $^3J_{HH}$ = 15.1 Hz, $^3J_{HH}$ = 5.9 Hz, one of -C*H*$_2$N$_{gu}$); 4.05 (dd, 1H, $^3J_{HH}$ = 15.1 Hz, $^3J_{HH}$ = 6.5 Hz, one of -C*H*$_2$N$_{gu}$); 5.78 (d, 1H, $^3J_{HH}$ = 2.0 Hz, C*H* (oxa bridge)); 7.18 (d, 1H, $^3J_{HH}$ = 5.2 Hz, =C*H*-); 7.38 (dd, 1H, $^3J_{HH}$ = 5.2 Hz, $^3J_{HH}$ = 2.0 Hz, =C*H*-); 8.33 (t, 1H, $^3J_{HH}$ = 6.1Hz, -N*H*CH$_2$-). Two NH protons are strongly broadened and hidden in the background.

9,10-dicarbomethoxy-14-oxa-3,6,8-triazatetracyclo[9.2.1.01,9.03,7]tetradec-12-en-7-ylium hexafluorophosphate (5c·HPF_6**)**

5c·HPF_6: ^{1}H–NMR (DMSO-d$_6$, 600 MHz) δ/ppm: 3.54–3.69 overlapping of multiple signals (s, 3H, OCH_3; s, 3H, OCH_3; m, 3H ethano bridge); 3.78 (d, 1H, $^3J_{HH}$ = 4.5 Hz, -C*H*COOMe, overlapped with one of the methylene protons); 3.78 (d, 1H, $^3J_{HH}$ = 14.5 Hz, -C*H*$_2$N$_{gu}$; overlapped with exo proton); 3.91 (d, 1H, $^3J_{HH}$ = 14.9 Hz, -C*H*COOMe, overlapped with one of imidazolidine CH protons); 3.91–3.96 (m, 1H, one of imidazolidine CH protons overlapped with C*H*COOMe); 5.31 (dd, 1H, $^3J_{HH}$ = 4.4 Hz $^3J_{HH}$ = 1.7 Hz, C*H*(oxa-bridge)); 6.28 (d, 1H, $^3J_{HH}$ = 5.7 Hz, -HC=CH-); 6.91 (dd, 1H, $^3J_{HH}$ = 5.6 Hz, $^3J_{HH}$ =1.7 Hz, -HC=CH-); 8.30 (s, 1H, NH); 9.00 (s, 1H, NH).

5c·HPF_6: ^{13}C–NMR (DMSO-d$_6$, 150.9 MHz) δ/ppm: 38.4; 40.5; 46.1; 51.9; 53.7; 55.7; 69.3; 79.6; 86.4; 132-1; 140.0; 153.5;167.0;168.6.

FTIR-ATR ν_{max}/cm^{-1}: 3403 (N-H stretch), 1720 (C=O stretch), 1675 (C=N asymm. stretch), 830 (P-F stretch).

Melting point/ °C: 183-184 (decomp.).

HRMS- MALDI-TOF/TOF found: [M]$^+$: 453.0902, calculated for $C_{14}H_{18}F_6N_3O_5P$, [M]$^+$: 453.0888.

***N*-[(2,3-dicarbomethoxy-7-oxabicyclo[2.2.1]hepta-2,5-dien-1-yl)methyl]-*N'*,*N''*-(propan-1,3-diyl)guanidinium hexafluorophosphate (4d**·HPF_6**)** (crude reaction mixture containing ca 10 % of **5d**·HPF_6) and **10,11-Dicarbomethoxy-15-oxa-3,7,9-triazatetracyclo[10.2.1.0^{1,10}.0^{3,8}]pentadec-13-en-8-ylium hexafluorophosphate (5d**·HPF_6**)**

The reaction of **3d**·HPF_6 (356 mg, 1.1 mmol), **DMAD** (957 mg, 6.7 mmol), and 1.1 cm^3 of acetonitrile in a capped microwave vessel under microwave heating (100 °C, 60 min) formed a brown mixture. The crude reaction mixture was washed with Et_2O (3x 7 cm^3) and the remaining brownish oil was dissolved in a small amount of EtOAc and passed over a short plug of silica gel (9 g). The first fraction was collected using 25 cm^3 of EtOAc containing **DMAD** and small amounts of **4d**·HPF_6, the second fraction was collected by

washing with EtOH (40 cm^3), after removing the solvent under vacuum, 398 mg of the product obtained was identified as **5d**·HPF_6 with some minor impurities and EtOH present in the ^{1}H-NMR spectrum.

5d·HPF_6: due to the strong overlapping of the signals, we were not able to assign the majority of the spectrum. However, peaks in the range of 4.0 – 8.5 ppm undoubtedly confirm polycyclic structure and the absence of **4d**·HPF_6. The product was purified and assigned in its hydrogenated form (see data for **7d**·HPF_6).

10,11-Dicarbomethoxy-15-oxa-3,7,9-triazatetracyclo[10.2.1.0^{1,10}.0^{3,8}]pentadecan-8-ylium hexafluorophosphate (7d·HPF_6**)**

To the above-mentioned sample of 398 mg, MeOH (10 cm^3) and Pd/C 10% (145 mg) were added and reduced in a hydrogen atmosphere (1 atm) at ambient temperature for 18h. Afterward, the crude reaction mixture was filtered through a short plug of Celite and additionally washed with MeOH (40 cm^3). After solvent evaporation under reduced pressure, a yellow oil remained (399 mg) to which 3 cm^3 of EtOAc was added and the organic layer was separated from the undissolved material. From EtOAc, mother liquor at 4°C **7d**·HPF_6 crystallized. The total mass of isolated **7d**·HPF_6 is 337 mg (0.72 mmol, 65% yield).

7d·HPF_6: ^{1}H–NMR (DMSO–d_6, 300 MHz) δ/ppm: 1.45–1.67 (m, 2H, –$CH_2C\mathit{H}_2CH_2$–); 1.74–1.95 (m, 1H, -2H, 2x –$C\mathit{H}_2CH_2$– (two unassigned oxanorbornane protons)); 1.95-2.10 (m, 1H, -$CH_2C\mathit{H}_2$-(one unassigned oxanorbornane proton), 2.21 – 2.36 (m, 1H, -$CH_2C\mathit{H}_2$-(one unassigned oxanorbornane proton); 3.11 – 3.33 (m, 3H, 1,3propylene linker)); 3.44 (d, 1H, $^3J_{HH}$ = 14.5 Hz, -$C\mathit{H}_2$–N_{gu}); 3.54 (d, 1H, $^3J_{HH}$ = 14.5 Hz, -$C\mathit{H}_2$–N_{gu}); 3.57-3.70 (m, 5H, $OC\mathit{H}_3$ (3H), C*H*(exo),(1H), 1,3propylene linker (1H)); 3,78 (s, 3H, OCH_3); 4.84 (t, 1H, $^3J_{HH}$ = 5.3 Hz, -CH(oxa-bridge); 7.98 (br s, 2H, NH).

^{13}C–NMR (DMSO–d_6, 75.5 MHz) δ/ppm: 20.1; 24.8, 29.4, 38.0, 39.1, 43.0, 52.0, 53.9, 57.3, 74.3, 78.3, 84.5, 149.4, 167.9, 168.5.

FTIR-ATR ν_{max}/cm^{-1}: 3406 (N-H stretch), 1734 (C=O stretch), 1613 (C=N asymm. stretch), 830 (P-F stretch).

Melting point/ °C: 195-197.

HRMS- MALDI-TOF/TOF found: $[M]^+$: 469.1200, calculated for $C_{15}H_{22}F_6N_3O_5P$, $[M]^+$: 469.1201.

***N*-[(2,3-dicarbomethoxy-7-oxabicyclo[2.2.1]heptan-1-yl)methyl]-*N'*-Phenyl-*N''*-propylguanidinium hexafluorophosphate (4e**·HPF_6**)**

and

6,7-dicarbomethoxy-5-phenyl-4-propylamino-11-oxa-3,5-diazatricyclo[6.2.1.0^{1,6}]undec-9-en-4-ylium hexafluorophosphate (5e·HPF_6**)**

The reaction of **3e**·HPF_6 (409 mg, 1.0 mmol), **DMAD** (750 µl, 870 mg, 6.0 mmol), and 1.0 cm^3 of acetonitrile in a capped microwave vessel under microwave heating (100 °C, 60 min) formed a light brownish mixture. After the removal of the volatile material under high vacuum conditions (p = 1-4 Pa), obtained viscous material was washed with 3 x 3 cm^3 of diethyl ether. Ethereal layers were removed and the remaining material was dried under a vacuum. According to ^{1}H and ^{13}C NMR data, the crude product mixture (654 mg) was identified as target oxanorbornadiene **4e** containing ca 7 % of cyclized product **5e** and some of

the unreacted **DMAD**. The crude reaction mixture was passed through the short column of silica (7.5 g) and eluted first with a petroleum ether/ethyl acetate mixture (1 : 1; 50 cm^3) and then with EtOAc (50 cm^3) and finally with ethanol (50 cm^3). Both ethyl acetate and ethanol fractions contained the same product (according to ^1H NMR) corresponding to cyclic structure **5e**. The product was crystallized from an EtOAc/Et_2O mixture providing 184 mg (0.34 mmol, 34 %) of the white crystalline **5e**·HPF_6 salt.

5e·HPF_6: ^1H–NMR (DMSO-d$_6$, 600 MHz) δ/ppm: 0.78 (t, 3H, $^3J_{HH}$ = 7.4 Hz, C*H*$_3$); 1.38–1.46 (m, 2H, $CH_3CH_2CH_2$); 3.03–3.13 (m, 2H, $CH_3CH_2CH_2$-N); 3.16 (s, 3H, COOC*H*$_3$); 3.64 (d, 1H, $^3J_{HH}$ = 4.5 Hz, C*H*COO$_{exo}$); 3.76 (s, 3H, COOC*H*$_3$); 3.88 (dd, 1H, $^3J_{HH}$ = 14.9 Hz, $^3J_{HH}$ = 4.0 Hz, C*H*$_2$(furfuryl)); 4.16 (d, 1H, $^3J_{HH}$ = 14.9 Hz, C*H*$_2$ (from furfuryl)); 5.24 (dd, 1H, $^3J_{HH}$ = 4.5 Hz, $^3J_{HH}$ = 1.7 Hz, C*H* (oxa-bridge)); 6.30 (d, 1H, $^3J_{HH}$ = 5.7 Hz, vinylic C*H*); 6.79 (t, 1H, $^3J_{HH}$ = 6.1 Hz, N*H* (endocyclic)); 6.95 (dd, 1H, $^3J_{HH}$ = 5.7 Hz, $^3J_{HH}$ = 1.7 Hz, C*H* (vinylic C*H*)); 7.14–7.17 (m, 1H, Ph); 7.39–7.42 (m, 1H, Ph); 7.53–7.60 (m, 3H, Ph); 8.62 (d, 1H, $^3J_{HH}$ = 3.6 Hz, N*H* (exocyclic)).

^{13}C–NMR (DMSO-d$_6$, 150.9 MHz) δ/ppm: 10.7, 21.5, 38.6, 42.8, 51.3, 53.7, 56.6, 74.4, 79.8, 86.7, 129.0, 130.1, 130.5, 130.7, 131.2, 132.0, 135.9, 141.7, 151.5, 167.7, 168.1

FTIR-ATR ν_{max}/cm^{-1}: 3639 (N-H stretch), 3570 (N-H stretch), 1732 (C=O stretch), 1626 (C=N asymm. stretch), 814 (P-F stretch).

Melting point/ °C: 129-131.

HRMS- Q-TOF found: $[MH]^+$: 400.1869, calculated for $[C_{21}H_{26}N_3O_5]^+$, $[MH]^+$: 400.1867

2,3-bis(methoxycarbonyl)-7-oxabicyclo[2.2.1]heptan-1-yl)methyl)amino)(phenylamino)methylene)propan-1-aminium hexafluorophosphate (6e·HPF_6**)**

The reaction of **3e**·HPF_6 (406 mg, 1.0 mmol), **DMAD** (737 µl, 855 mg, 6.0 mmol), and 1.0 cm^3 of acetonitrile in a capped microwave vessel under microwave heating (100 °C, 60 min) formed a dark yellowish mixture. The solvent was evaporated using a rotary evaporator and **DMAD** was partially removed under a high vacuum. ^1H NMR spectrum of crude reaction mixture shows the major product to be a **4e**·HPF_6 with ca 5% of **5e**·HPF_6 and with ca 2.6 mol-equivalents of **DMAD** still present in the crude product. The mixture was reduced for 18 hours under ambient temperature under a hydrogen atmosphere in 20 cm^3 of ethyl acetate and Pd-catalyst (249 mg of Pd/C 10%). The reaction was filtered over a short plug of Celite and was washed additionally with 20 cm^3 of MeOH. Solvents were evaporated under reduced pressure. The ratio of **4e**·HPF_6 and **5e**·HPF_6 remained essentially unchanged as confirmed by the ^1H NMR spectrum of the crude reaction mixture. **4e**·HPF_6 was purified by column chromatography over 20 g of silica, eluted first with a petroleum ether/ethyl acetate mixture (1 : 2; 30 cm^3) and afterward with ethyl acetate (90 cm^3). In this way, 350 mg (0.64 mmol) of product **6e**·HPF_6 was obtained as a white solid with an overall yield of 64% after two steps.

6e·HPF_6: ^1H–NMR (DMSO-d$_6$, 600 MHz) δ/ppm: 0.88 (t, 3H, $^3J_{HH}$ = 7.4 Hz, C*H*$_3$CH$_2$CH$_2$-); 1.55 (m, 3H, 1H + 2H (-CH$_2$C*H*$_2$CH$_3$); 1.72 (m, 1H); 1.88 (m, 1H); 2.03 (m, 1H); 3.12 (dd, 1H, $^3J_{HH}$ = 11.9 Hz, $^3J_{HH}$ = 5.2 Hz, C*H*COO$_{exo}$); 3.18 (m, 2H, -C*H*$_2$CH$_2$CH$_3$); 3.46 (dd, 1H, $^3J_{HH}$ = 15.2 Hz, $^3J_{HH}$ = 11.7 Hz, C*H*COO$_{exo}$); 3.53 (s, 3H, COOC*H*$_3$); 3.59 (s, 3H, COOC*H*$_3$); 3.71 (m, 2H, C*H*$_2$-NGu); 4.74 (t, 1H, $^3J_{HH}$ = 5.2 Hz, C*H* (oxa-bridge)); 7.10 (m, 1H, Ph); 7.27 (m, 1H, Ph); 7.44 (m, 1H, Ph); 7.78 (br s, 1H, N*H*-Ph); 7.95 (br s, 1H, N*H*-Pr); 9.30 (br s, 1H, N*H*-CH$_2$-oxa-bridge)

^{13}C–NMR (DMSO-d$_6$, 150.9 MHz) δ/ppm: 11.1, 21.8, 27.1, 27.3, 43.4, 43.7, 48.6, 48.8, 51.6, 51.7, 77.9, 87.9, 124.0, 124.5, 126.1, 129.7 (2C overlapping), 136.4, 154.6, 170.5, 170.7

FTIR-ATR ν_{max}/cm^{-1}: 3390 (N-H stretch), 1734 (C=O stretch), 1644 (C=N asymm. stretch), 826 (P-F stretch).

Melting point/ °C: 141-142.

HRMS- Q-TOF found: $[MH]^+$: 404.2186, calculated for $[C_{21}H_{30}N_3O_5]^+$, $[MH]^+$: 404.2185

6,7-dicarbomethoxy-4-ethylamino-5-(2-furanyl)methyl-11-oxa-3,5-diazatricyclo[6.2.1.0^{1,6}]undec-9-en-4-ylium hexafluorophosphate (5f·HPF_6)

Teflon vial for high pressure was charged with **3f**·HPF_6 (172 mg, 0.44 mmol), **DMAD** (71 mg, 0.5 mmol), and DCM (1 cm^3). The reaction mixture was left under high pressure (5-7 kbar) for 6 days. After the reaction solvent was evaporated, two samples of 56 mg and 70 mg of crude product were chromatographed. Each sample was passed over a short column of silica gel (15 g, eluent EtOAc: EtOH= 3:1) and 47 and 56 mg respectively of product **5f**·HPF_6 were collected. The total isolated mass of product **5f**·HPF_6 was 103 mg (0.16 mmol, 75 % yield, corrected for the EtOAc present in the structure. To obtain monocrystals for X-ray diffraction a sample of the product was recrystallized using diffusion of Et_2O vapors to a solution of the product in EtOAc.

The product was isolated by crystallization from ethyl acetate as a solvate. X-ray structural determination revealed cocrystallization of **5f**·HPF_6 and EtOAc in 1 : 1 ratio. The structure is shown in Figure S14c. Solvent coordinates guanidinium cation by carbonyl – HN hydrogen bonding interactions.

5f·HPF_6 (EtOAc solvate): ^{1}H–NMR (CD_3CN, 300 MHz) δ/ppm: 1.16–1.26 (m, 6H, overlapping of two -CH_3 groups from product and from EtOAc); 1.97 (s, 3H, CH_3CO from EtOAc); 3.18–3.32 (m, 2H, -C$H_2$$CH_3$); 3.59 (s, 3H, -COOC$H_3$); 3.68 (s, 3H, -COOC$H_3$); 3.60–3.85 (m, 4H, overlapping of multiple signals); 3.99 (d, 1H, $^3J_{HH}$ = 14.7 Hz, one of endocyclic methylene protons from the 2-aminopyrimidine ring); 4.06 (q, 2H, $^3J_{HH}$ = 7.1 Hz, -C$H_2$$CH_3$ from EtOAc); 4.85 (d, 2H, $^3J_{HH}$ = 2.4 Hz, -CH_2Furan); 5.22 (dd, 1H, $^3J_{HH}$ = 4.2 Hz, $^3J_{HH}$ = 1.3 Hz, C*H* (oxa bridge)); 6.14 (d, 1H, $^3J_{HH}$ = 5.7 Hz, Furan C(3)*H*); 6.25 (br s, 1H NH); 6.35–6.47 (m, 2H, overlapping of =CH- and Furan C(4)*H*); 6.70 (br s, 1H, NH); 7.00 (dd, 1H, $^3J_{HH}$ = 5.7 Hz, $^3J_{HH}$ = 1.6 Hz, =C*H*-); 7.53 (d, 1H, $^3J_{HH}$ = 0.9 Hz, Furan C(5)*H*).

5f·HPF_6 (EtOAc solvate): ^{13}C–NMR (CD_3CN, 75.5 MHz) δ/ppm:14.0, 14.5 (EtOAc), 21.2 (EtOAc), 38.2, 39.2, 46.1, 53.0, 54.6, 58.7, 61.0 (EtOAc), 75.9, 80.9, 87.3, 111.0, 111.8, 132.1, 143.0, 144.7, 148.6, 152.6, 168.4, 169.6, 171.7 (EtOAc)

FTIR-ATR ν_{max}/cm^{-1}: 3415 (N-H stretch), 1743 (C=O stretch), 1626 (C=N asymm. stretch), 827 (P-F stretch)

Melting point/ °C: 55 (dissolution in EtOAc present as the crystal solvent).

HRMS- Q-TOF found: $[MH]^+$: 390.1669, calculated for $[C_{19}H_{24}N_3O_6]^+$, $[MH]^+$: 390.1665

S1.3. Aza-Michael reactions

General procedure for tandem Aza-Michael/Cyclization reaction to DMAD: 1 mol-eq of the guanidinium salt was weighed to a microwave vessel equipped with a magnetic stirrer and 1 cm^3 of acetonitrile per 1 mmol of guanidinium salt was added and briefly stirred. To this mixture, 2 mol-eq of **DMAD** was added and the vessel was capped. The reaction mixture was stirred either at room temperature for 48 h or at 100 °C for 1 h.

(2*Z*)-methyl 2-((Z)-3-((furan-2-yl)methyl)-1-isopropyl-2-(isopropylimino)-5-oxoimidazolidin-4-ylidene)acetate (*Z*–9b) and

(2*E*)-methyl 2-((*Z*)-3-((furan-2-yl)methyl)-1-isopropyl-2-(isopropylimino)-5-oxoimidazolidin-4-ylidene)acetate (*E*–9b)

The reaction of **3b**·HCl (260 mg, 1.0 mmol), **DMAD** (0.250 cm^3, 290 mg, 2.0 mmol), and 1 cm^3 of acetonitrile in a capped microwave vessel under microwave heating (100 °C, 60 min) formed a reddish-brown mixture. The crude reaction mixture was evaporated and passed through the short column of silica using EtOAc as the eluent. The collected crude material (325 mg) was chromatographed again using the mixture of petroleum ether : EtOAc = 2 : 1. Three main fractions were separated. The first fraction (R_f = 0.69) was identified as the product *Z*–**9b** (71 mg, 0.21 mmol, 21 %). The second fraction (R_f = 0.56) corresponds to 2-chlorofumarate (**8Cl**, 76 mg, 43 %). The third fraction (R_f = 0.45) contains the product *E*–**9b** (33 mg, 0.10 mmol, 10 %). The remaining material was eluted either with EtOAc or with MeOH and contained a mixture of various unidentifiable compounds. The products *Z*– and *E*–**9b** were recrystallized from the hexane providing sufficiently pure white crystalline products suitable for the X-ray analysis.

Z–**9b**: ^{1}H–NMR ($CDCl_3$, 600 MHz) *δ*/ppm: 1.22 (d, 6H, $^3J_{HH}$ = 6.1 Hz, -CH(C$\underline{H_3}$)$_2$); 1.42 (d, 6H, $^3J_{HH}$ = 6.9 Hz, -CH(C$\underline{H_3}$)$_2$); 3.74 (s, 3H, OC*H*$_3$); 4.21 (hep, 1H, $^3J_{HH}$ = 6.1 Hz, -C$\underline{H}$(CH$_3$)$_2$); 4.53 (hep, 1H, $^3J_{HH}$ = 6.9 Hz, -C$\underline{H}$(CH$_3$)$_2$); 5.39 (s, 2H, NC*H*$_2$Furan); 5.72 (s, 1H, =C*H*-COO); 6.06 (d, 1H, $^3J_{HH}$ = 3.2 Hz, Furyl C4H); 6.24–6.25 (dd, 1H, $^3J_{HH}$ = 3.2 Hz, $^3J_{HH}$ =1.9 Hz, Furyl C3H); 7.27 (d, 1H, $^3J_{HH}$ = 1.9 Hz, Furyl C2H).

^{13}C–NMR ($CDCl_3$, 75.5 MHz) *δ*/ppm: 19.4 (-CH$\underline{Me_2}$), 25.2 (-CH$\underline{Me_2}$), 43.3 (broad, -*C*H$_2$Furyl), 45.5 (broad, -$\underline{C}$HMe$_2$), 47.4 (-$\underline{C}$HMe$_2$), 51.8 (COO$\underline{Me}$), 93.4 (exocyclic vinyl $\underline{C}$(H)COOMe), 109.0 (Furyl C4), 110.2 (Furyl C3), 138.6 (N-C(N)=N), 141.1 (endocyclic vinyl $\underline{C}$=C(H)COOMe), 142.7 (Furyl C2), 149.6 (Furyl C5), 163.1 (C=O), 166.2 ($\underline{C}$OOMe).

FTIR-ATR ν_{max}/cm^{-1}: 2966 (C-H stretch), 1736 (C=O stretch), 1690 (C=N asymm. stretch), 1634 (C=N asymm. stretch).

Melting point/ °C: 57-59.

HRMS- Q-TOF found: [MH]$^+$: 334.1777, calculated for $[C_{17}H_{24}N_3O_4]^+$, [MH]$^+$: 334.1761

E–**9b**: ^{1}H–NMR ($CDCl_3$, 300 MHz) *δ*/ppm: 1.18 (d, 6H, $^3J_{HH}$ = 6.1 Hz, -CH(C$\underline{H_3}$)$_2$); 1.43 (d, 6H, $^3J_{HH}$ = 6.8 Hz, -CH(C$\underline{H_3}$)$_2$); 3.77 (s, 3H, OC*H*$_3$); 4.18 (hep, 1H, $^3J_{HH}$ = 6.1 Hz, -C$\underline{H}$(CH$_3$)$_2$); 4.53 (hep, 1H, $^3J_{HH}$ = 6.8 Hz, -C$\underline{H}$(CH$_3$)$_2$); 4.74 (s, 2H, NC*H*$_2$Furan); 5.35 (s, 1H, =C$\underline{H}$-COO); 6.23 (d, 1H, $^3J_{HH}$ = 3.1 Hz, Furyl C4H); 6.33–6.35 (dd, 1H, $^3J_{HH}$ = 3.1 Hz, $^3J_{HH}$ =1.9 Hz, Furyl C3H); 7.38 (br s, 1H, Furyl C2H).

^{13}C–NMR ($CDCl_3$, 75.5 MHz) *δ*/ppm: 19.5 (-CH*Me*$_2$), 25.8 (-CH*Me*$_2$), 42.2 (broad), 45.2 (broad), 46.4; 52.2 (COO*Me*), 95.4 (exocyclic vinyl ***C***(H)COOMe), 108.3 (Furyl C4), 110.7 (Furyl C3), 136.2, 140.2, 142.8 (Furyl C2), 148.9 (Furyl C5), 160.2 (C=O), 165.9 (*C*OOMe).

FTIR-ATR ν_{max}/cm^{-1}: 2970 (C-H stretch), 1748 (C=O stretch), 1688 (C=N asymm. stretch), 1632 (C=N asymm. stretch).

Melting point/ °C: 67-68.

HRMS- Q-TOF found: $[MH]^+$: 334.1777, calculated for $[C_{17}H_{24}N_3O_4]^+$, $[MH]^+$: 334.1761

(2*Z*)-Methyl 2-(1-((furan-2-yl)methyl)-1,2,5,6-tetrahydro-2-oxo-1H-imidazo[1,2-a]imidazol-3-ylidene) acetate (10c),

methyl 8-((furan-2-yl)methyl)-2,3,5,8-tetrahydro-5-oxoimidazo[1,2-a]pyrimidine-7-carboxy-late (11) and

methyl 8-((furan-2-yl)methyl)-2,3,7,8-tetrahydro-7-oxoimidazo[1,2-a]pyrimidine-5-carboxy-late (12)

The reaction of **3c**·HI (293 mg, 1.0 mmol) and **DMAD** (0.260 cm^3, 302 mg, 2.1 mmol) in 1 cm^3 of acetonitrile during 5 days resulted in a dark reddish-brown mixture showing three dominant spots on TLC (EtOAc as eluent). The reaction mixture was evaporated and transferred to the short column of silica and eluted with hexane (elutes iodofumarate **8I**, first spot, F1 (202 mg, 0.75 mmol, 75 %). The second fraction (F2, 180 mg, 0.65 mmol, 65 %) was eluted with ethyl acetate (two other spots that contain a mixture of three components of the same molecular mass (according to GC-MS). The third fraction was eluted with ethanol and contains some unreacted guanidine contaminated with the unidentified polymeric material (F3, 56 mg). This quick filtration over SiO_2 allowed us for rough separation of the components as well as the removal of the polymer formed during the reaction. The ethyl acetate fraction was chromatographed again on a larger column of silica using ethyl acetate as eluent. The second chromatography of fraction F2 provided triazabicyclooctane derivative **10c** (58 mg, 0.21 mmol, 21 %) and a mixture of two components that elute practically together (107 mg). These components were identified as triazabicyclononane derivatives **11** and **12** based on the comparison of the experimental and calculated NMR data. The mixture of **11** and **12** was still contaminated with ca 10 % of some unidentified material.

10c: ^1H–NMR ($CDCl_3$, 300 MHz) *δ*/ppm: 3.71 (s, 3H, OC*H*$_3$); 4.12–4.30 (m, 4H, -C*H*$_2$C*H*$_2$-); 4.84 (s, 2H, furyl-C*H*$_2$-N); 5.58 (s, 1H, =C*H*-); 6.28–6.34 (dd, 1H, $^3J_{HH}$ = 3.0 Hz, $^3J_{HH}$ =1.9 Hz, Furyl C4H); 6.39 (d, 1H, $^3J_{HH}$ = 3.0 Hz, Furyl C3H); 7.36 (d, 1H, $^3J_{HH}$ = 1.9 Hz, Furyl C2H).

^{13}C–NMR ($CDCl_3$, 75.5 MHz) *δ*/ppm: 37.0, 48.2, 51.5, 57.3, 89.8 (=*C*H-), 109.7, 110.7, 137.1, 143.0, 148.0, 155.0 (C_{gv}), 166.0, 166.2.

FTIR-ATR ν_{max}/cm^{-1}: 2952 (C-H stretch), 1749 (C=O stretch), 1692 (C=N asymm. stretch), 1634 (C=N asymm. stretch).

Melting point/ °C: 119-121.

HRMS- Q-TOF found: $[MH]^+$: 276.0985, calculated for $[C_{13}H_{14}N_3O_4]^+$, $[MH]^+$: 276.0979

11: (deduced from the mixture with ca 22 % of **12**) ^{1}H–NMR ($CDCl_3$, 600 MHz) δ/ppm: 3.87 (s, 3H, OCH_3); 3.90–3.96 and 3.98-4.05 (m, 2H + m, 2H, -CH_2CH_2-); 5.34 (s, 2H, furyl-CH_2-N); 5.82 (s, 1H, =C*H*-); 6.26 (d, 1H, $^3J_{HH}$ = 3.0 Hz, Furyl C3H); 6.29–6.31 (m, 1H, Furyl C4H); 7.34 (br.s., 1H, Furyl C2H).

^{13}C–NMR ($CDCl_3$, 150.9 MHz) δ/ppm: 42.5, 45.1, 50.2, 53.5, 104.7 (=*C*H-), 109.6, 110.5, 143.1, 143.3, 149.5, 152.3 (C_{gv}), 159.4, 162.3.

12: (deduced from the mixture with ca 78 % of **11**) ^{1}H–NMR ($CDCl_3$, 600 MHz) δ/ppm**:** 3.88 (s, 3H, OCH_3); 3.94 and 4.28 (t, 2H, $^3J_{HH}$ = 9.7 Hz + t, 2H, $^3J_{HH}$ = 9.1 Hz, -CH_2CH_2-); 5.08 (s, 2H, furyl-CH_2-N); 6.02 (s, 1H, =C*H*-); 6.29–6.30 (m, 1H, Furyl C4H); 6.38 (d, 1H, $^3J_{HH}$ = 3.0 Hz, Furyl C3H); 7.33 (br.s., 1H, Furyl C2H).

^{13}C–NMR ($CDCl_3$, 150.9 MHz) δ/ppm **:** 38.8, 50.1, 51.5, 53.5, 101.5 (=*C*H-), 109.4, 110.5, 139.7, 142.2, 149.7, 152.8 (C_{gv}), 161.6, 161.9.

(2*Z*)-Methyl 2-(1-((furan-2-yl)methyl)-1,2,6,7-tetrahydro-2-oxoimidazo[1,2-*a*]pyrimidin-3(5*H*)-ylidene) acetate (9d) and

(2*Z*)-methyl 2-(1-((furan-2-yl)methyl)-6,7-dihydro-3-oxoimidazo[1,2-a]pyrimidin-2(1*H*,3*H*,5*H*)-ylidene) acetate (10d)

The reaction of **3d**·HI (154 mg, 0.5 mmol), **DMAD** (0.123 cm^3, 142 mg, 1.0 mmol), and 0.5 cm^3 of acetonitrile in a capped microwave vessel was stirred at room temperature for 14 days (336 h). Products were purified on column chromatography over silica gel (45 g) using EtOAc : petroleum ether 2 : 1 as eluent. The total mass of the two products is 43 mg (0.15 mmol, 30 % yield). ^{1}H-NMR of crude reaction mixture shows 0.4:1 ratios **9d** : **10d**. Practically the same ratio has been obtained if the reaction was heated in a MW reactor at 100 °C for 1h (0.5 : 1) or stirred for 48 h (0.6 : 1) indicating an absence of interconversion between the products.

Additional purification could be performed by selective crystallization of the chromatographed mixture of products from hexane. To the 132 mg of a chromatographed mixture of products, 10 cm^3 of hexane was added, heated until boiling, and separated while hot from undissolved material. The hexane mother liquor was left at room temperature, 61 mg (0.11 mmol, 21 %) of product **9d** crystallized from the solution, and the second crystallization at 4°C gave 24 mg (0.04 mmol, 8%) of **10d**. ^{1}H-NMR of undissolved material from hexane heating (27 mg) shows the presence of a 0.1:1 ratio of **9d** : **10d** with some minor polymeric impurities. The remaining hexane mother liquor gave 18 mg of 0.8:1 mixture of **9d** : **10d** with some minor unidentified impurities.

9d: ^{1}H–NMR ($CDCl_3$, 600 MHz) δ/ppm: 1.87–1.92 (m, 2H, $CH_2CH_2CH_2$); 3.66 (t, 2H, $^3J_{HH}$ = 5.6 Hz, N$CH_2CH_2CH_2$NCO); 3.68–3.71 (m, 2H, N$CH_2CH_2CH_2$NCO); 3.73 (s, 3H, COOCH_3); 5.48 (s, 2H, furyl–CH_2N); 5.74 (s, 1H, C*H* vinyl); 6.14 (dd, 1H, $^3J_{HH}$ = 3.2 Hz, $^3J_{HH}$ = 0.6 Hz, Furyl C3*H*); 6.25 (dd, 1H, $^3J_{HH}$ = 3.3 Hz, $^3J_{HH}$ = 1.8 Hz, Furyl C4*H*); 7.28 (dd, 1H, $^3J_{HH}$ = 1.8 Hz, $^3J_{HH}$ = 0.7 Hz, Furyl C2*H*);

^{13}C–NMR ($CDCl_3$, 150.9 MHz) δ/ppm: 20.4, 38.4, 39.1, 44.7, 51.8, 93.3, 109.0, 110.2, 137.2, 142.5, 145.1, 150.0, 161.8, 166.0

FTIR-ATR ν_{max}/cm^{-1}: 2954 (C-H stretch), 1742 (C=O stretch), 1693 (C=N asymm. stretch), 1641 (C=N asymm. stretch).

Melting point/ °C: 105-107.

10d: ^{1}H–NMR ($CDCl_3$, 600 MHz) δ/ppm: 1.91–1.96 (m, 2H, $CH_2CH_2CH_2$); 3.58 (dd, 2H, $^{3}J_{HH}$ = 5.7 Hz, $^{3}J_{HH}$ = 5.5 Hz, $NCH_2CH_2CH_2NCO$); 3.71 (s, 3H, $COOCH_3$); 4.14 (pseudo t, 2H, $^{3}J_{HH}$ = 6.1 Hz, $NCH_2CH_2CH_2NCO$); 4.78 (s, 2H, furyl–CH_2N); 5.68 (s, 1H, C*H* vinyl); 6.29 (dd, 1H, $^{3}J_{HH}$ = 3.2 Hz, $^{3}J_{HH}$ = 1.8 Hz, Furyl C4*H*); 6.33 (dd, 1H, $^{3}J_{HH}$ = 3.3 Hz, $^{3}J_{HH}$ = 0.6 Hz, Furyl C3*H*); 7.33 (dd, 1H, $^{3}J_{HH}$ = 1.8 Hz, $^{3}J_{HH}$ = 0.7 Hz, Furyl C2*H*);

^{13}C–NMR ($CDCl_3$, 150.9 MHz) δ/ppm: 21.7, 35.6, 43.6, 44.6, 51.7, 91.5, 109.1, 110.6, 138.3, 142.5, 144.4, 148.9, 162.3, 165.9

FTIR-ATR ν_{max}/cm^{-1}: 2954 (C-H stretch), 1748 (C=O stretch), 1692 (C=N asymm. stretch), 1649 (C=N asymm. stretch).

Melting point/ °C: 134-136.

HRMS- Q-TOF found: $[MH]^+$: 290.1144, calculated for $[C_{14}H_{16}N_3O_4]^+$, $[MH]^+$: 290.1135

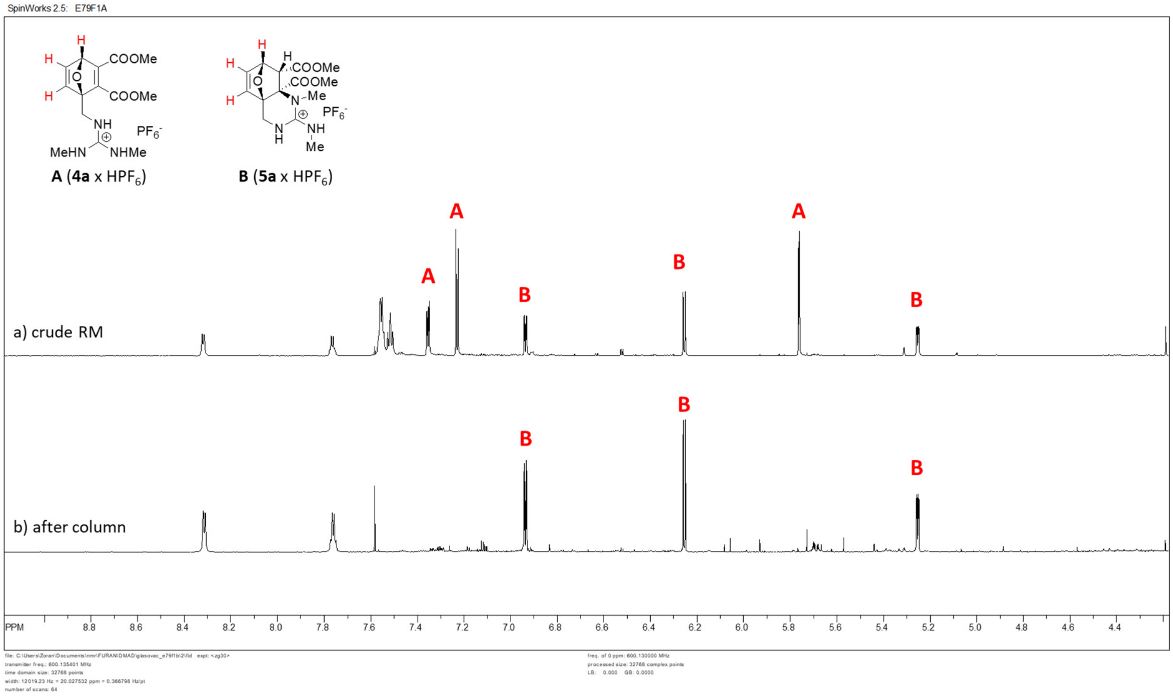

Figure S1. ^{1}H NMR spectra (DMSO-d_6) of a) crude RM and b) after passing over a short column of SiO_2 using EtOAc as eluent.

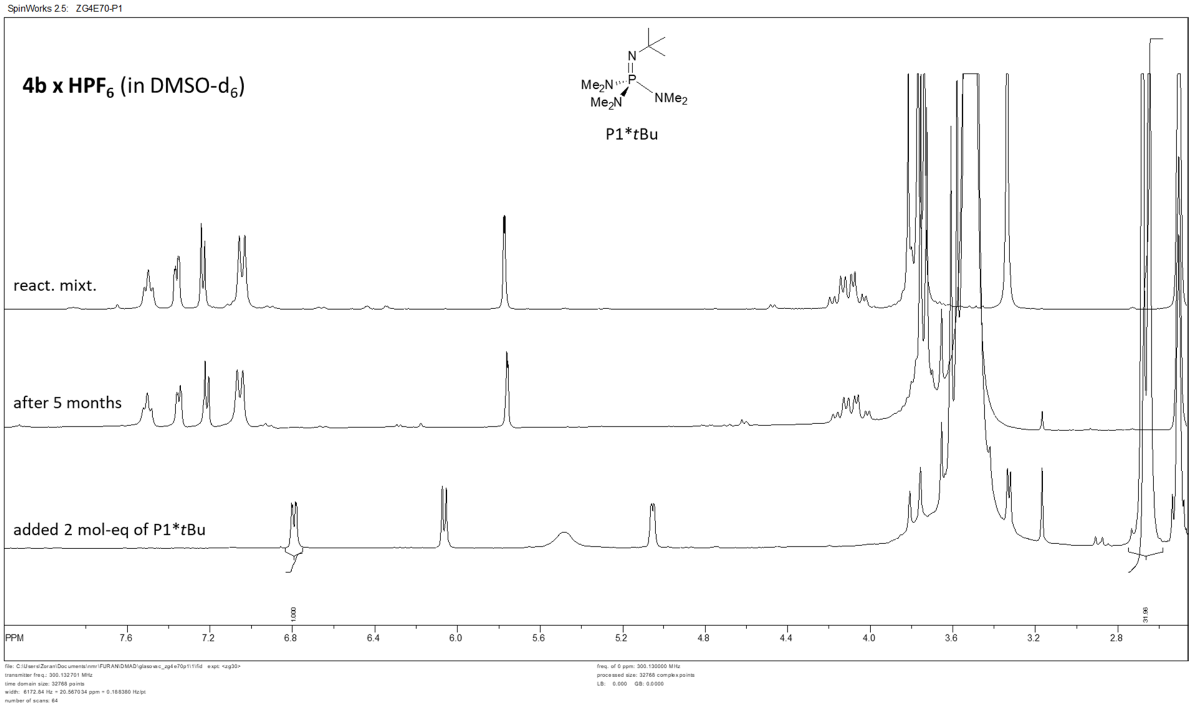

Figure S2. ^{1}H NMR spectra (DMSO-d_6) of a) freshly prepared **4b**·HPF_6, crude RM; b) **4b**·HPF_6 after 5 months in NMR tube; c) after addition of P1**t*Bu.

S2. NMR analysis of structures of products

a) 2D NMR Analysis of the structure of CA/IMC products

We have employed 2D NMR techniques for the analysis of the stereochemistry of intramolecular cyclization. All products of the **5x** type have the same stereochemistry as evidenced by doublet located between 3.0 and 4.0 ppm as indicated in the main text. Additional proofs of syn/exo addition came from NOESY spectra exemplified by that of **5e**·HPF_6 shown in Figure S3. Although weak, off-diagonal peaks indicating through space H–H coupling between exo bridgehead and oxa bridge hydrogen atoms is visible (interaction A). A coupling between one phenyl hydrogen and the exo bridgehead one also confirms their syn position (B). Position of the propyl group is also indicated by through space interaction of the NH hydrogens with the α-methylene protons (C and D).

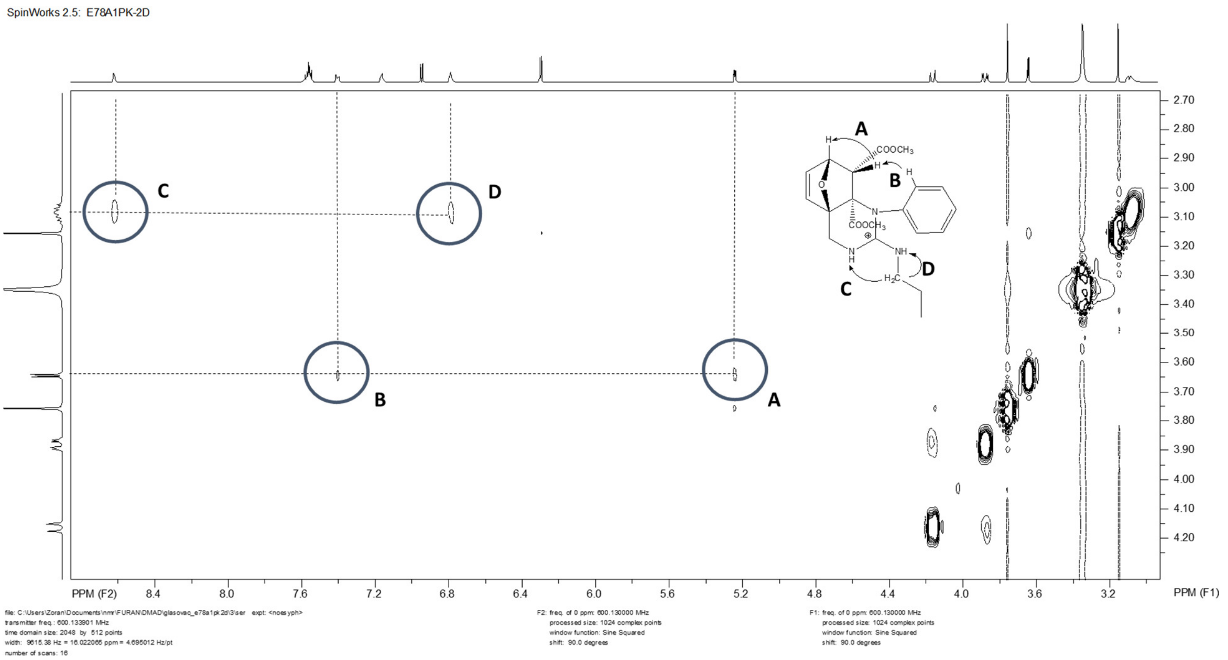

Figure S3. 2D NMR (NOESY, DMSO-d_6) spectrum of guanidine derivative **5e**·HPF_6.

Additional evidence for the assigned structure was obtained from $^1H,^{13}C$–HMBC spectra in which signals corresponding to the coupling across 2 or 3 bonds to guanidine carbon atom were observed. Interestingly, no coupling of phenyl hydrogen atoms with carbons outside the phenyl ring was observed. This, along with the large separation of two signals of *ortho* hydrogens in ^1H spectra and above–mentioned NOESY results indicates the proximity of the rigid part of the molecule and confirms the assigned structure.

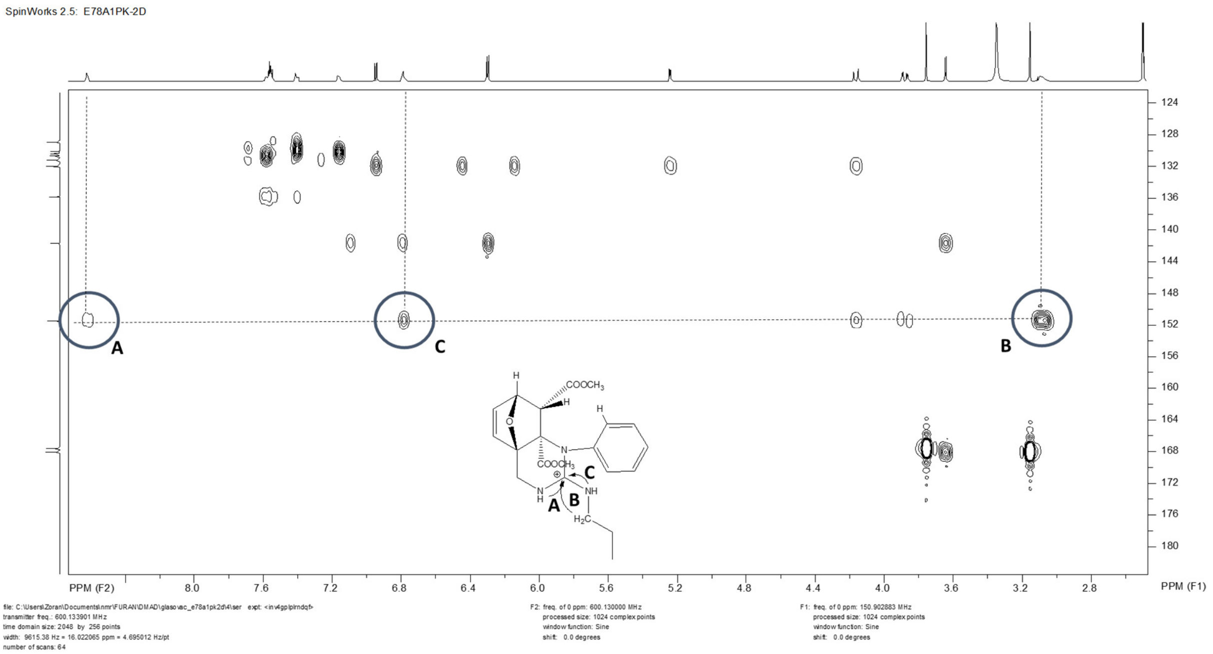

Figure S4. 2D NMR (^1H,^{13}C HMBC, DMSO-d$_6$) spectrum of guanidine derivative **5e**·HPF_6.

*b) 2D NMR Structural assignation of **AMA/CYC** products*

In combination with the X-ray structure, ^1H,^{13}C HMBC spectra revealed the regiochemistry of the **AMA/CYC** products obtained in the mixtures. For that purpose, heteronuclear correlation spectra were recorded for the separated (*Z*/*E*)-**9b** and **10c** derivatives, and the characteristic correlation peaks were used for the assignation of the spectra in the mixture.

Observed peaks that correspond to the H–C coupling through 3 or 4 bonds, clearly indicate the position of the furfuryl group to the carbonyl group and the endocyclic carbon atom of the vinylic group. The same motif of the long-range coupling was observed for **10c** and **10d** which implies the same configuration. Thus, coupling between furfuryl methylene protons and either carbonyl or vinylic carbon is visible if it occurs across three bonds but not across four bonds. Due to the similarity in observed coupling and with the help of the determined crystal structure of **10c**, we assigned the structure of **10d**. The lower-yielding isomer **9d** was assigned because of the observed correlation peak between furfuryl methylene protons with the endocyclic vinylic carbon. Such interaction was also observed in the case of *Z*-**9b** for which the crystal structure was determined.

The distinction between 2-aminoimidazolidinone derivatives (**9(b,d)** and **10(c,d)**) from 2-aminopyrimidinone derivatives (**11** and **12**) is visible in their ^{13}C spectra where the former type of derivatives has the characteristic signal of the exocyclic vinylic carbon atom located at 85-95 ppm. On the other hand, derivatives **11** and **12** have the vinylic signal located at 100-105 ppm which is characteristic of the endocyclic carbon atom of the 2-aminopyrimidinone type [2]. Again, the position of the furfuryl fragment was assigned from the heteronuclear correlation peaks as shown in Figure S4. No correlation

peak between furfuryl methylene protons and the carbon atom of the carbonyl group was observed if they a separated by 5 bonds.

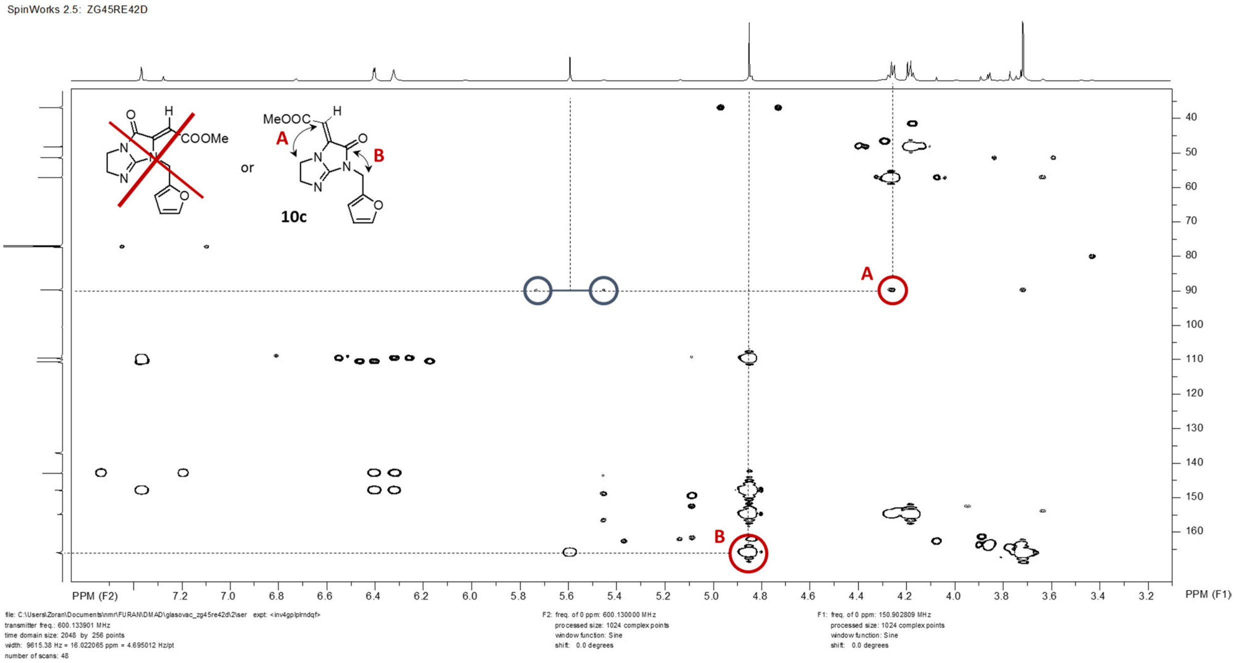

Figure S5. 2D NMR (^{1}H,^{13}C-HMBC, $CDCl_3$) assignment of the structure of **10c**.

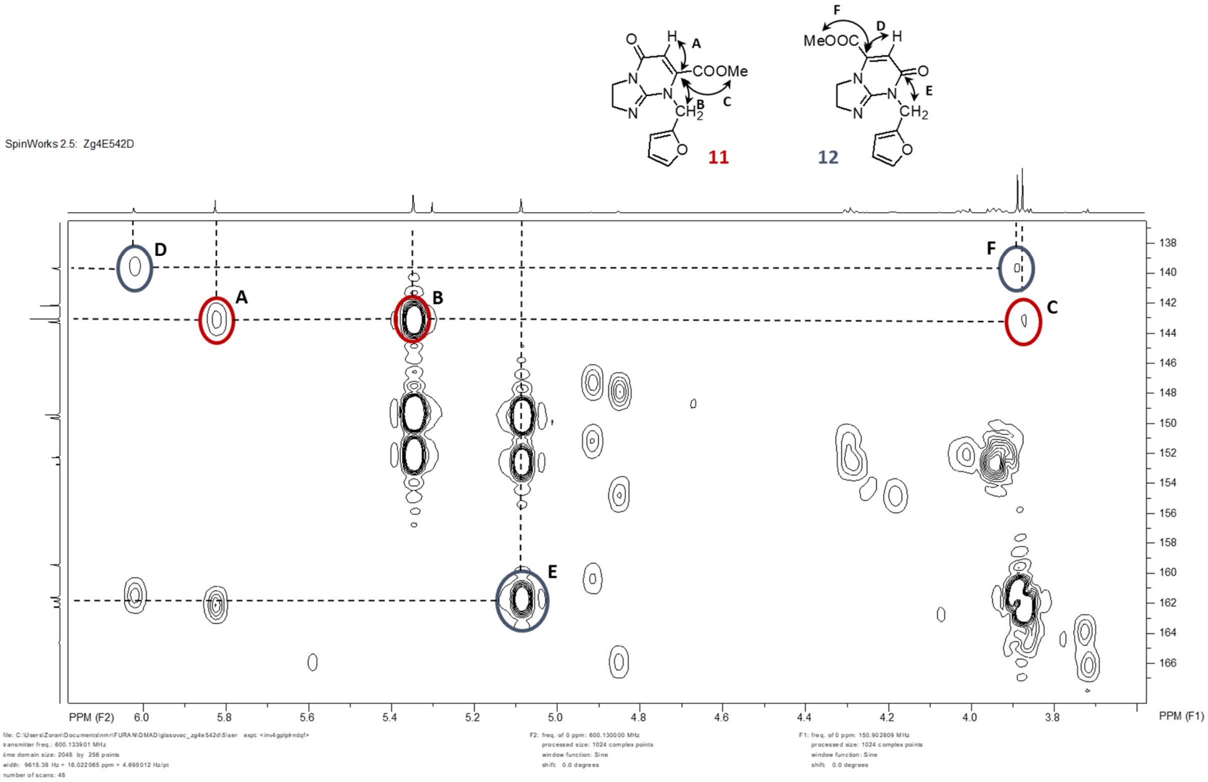

Figure S6. 2D NMR (^{1}H,^{13}C-HMBC, $CDCl_3$) assignment of the structures of **11** and **12**

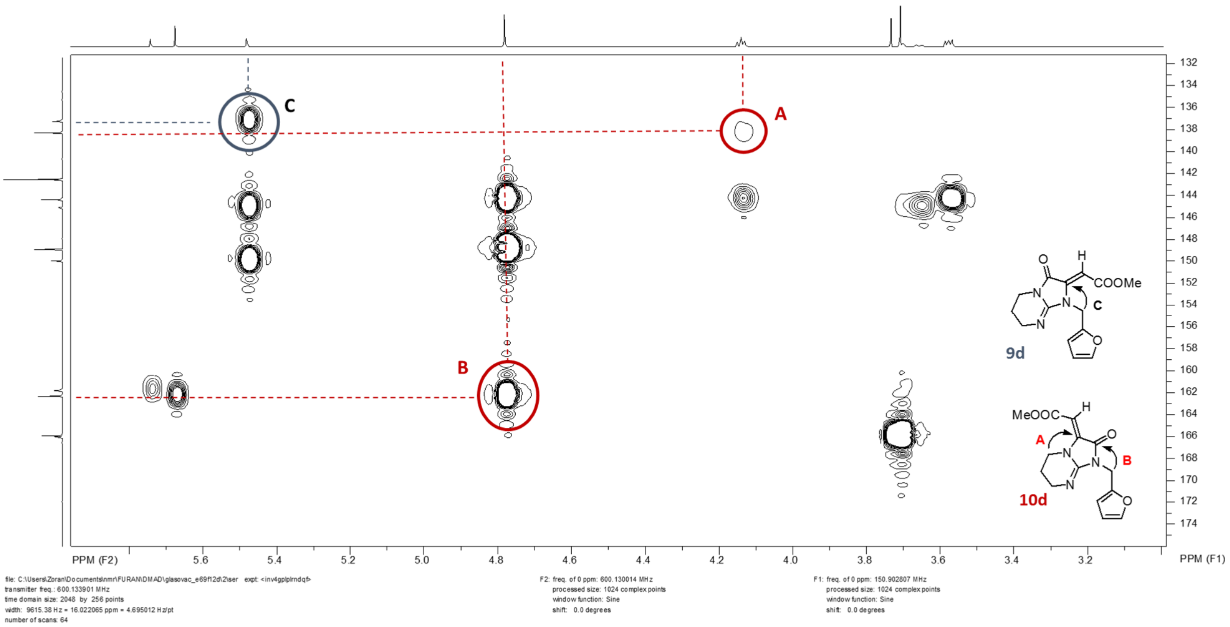

Figure S7. 2D NMR (^{1}H,^{13}C-HMBC, $CDCl_3$) assignment of the structure of **9d** and **10d**.

*b) NMR calculations of the possible isomers obtained by the reaction of **3c** HI with **DMAD**:*

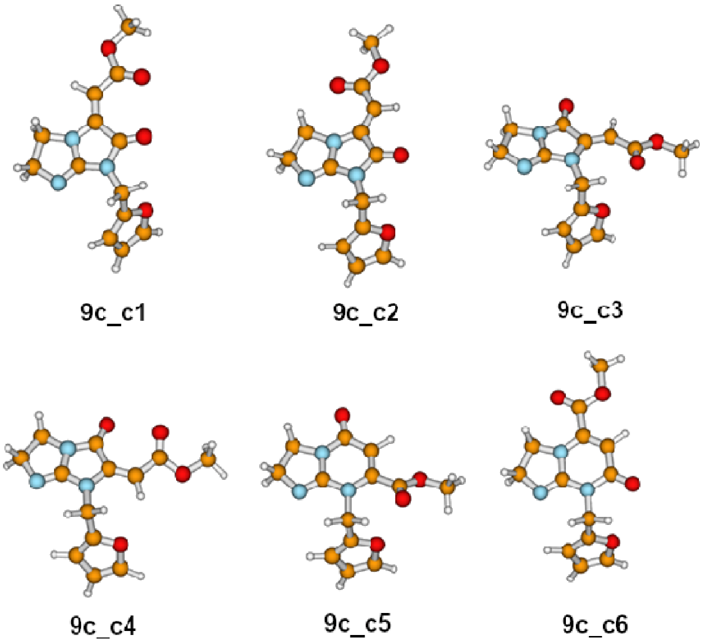

Figure S8. Structures of all regioisomers possibly formed in the aza-Michael reaction.

Table S1. Relative Gibbs energies (G_{rel}) of all possible regioisomeric products of the aza-Michael addition of **3c**·HI to **DMAD**.[a]

Structure	E_{scf}	G_{corr}	G_{tot}	ΔG_{rel} [b]
**9c_c1**	-968.62582	0.20536	-968.42046	20
**9c_c2 (10c)**	-968.63494	0.20681	-968.42813	0
**9c_c2b**[c]	-968.63495	0.20680	-968.42815	0
**9c_c3 (9c)**	-968.62926	0.20798	-968.42128	18
**9c_c4**	-968.62342	0.20631	-968.41711	29
**9c_c5 (11)**	-968.63425	0.20912	-968.42513	8
**9c_c6 (12)**	-968.63556	0.20783	-968.42773	1

[a] Energies, $G_{corr,}$ and G_{tot} were given in a.u. ΔG_{rel} is given in kJ mol^{-1}.

[b] Despite being relatively high in energy (the red marked values), structures **9c_c1**, **9c_c3** and **9c_c4** show also poorer agreement with the experimental NMR data. Structure **9c_c2** shows a slightly higher R^2 value than **9c_c2b** (0.994 vs. 0.989) implying little impact of furan orientation.

[c] X-ray geometry was used as the initial point for the optimization.

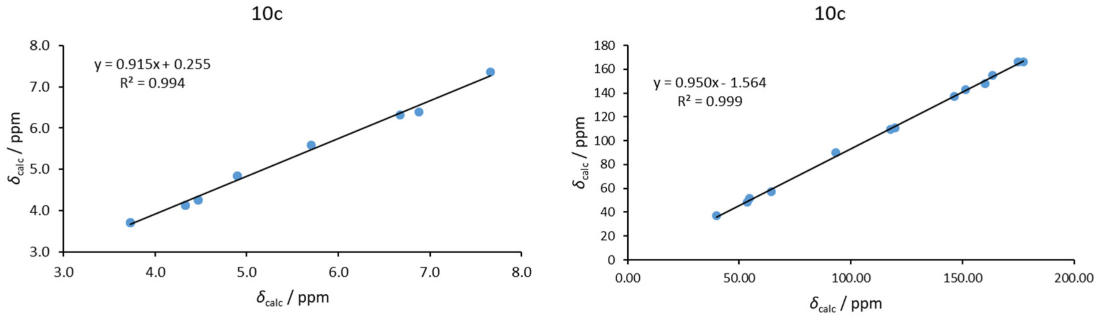

Figure S9a. Correlation of the experimental against calculated ^{1}H (left) and ^{13}C (right) chemical shifts for **10c**.

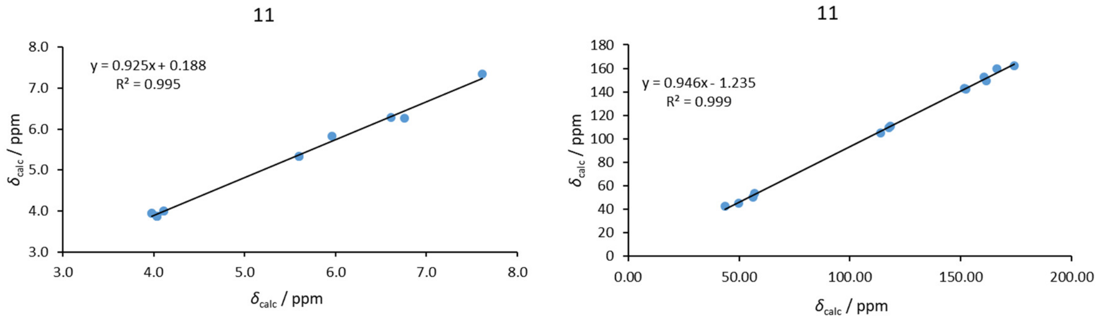

Figure S9b. Correlation of the experimental against calculated ^{1}H (left) and ^{13}C (right) chemical shifts for **11**.

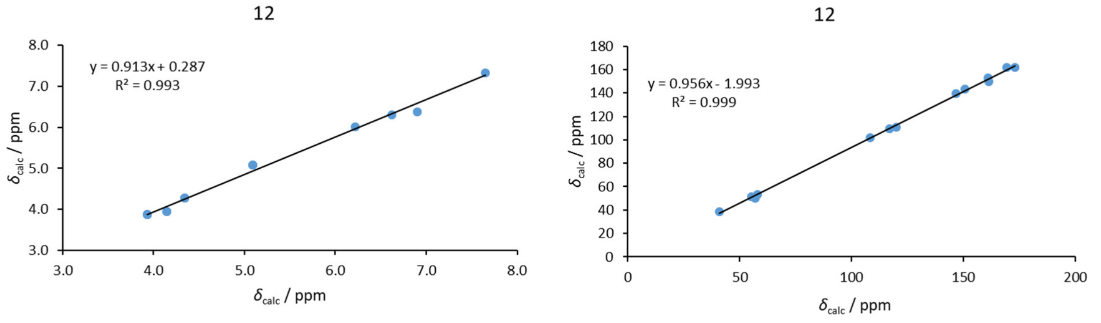

Figure S9c. Correlation of the experimental against calculated ^{1}H (left) and ^{13}C (right) chemical shifts for **12**.

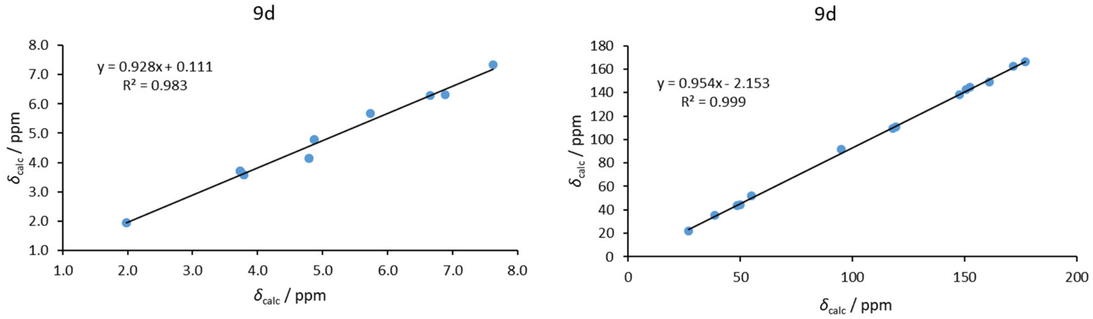

Figure S10a. Correlation of the experimental against calculated ^{1}H (left) and ^{13}C (right) chemical shifts for **9d**.

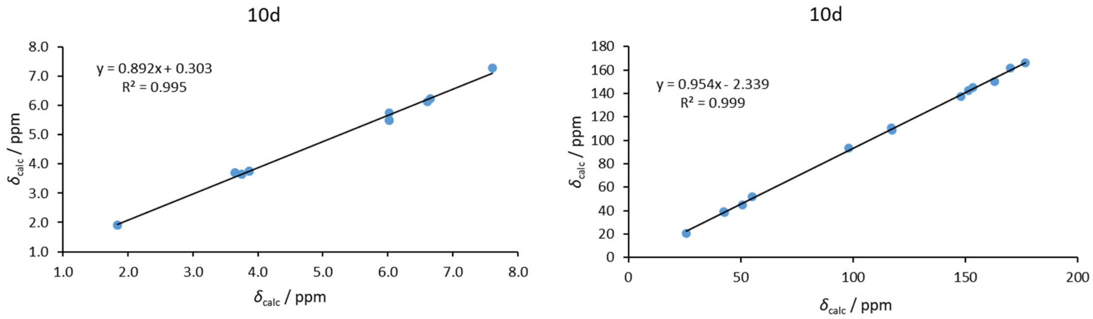

Figure S10b. Correlation of the experimental against calculated ^{1}H (left) and ^{13}C (right) chemical shifts for **10d**.

*c) GC-MS data for the **AMA/CYC** products obtained from **3c**·HI.*

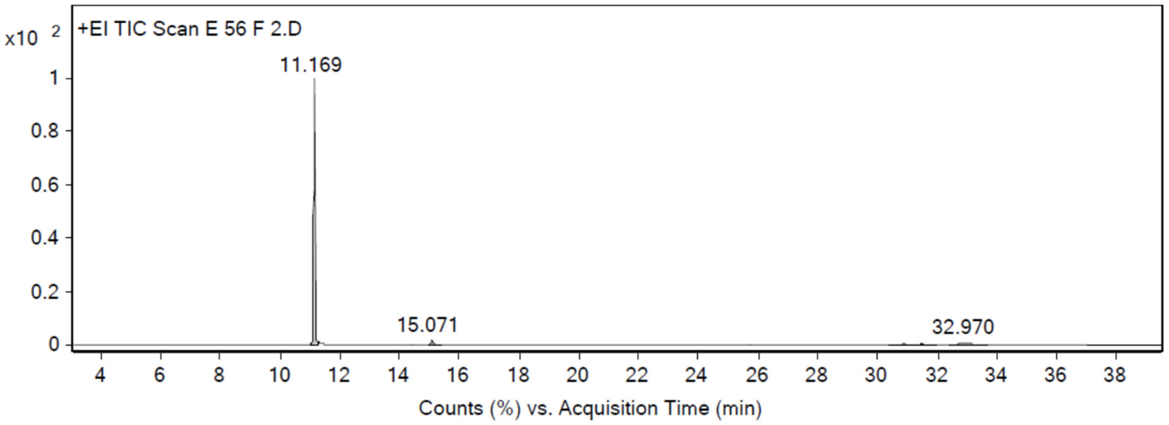

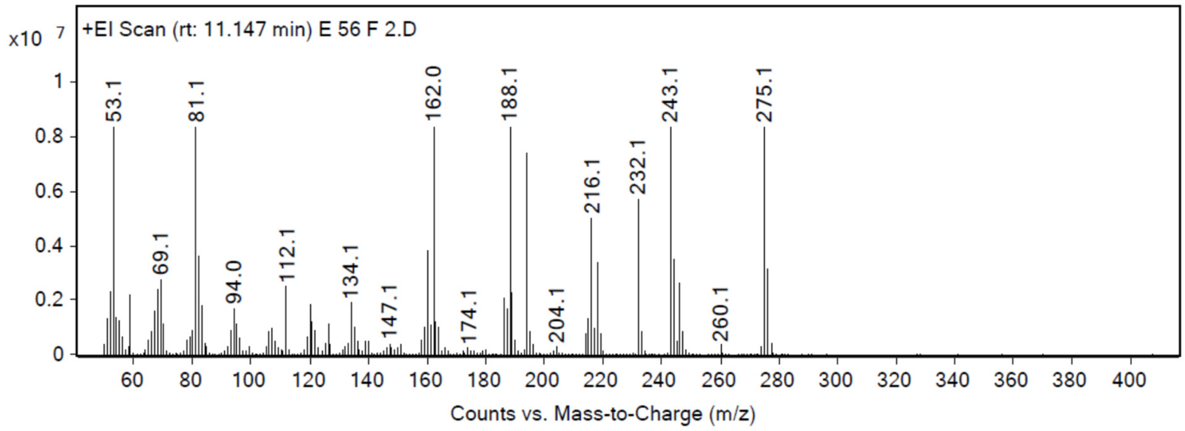

Figure S11. GC-MS analysis of **10c**.

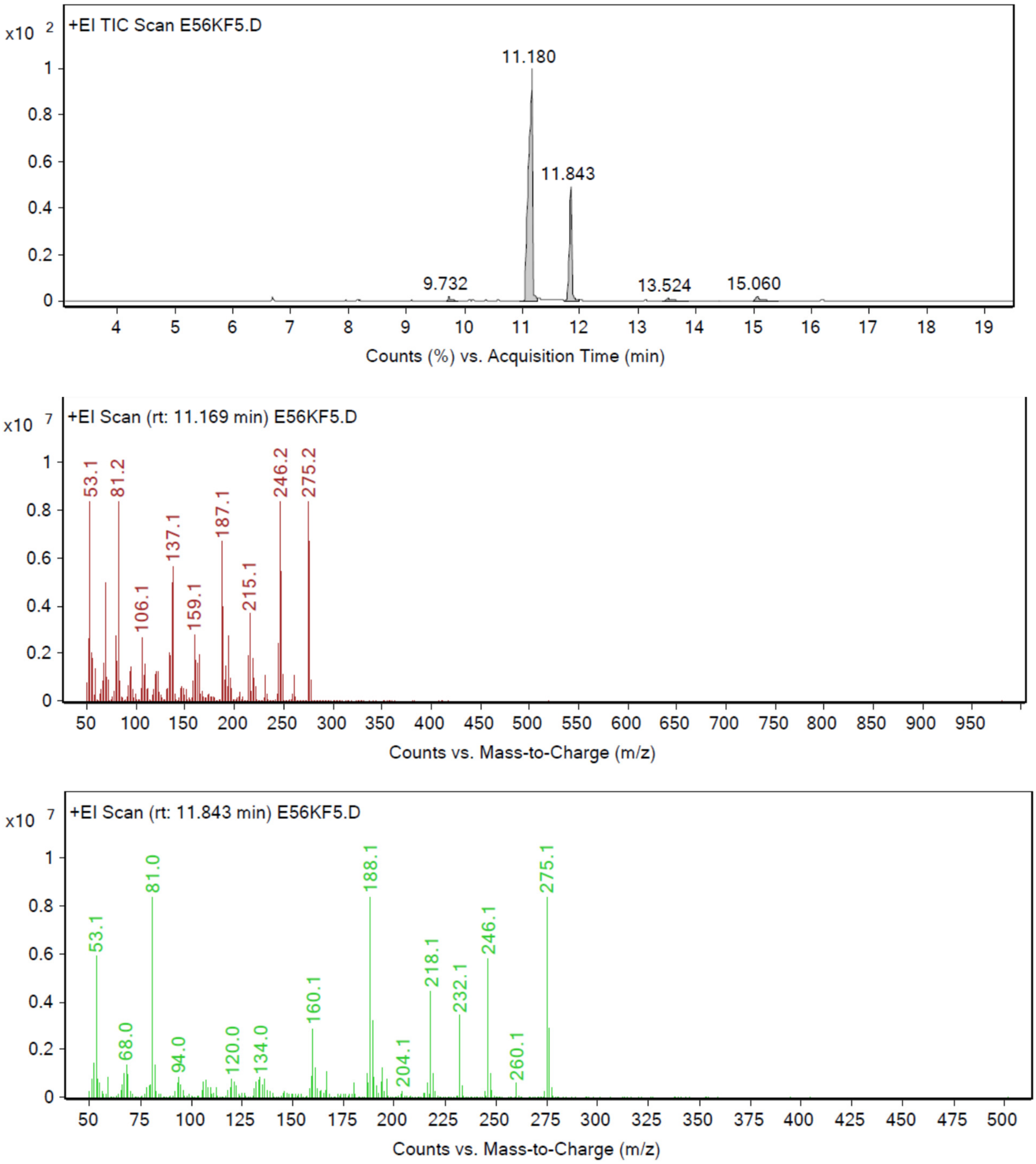

Figure S12. GC-MS analysis of the mixture containing **11** and **12**.

d) X-ray structures

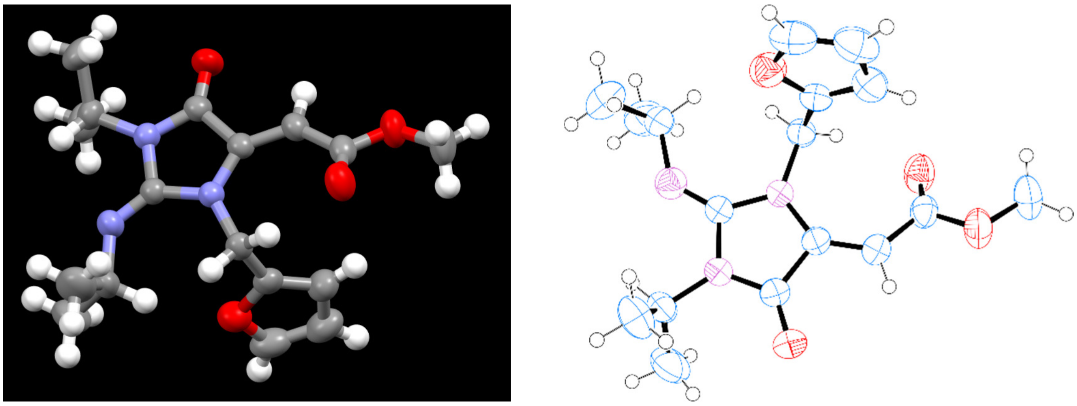

Figure S13a. Mercury (left) and ORTEP (right) presentation of *Z*-**9b**, showing thermal ellipsoids at 50% probability level.

Crystals of *Z*-**9b** suitable for single-crystal X-ray diffraction were obtained from a hexane solution at 4 °C. Crystal data for *Z*-**9b** ($C_{17}H_{23}N_3O_4$, M_r = 334.398): triclinic, space group P -1 (No 2), a = 9.1061(5) Å, b = 9.5071(5) Å, c = 11.4438(5) Å, α = 108.170(4) °, β = 102.471(5) °, γ = 98.706(5) °, V = 893.20 Å^3, Z = 2, T = 293(2) K, μ(CuKα) = 1.54184 mm^{-1}, 6141 reflections measured (5.30 ° $\leq \Theta \leq$ 74.79 °), 3519 unique (R_{int} = 0.0217, R_{sigma} 0.0161) were used in all calculations. The final R_1 was 0.0397 ($I > 2\sigma(I)$) and wR_2 was 0.1109 (all data).

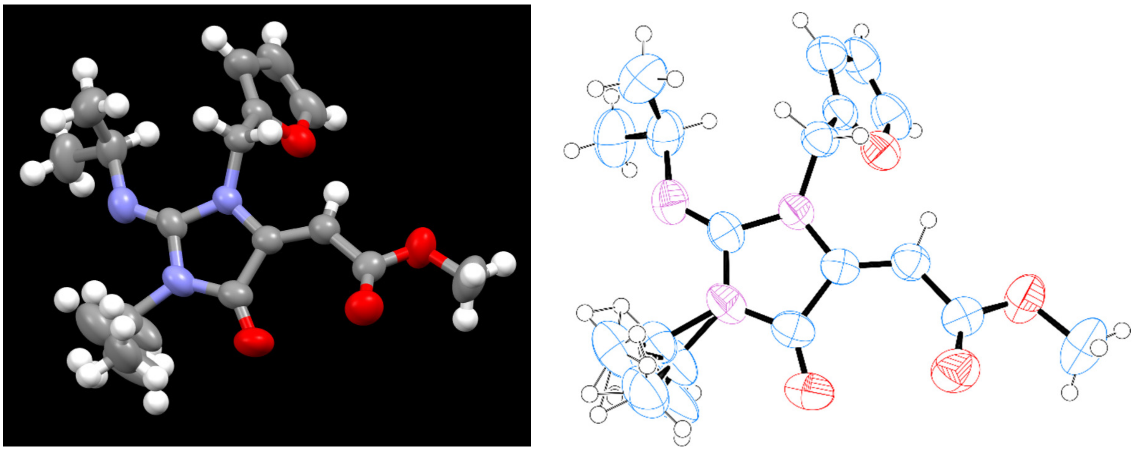

Figure S13b. Mercury (left) and ORTEP (right) presentation of *E*-**9b**, showing thermal ellipsoids at 50% probability level. A disorder of one isopropyl group is visible.

Crystals of *E*-**9b** suitable for single-crystal X-ray diffraction were obtained from a hexane solution at 4 °C. Crystal data for *E*-**9b** ($C_{17}H_{23}N_3O_4$, M_r = 334.398): triclinic, space group P -1 (No 2), a = 9.6156(4) Å, b = 9.7519(3) Å, c = 10.4067(2) Å, α = 104.761(2) °, β = 97.153(3) °, γ = 104.095(4) °, V = 896.934 Å^3, Z = 2, T = 303 (2) K, μ(CuKα) = 1.54184 mm^{-1}, 9980 reflections measured (4.483° $\leq \Theta \leq$ 76.976 °), 3568 unique (R_{int} =

0.0379, R_{sigma} 0.0433) were used in all calculations. The final R_1 was 0.0484 ($I > 2\sigma(I)$) and wR_2 was 0.1428 (all data).

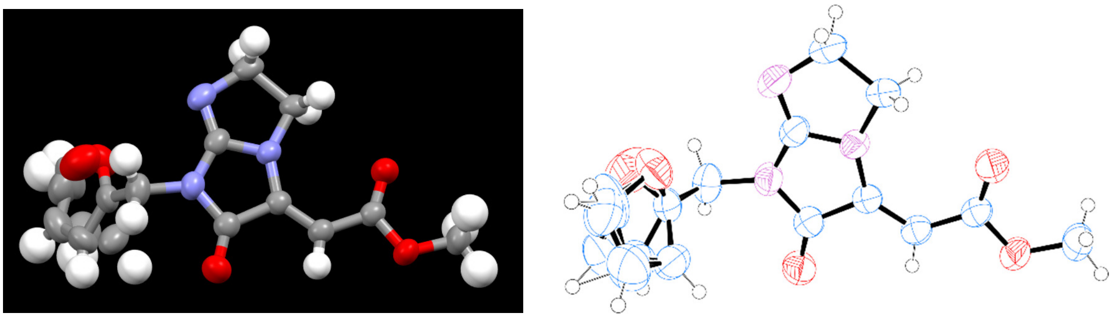

Figure S13c. Mercury (left) and ORTEP (right) presentation of **10c**, showing thermal ellipsoids at 50% probability level. A disorder of the furfuryl group is visible.

Crystals of **10c** suitable for single-crystal X-ray diffraction were obtained from a hexane solution at 4 °C. Crystal data for **10c** ($C_{13}H_{13}N_3O_4$, M_r = 272.28): monoclinic, space group P $2_1/n$ (No 14), a = 12.0617(2) Å, b = 5.09640(10) Å, c = 21.2023(3) Å, α = 90.0 °, β = 95.3400(10) °, γ = 90.0 °, V = 1297.68 Å^3, Z = 4, T = 297(2) K, μ(CuKα) = 1.54184 mm^{-1}, 9880 reflections measured (4.062° ≤ Θ ≤ 79.368 °), 2775 unique (R_{int} = 0.0321, R_{sigma} 0.0324) were used in all calculations. The final R_1 was 0.0576 ($I > 2\sigma(I)$) and wR_2 was 0.1503 (all data).

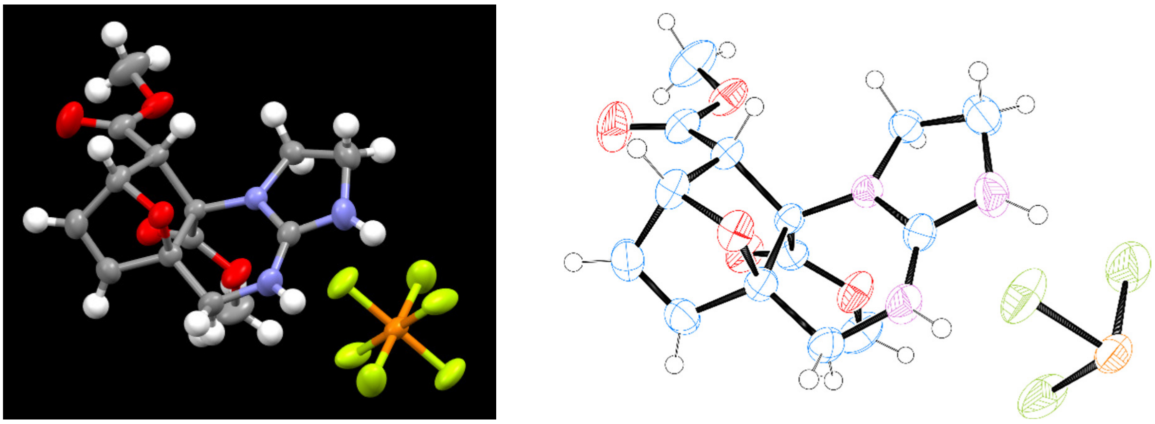

Figure S14a. Mercury (left) and ORTEP (right) presentation of **5c**·HPF_6, showing thermal ellipsoids at 50% probability level.

Crystals of **5c**·HPF_6 suitable for single-crystal X-ray diffraction were obtained from a methanol solution at 4 °C. Crystal data for **5c**·HPF_6 ($C_{14}H_{18}F_6N_3O_5P$, M_r = 453.27): triclinic, space group P -1 (No. 2), a = 9.1785(3) Å, b = 9.2352(3) Å, c = 10.5619(4) Å, α = 93.481(3) °, β = 109.408(3) °, γ = 102.548(3) °, V = 815.61(5) $Å^3$, Z = 2, T = 293.72(10) K, μ(CuKα) = 1.54184 mm^{-1}, 8880 reflections measured (4.463 ° ≤ Θ ≤ 76.318 °), 3252 unique (R_{int} = 0.0238, R_{sigma} 0.0256) were used in all calculations. The final R_1 was 0.0401 (I > 2σ(I)) and wR_2 was 0.1183 (all data).

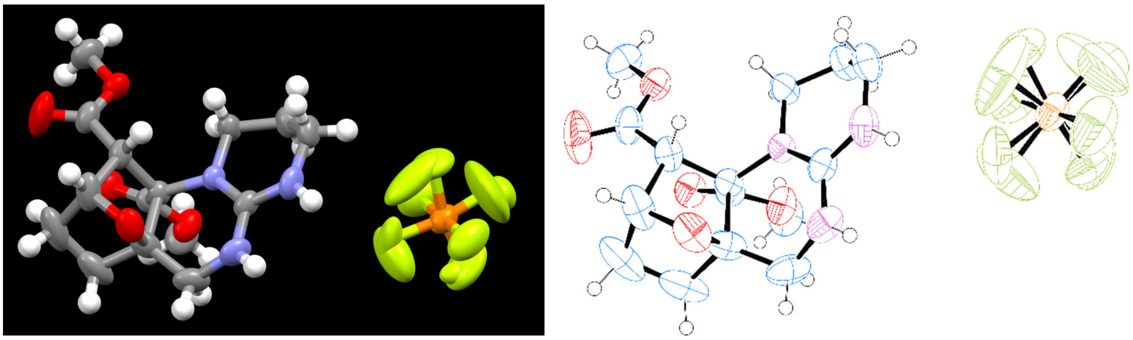

Figure S14b. Mercury (left) and ORTEP (right) presentation of **7d**·HPF_6, showing thermal ellipsoids at 50% probability level. A disorder of hexafluorophosphate anion is visible.

Crystals of **7d**·HPF_6 suitable for single-crystal X-ray diffraction were obtained from an ethyl acetate solution at 4 °C. Crystal data for **7d**·HPF_6 ($C_{15}H_{22}F_6N_3O_5P$, M_r = 469.32): monoclinic, space group P 12_1/n 1 (No 14), a = 7.92120(10) Å, b = 15.9628(2) Å, c = 15.9628(2) Å, α = 90.0 ° β = 94.2430(10) °, γ = 90.0 °, V = 1946.21(4) $Å^3$, Z = 4, T = 296(2) K, μ(CuKα) = 1.54184 mm^{-1}, 14344 reflections measured (3.990 ° ≤ Θ ≤ 79.502 °), 3981 unique (R_{int} = 0.0202, R_{sigma} 0.0234) were used in all calculations. The final R_1 was 0.0559 (I > 2σ(I)) and wR_2 was 0.1580 (all data).

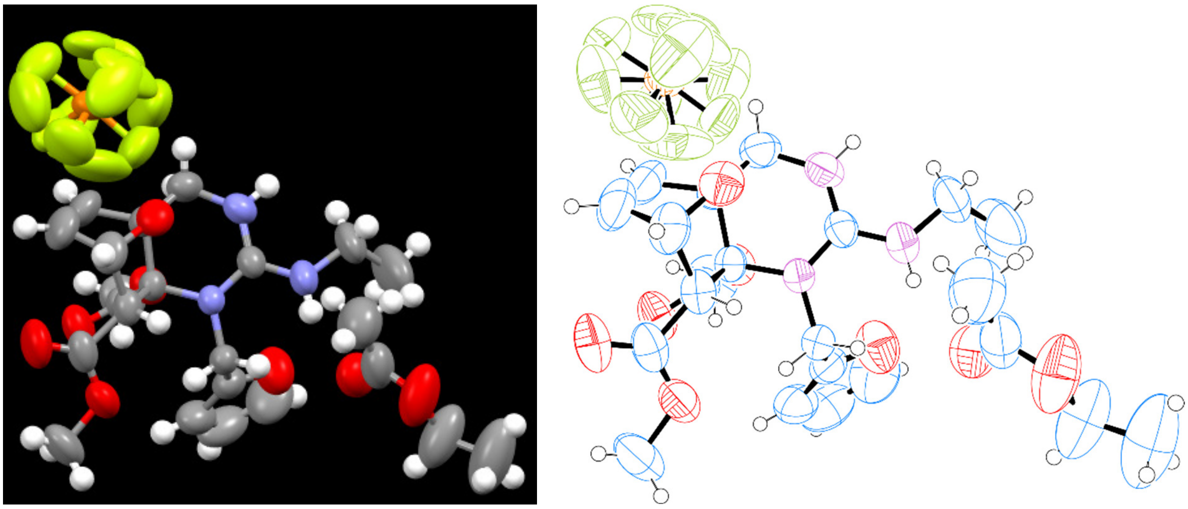

Figure S14c. Mercury (left) and ORTEP (right) presentation of **5f**·HPF_6, showing thermal ellipsoids at 50% probability level. A disorder of hexafluorophosphate anion is visible.

Crystals of **5f**·HPF_6 suitable for single-crystal X-ray diffraction were obtained by slow diffusion of ether vapors to an ethyl acetate solution at room temperature °C. Crystal data for **5f**·HPF_6 ($C_{23}H_{32}F_6N_3O_8P$, M_r = 620.48): monoclinic, space group P 12_1/n 1 (No 14), a = 15.1081(2) Å, b = 11.0670(2) Å, c = 17.9998(2) Å, α = 90.0 ° β = 102.9010(10) °, γ = 90.0 °, V = 2933.62(7) Å^3, Z = 4, T = 297(2) K, μ(CuKα) = 1.54184 mm^{-1}, 58858 reflections measured (3.461 ° ≤ Θ ≤ 79.782 °), 6308 unique (R_{int} = 0.0195, R_{sigma} 0.0376) were used in all calculations. The final R_1 was 0.0776 (I > 2σ(I)) and wR_2 was 0.2433 (all data).

S3. NMR and IR spectra

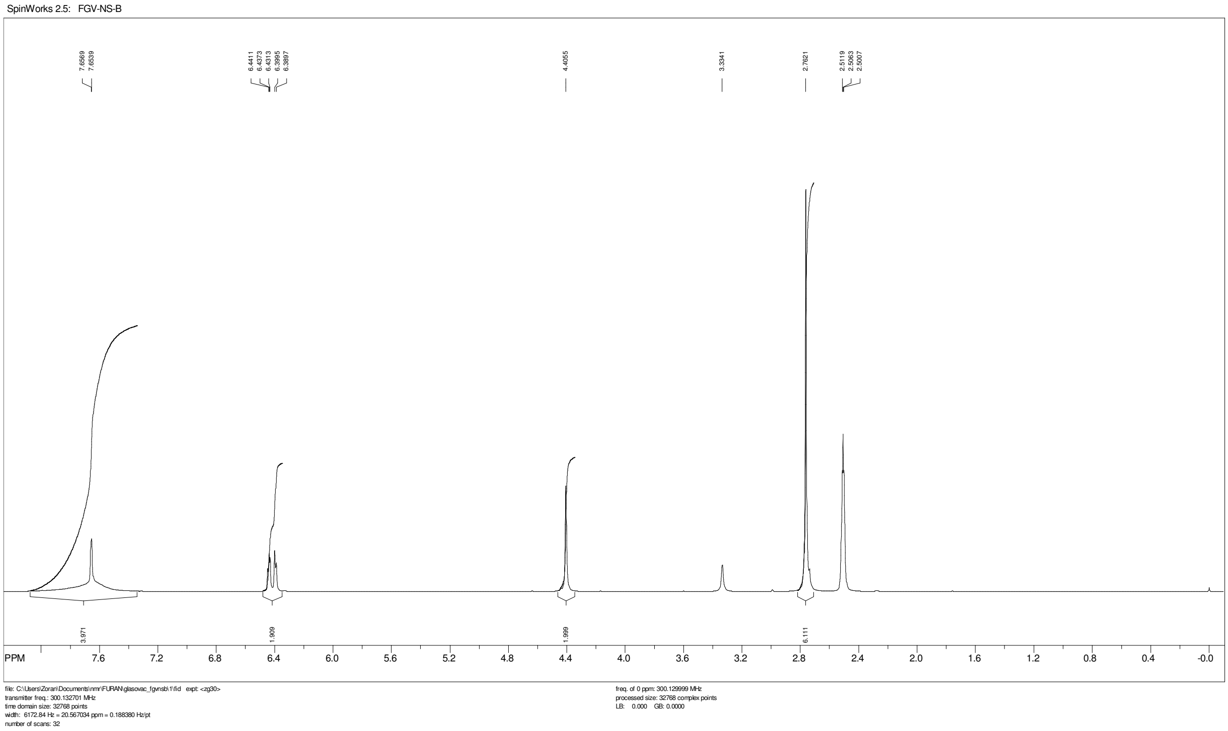

^{1}H NMR (DMSO-d_6, 300 MHz) of the **3a**·HI

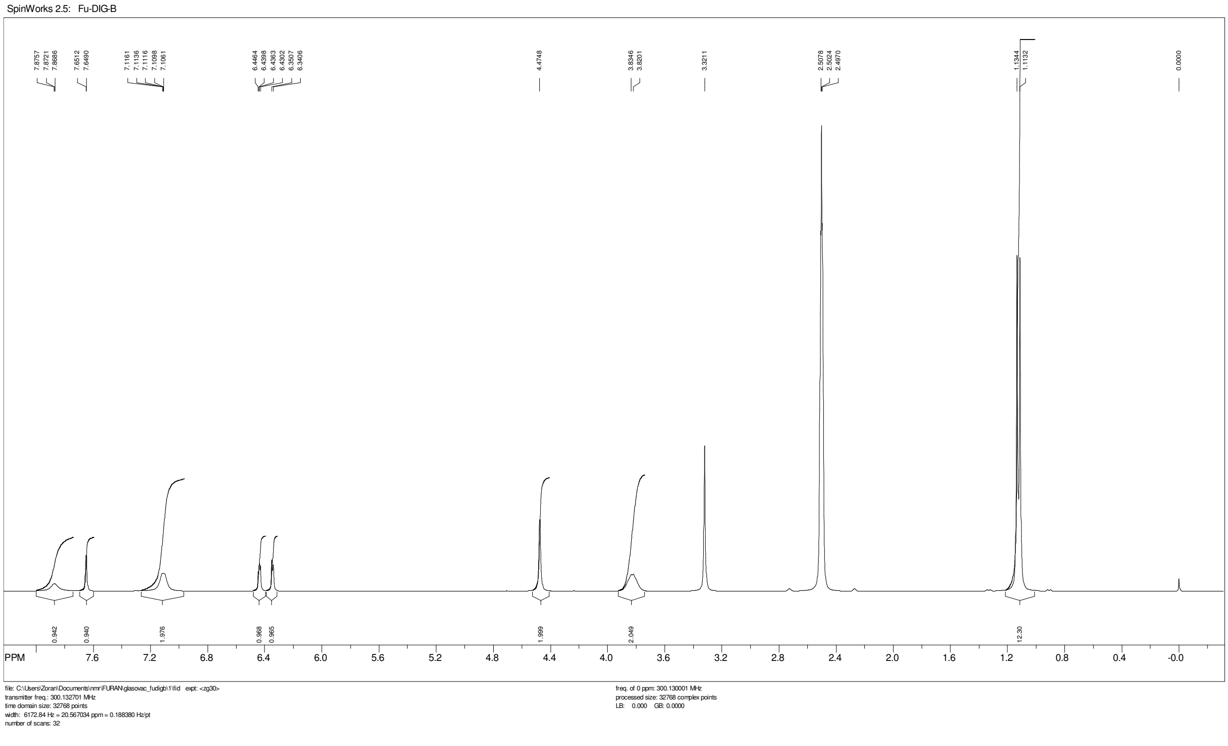

^{1}H NMR (DMSO-d_6, 300 MHz) of the **3b**·HI.

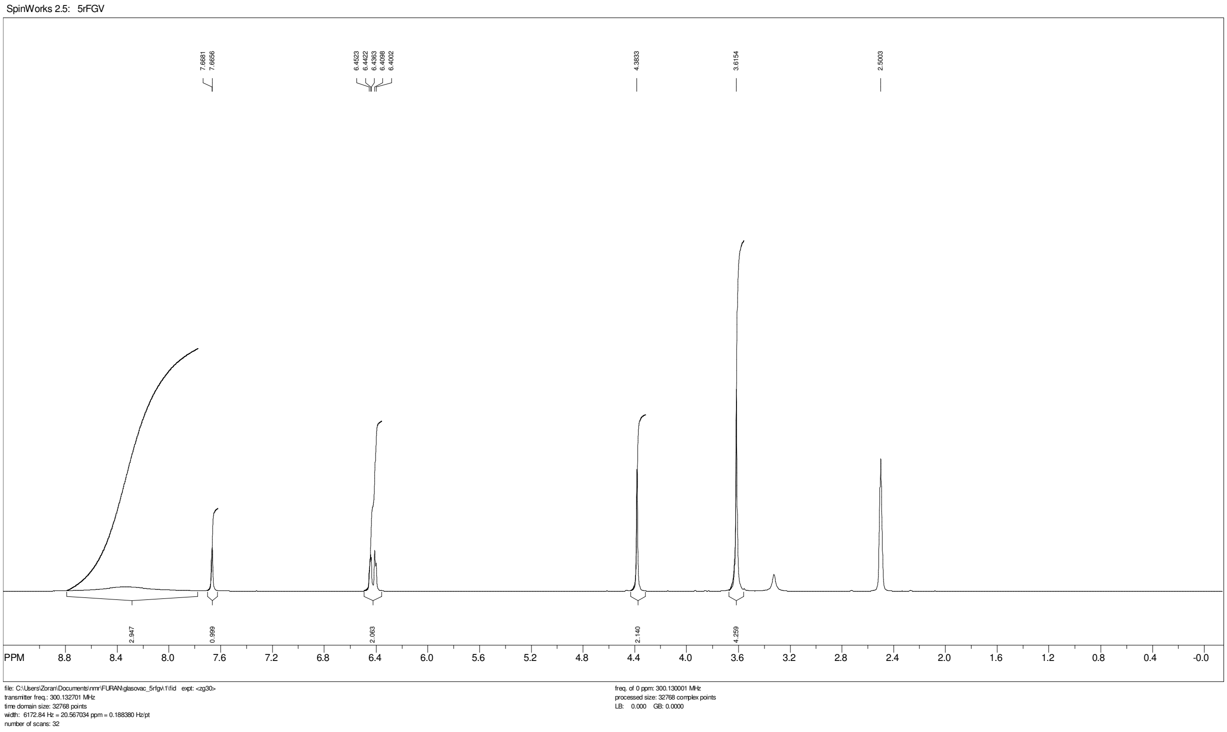

^{1}H NMR (DMSO-d_6, 300 MHz) of the **3c**·HI.

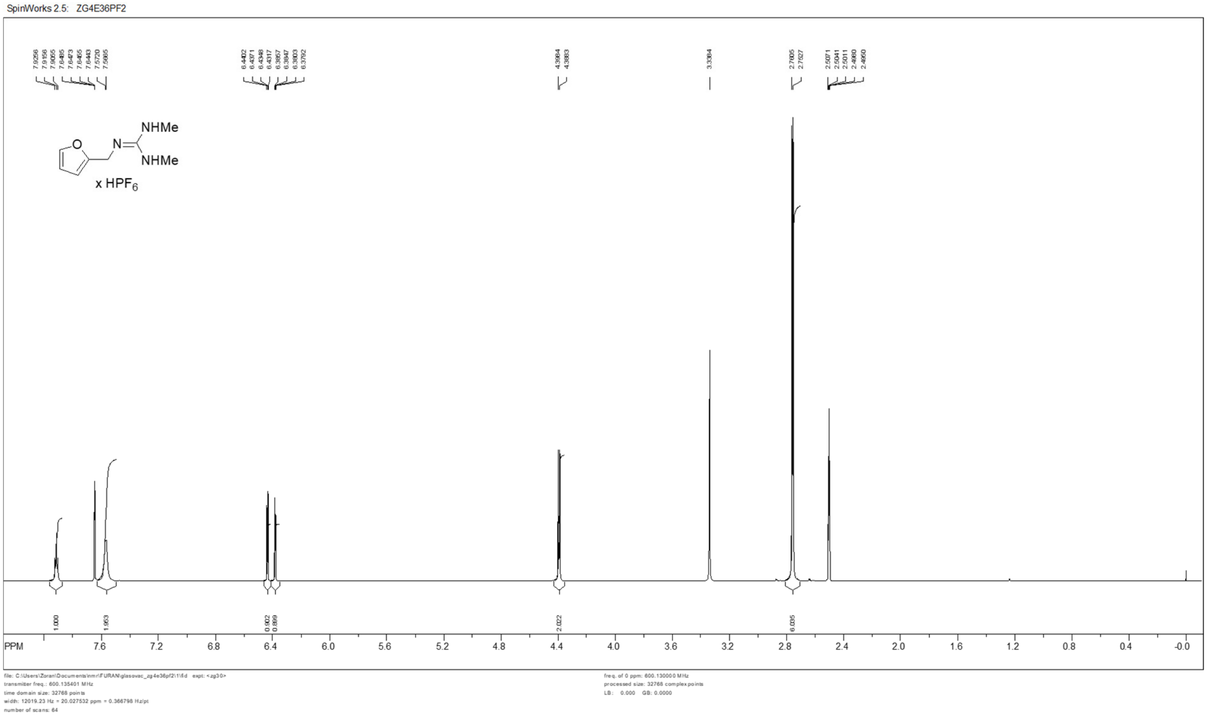

^{1}H NMR (DMSO-d_6, 300 MHz) of the **3a**·HPF_6

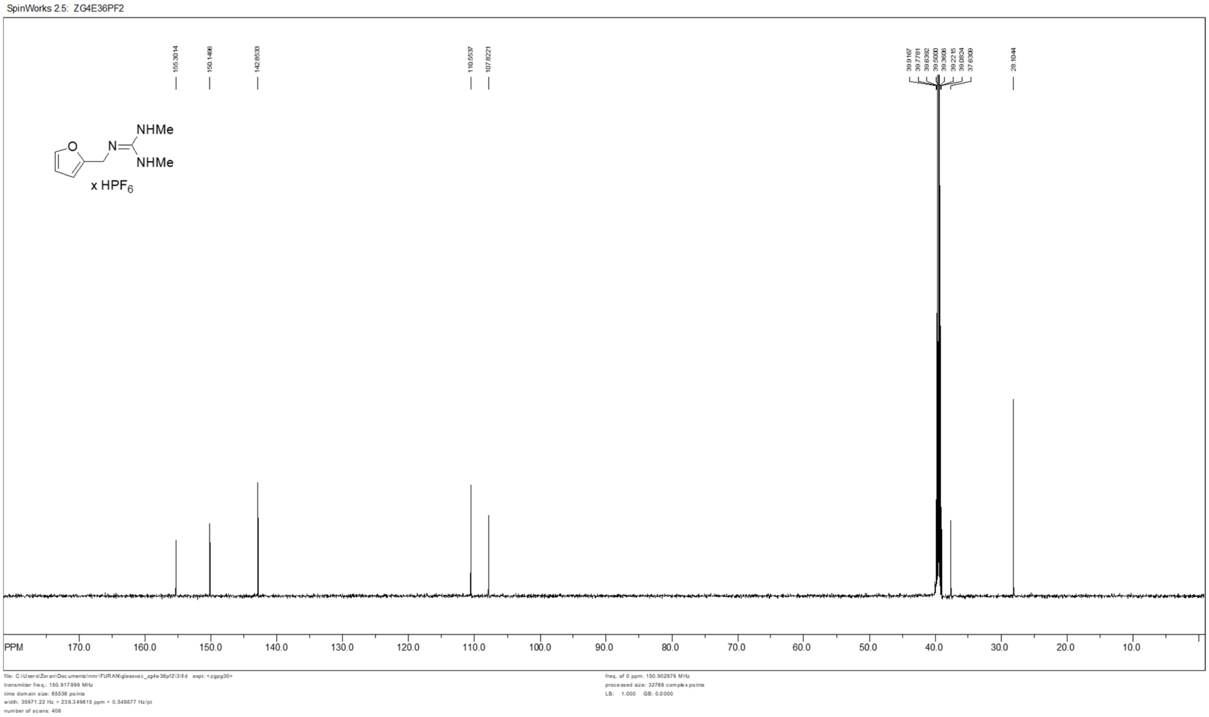

^{13}C NMR (DMSO-d_6, 150.9 MHz) of the **3a**·HPF_6.

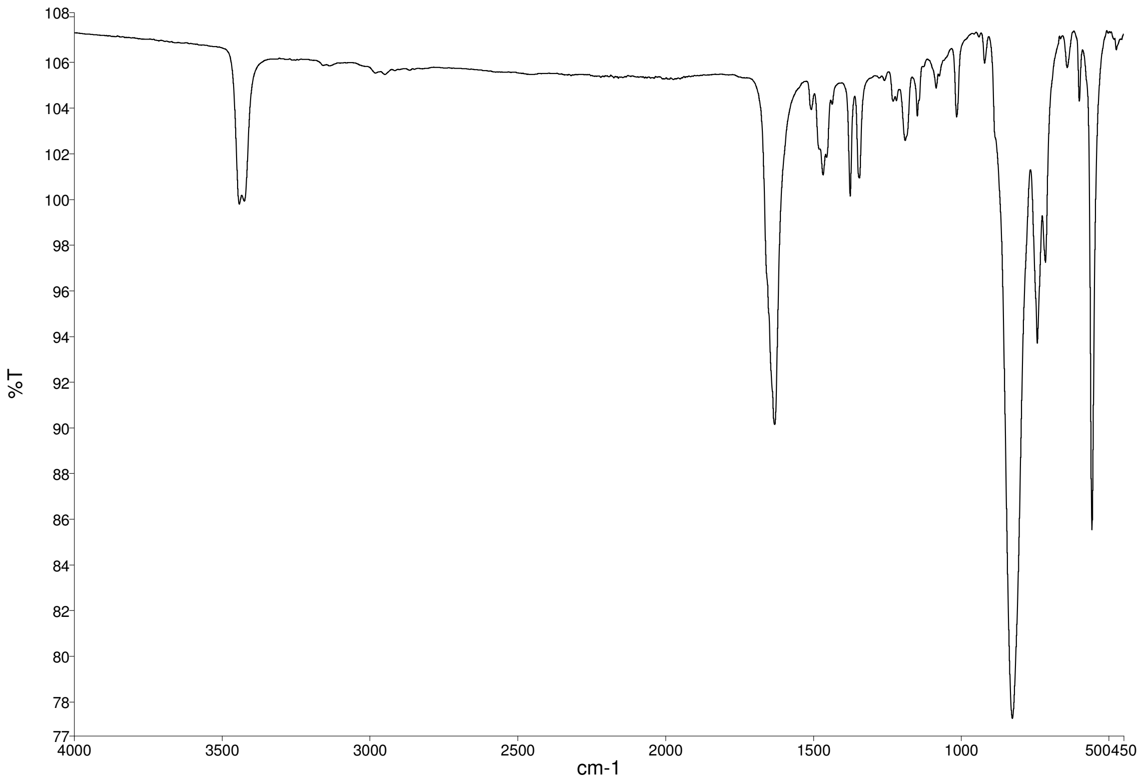

FTIR-ATR spectrum of **3a**·HPF_6.

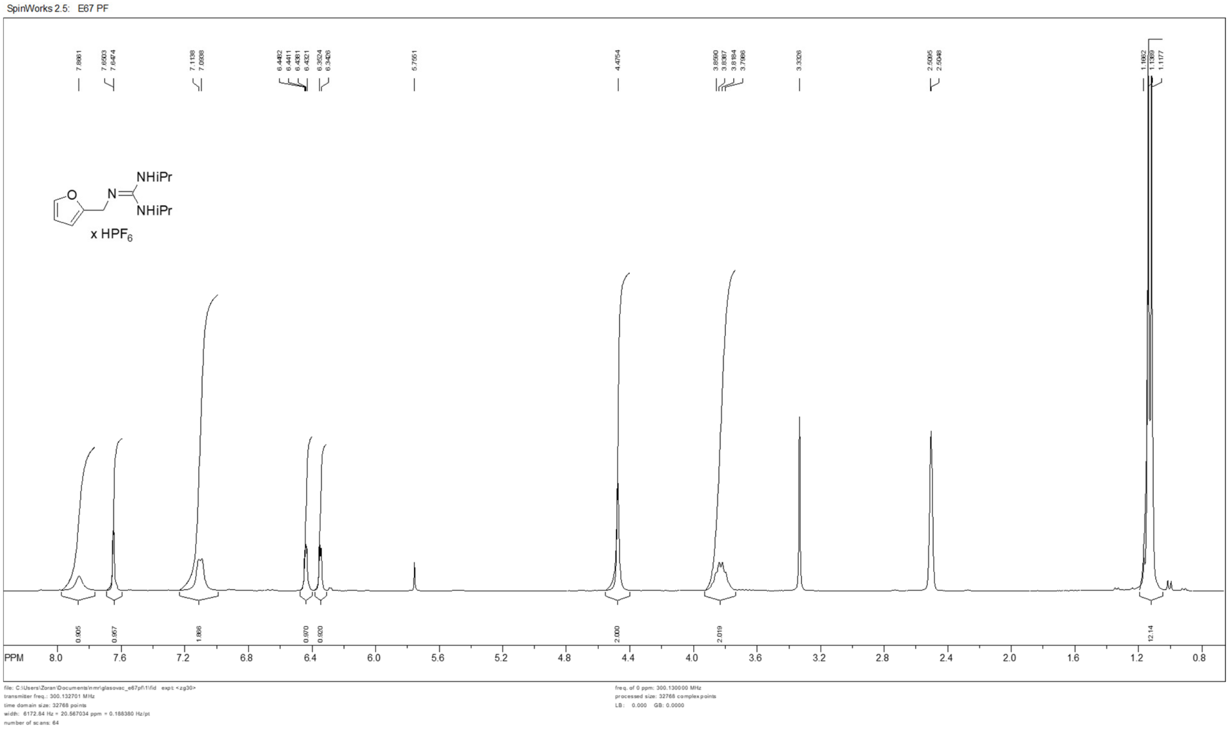

^{1}H NMR (DMSO-d_6, 300 MHz) of the **3b**·HPF_6.

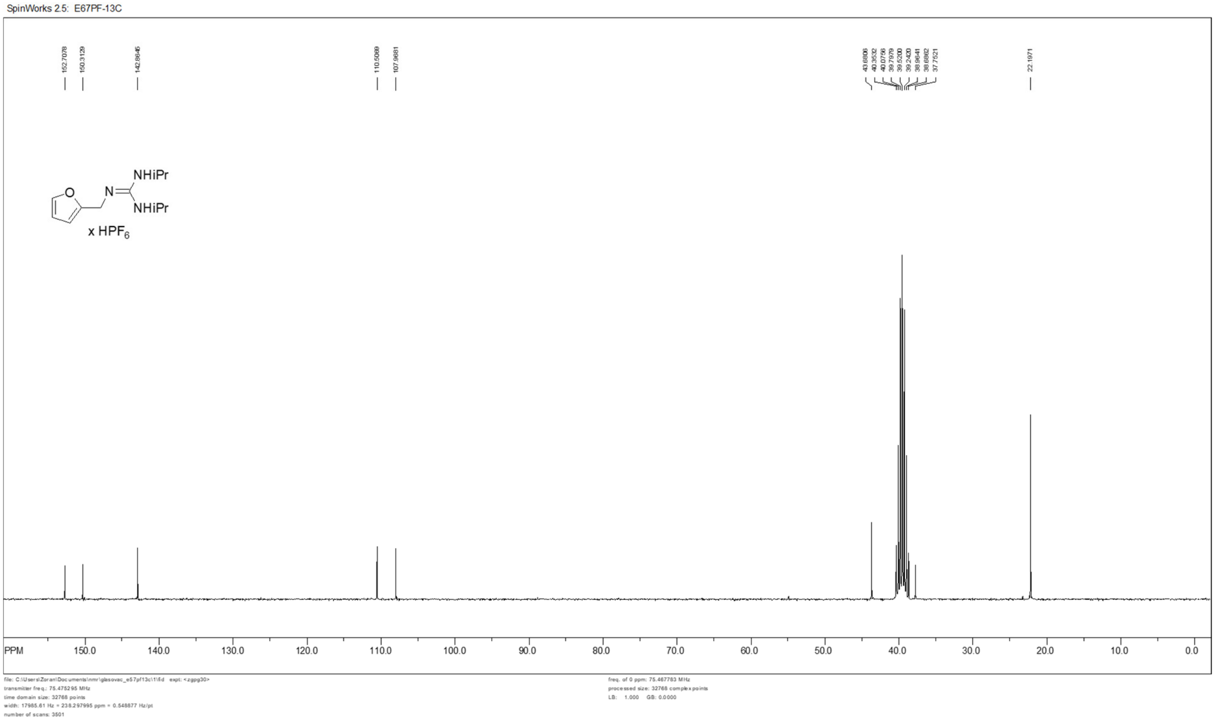

^{13}C NMR (DMSO-d_6, 75.5 MHz) of the **3b**·HPF_6.

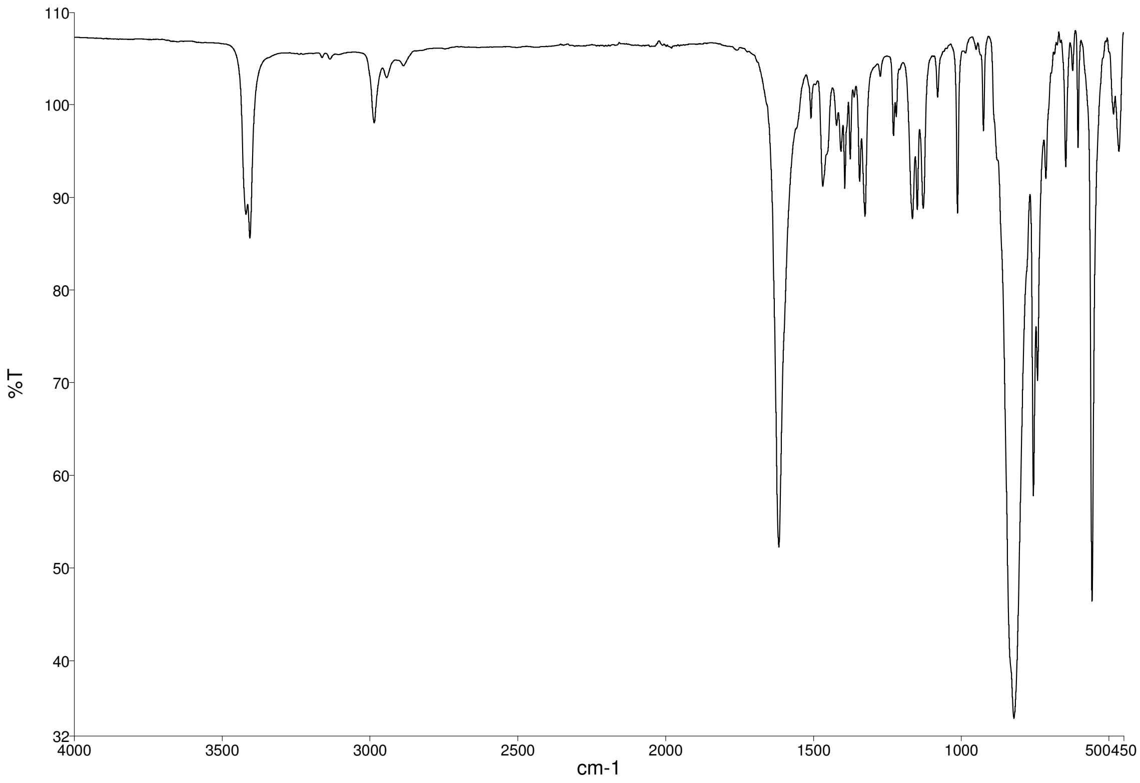

FTIR-ATR spectrum of **3b**·HPF_6.

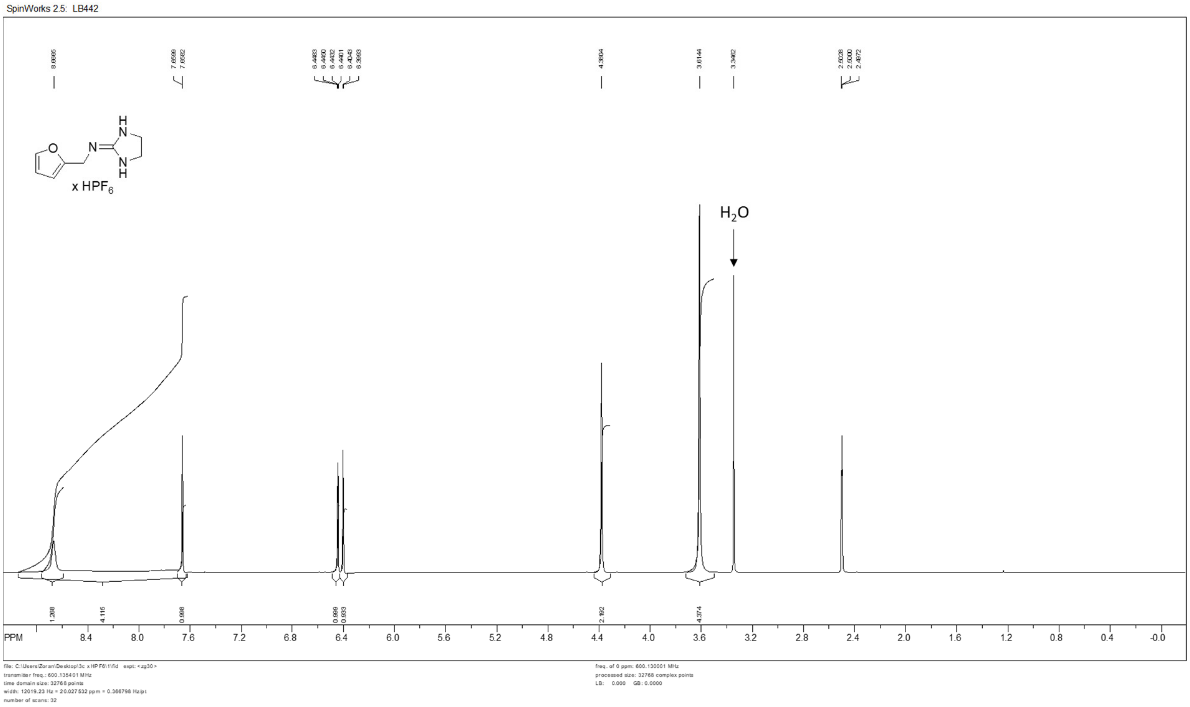

^{1}H NMR (DMSO-d_6, 600 MHz) of the **3c**·HPF_6.

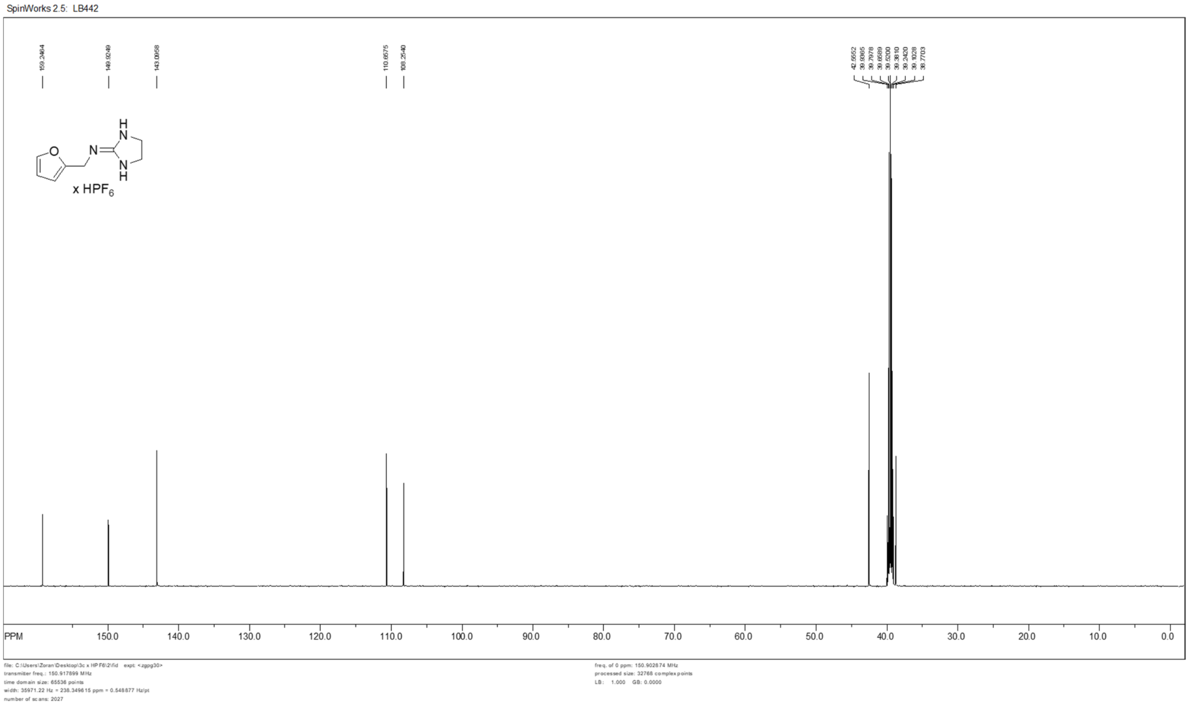

^{13}C NMR (DMSO-d_6, 150.9 MHz) of the **3c**·HPF_6.

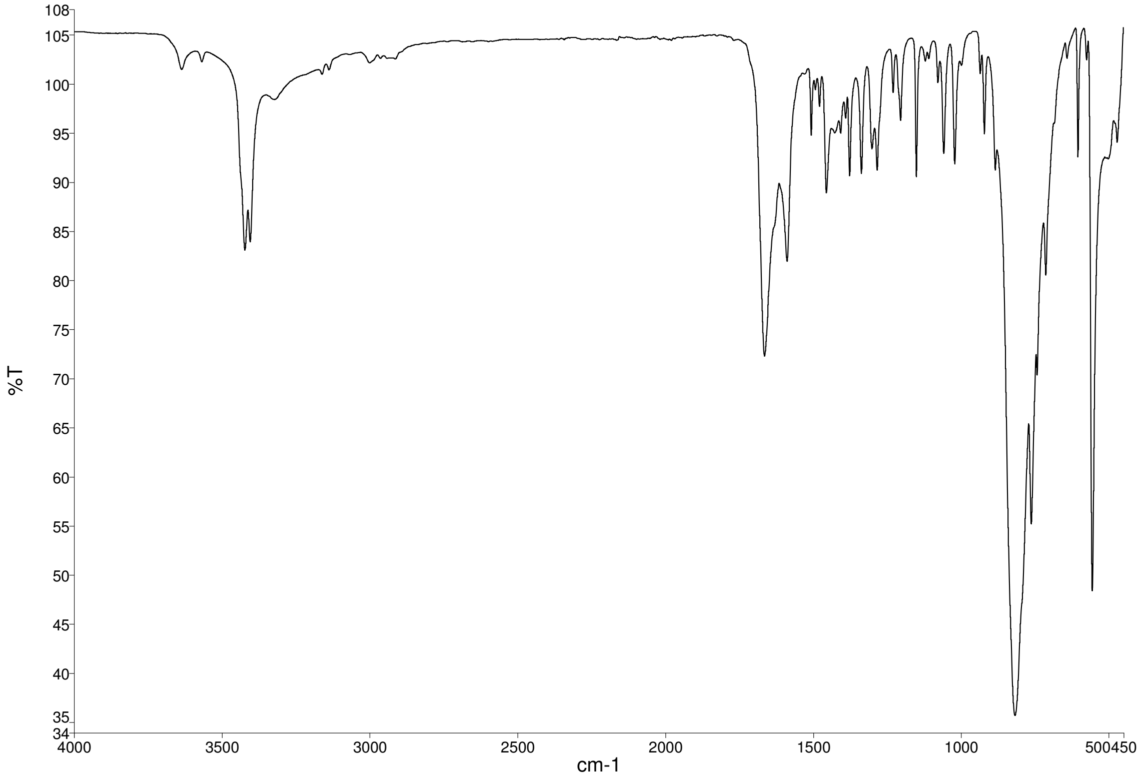

FTIR-ATR spectrum of **3c**·HPF_6.

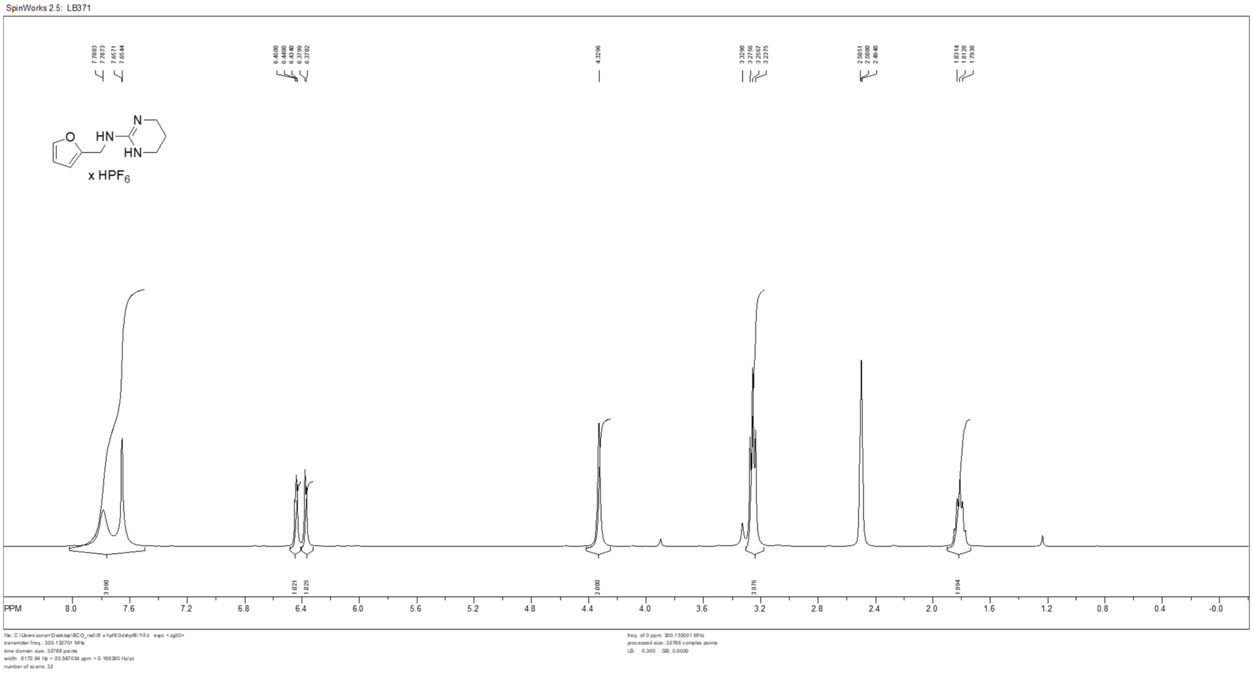

^{1}H NMR (DMSO-d_6, 300 MHz) of the **3d**·HPF_6.

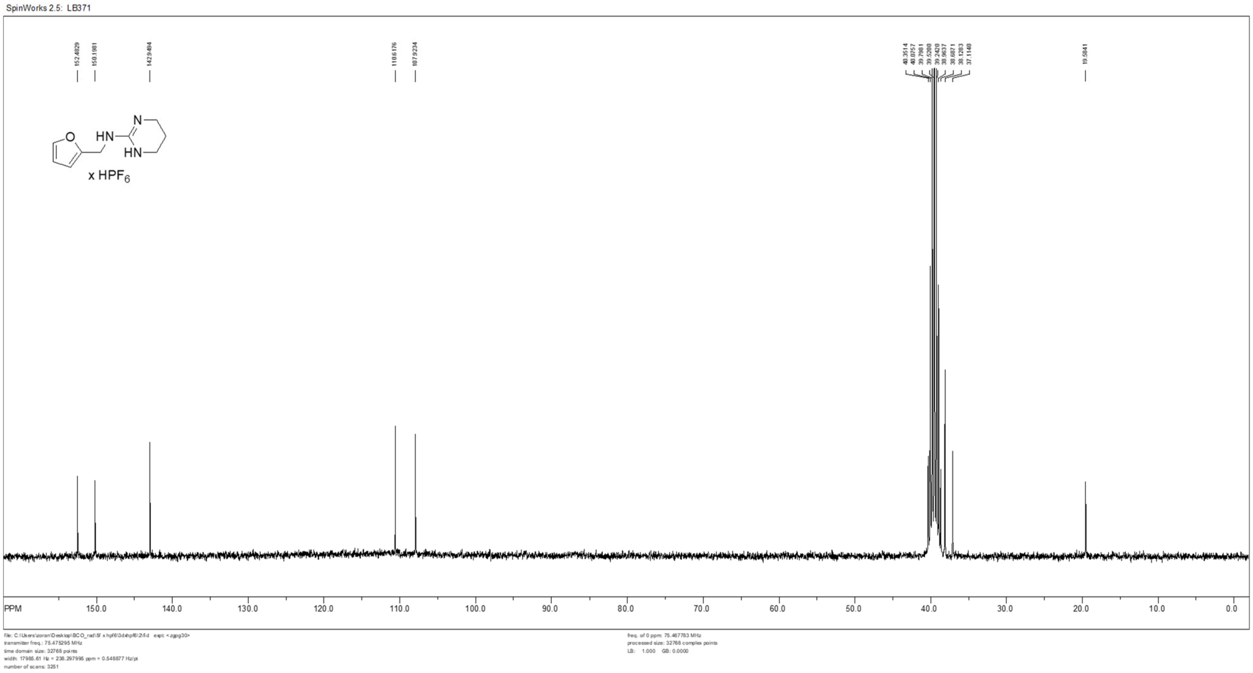

^{13}C NMR (DMSO-d_6, 75.5 MHz) of the **3d**·HPF_6.

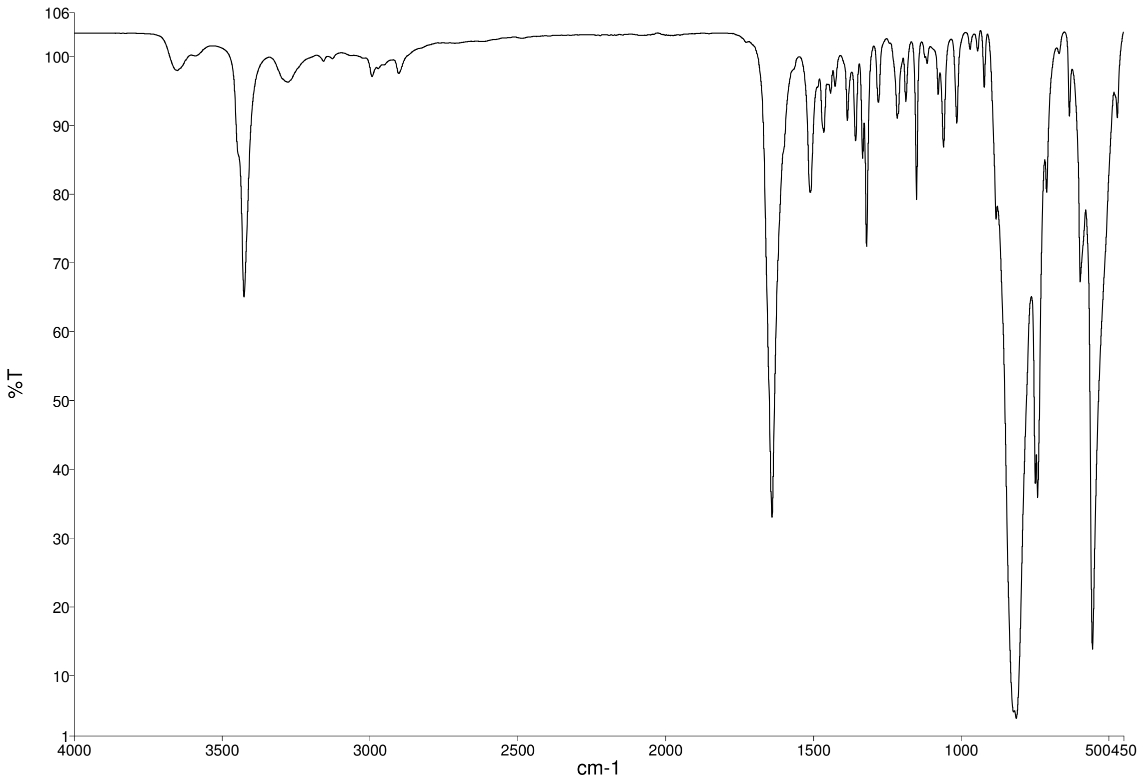

FTIR-ATR spectrum of **3d**·HPF_6.

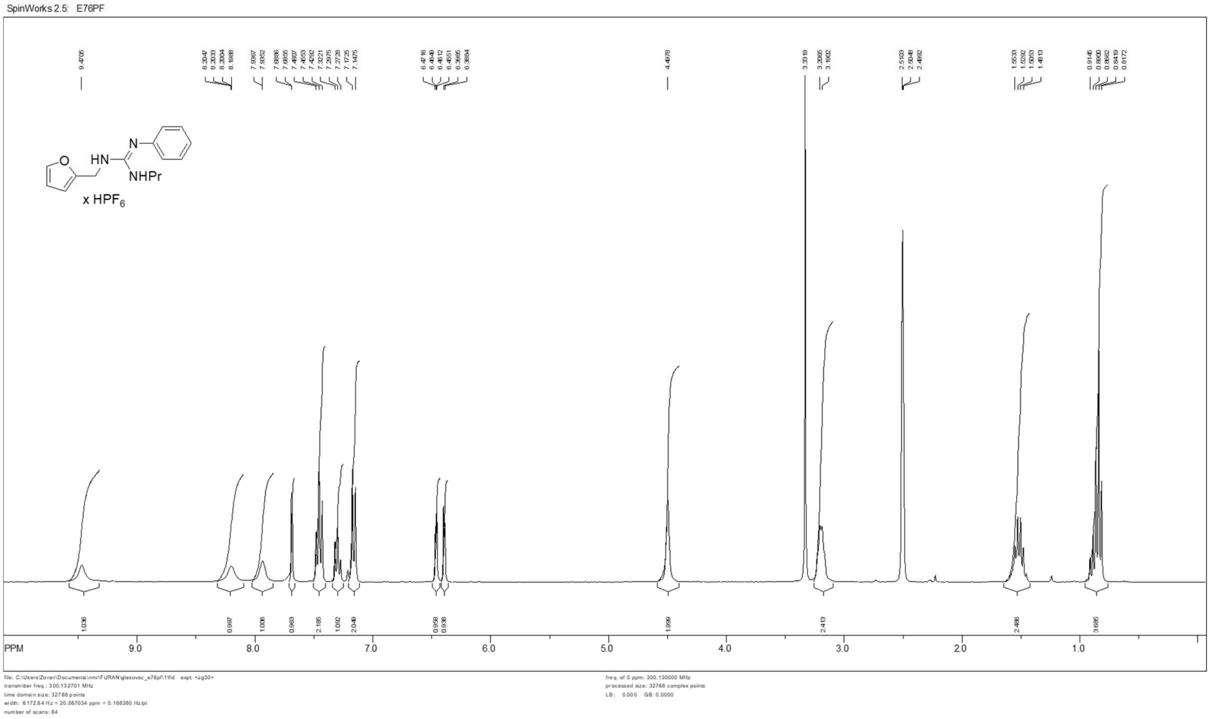

^1H NMR (DMSO-d_6, 300 MHz) of the **3e**·HPF_6.

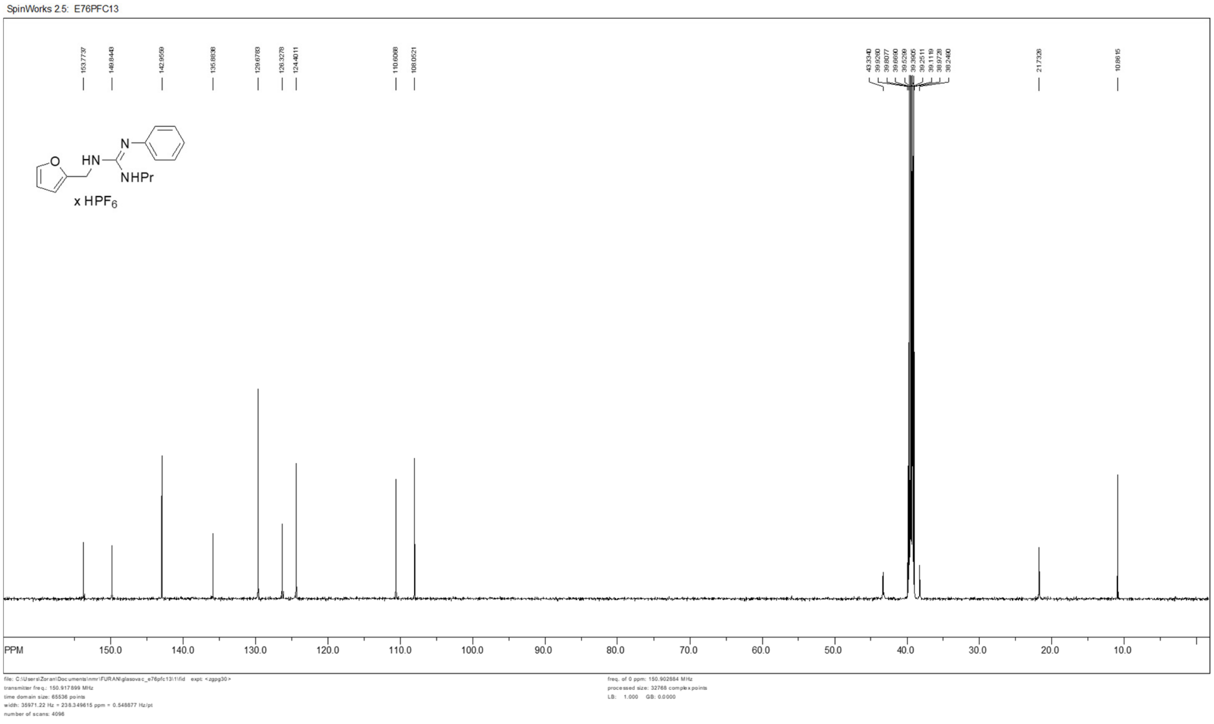

^{13}C NMR (DMSO-d_6, 150.9 MHz) of the **3e**·HPF_6.

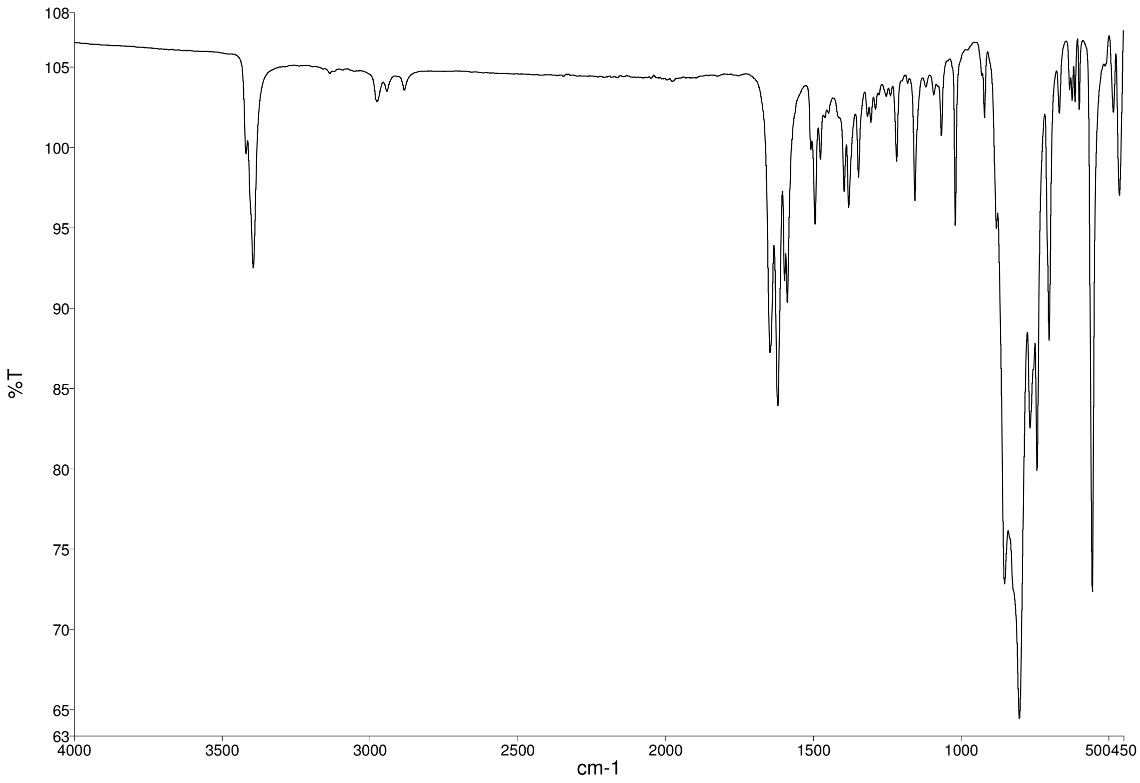

FTIR-ATR spectrum of **3e**·HPF_6.

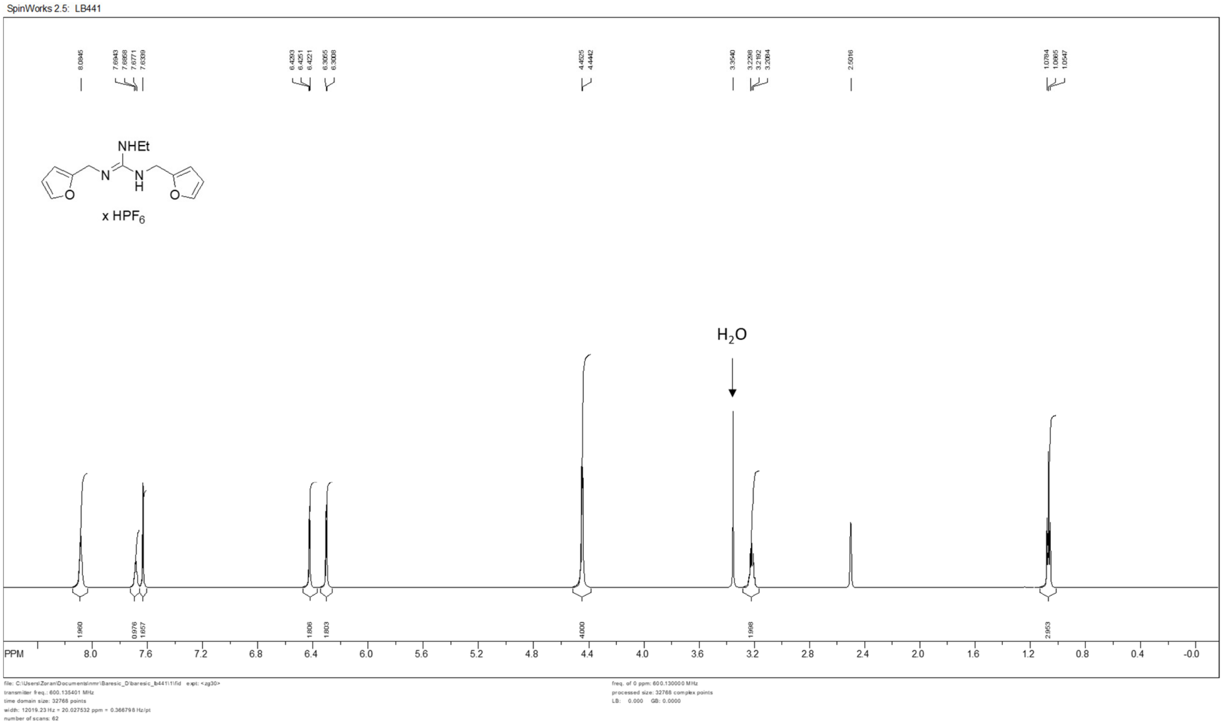

^{1}H NMR spectrum (DMSO-d$_6$, 600 MHz) of the **3f**·HPF$_6$.

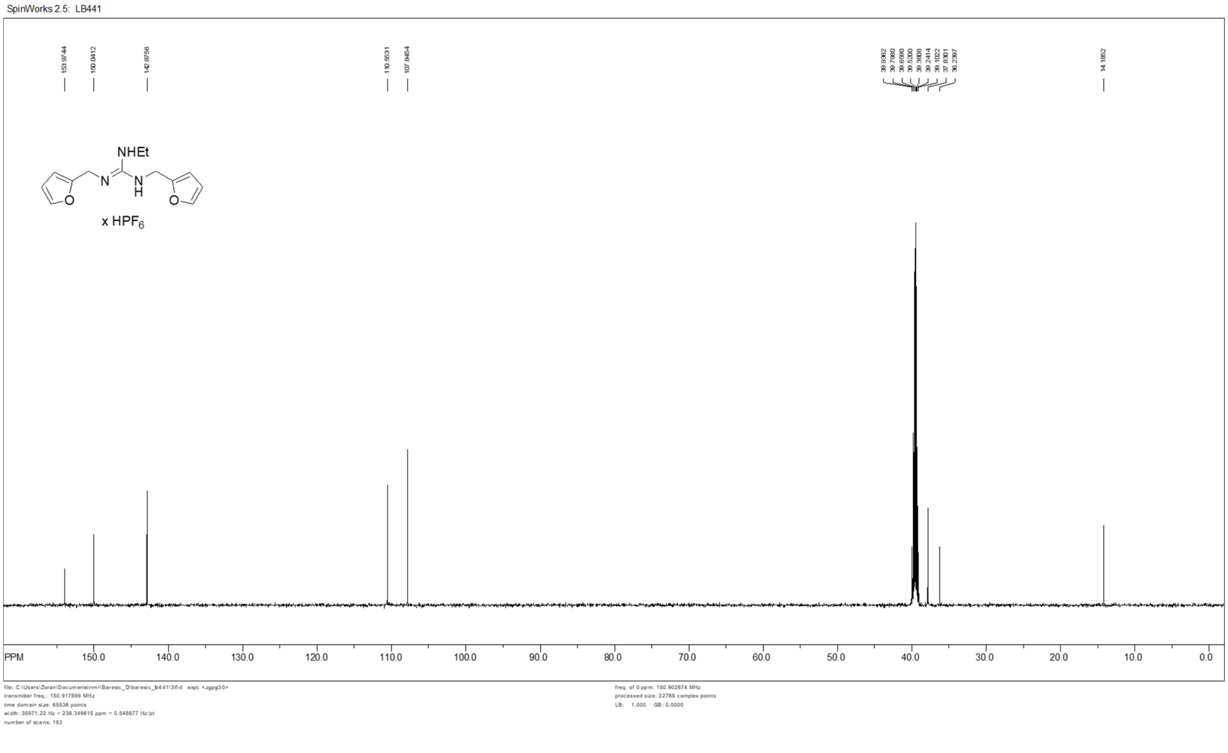

^{13}C NMR (DMSO-d$_6$, 150.9 MHz) of the **3f**·HPF$_6$.

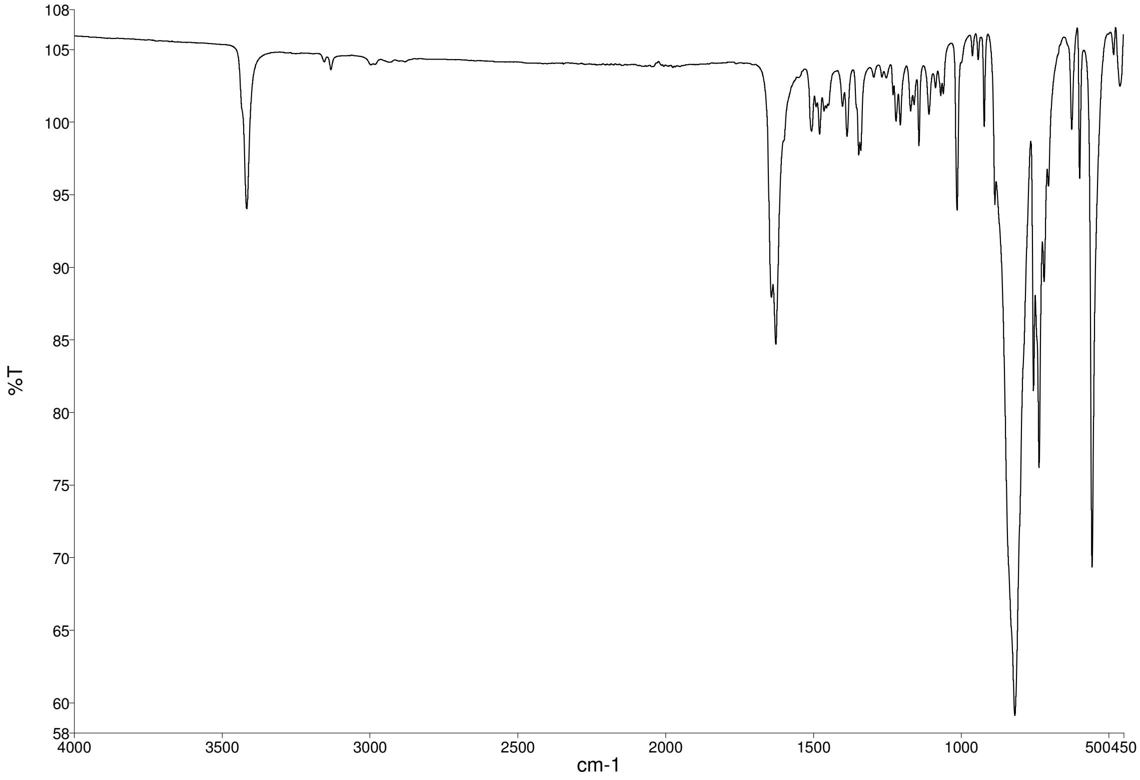

FTIR-ATR spectrum of **3f**·HPF_6.

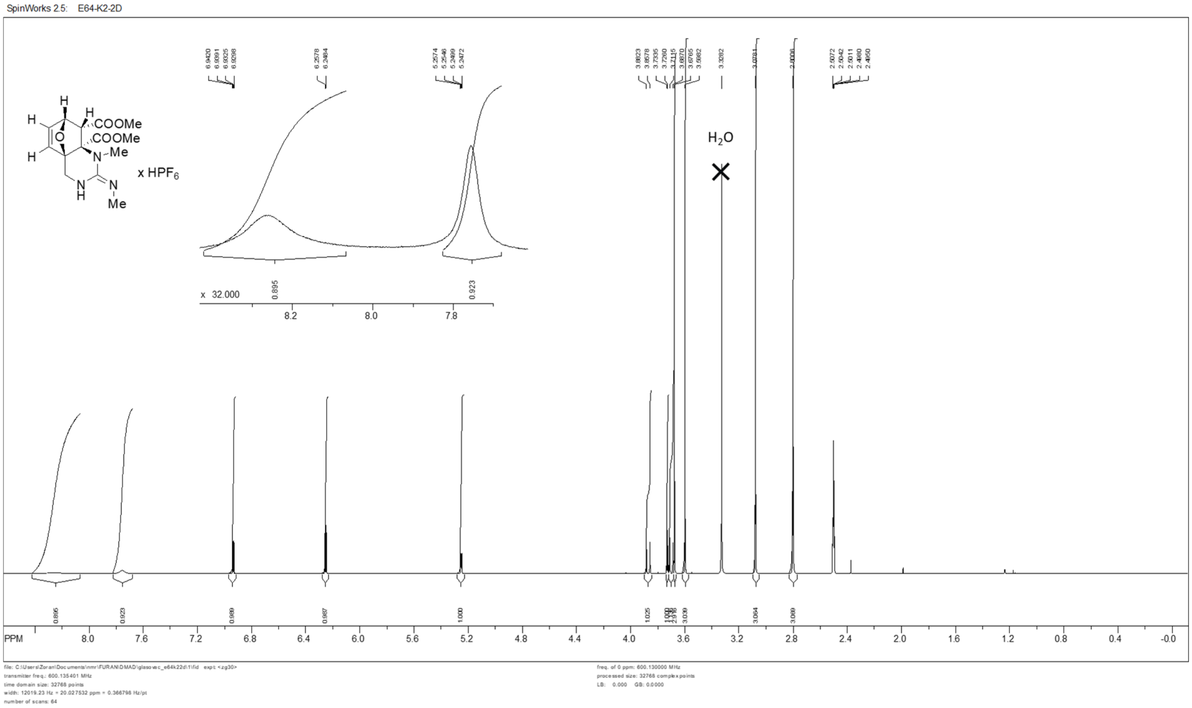

^{1}H NMR spectrum (DMSO-d_6, 600 MHz) of the **5a**·HPF_6.

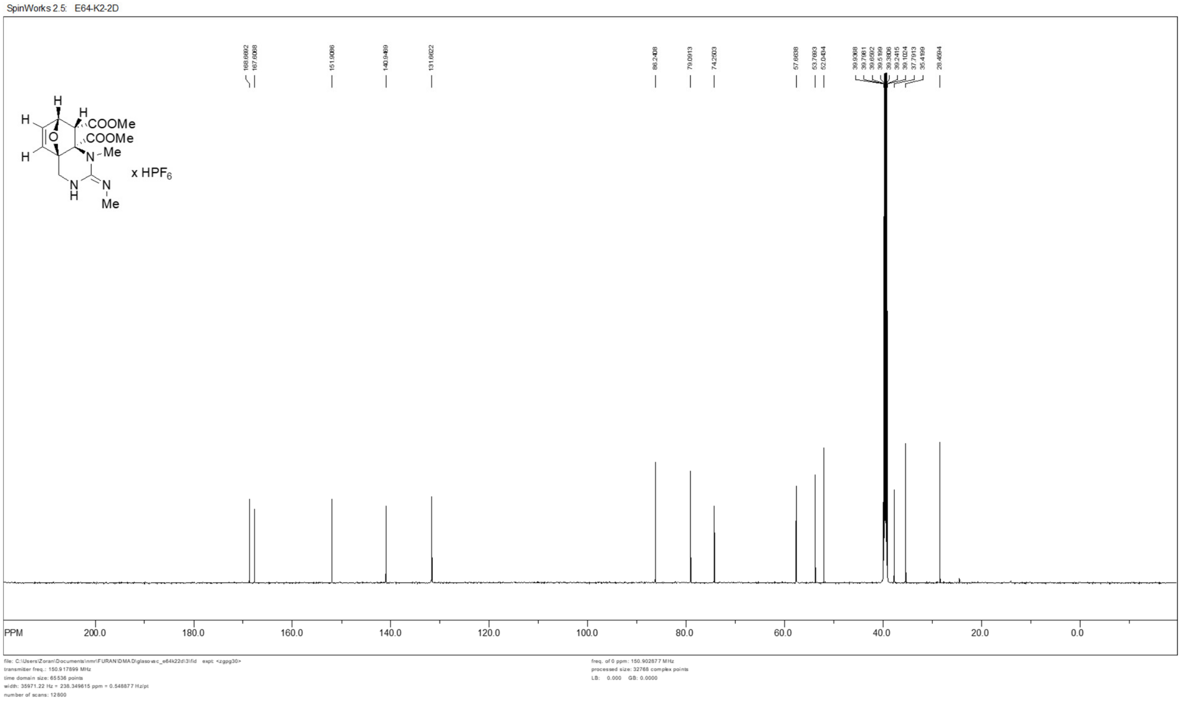

^{13}C NMR spectrum (DMSO-d_6, 150.9 MHz) of the **5a**·HPF_6.

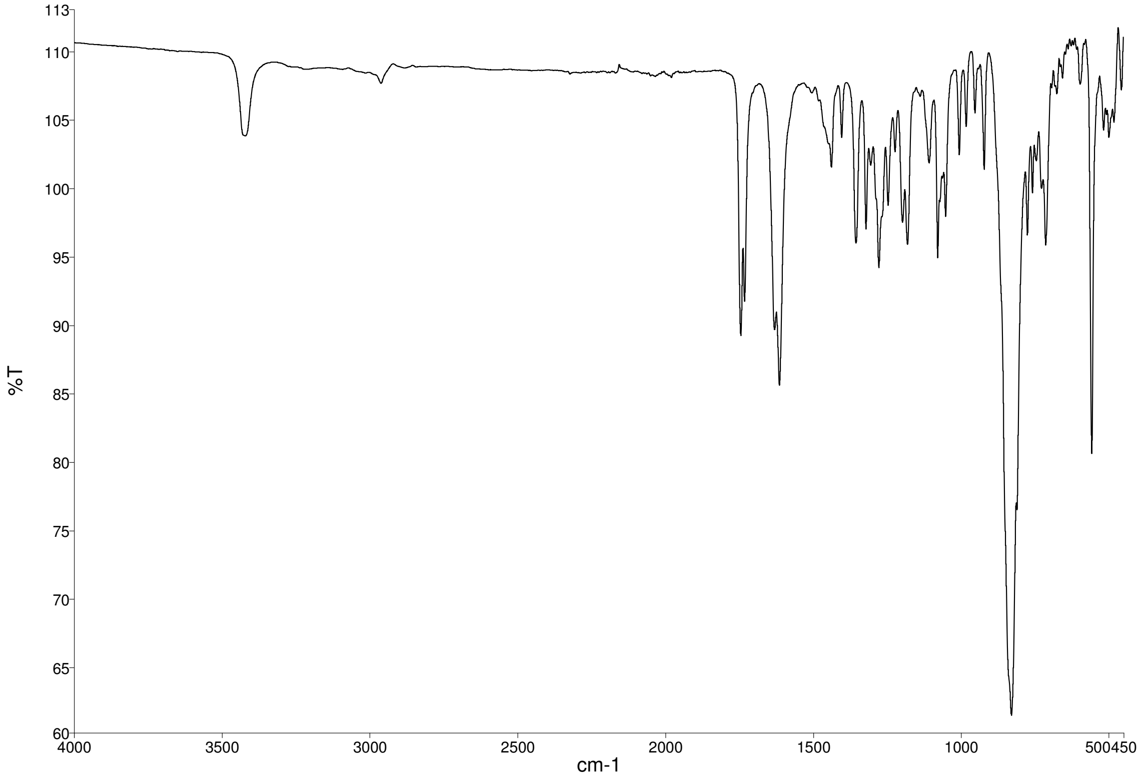

FTIR-ATR spectrum of **5a**·HPF_6.

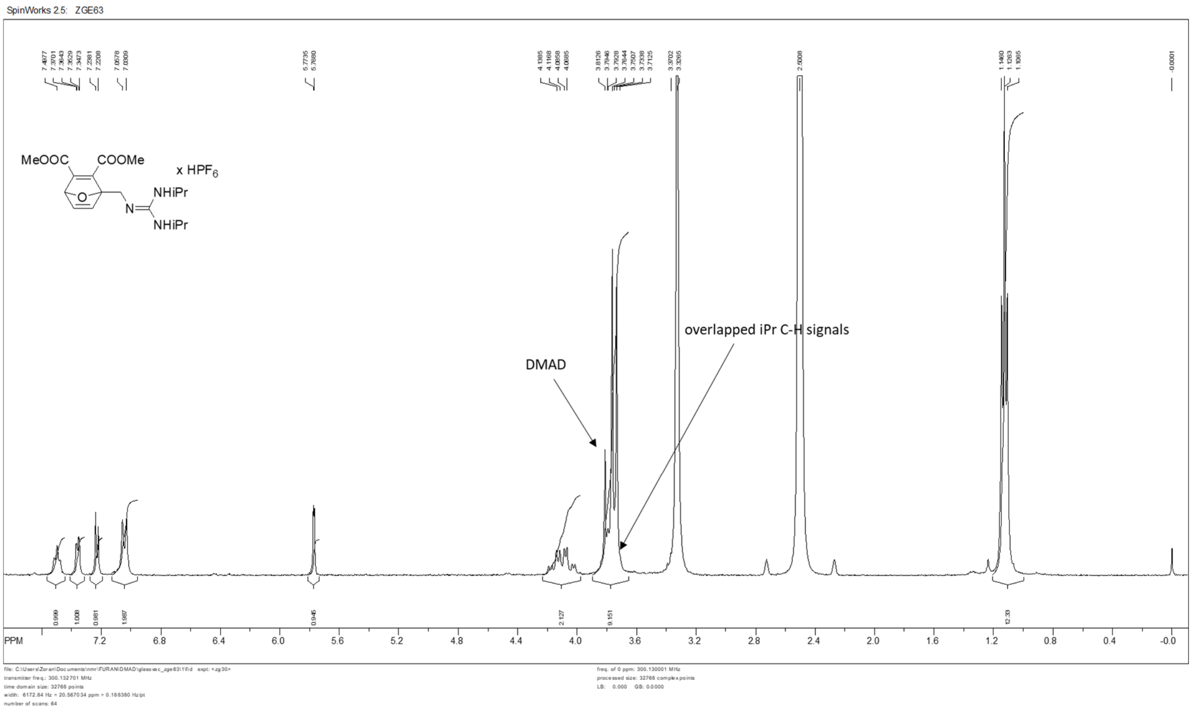

^{1}H NMR spectrum (DMSO-d_6, 300 MHz) of the **4b**·HPF_6.

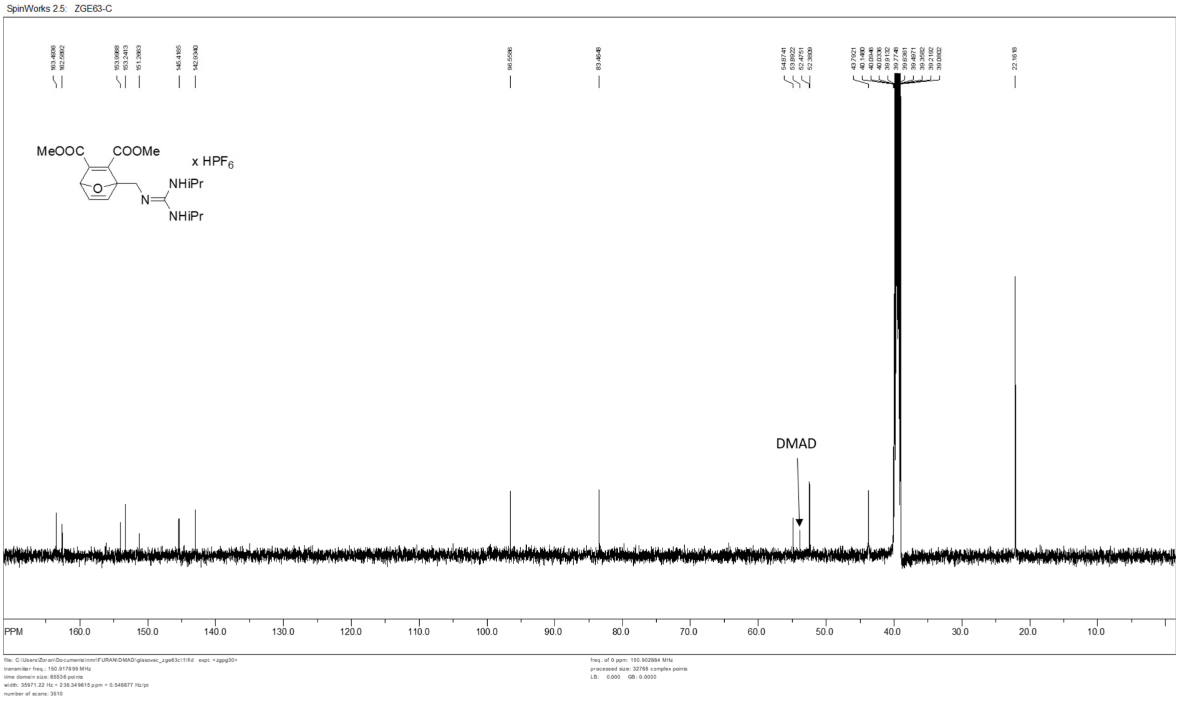

^{13}C NMR spectrum (DMSO-d_6, 75.5 MHz) of the **4b**·HPF_6 (the other two signals from **DMAD** are hidden in the noise).

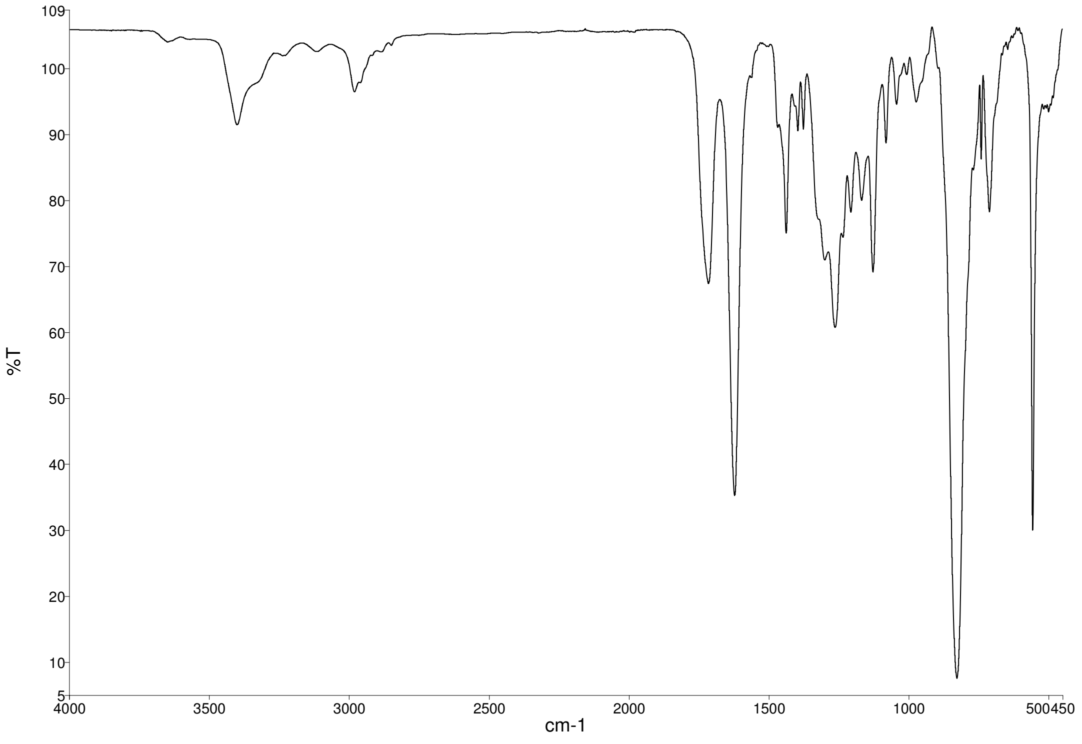

FTIR-ATR spectrum of **4b**·HPF_6.

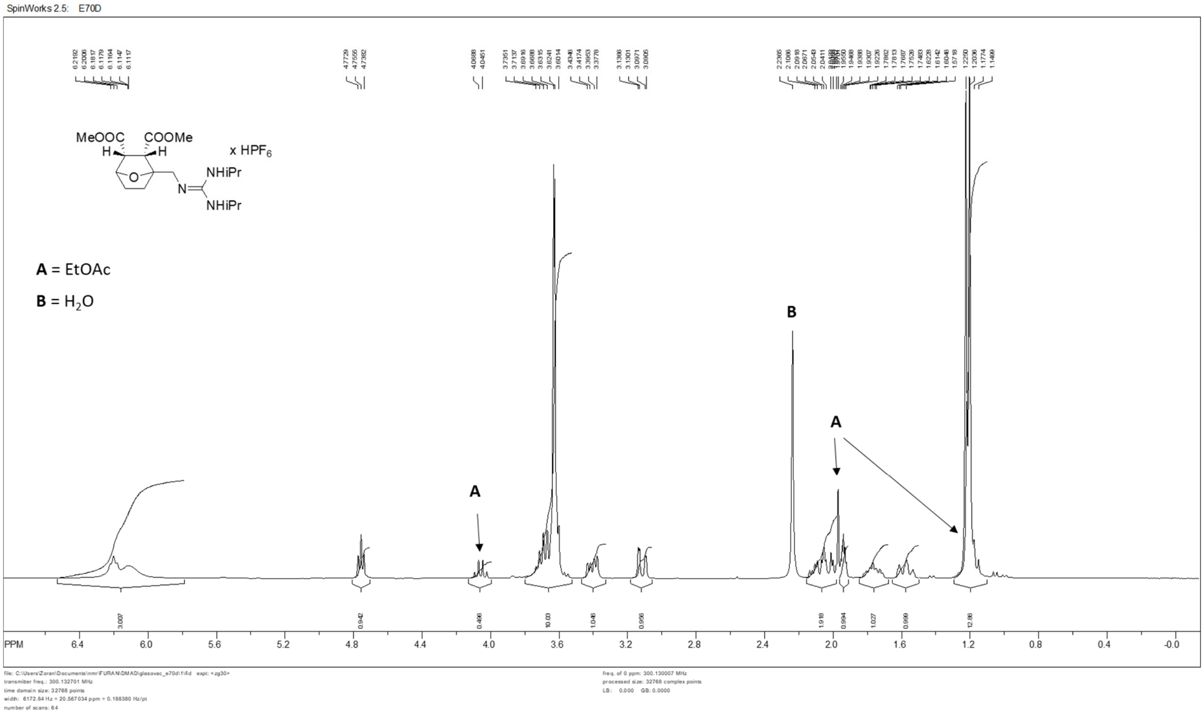

^{1}H NMR spectrum (CD_3CN, 300 MHz) of the **6b**·HPF_6. (contaminated with water and EtOAc)

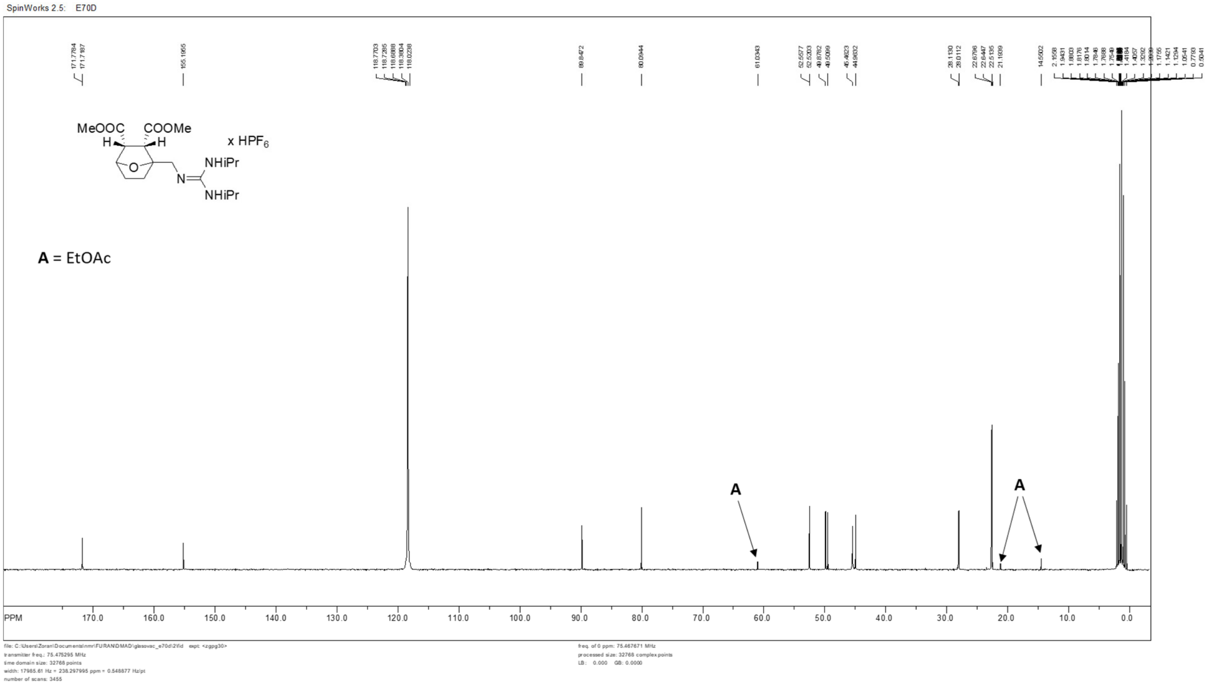

^{13}C NMR spectrum (CD_3CN, 75.5 MHz) of the **6b**·HPF_6. (contaminated with water and EtOAc)

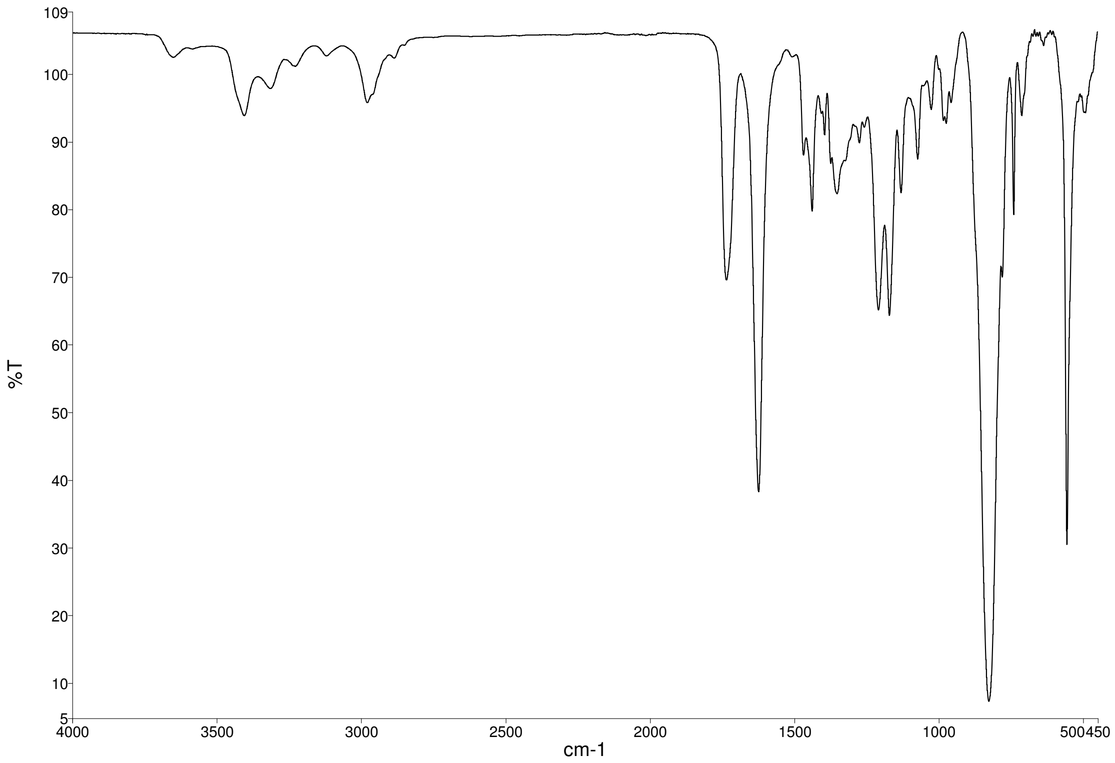

FTIR-ATR spectrum of **6b**·HPF_6.

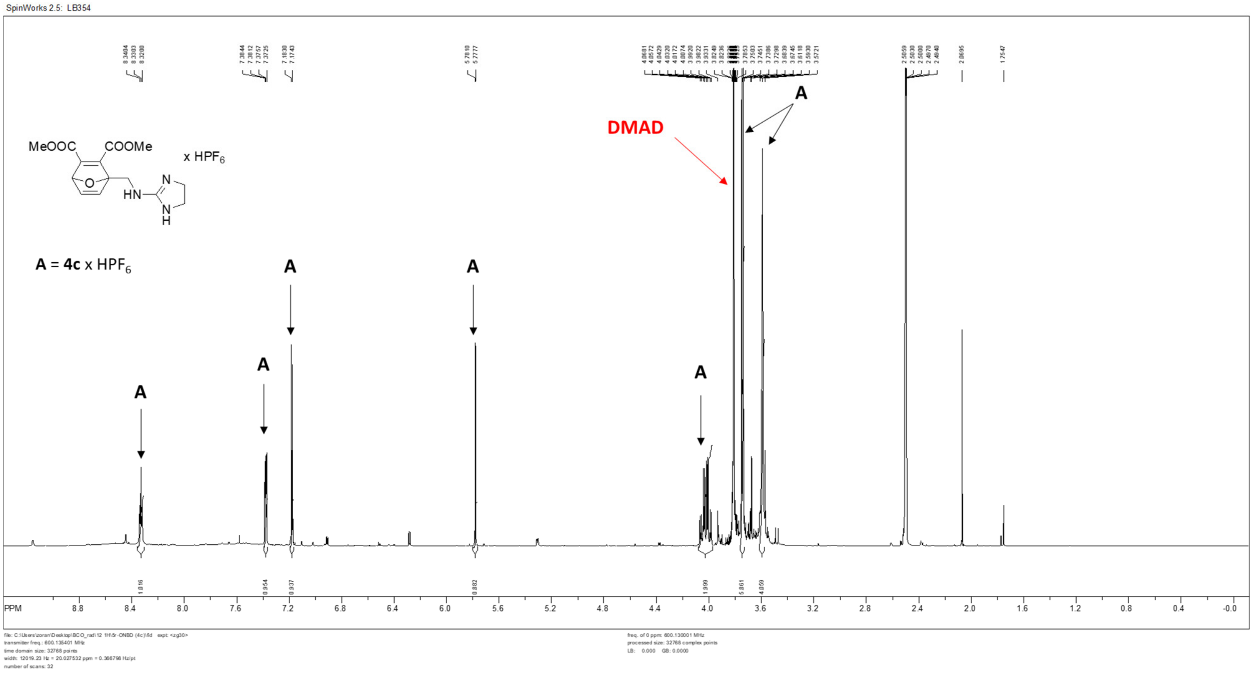

^{1}H NMR spectrum (DMSO-d_6, 600 MHz) of the **4c**·HPF_6.(crude reaction mixture with ca 10 % of **5c**·HPF_6 and significant amount of **DMAD**.)

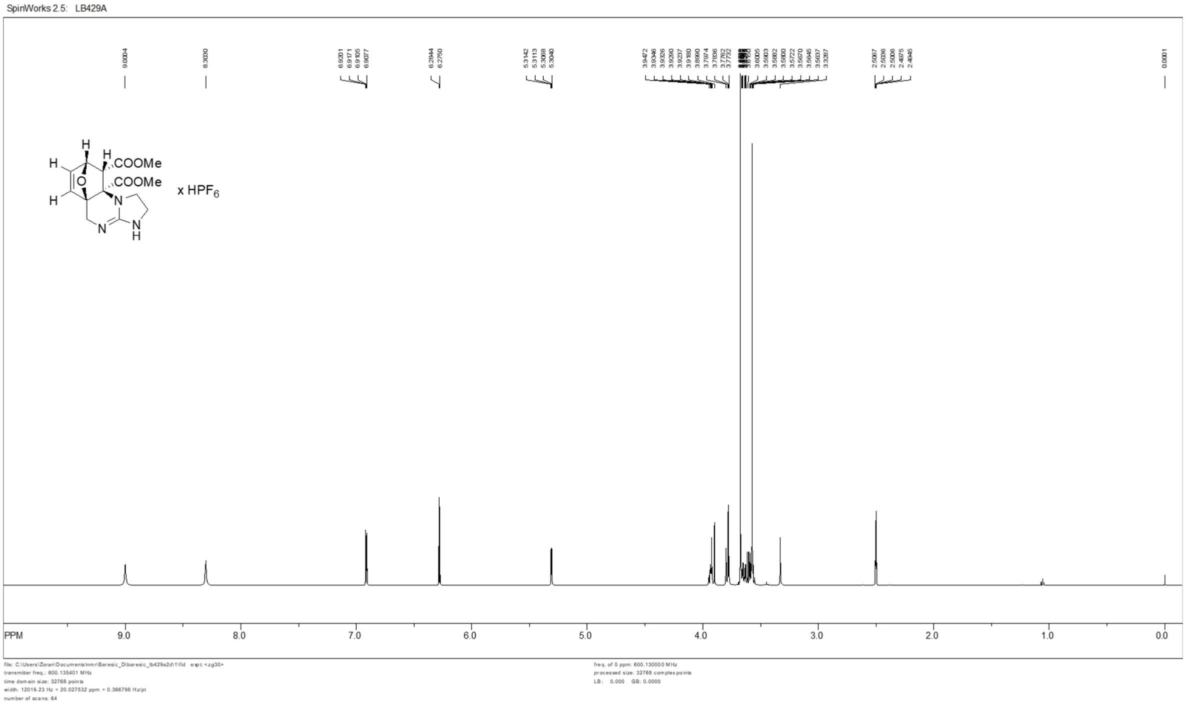

^{1}H NMR spectrum (DMSO-d_6, 600 MHz) of the **5c**·HPF_6.

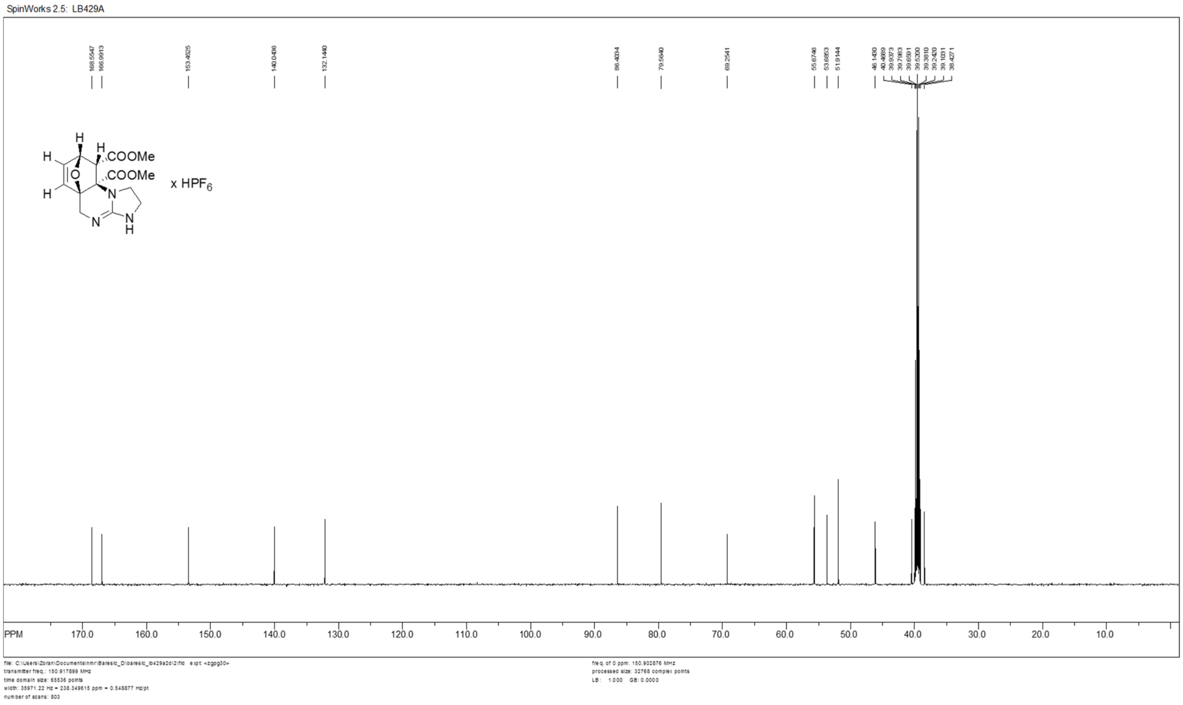

^{13}C NMR spectrum (DMSO-d_6, 150.9 MHz) of the **5c**·HPF_6.

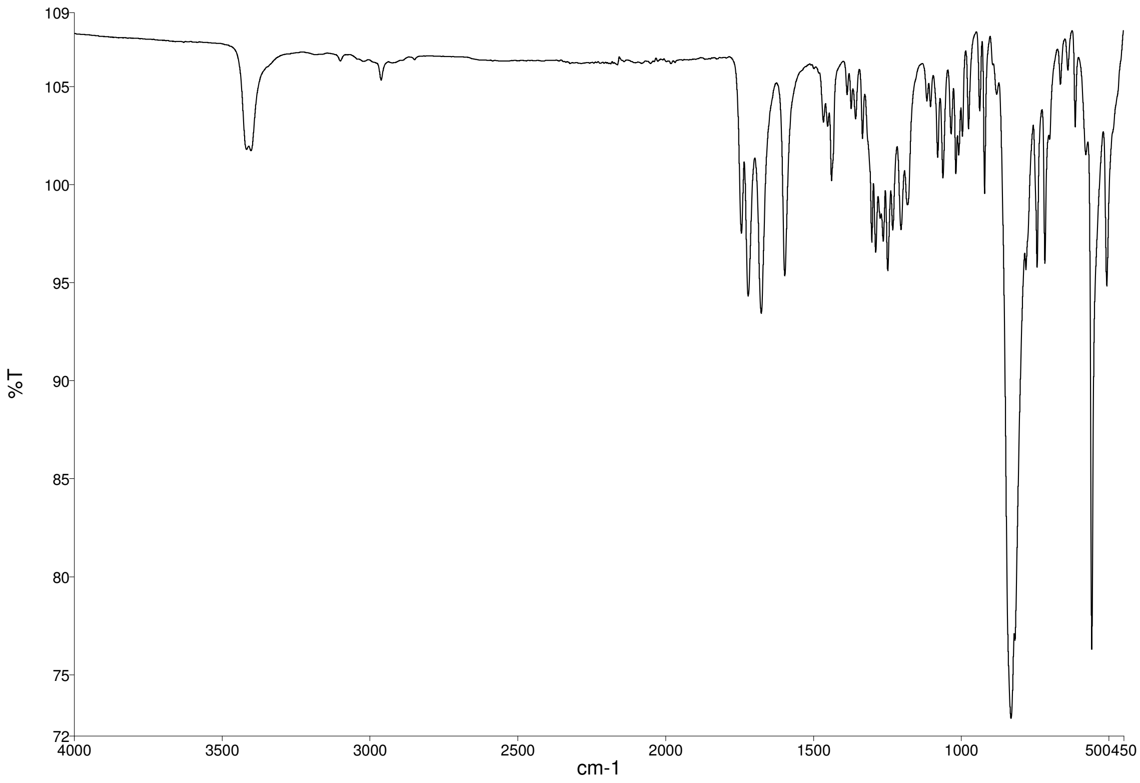

FTIR-ATR spectrum of **5c**·HPF_6.

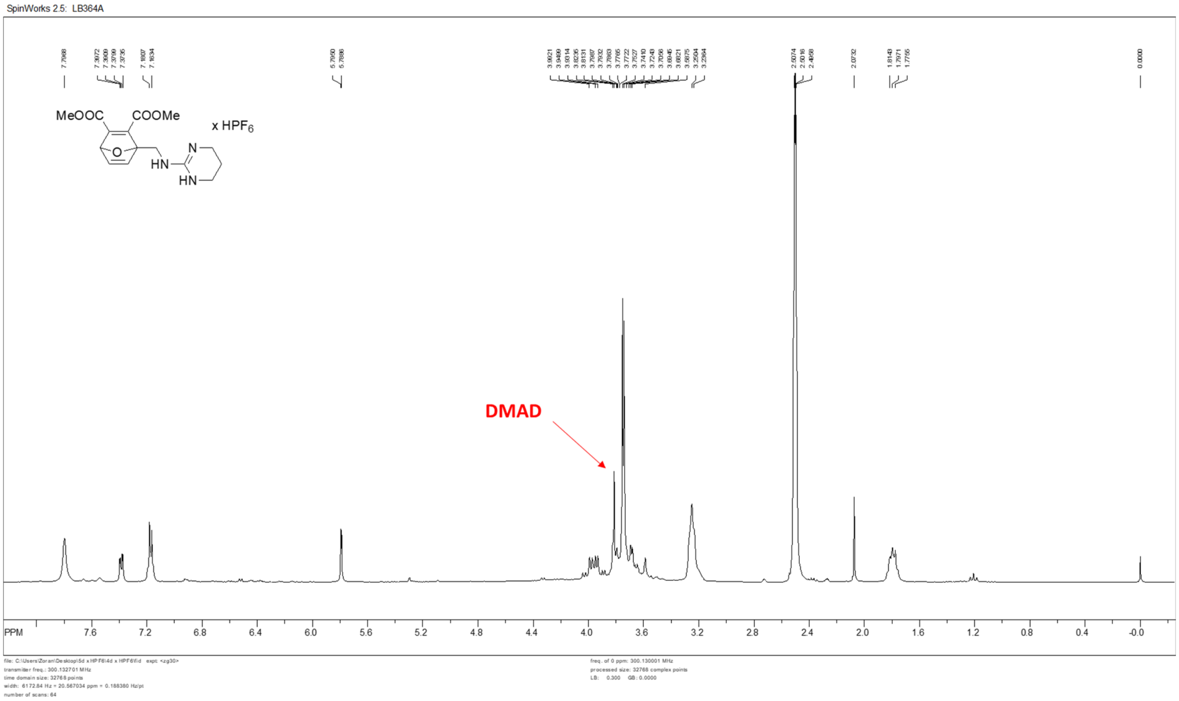

^{1}H NMR spectrum (DMSO-d_6, 300 MHz) of the **4d**·HPF_6.(crude reaction mixture with ca 8 % of **5d**·HPF_6 and small amount of **DMAD**.)

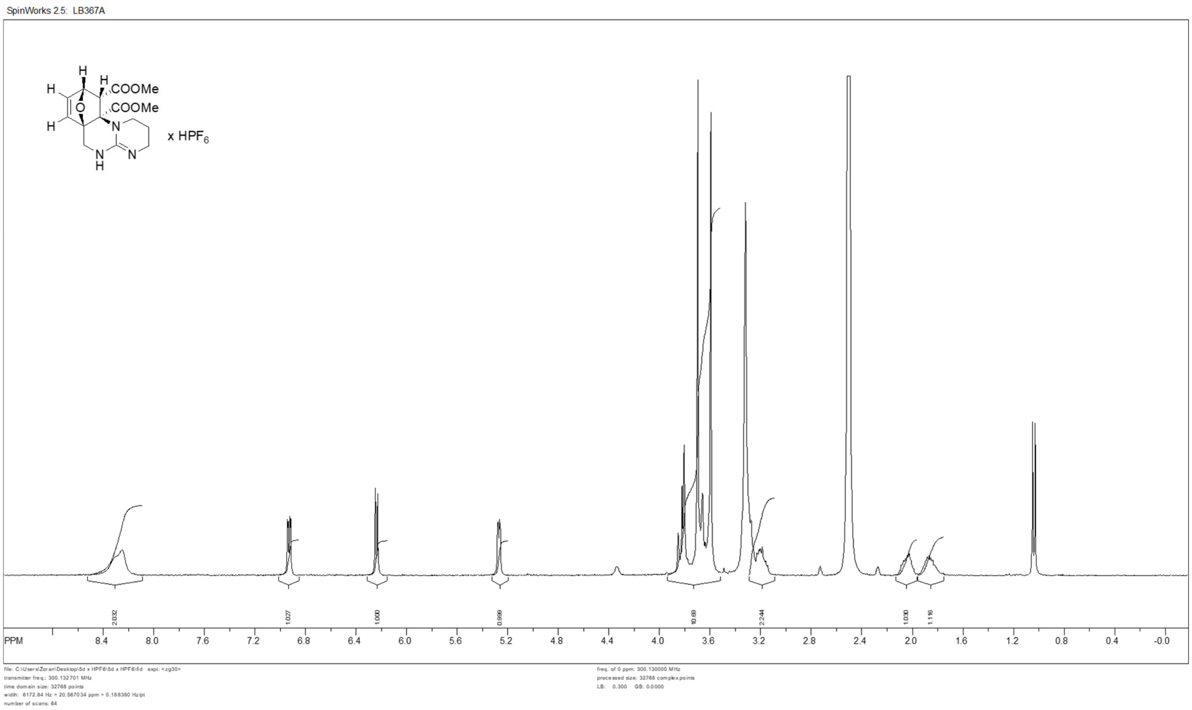

^{1}H NMR spectrum (DMSO-d$_6$, 300 MHz) of the **5d**·HPF$_6$.(crude reaction mixture with small amount of **DMAD** and isopropanol.)

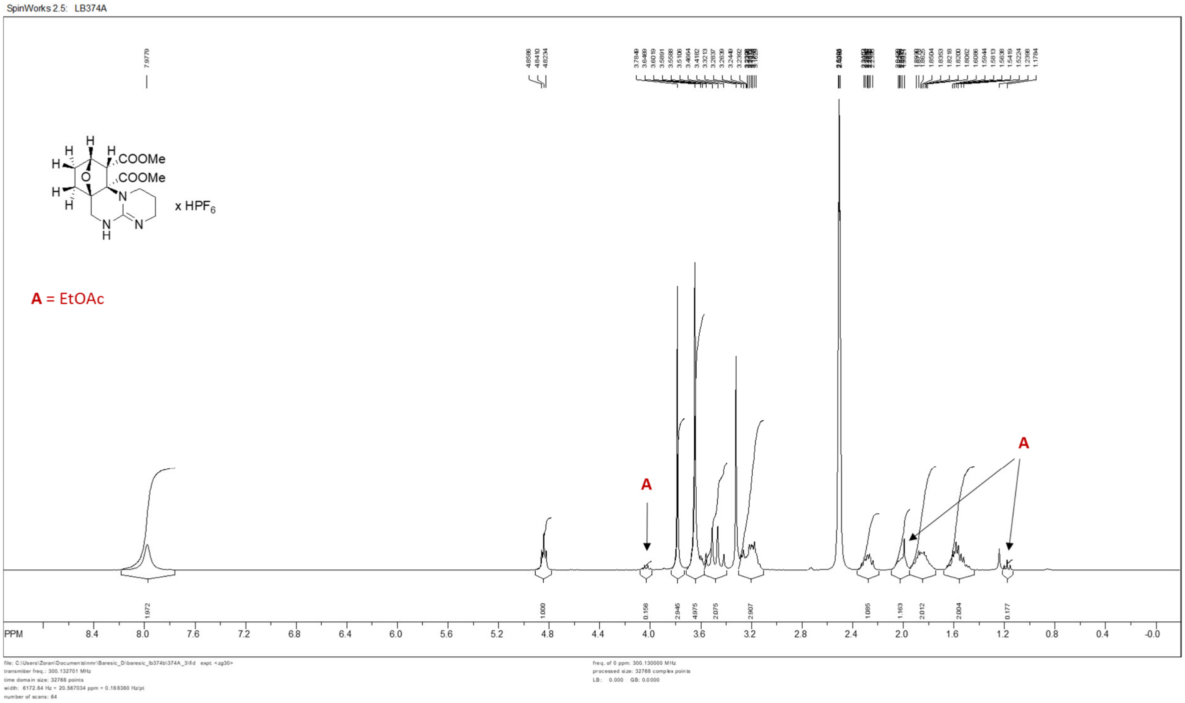

^{1}H NMR spectrum (DMSO-d$_6$, 300 MHz) of the **7d**·HPF$_6$. (contaminated with EtOAc)

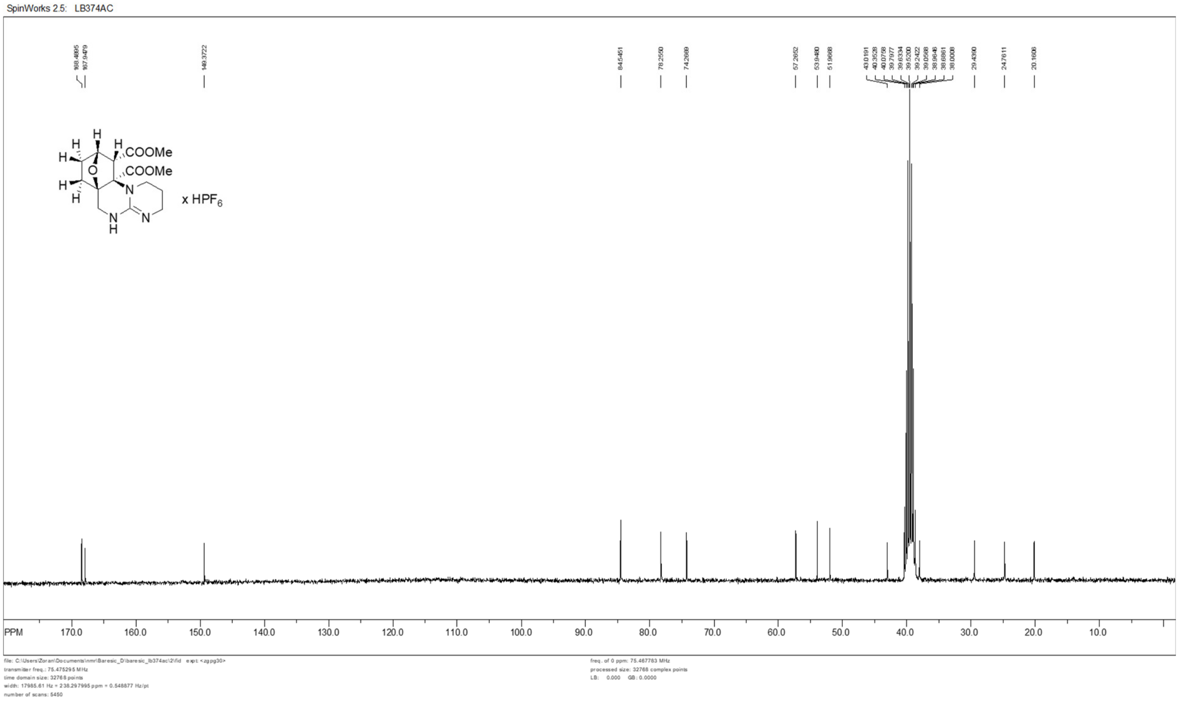

^{13}C NMR spectrum (DMSO-d$_6$, 75.5 MHz) of the **7d**·HPF$_6$.

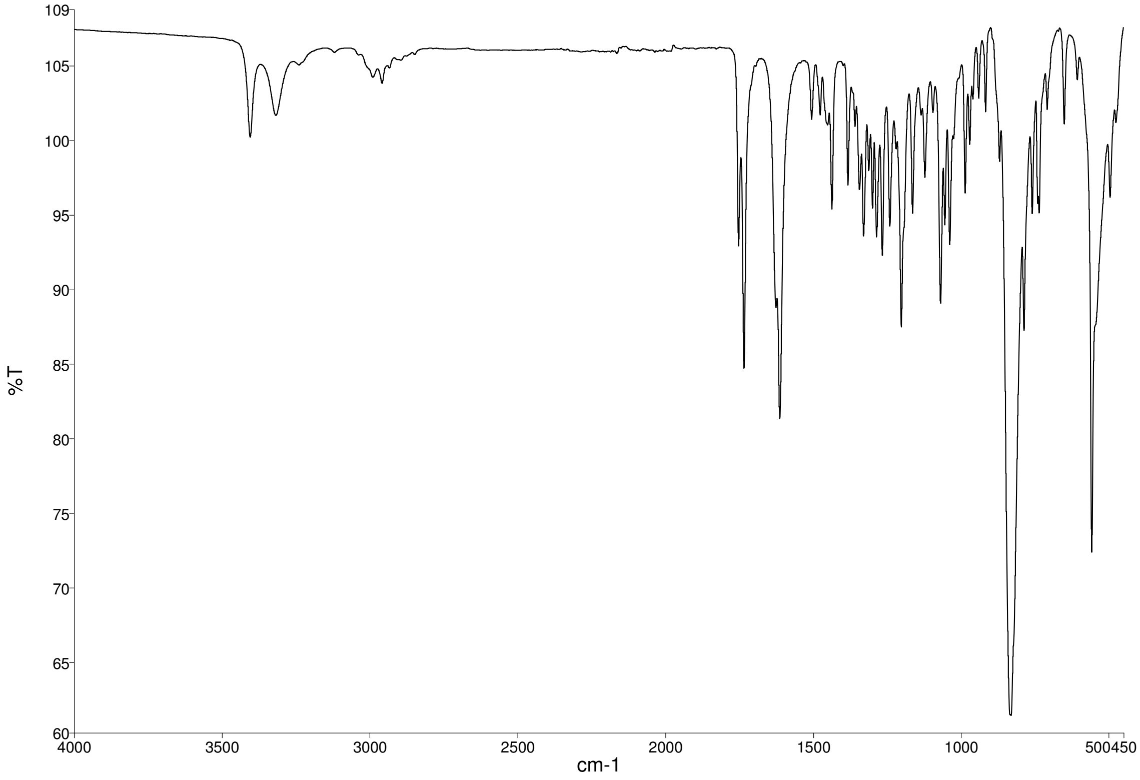

FTIR-ATR spectrum of **7d**·HPF_6.

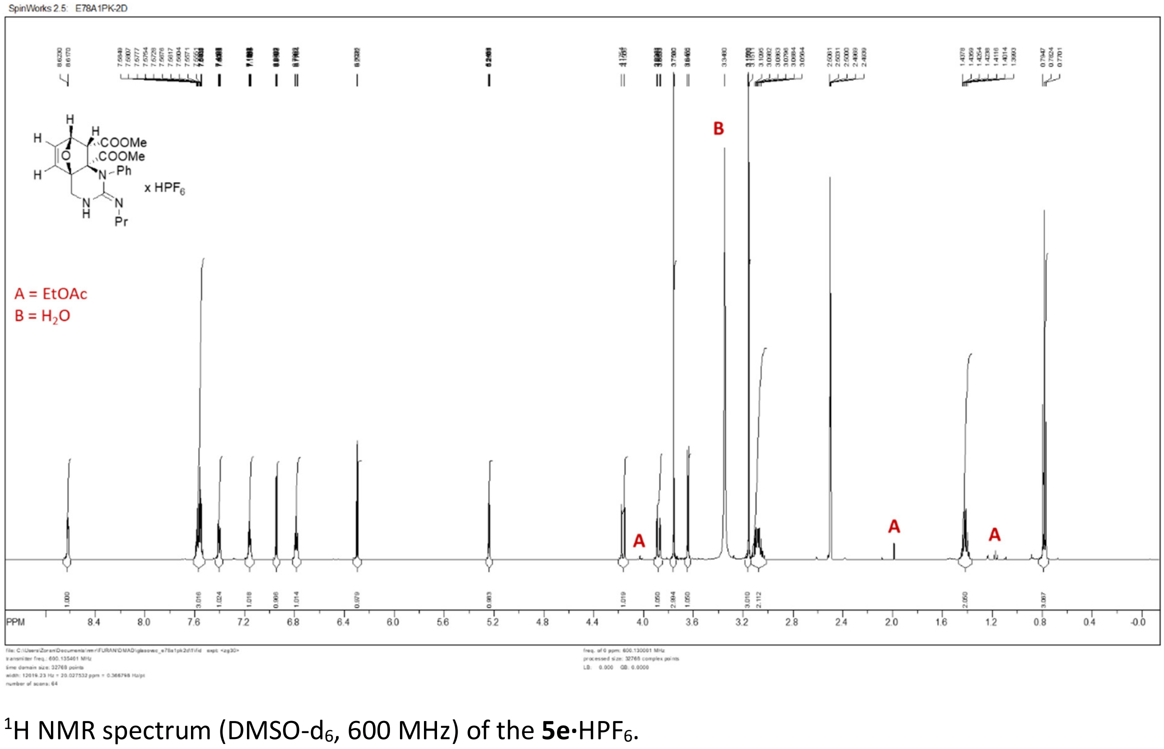

^{1}H NMR spectrum (DMSO-d$_6$, 600 MHz) of the **5e**·HPF_6.

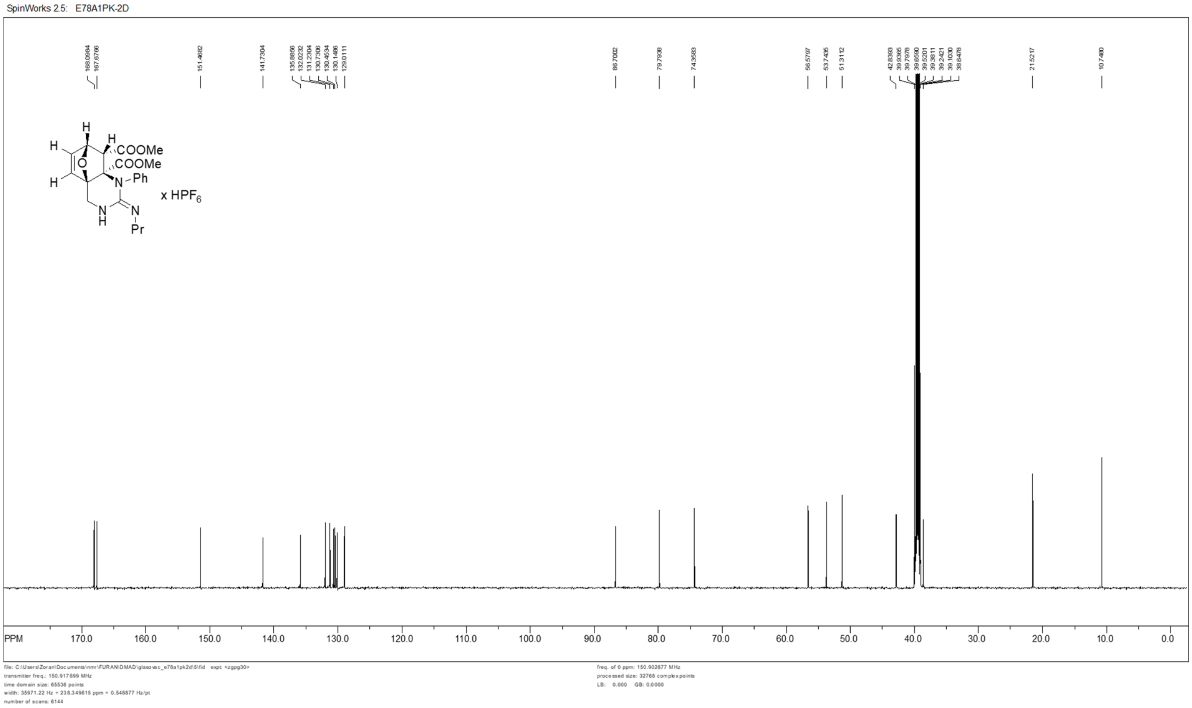

^{13}C NMR spectrum (DMSO-d$_6$, 150.9 MHz) of the **5e**·HPF$_6$.

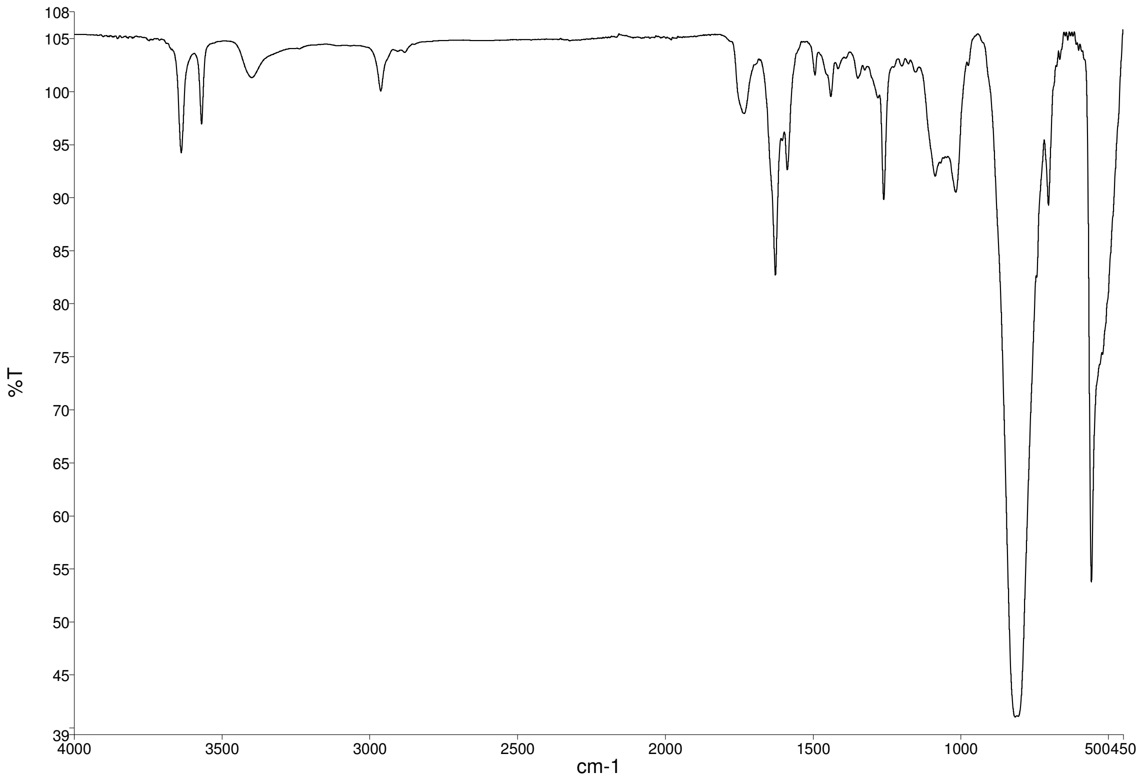

FTIR-ATR spectrum of **5e**·HPF$_6$.

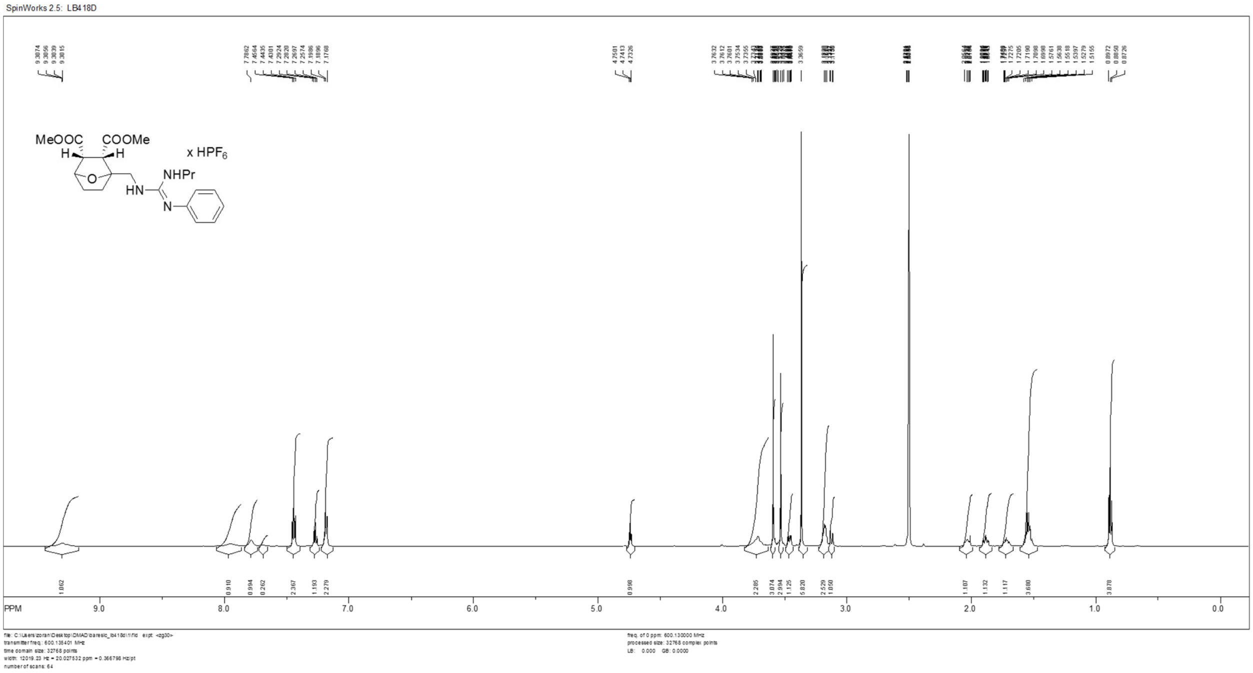

^{1}H NMR spectrum (DMSO-d_6, 600 MHz) of the **6e**·HPF_6.

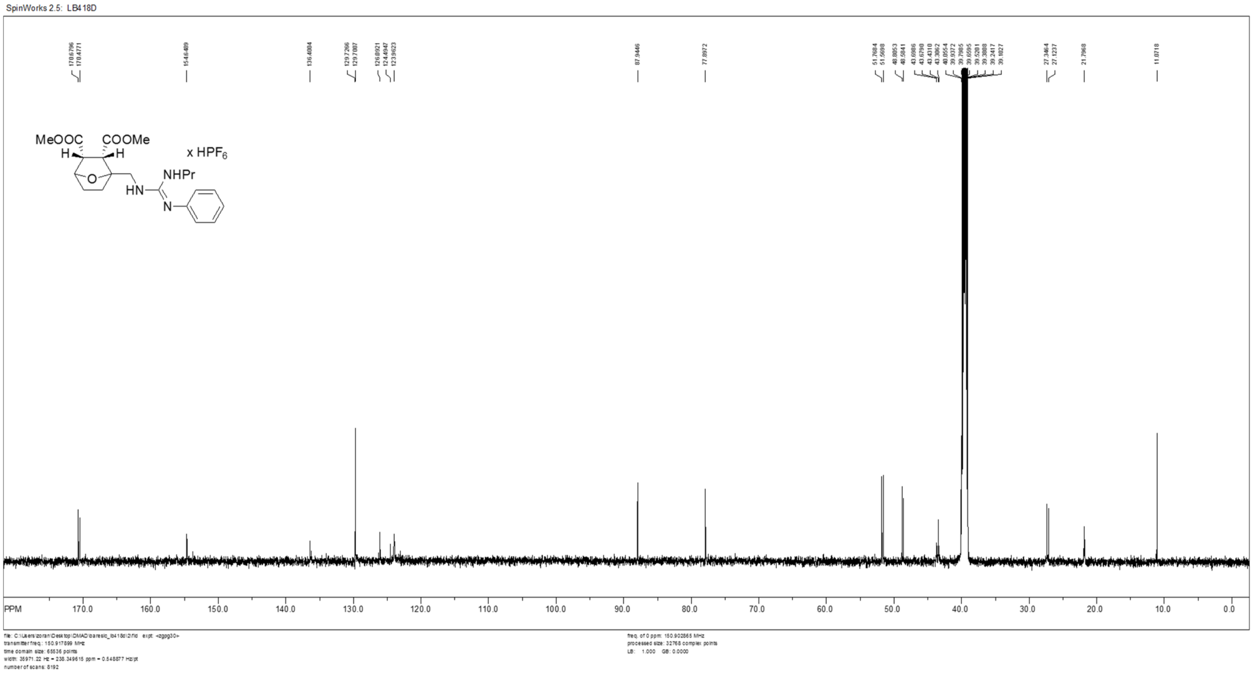

^{13}C NMR spectrum (DMSO-d_6, 150.9 MHz) of the **6e**·HPF_6.

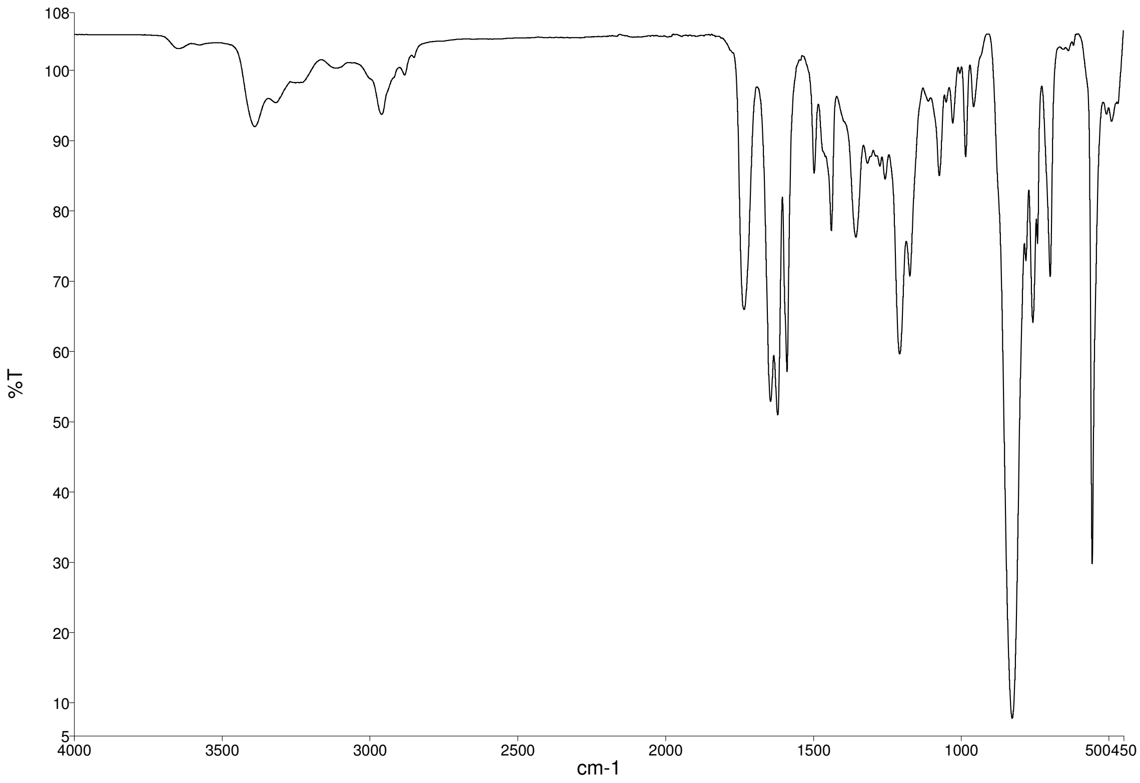

FTIR-ATR spectrum of **6e**·HPF_6.

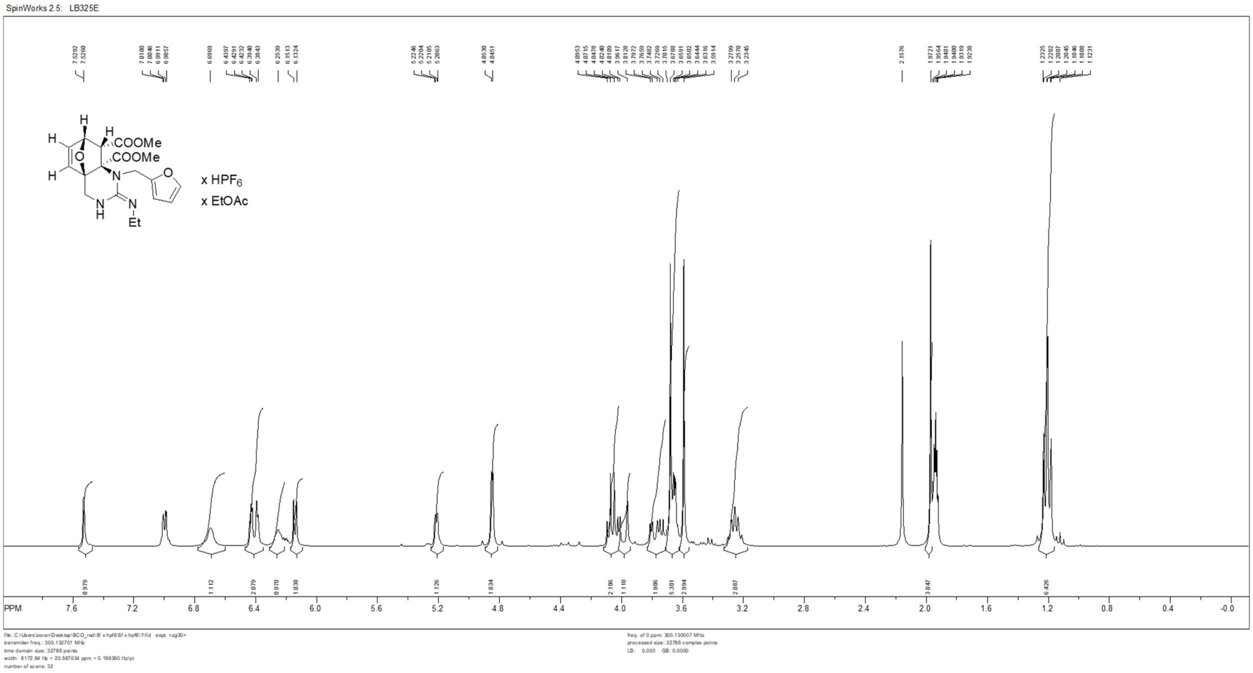

^1H NMR spectrum (DMSO-d_6, 600 MHz) of the **5f**·HPF_6 (solvate with EtOAc in 1 : 1 ratio).

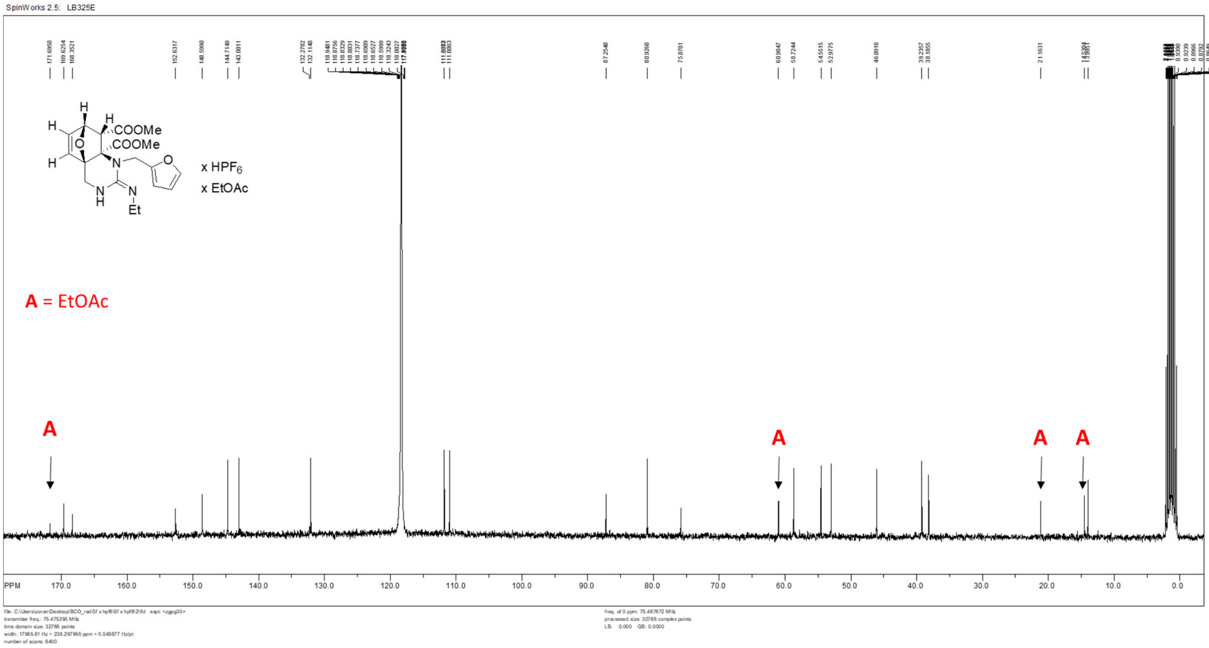

^{13}C NMR spectrum (DMSO-d$_6$, 150.9 MHz) of the **5f**·HPF$_6$. (solvate with EtOAc in 1 : 1 ratio)

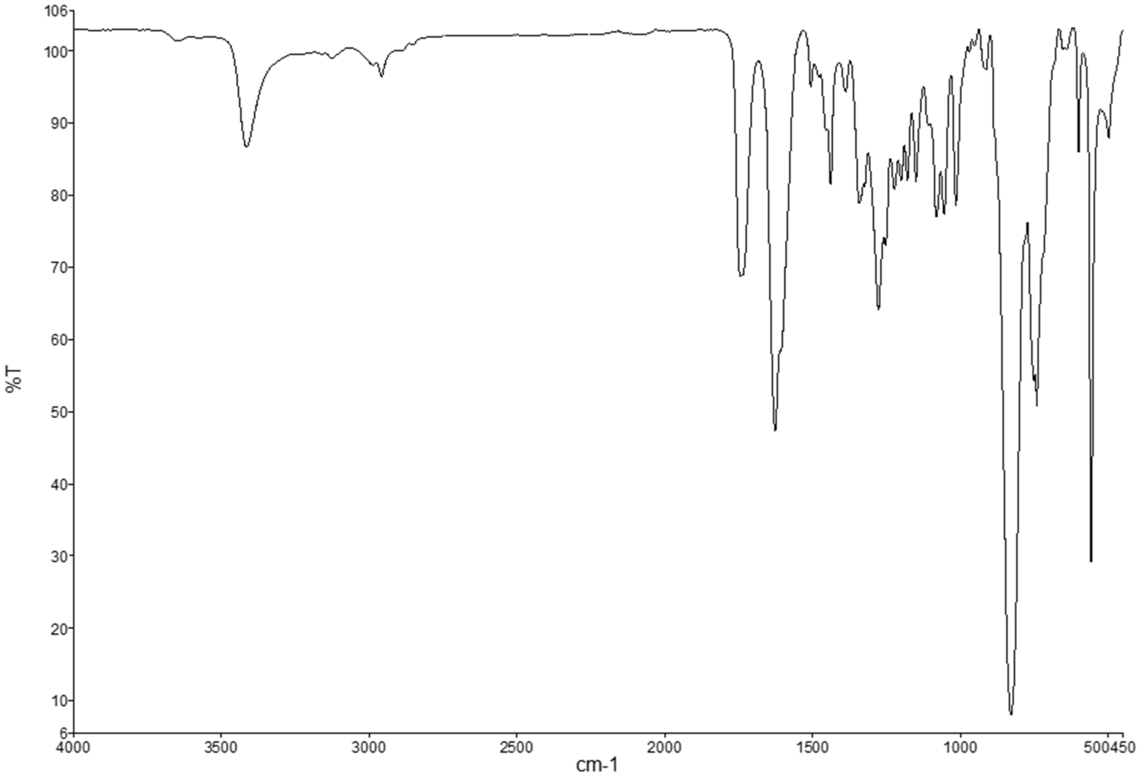

FTIR-ATR spectrum of **5f**·HPF$_6$.

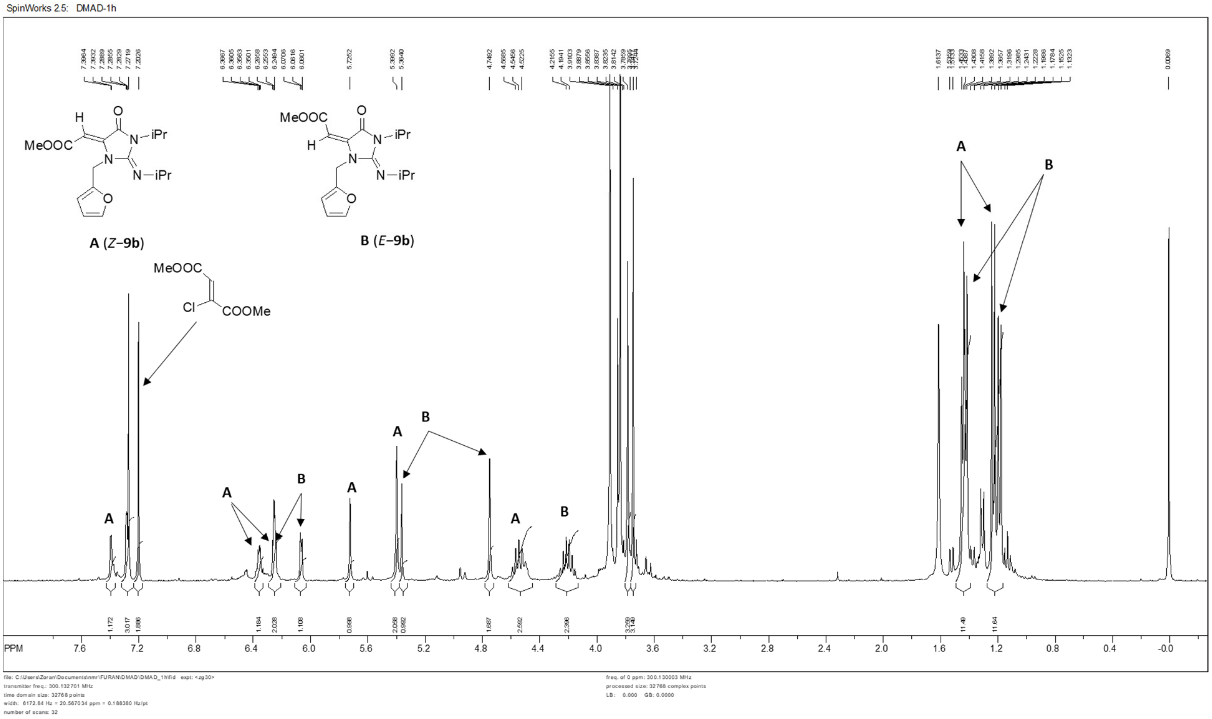

^{1}H NMR spectrum ($CDCl_3$, 300 MHz) of the crude reaction mixture of *Z*–**9b** and *E*–**9b** (Scheme 2 in the main text)

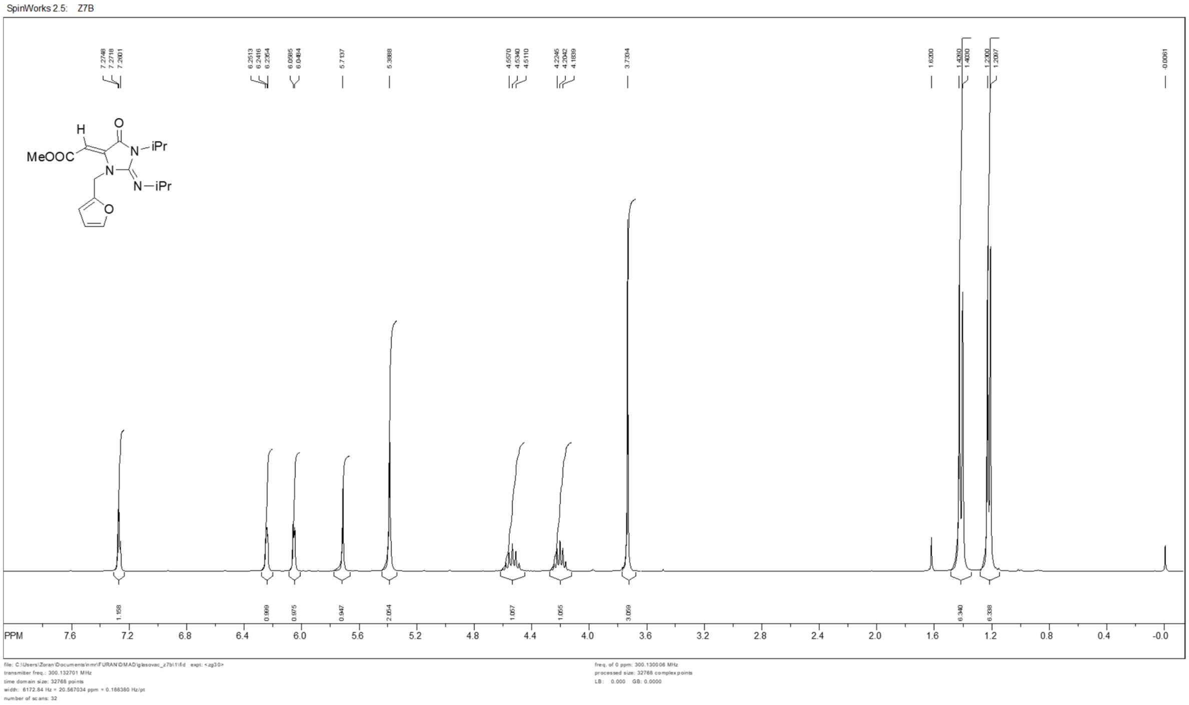

^{1}H NMR spectrum ($CDCl_3$, 300 MHz) of the product *Z*–**9b**

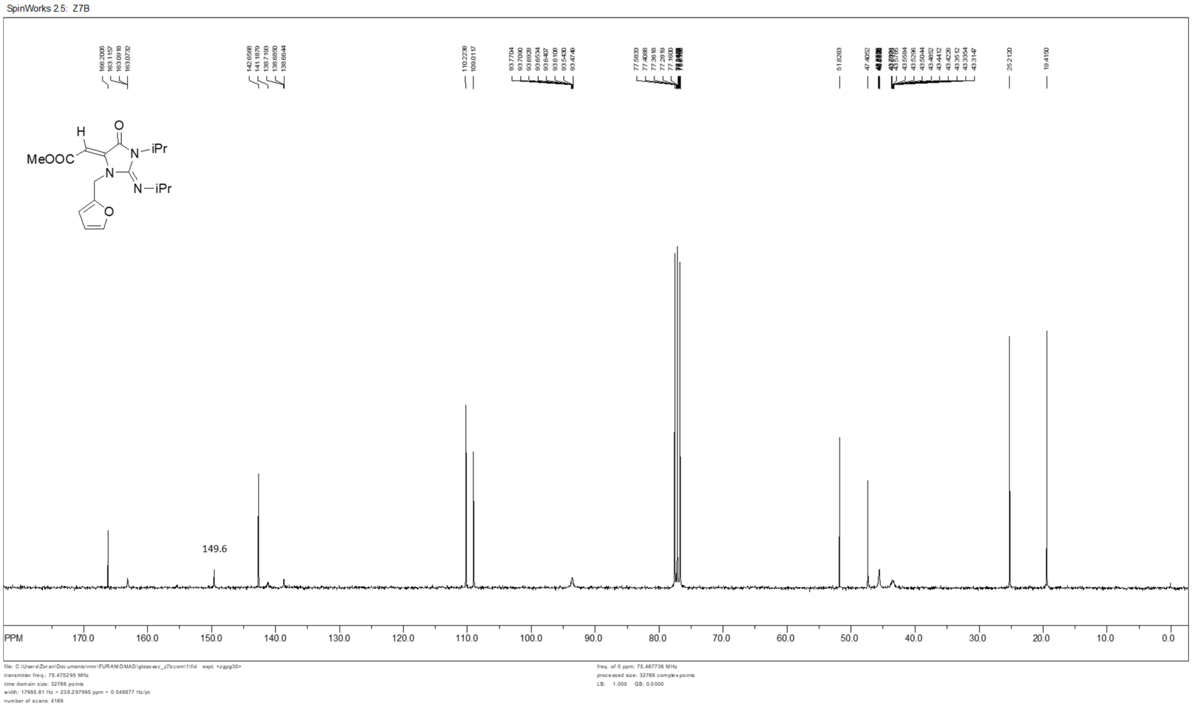

^{13}C NMR spectrum ($CDCl_3$, 75.5 MHz) of the product *Z*–**9b.**

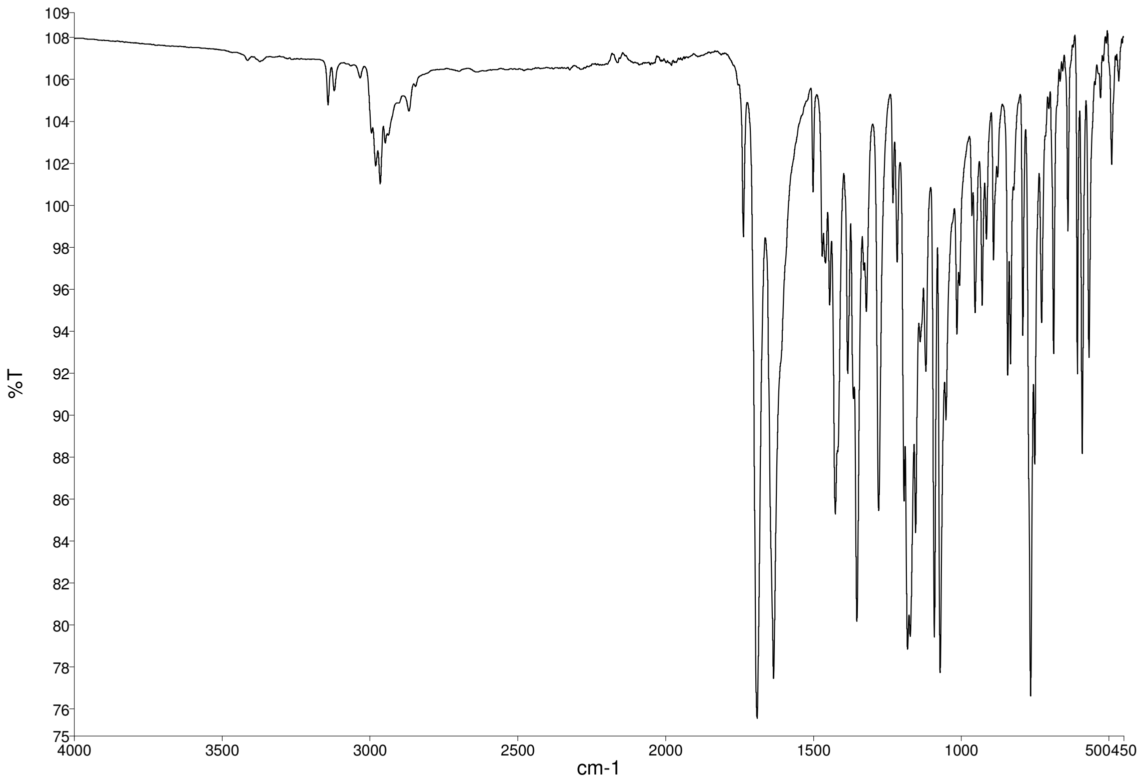

FTIR-ATR spectrum of *Z*–**9b**.

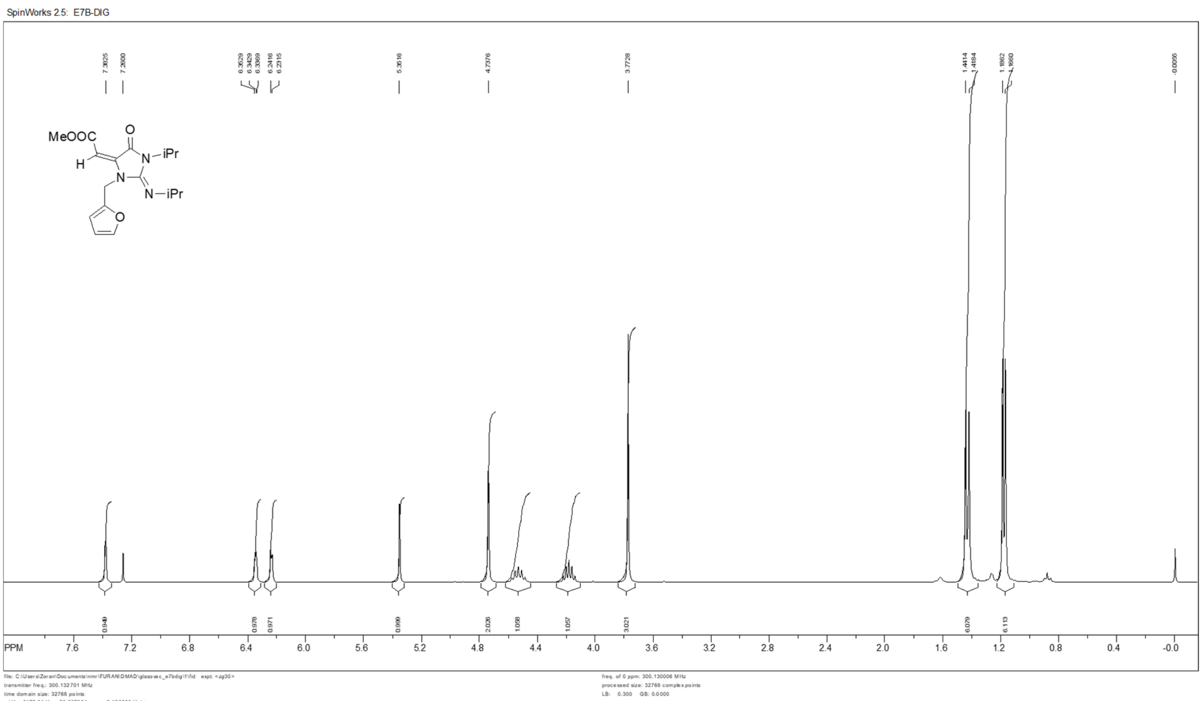

^{1}H NMR spectrum ($CDCl_3$, 300 MHz) of the product *E*–**9b**

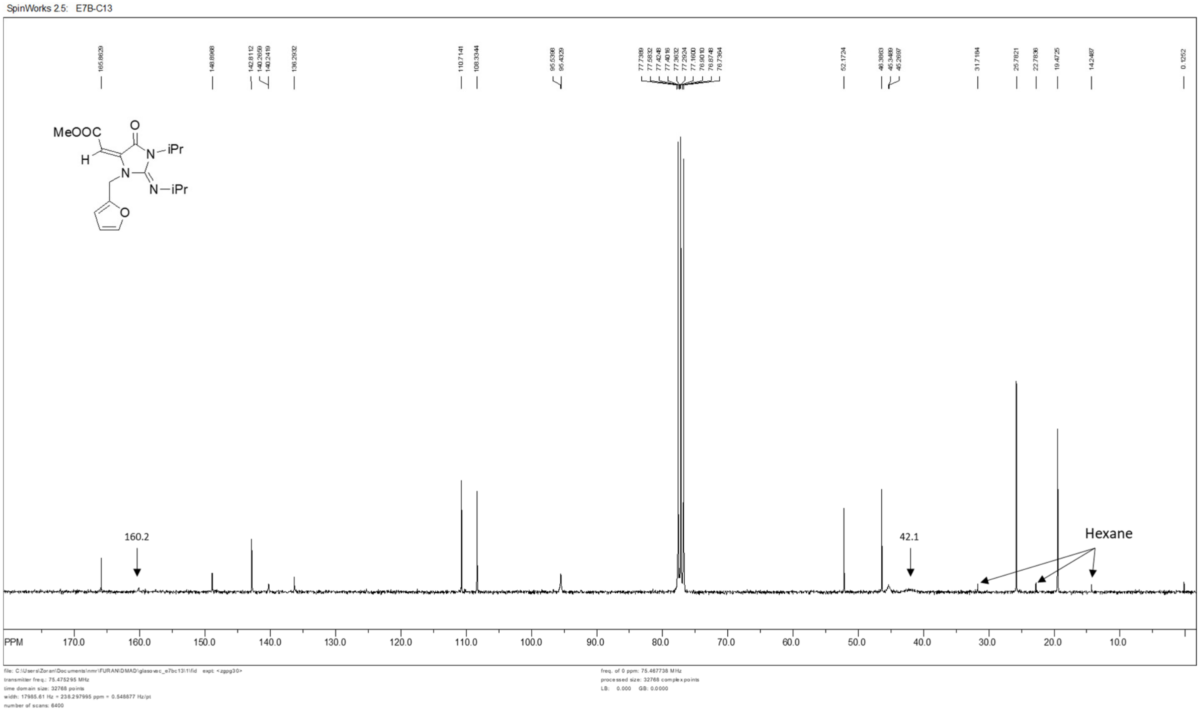

^{13}C NMR spectrum ($CDCl_3$, 75.5 MHz) of the product *E*–**9b**

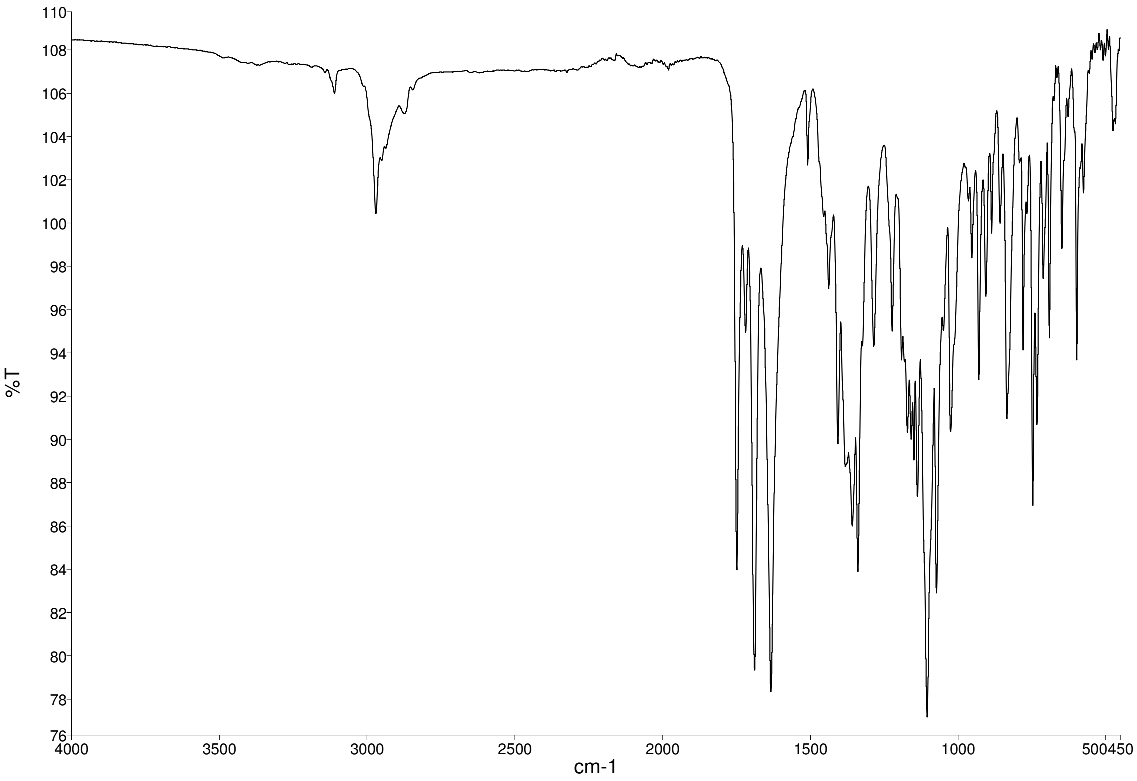

FTIR-ATR spectrum of *E*–**9b**.

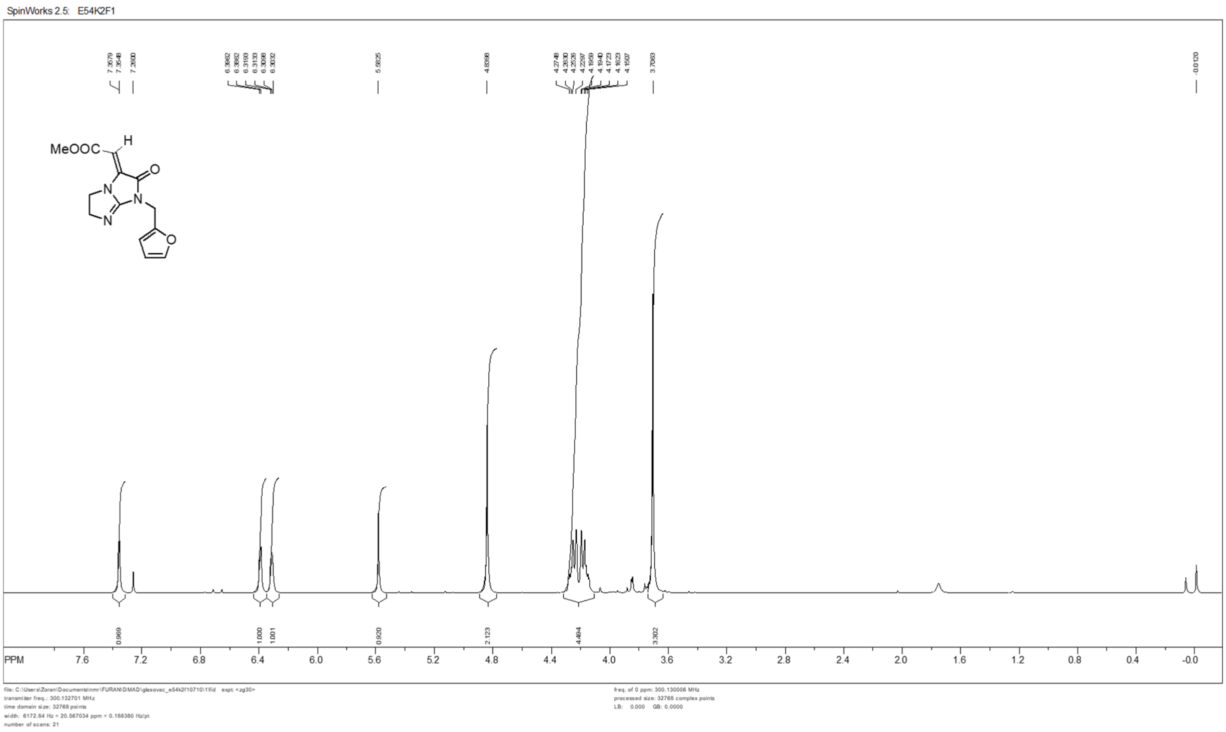

^{1}H NMR spectrum of **10c** ($CDCl_3$, 300 MHz)

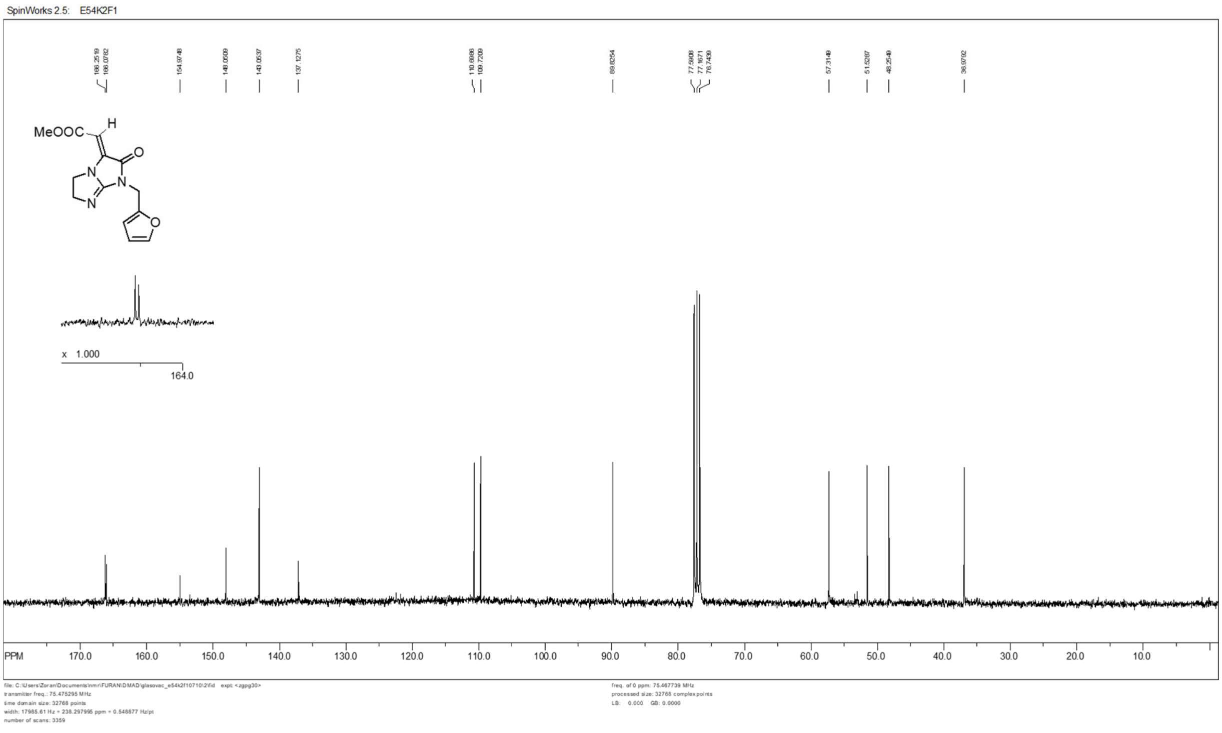

^{13}C NMR spectrum of **10c** ($CDCl_3$, 75.5 MHz)

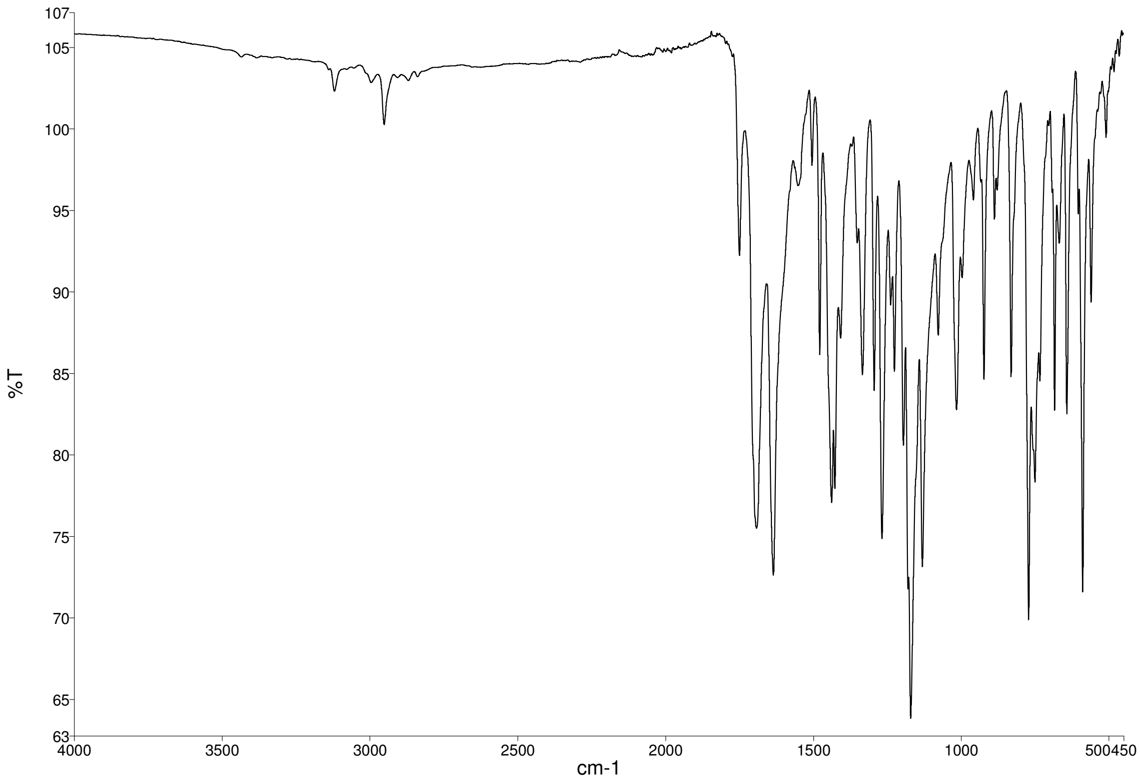

FTIR-ATR spectrum of **10c**.

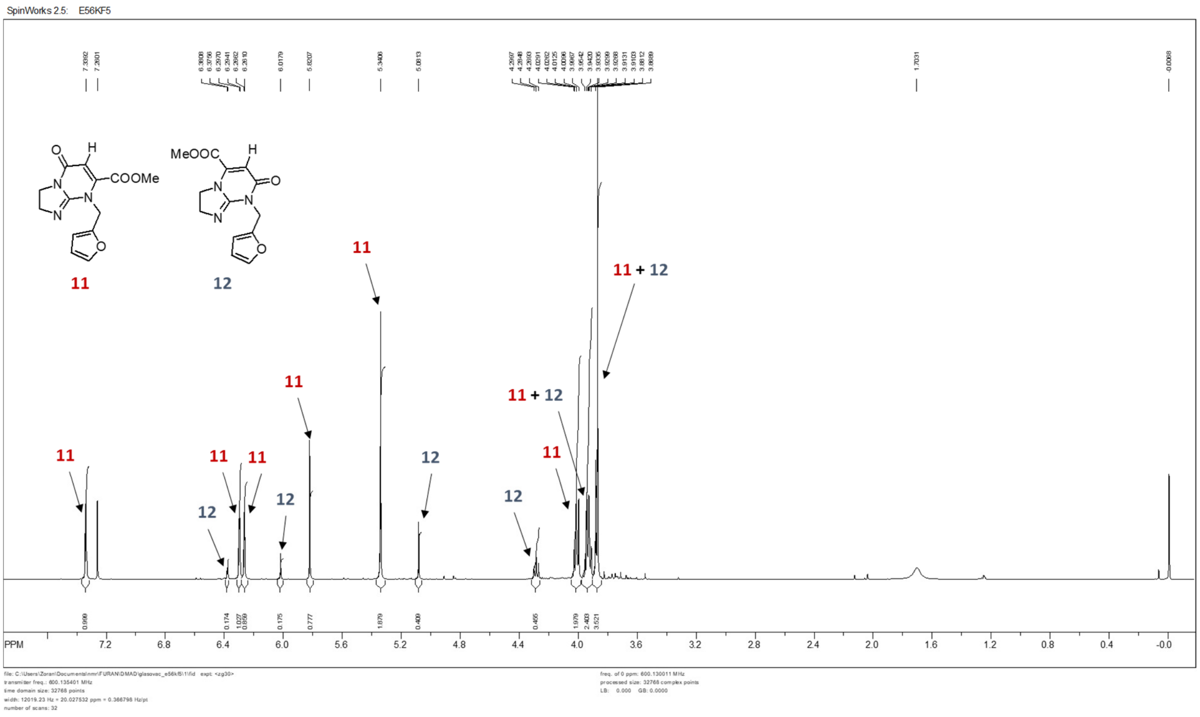

^{1}H NMR spectrum ($CDCl_3$, 600 MHz) of the mixture **11** : **12** = 78 : 22.

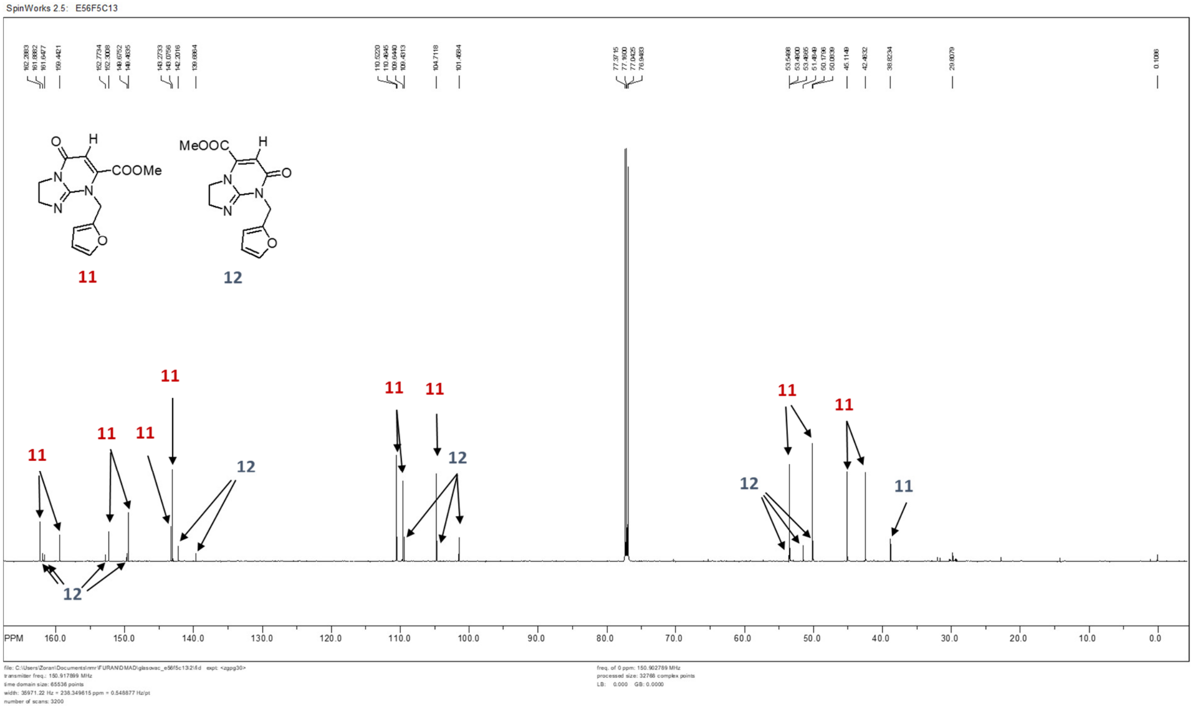

^{13}C NMR spectrum ($CDCl_3$, 150.9 MHz) of the mixture **11** : **12** = 78 : 22. Some minor contaminants at d < 40 ppm do not obscure assignation.

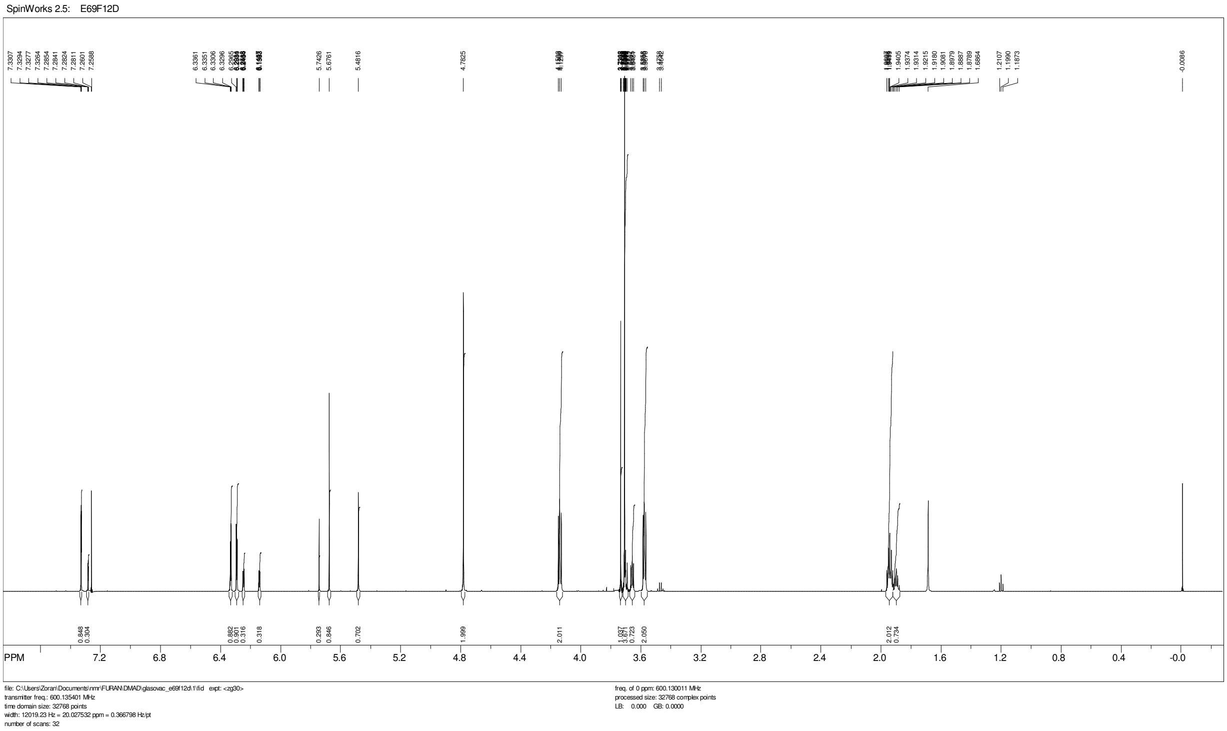

^1H NMR spectrum ($CDCl_3$, 600 MHz) of the mixture of **9d : 10d** = 26 : 74.

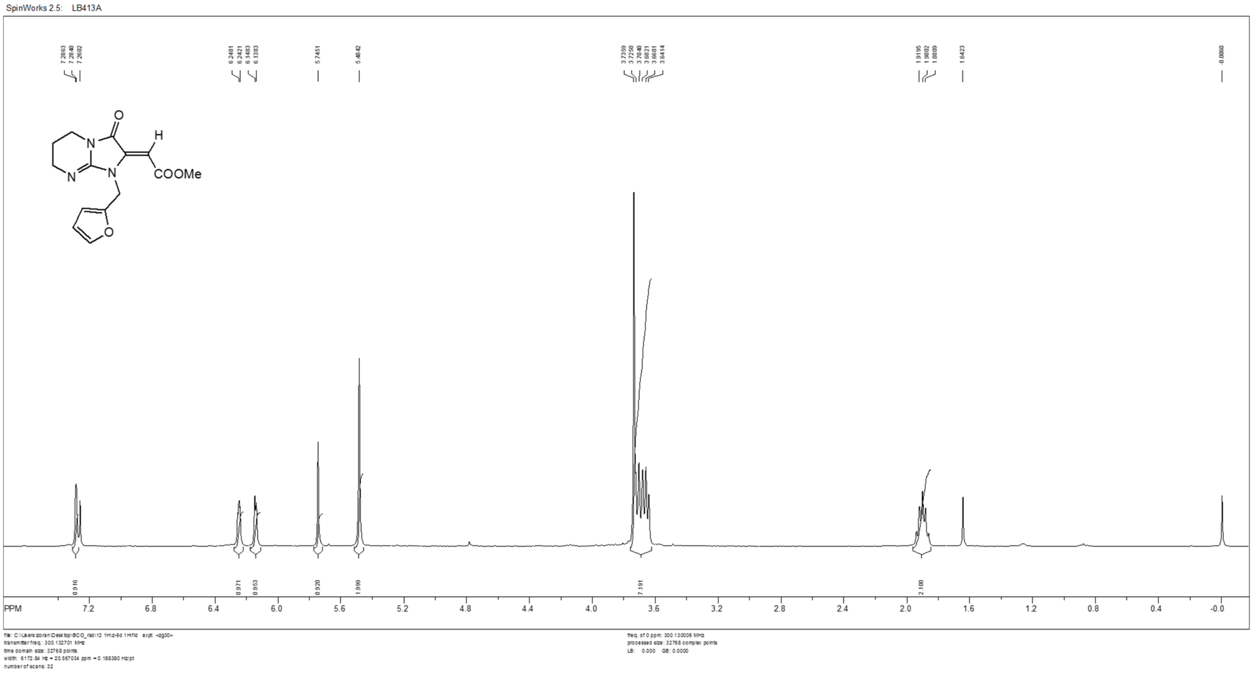

^1H NMR spectrum ($CDCl_3$, 300 MHz) of **9d**. (contaminated with hexane and traces of **10d**)

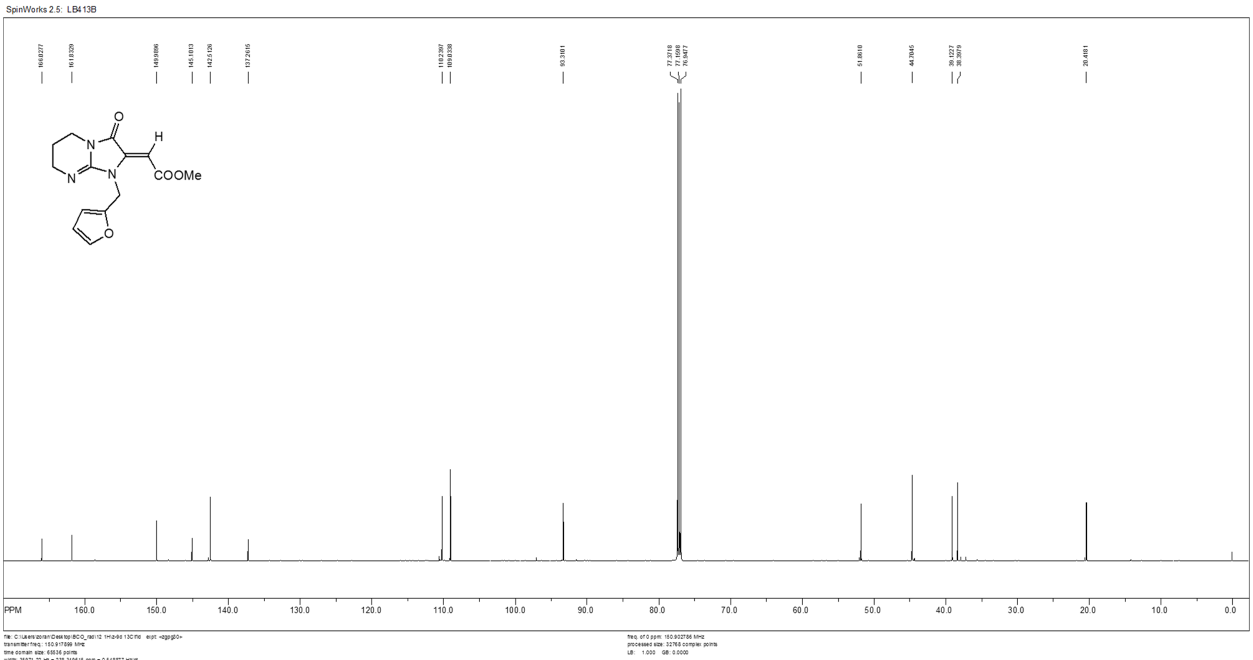

^{13}C NMR spectrum ($CDCl_3$, 75.5 MHz) of **9d**. (contaminated with hexane and traces of **10d**)

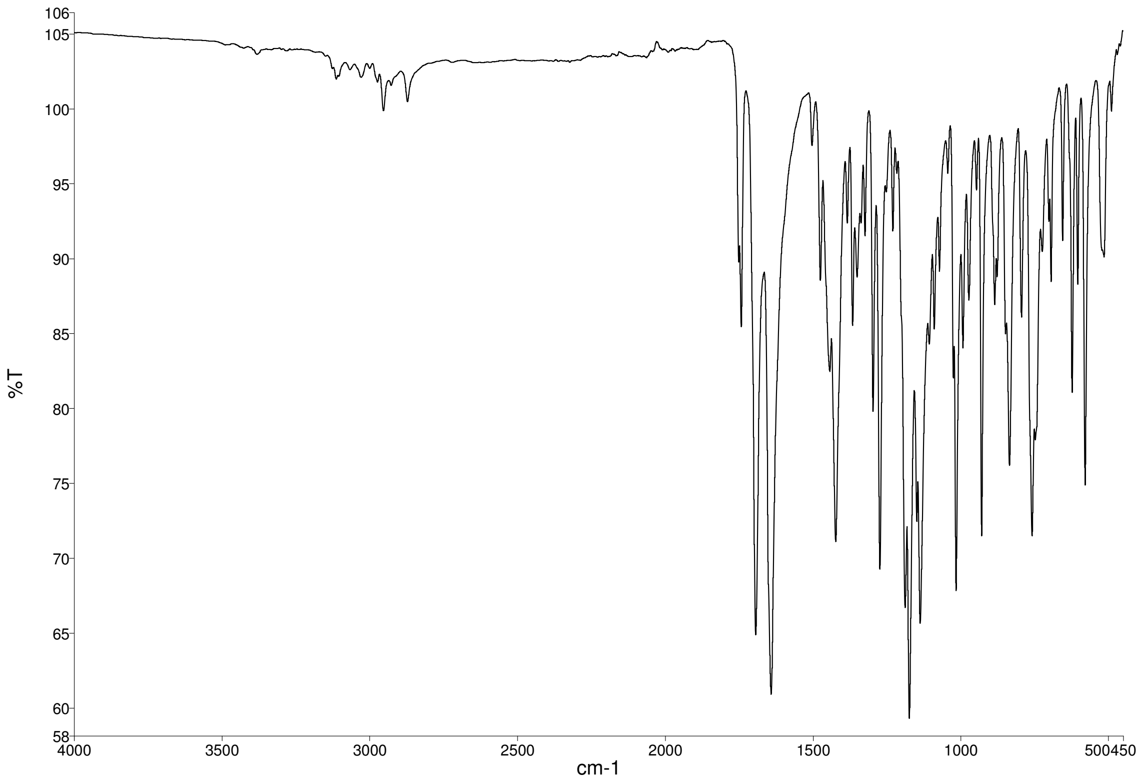

FTIR-ATR spectrum of **9d**.

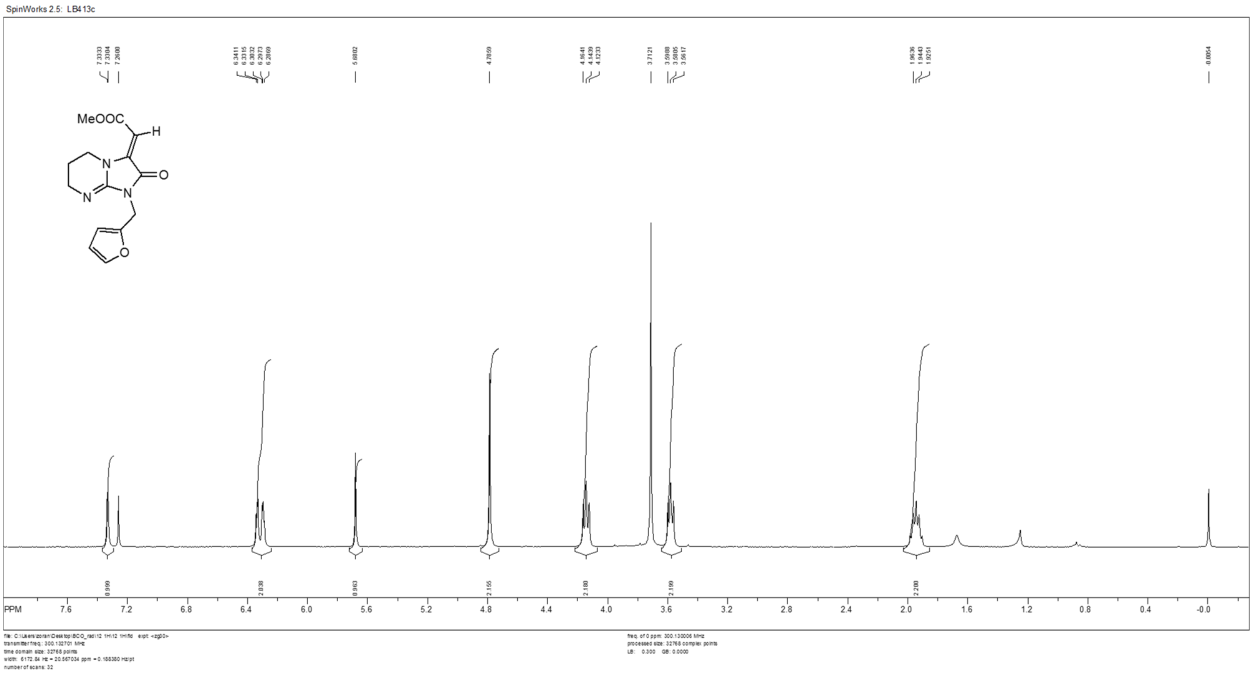

^{1}H NMR spectrum ($CDCl_3$, 300 MHz) of **10d**. (hexane present as residual solvent)

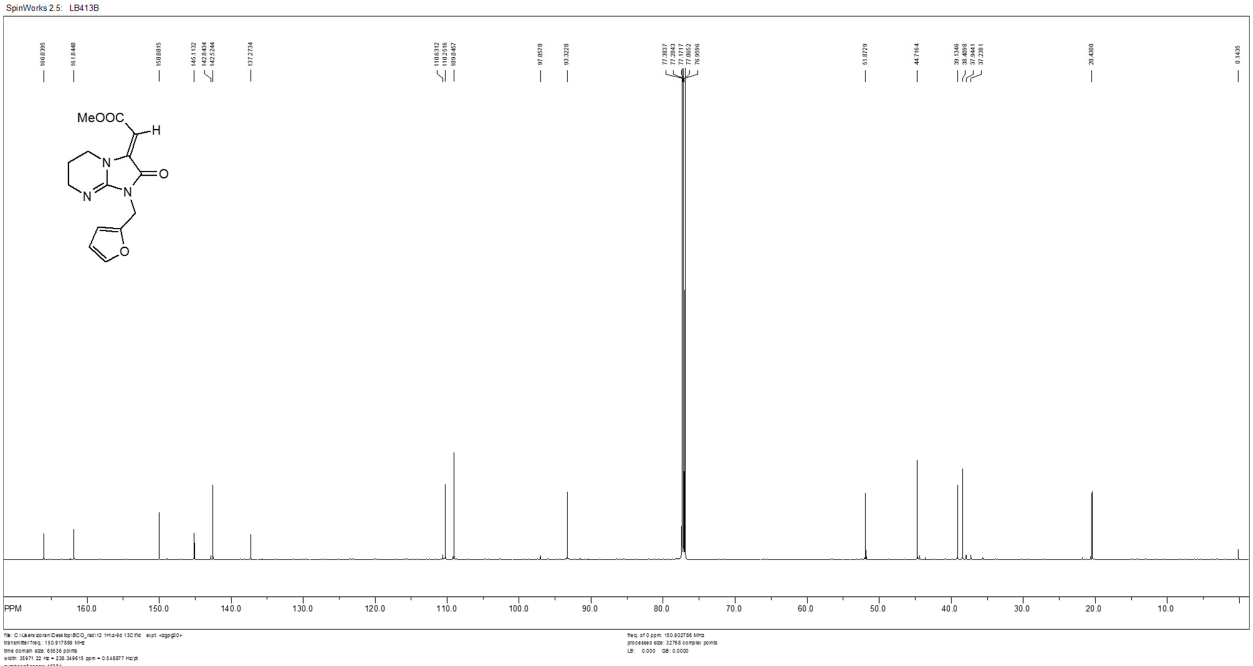

^{13}C NMR spectrum ($CDCl_3$, 75.5 MHz) of **10d**. (Signals for another minor unidentified isomer are visible. Hexane present as residual solvent)

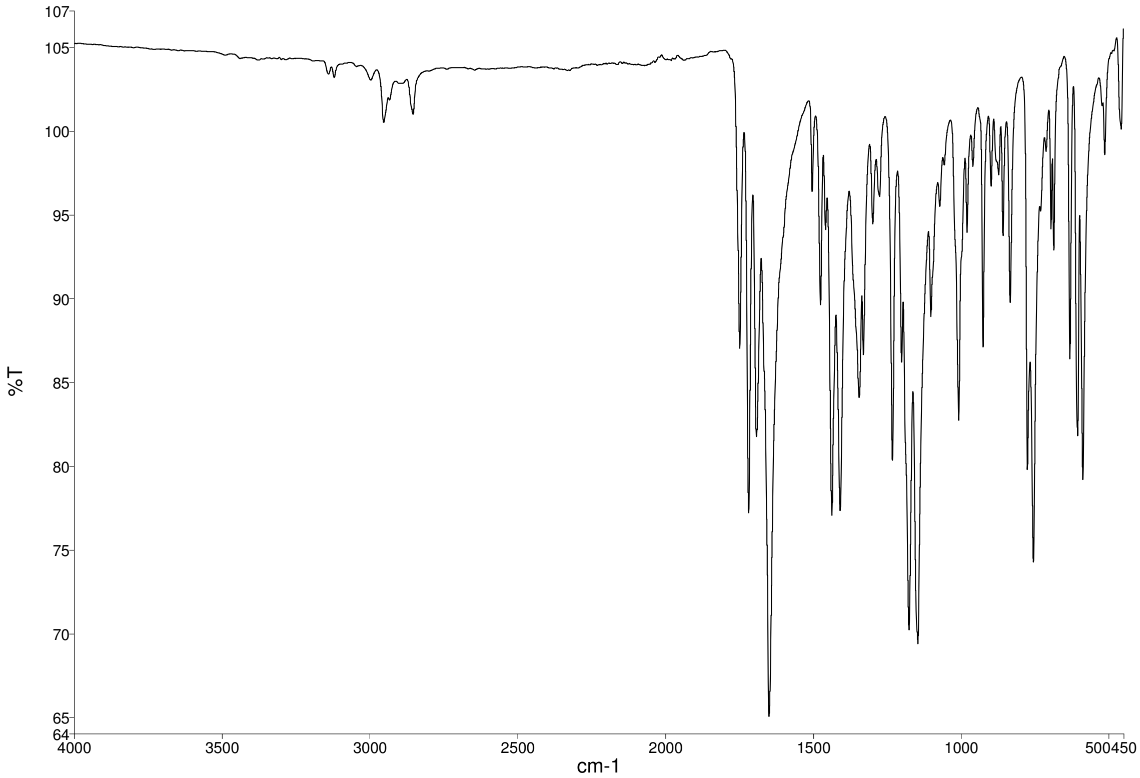

FTIR-ATR spectrum of **10d**.

S4. HRMS intrumentation and spectra

Method A:

Sample preparation

Samples were dissolved in 1 cm^3 of methanol and additionally diluted with the solvent mixture MeOH:H_2O = 50:50.

Instrumentation for HPLC/HRMS measurements:

HPLC:

Agilent 1290 Infinity II equipped with Zorbax Eclipse Plus C18 column, 3.0 × 50 mm, 1.8 µm

T (column): 40°C; Injection volume: 1 µL, Mobile phase: 0.1% HCOOH in water (A) and 0.1% HCOOH in methanol (B); flow: 0.2 mL min^{-1} and gradient as give in the table:

t / min	% A
0	95
9	5
13	5
13.1	95
14	95

HRMS:

Agilent 6550 Series Accurate-Mass-Quadrupole Time-of-Flight (Q-TOF)

Spectra were recorded in positive mode in the range of *m/z* 100 – 1000

Parameters: Temp. of sheath gas (nitrogen): 350°C; gas flow: 11 L min^{-1}; nebulizer pressure: 35 psi; VCap: 3500V; nozzle voltage: 1000 V; drying gas (nitrogen) temperature: 200°C and flow: 14 L min^{-1}

Method B:

HRMS:

4800 MALDI TOF/TOF Analyzer, Applied Biosystems

Spectra were recorded in positive mode using MALDI technology where small amount of sample was mixed with the CHCA matrix and dripped on a tile. MS/MS collision energy 1 and 2 keV, laser: Nd/Yag, 355nm, 3-7 ns pulse, 200 Hz frequency.

Precursors

3a·HPF_6: $C_8H_{14}N_3O$; Calculated: m/z([**3a**H^+]) = 168.1131

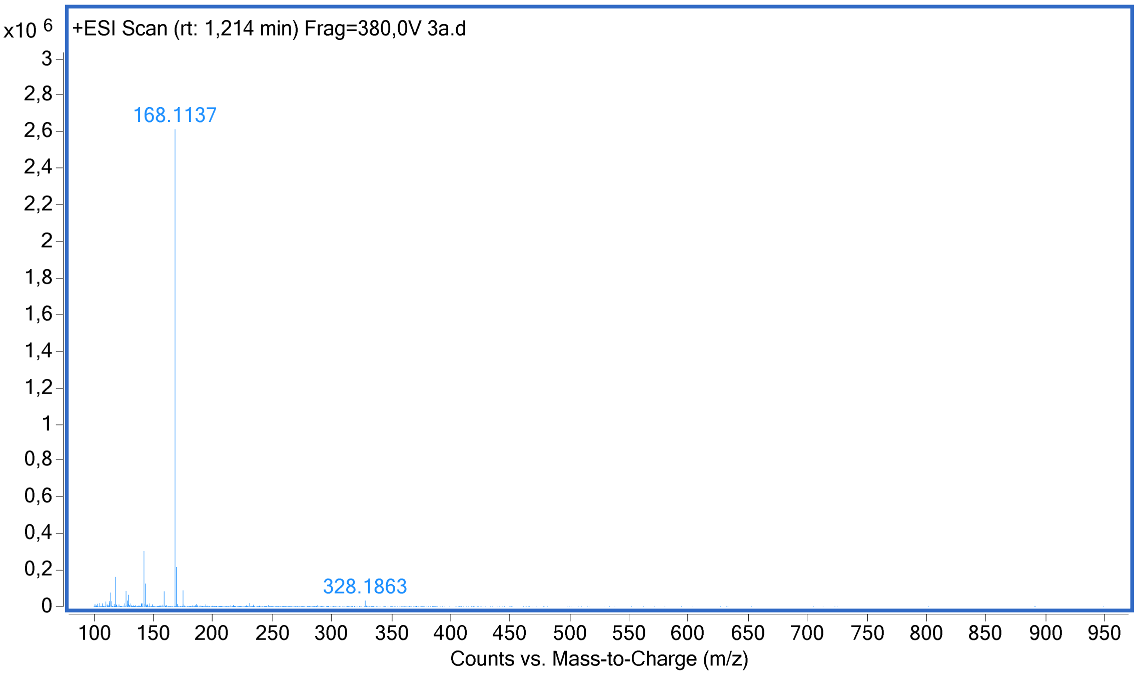

3b·HPF_6: $C_{12}H_{23}N_3O$; Calculated: m/z([**3b**H^+]) = 224.1757

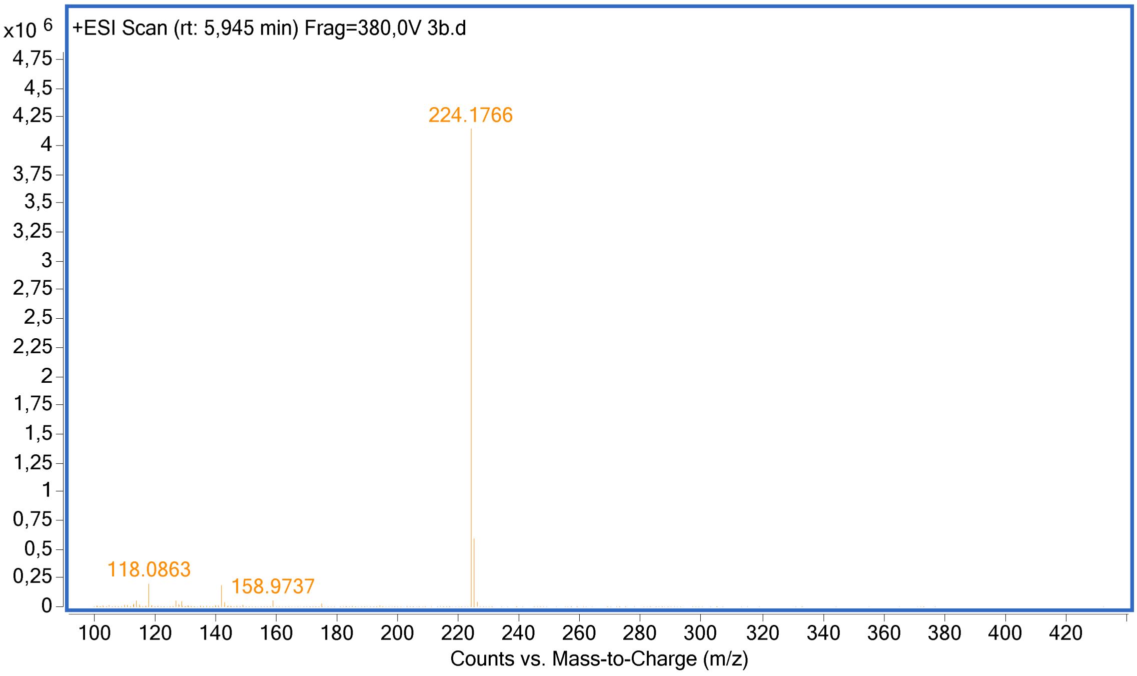

3d·HPF_6: $C_9H_{14}N_3O$; Calculated: m/z([**3d**H^+]) = 180.1137

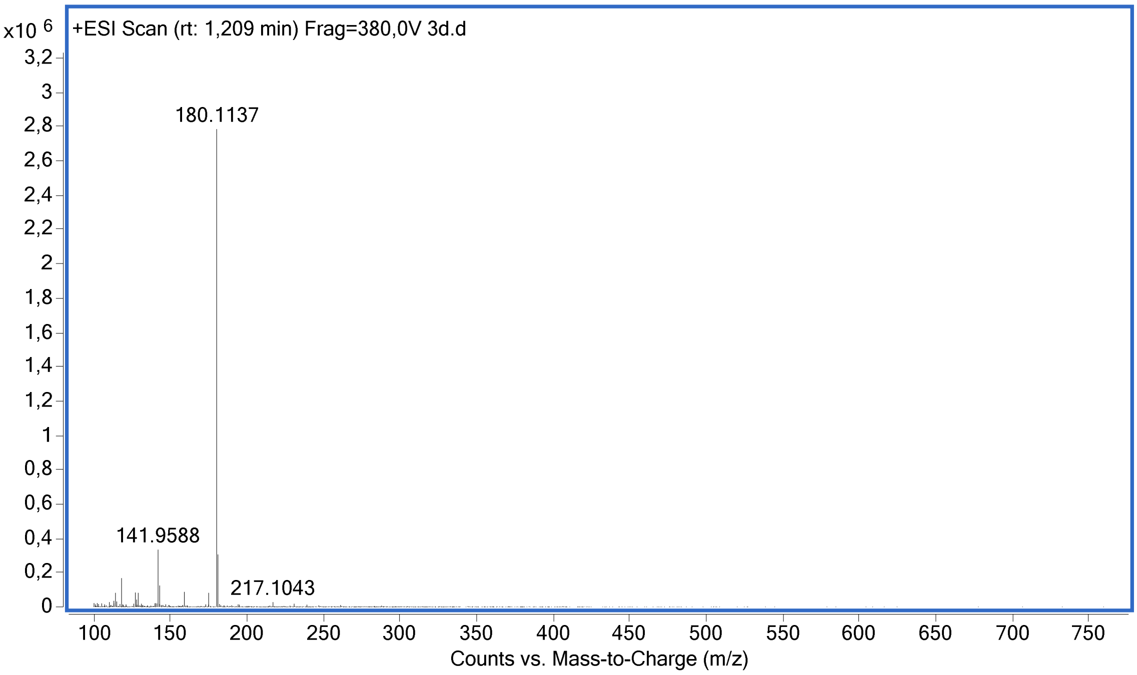

3e·HPF_6: $C_{15}H_{20}N_3O_2$; Calculated: m/z([**3e**H^+]) = 258.1601

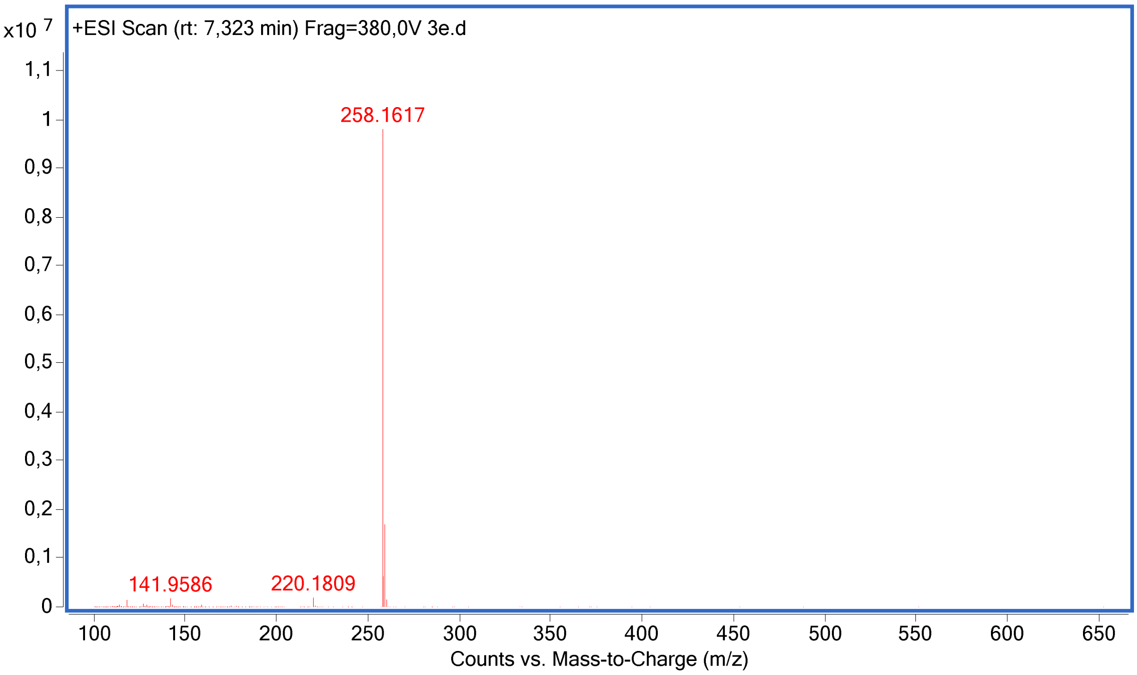

3f·HPF_6: $C_{13}H_{18}N_3O$; Calculated: m/z([**3f**H^+]) = 248.1399

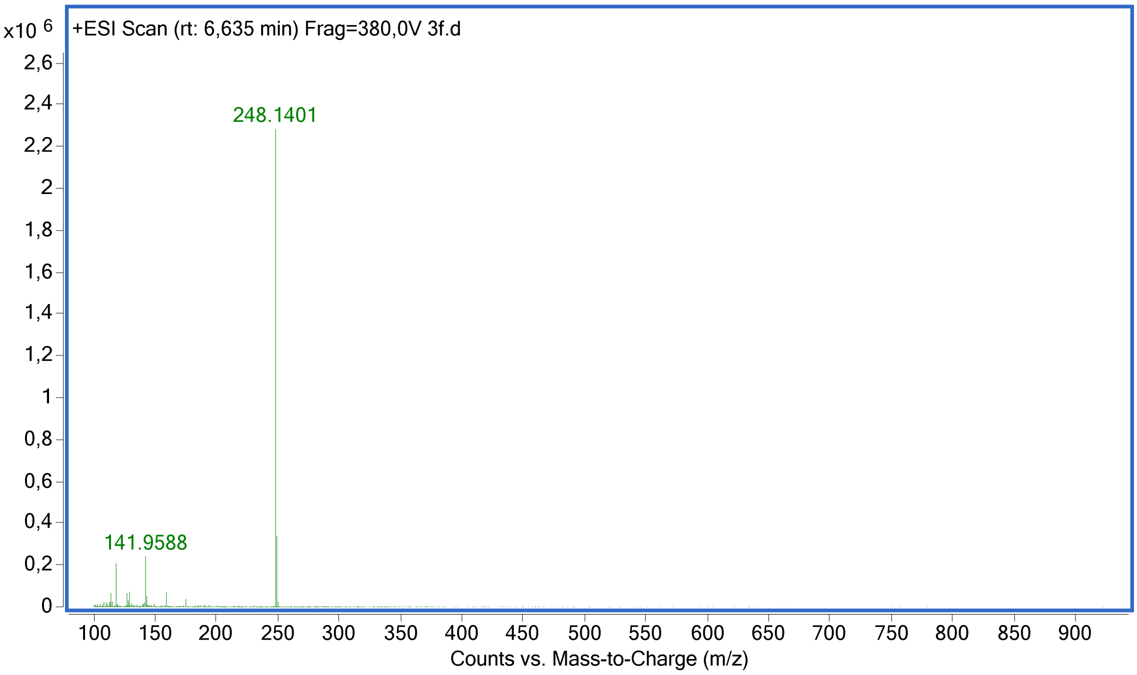

Cycloaducts

5a·HPF_6: $C_{14}H_{20}N_3O_5$; Calculated: m/z([**5a**H^+]) = 310.1397

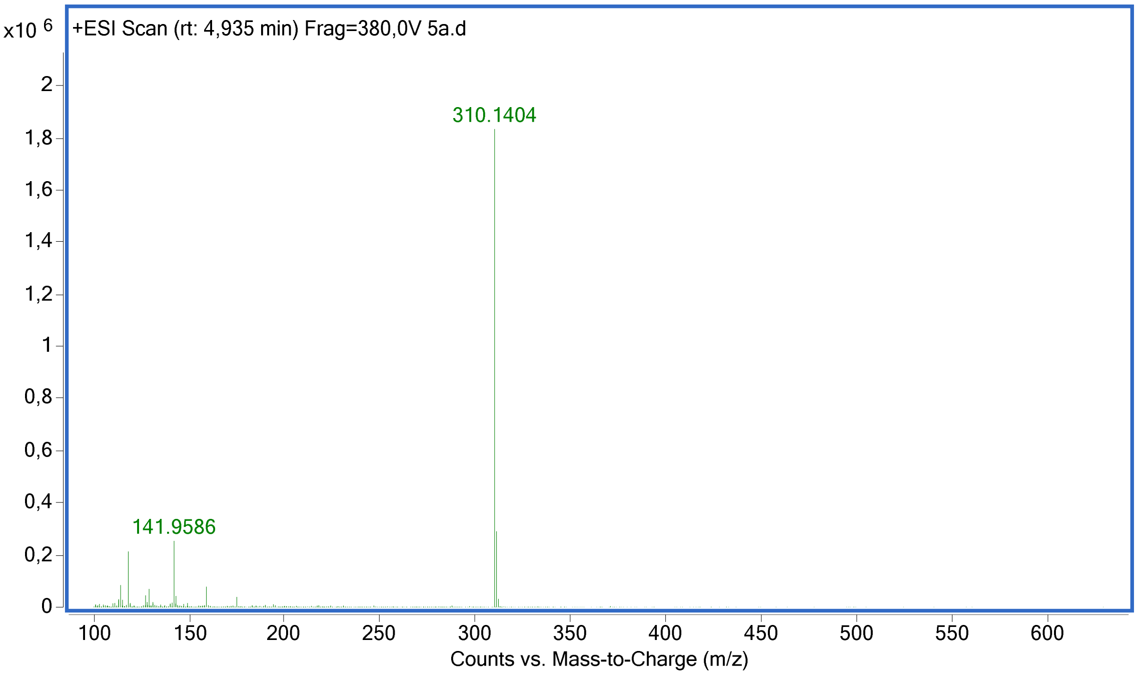

4b·HPF_6: $C_{18}H_{28}N_3O_5$; Calculated: m/z([**4b**H^+]) = 366.2023

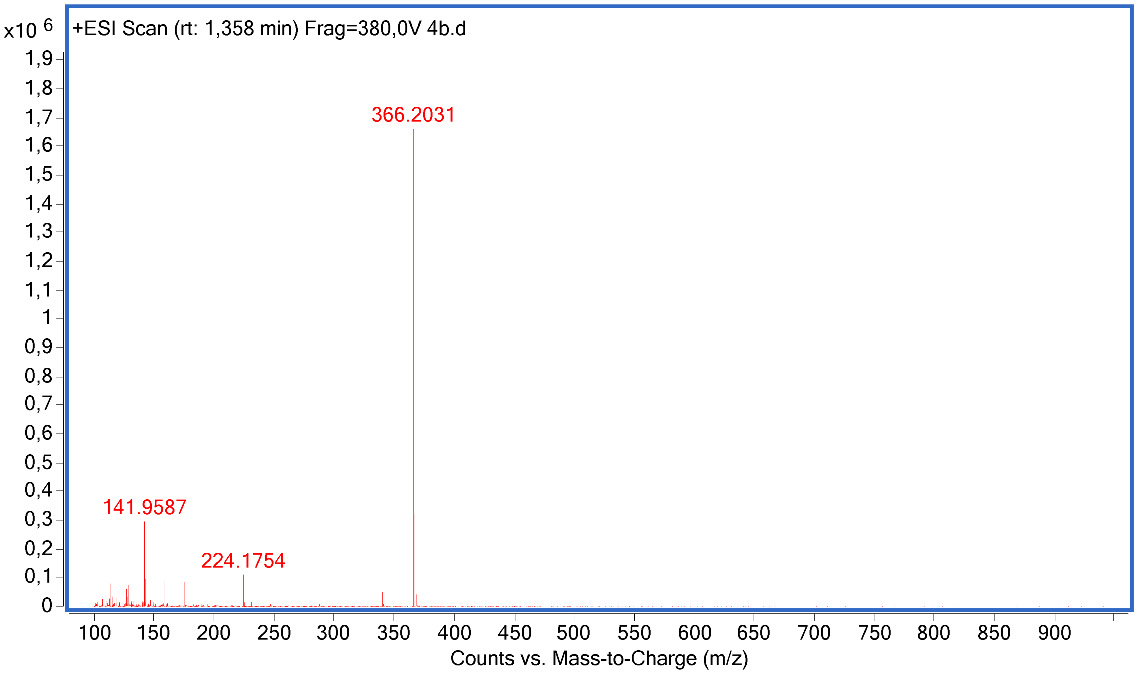

6b·HPF_6: $C_{18}H_{32}N_3O_5$; Calculated: m/z([**6b**H^+]) = 370.2336

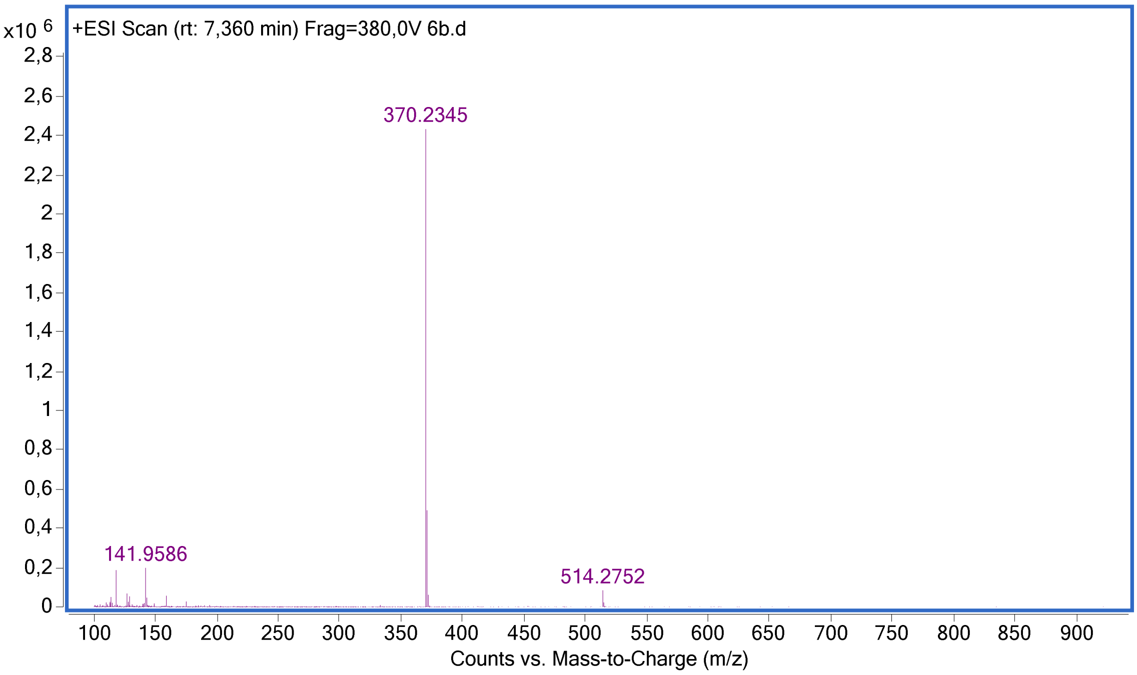

5c·HPF$_6$: $C_{14}H_{18}F_6N_3O_5P$; Calculated: m/z([**5c**·HPF$_6^+$]) = 453.0888; (**Method B**)

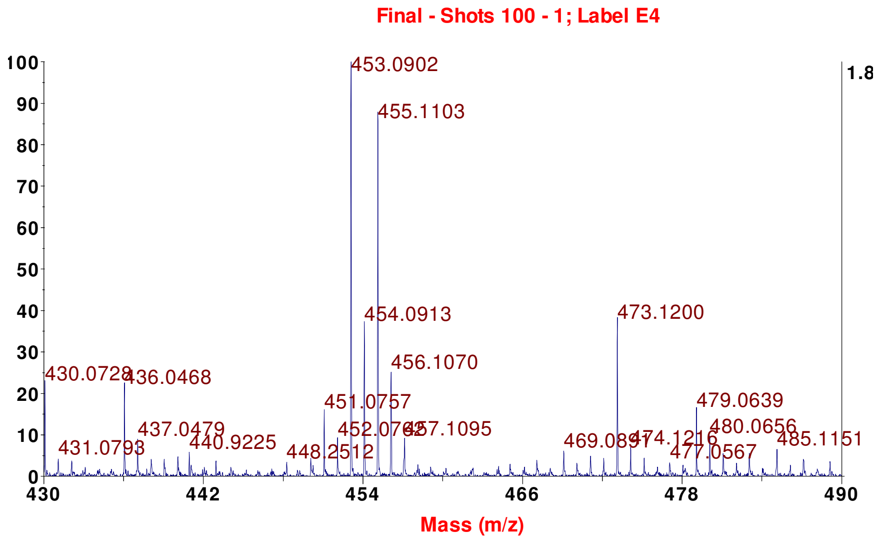

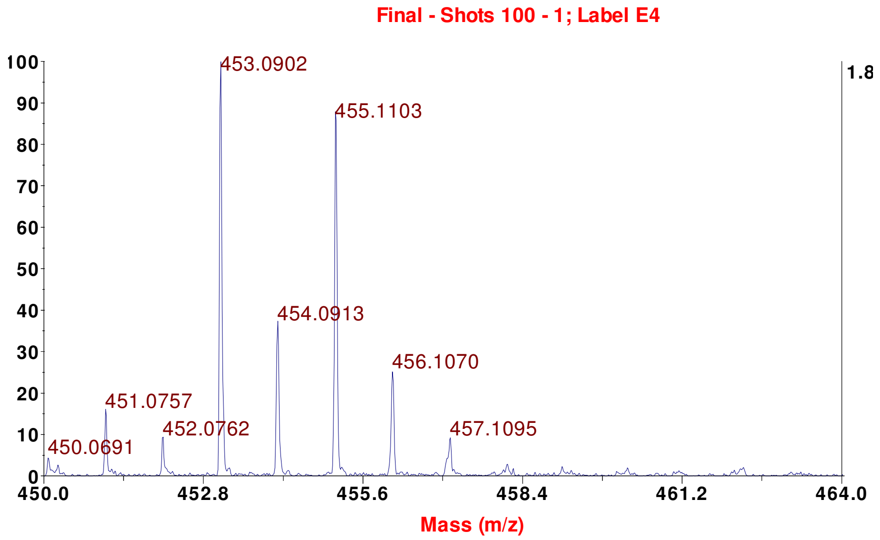

5d·HPF$_6$: $C_{15}H_{20}N_3O_5$; Calculated: m/z([**5d**H$^+$]) = 322.1403

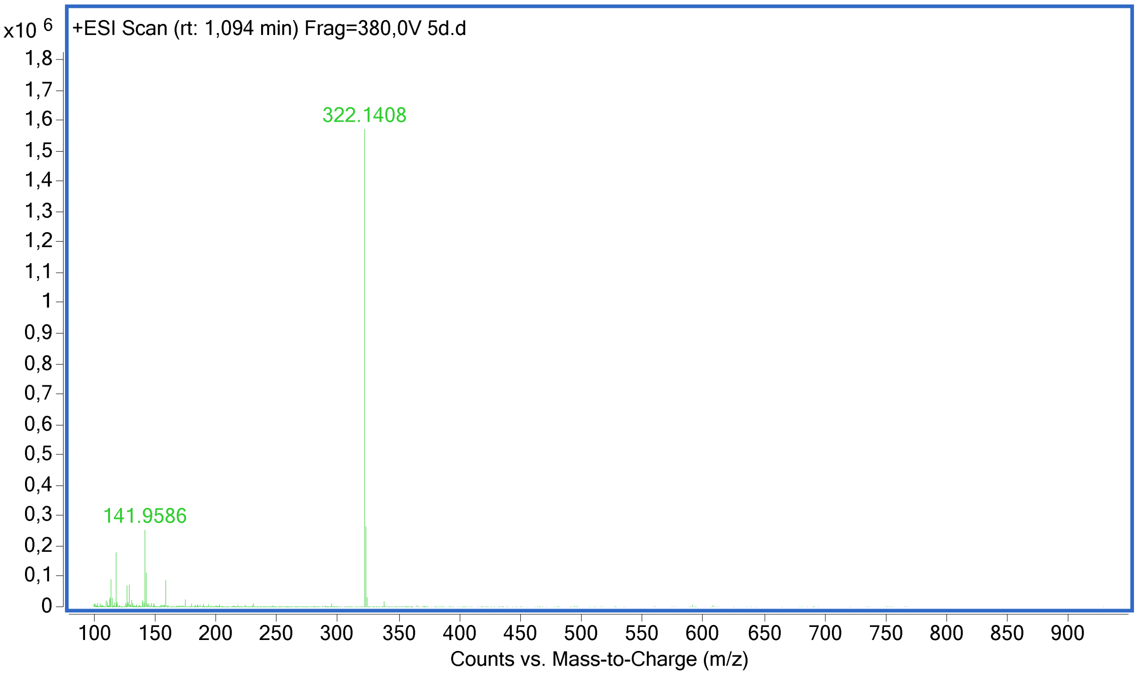

x10 6
+ESI Scan (rt: 1,094 min) Frag=380,0V 5d.d
1,8
1,7
1,6
1,5
1,4
1,3
1,2
1,1
1
0,9
0,8
0,7
0,6
0,5
0,4
0,3
0,2
0,1
0
322.1408
141.9586
100 150 200 250 300 350 400 450 500 550 600 650 700 750 800 850 900
Counts vs. Mass-to-Charge (m/z)

7d·HPF_6: $C_{15}H_{22}F_6N_3O_5P$; Calculated: m/z([**7d**·HPF_6^+]) = 469.1201; (**Method B**)

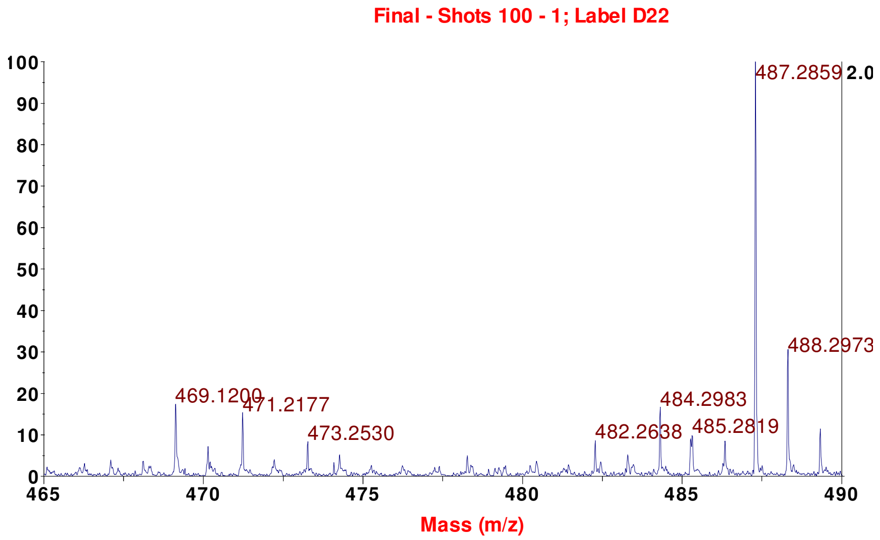

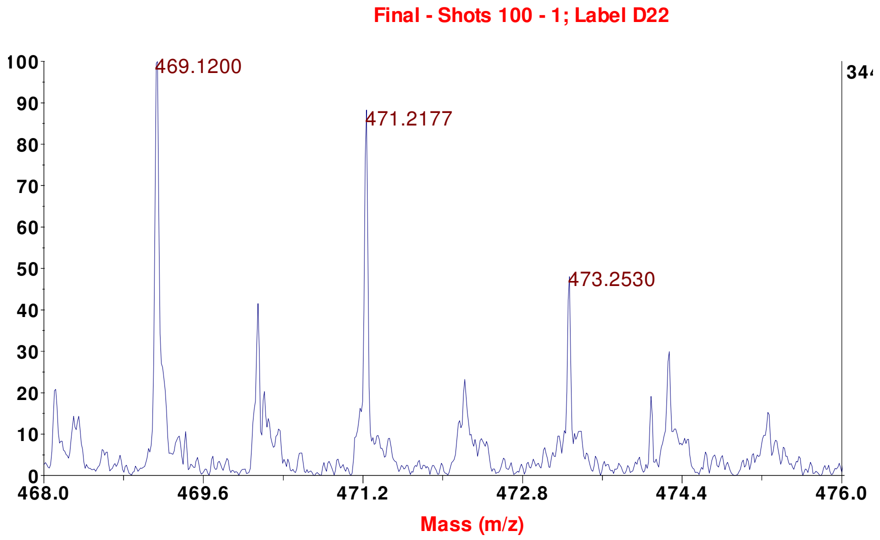

5e·HPF_6: $C_{21}H_{26}N_3O_5$; Calculated: m/z([**5e**H^+]) = 400.1867

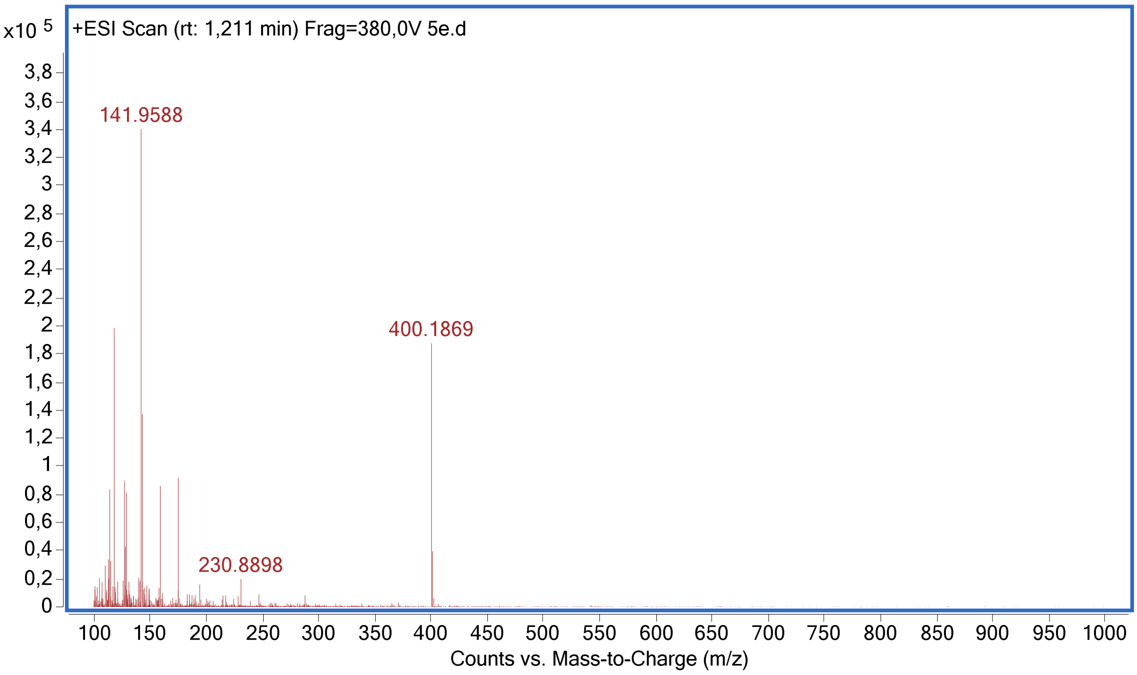

6e·HPF_6: $C_{21}H_{30}N_3O_5$; Calculated: m/z([**6e**H^+]) = 404.2185

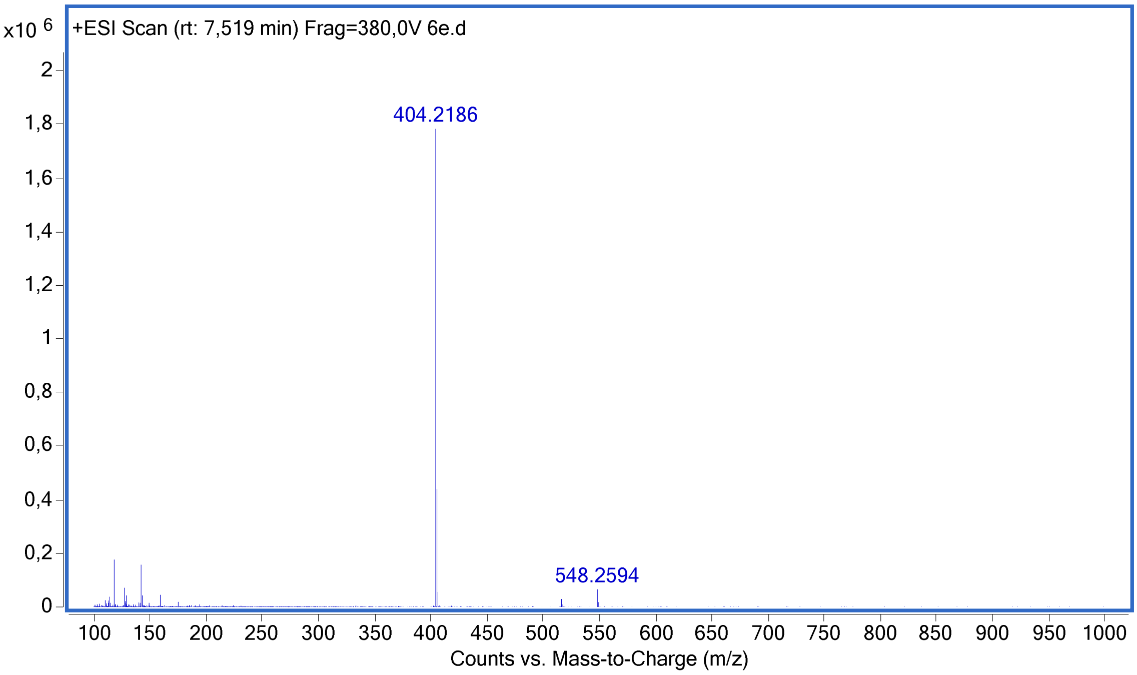

5f·HPF_6: $C_{19}H_{24}N_3O_6$; Calculated: m/z([**5f**H^+]) = 390.1665

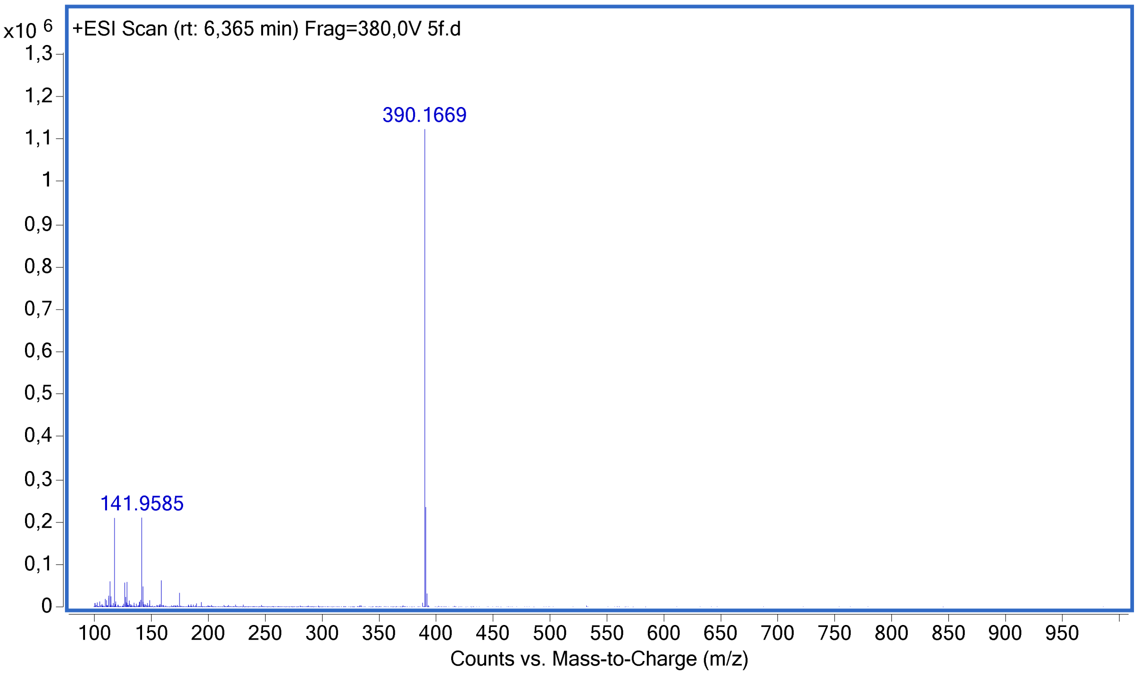

Z–**9b**: $C_{17}H_{24}N_3O_4$; Calculated: m/z([*Z*–**9b**H$^+$]) = 334.1761

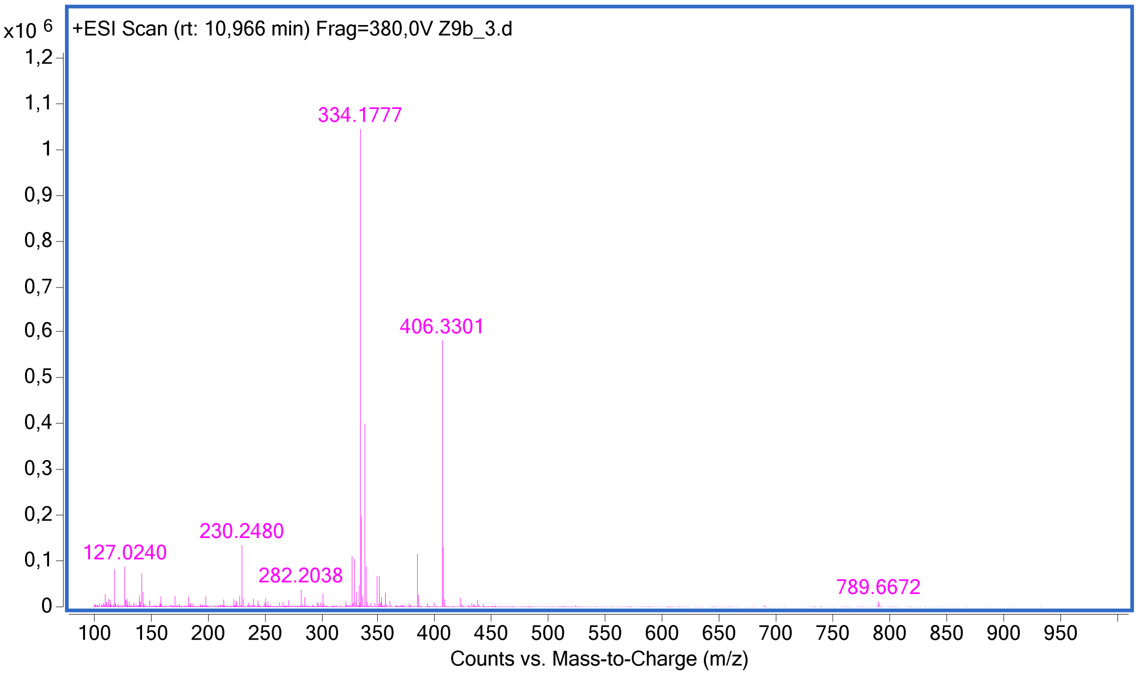

E–**9b**: $C_{17}H_{24}N_3O_4$; Calculated: m/z([*Z*–**9b**H$^+$]) = 334.1761

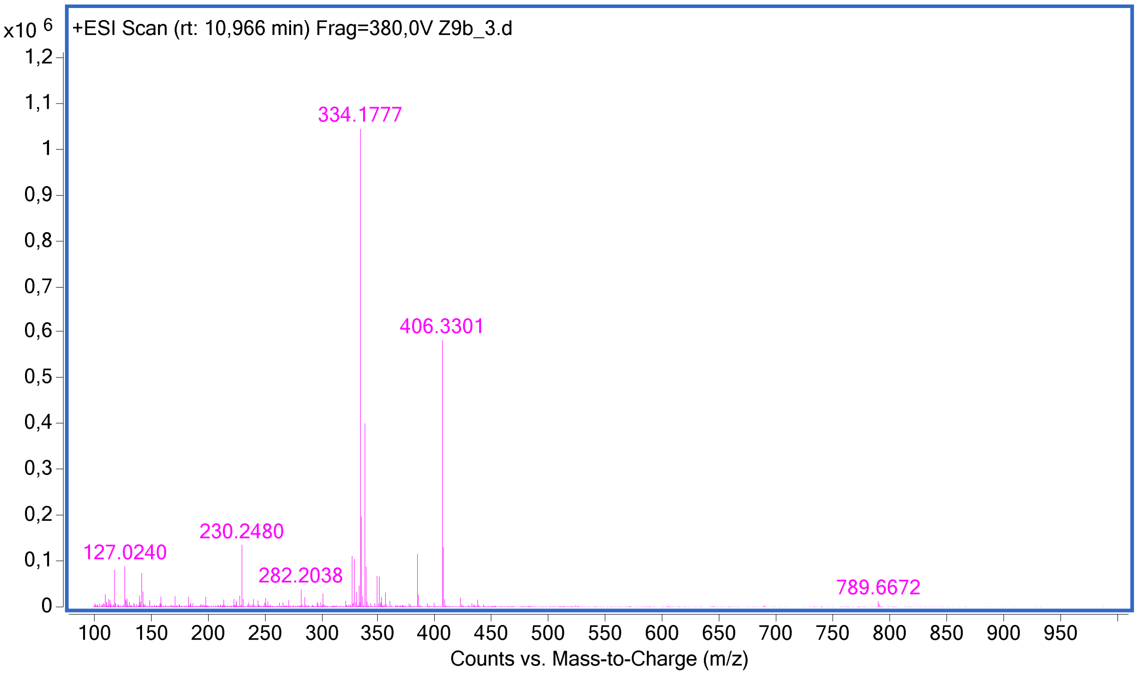

10c: $C_{13}H_{14}N_3O_4$; Calculated: m/z([**10c**H$^+$]) = 276.0979

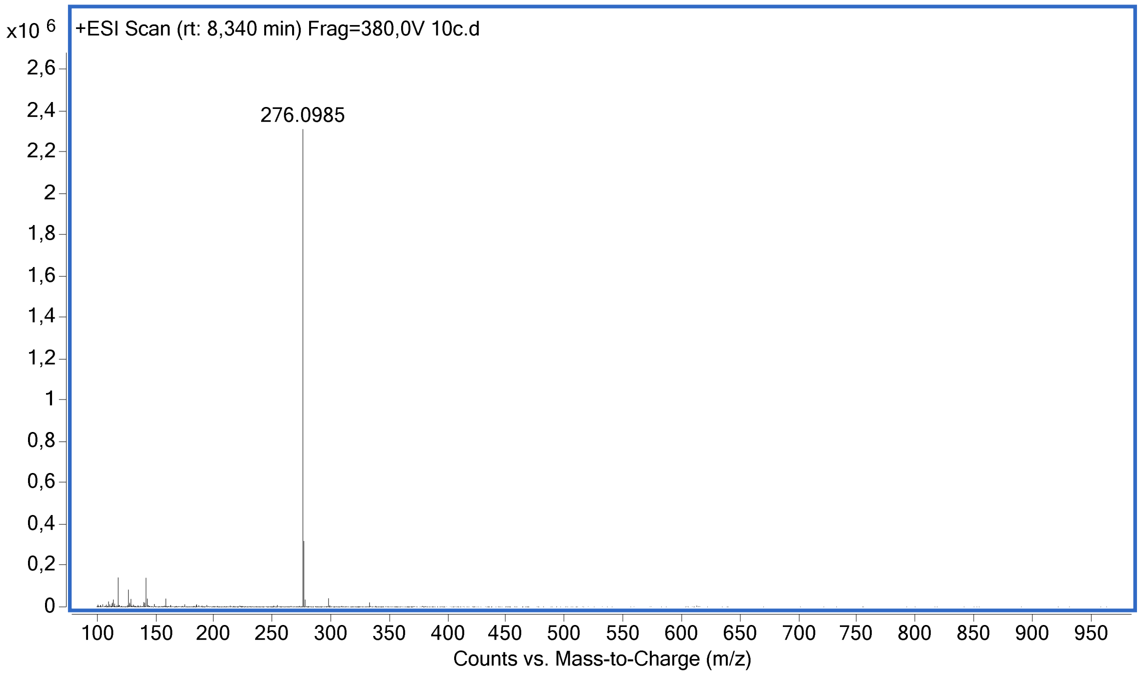

10d: $C_{14}H_{16}N_3O_4$; Calculated: m/z([**10d**H$^+$]) = 290.1135

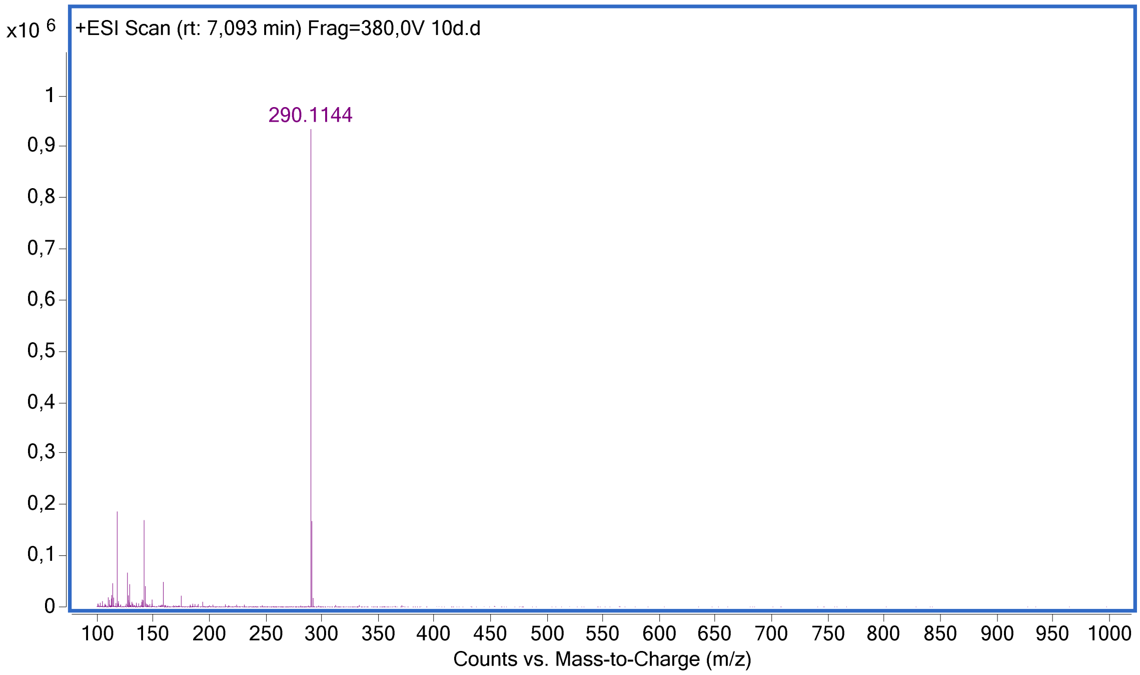

S5. pK_a calculations

Computational details: All structures were fully optimized and the nature of the stationary points was confirmed by vibrational analysis (NImag = 0). Calculations were performed using Gaussian 09 [3] (pK_a calculations) or Gaussian16 [4] (thermodynamics of the reactions) program packages. pK_a calculations were conducted using the computational scheme developed earlier and improved recently [5,6]. Thermochemistry of the reactions was calculated using B3LYP/6-31G(d,p) level of theory (M1). The presence of solvent (acetonitrile) was treated implicitly by employing the SMD continuum solvation approach [7]. The electronic energies were further corrected using M06-2X/6-311++G(3df,2pd) (M2) single-point calculations.

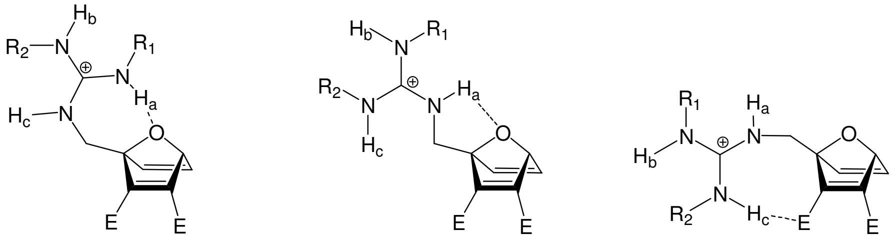

Type (IMHB): **I** **II** **III**

Figure S15. Possible configurations of the protonated oxanorbornadienes **4a**H$^+$ – **4f**H$^+$.

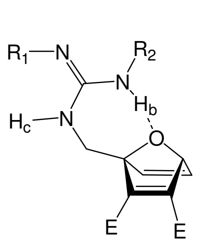

4xIa

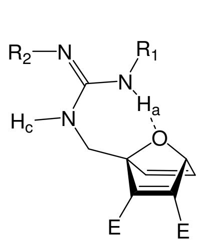

4xIb

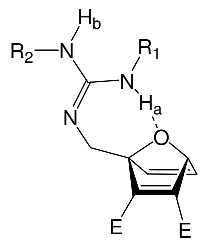

4xIc

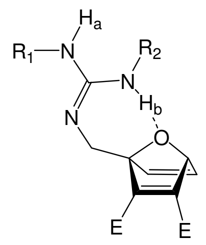

4xId

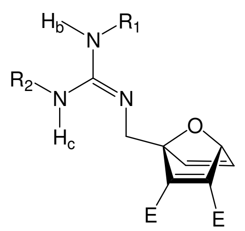

4xIIa

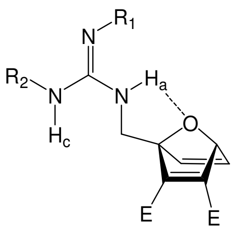

4xIIb

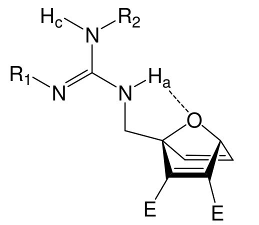

4xIIc

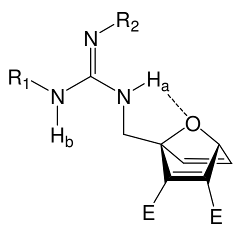

4xIIb'

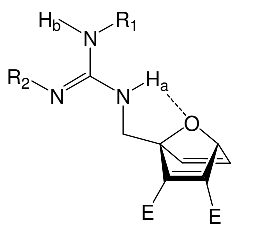

4xIIc'

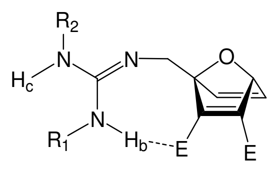

4xIIIa

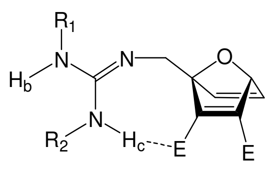

4xIIIa'

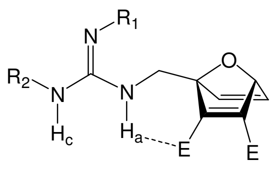

4xIIIb

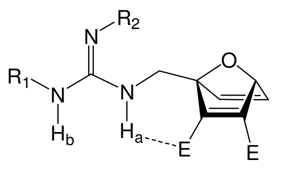

4xIIIb'

Figure S16. Possible configurations of the neutral oxanorbornadienes **4a** – **4f**.

Table S2. Calculated data for chemically non-equivalent isomers of guanidine substituted oxanorbornadienes **4(a-f)** and for the lowest energy conformer of their conjugated acid **4(a-f)**H^+.[a]

Dimethylguanidine derivative **4a** ($R_1 = R_2 =$ Me)

Isomer		E_{scf}	G_{corr}	G_{tot}	GB	$\Delta(\Delta G_{sol})$	$^{ACN}pK_a$
**4aIa**		-1085.58659	0.27797	-1085.30862	252.0	-29.7	23.6
**4aIb**		same as 4aIa					
**4aIc**		-1085.58922	0.27886	-1085.31036	250.9	-30.3	23.3
**4aId**		same as 4aIc					
**4aIIa**	**N(CH$_2$)**	**-1085.58506**	**0.27658**	**-1085.30847**	**252.1**	**-27.8**	**22.6**
**4aIIb**		-1085.58694	0.27826	-1085.30869	252.0	-30.7	24.1
**4aIIc**	**N(Me)**	**-1085.58625**	**0.27705**	**-1085.30920**	**251.7**	**-29.0**	**<u>23.0</u>**
**4aIIb'**		same as 4aIIb					
**4aIIc'**		same as 4aIIc					
**4aIIIa**		-1085.58298	0.27812	-1085.30487	254.4	-28.9	24.4
**4aIIIb**		same as 4aIIIa					
**4aIIIc**		-1085.58194	0.27655	-1085.30539	254.1	-28.8	24.2
**4aIIId**		same as 4aIIIc					
**4a**H^+		-1086.01376	0.29350	-1085.72027			

Diisopropylguanidine derivative **4b** ($R_1 = R_2 =$ *i*Pr)

Isomer		E_{scf}	G_{corr}	G_{tot}	GB	$\Delta(\Delta G_{sol})$	$^{ACN}pK_a$
**4bIa**		-1242.89792	0.38370	-1242.51422	254.9	-29.3	24.9
**4bIb**		same as 4bIa					
**4bIc**		-1242.90021	0.38498	-1242.51524	254.3	-27.9	23.8
**4bId**		same as 4bIc					
**4bIIa**	**N(CH$_2$)**	**-1242.89749**	**0.38244**	**-1242.51505**	**254.4**	**-26.8**	**23.3**
**4bIIb**		-1242.89725	0.38451	-1242.51274	255.8	-28.9	25.2
**4bIIc**	**N(*i*Pr)**	**-1242.89857**	**0.38382**	**-1242.51475**	**254.6**	**-27.9**	**24.0**
**4bIIb'**		same as 4bIIb					
**4bIIc'**		same as 4bIIc					
**4bIIIa**		-1242.89335	0.38451	-1242.50883	258.3	-27.1	25.6
**4bIIIb**		same as 4bIIIa					
**4bIIIc**		-1242.89223	0.38326	-1242.50896	258.2	-27.3	25.7
**4bIIId**		same as 4bIIIc					
**4b**H^+		-1243.32873	0.39828	-1242.93045			

a) Electronic energies (E_{scf}), corrections to Gibbs energy (G_{corr}), and total Gibbs energies (G_{tot}) are given in a.u. while the gas-phase basicities (GBs) and the difference in solvation energies ($\Delta(\Delta G_{sol})$) are given in kcal mol^{-1}.

Table S2.(continued)

2-Aminoimidazole derivative **4c** (R_1 = R_2 = $-CH_2CH_2-$)

Isomer		E_{scf}	G_{corr}	G_{tot}	GB	$\Delta(\Delta G_{sol})$	$^{ACN}pK_a$
**4cIa**		-1084.37820	0.25891	-1084.11929	249.8	-30.3	22.7
**4cIb**		same as 4cIa					
**4cIc**		-1084.38401	0.26038	-1084.12363	247.1	-30.7	21.5
**4cId**		same as 4cIc					
**4cIIa**	**N(CH$_2$)**	**-1084.37949**	**0.25757**	**-1084.12192**	**248.1**	**-28.0**	**20.5**
**4cIIb**		-1084.37834	0.25907	-1084.11927	249.8	-29.2	22.1
**4cIIc**	**N(cyc)**	**-1084.38232**	**0.25905**	**-1084.12327**	**247.3**	**-30.3**	**21.4**
**4cIIb'**		same as 4cIIb					
**4cIIc'**		same as 4cIIc					
**4cIIIa**		-1084.37951	0.26088	-1084.11863	250.2	-29.7	22.6
**4cIIIb**		same as 4cIIIa					
**4cIIIc**		-1084.37866	0.25940	-1084.11926	249.8	-30.12	22.6
**4cIIId**		same as 4cIIIc					
**4c**H$^+$		-1084.80275	0.27538	-1084.52737			

2-Aminopyrimidine derivative **4d** (R_1 = R_2 = $-CH_2CH_2CH_2-$)

Isomer		E_{scf}	G_{corr}	G_{tot}	GB	$\Delta(\Delta G_{sol})$	$^{ACN}pK_a$
**4dIa**	**N(cyc)**	**-1123.71025**	**0.28787**	**-1123.42238**	**251.8**	**-29.8**	**23.5**
**4dIb**		same as 4dIa					
**4dIc**		-1123.70930	0.28751	-1123.42180	252.2	-29.5	23.6
**4dId**		same as 4dIc					
**4dIIa**	**N(CH$_2$)**	**-1123.70745**	**0.28595**	**-1123.42150**	**252.4**	**-27.1**	**22.3**
**4dIIb**		-1123.71021	0.28867	-1123.42154	252.3	-29.9	23.9
**4dIIc**		-1123.70723	0.28808	-1123.41915	253.8	-30.1	24.8
**4dIIb'**		same as 4dIIb					
**4dIIc'**		same as 4dIIc					
**4dIIIa**		-1123.70537	0.28840	-1123.41697	255.2	-28.5	24.7
**4dIIIb**		same as 4dIIIa					
**4dIIIc**		-1123.70654	0.28734	-1123.41920	253.8	-29.3	24.4
**4dIIId**		same as 4dIIIc					
**4d**H$^+$		-1124.13733	0.303651	-1123.83368			

a) Electronic energies (E_{scf}), corrections to Gibbs energy (G_{corr}), and total Gibbs energies (G_{tot}) are given in a.u. while the gas-phase basicities (GBs) and the difference in solvation energies ($\Delta(\Delta G_{sol})$) are given in kcal mol^{-1}.

Table S2.(continued)

N^1–Phenyl–N^3–propylguanidine derivative **4e** (R_1 = Ph, R_2 = *n*Pr)

Isomer		E_{scf}	G_{corr}	G_{tot}	GB	$\Delta(\Delta G_{sol})$	$^{ACN}pK_a$
**4eIa**		-1356.04596	0.37817	-1355.66779	249.4	-26.2	20.3
**4eIb**		-1356.03709	0.37723	-1355.65987	254.4	-26.6	23.2
**4eIc**		-1356.04300	0.37852	-1355.66448	251.5	-26.8	21.7
**4eId**		-1356.03930	0.37865	-1355.66064	253.9	-26.1	22.6
**4eIIa**	**N(CH₂)**	**-1356.03780**	**0.37718**	**-1355.66062**	**253.9**	**-24.2**	**21.6**
**4eIIb**		-1356.04548	0.37852	-1355.66696	249.9	-26.9	20.9
**4eIIc**	**N(Ph)**	**-1356.04504**	**0.37610**	**-1355.66893**	**248.7**	**-25.6**	**19.6**
**4eIIb'**		-1356.03905	0.37914	-1355.65992	254.3	-27.0	23.4
**4eIIc'**	**N(Pr)**	**-1356.03860**	**0.37564**	**-1355.66296**	**252.4**	**-26.0**	**21.8**
**4eIIIa**		-1356.03457	0.37817	-1355.65640	256.5	-25.5	23.8
**4eIIIb**		-1356.03848	0.37872	-1355.65977	254.4	-25.9	22.9
**4eIIIc**		-1356.04281	0.37762	-1355.66519	251.0	-25.8	20.9
**4eIIId**		-1356.03476	0.37704	-1355.65772	255.7	-26.4	23.8
**4e**H^+		-1356.46836	0.39312	-1356.07524			

N^1–Furfuryl–N^3–ethylguanidine derivative **4f** (R_1 = Et, R_2 = Furfuryl)

Isomer		E_{scf}	G_{corr}	G_{tot}	GB	$\Delta(\Delta G_{sol})$	$^{ACN}pK_a$
**4fIa**	**N(Et)**	**-1353.81655**	**0.34858**	**-1353.46798**	**253.9**	**-27.4**	**23.4**
**4fIb**		-1353.81515	0.34860	-1353.46656	254.7	-26.3	23.2
**4fIc**		-1353.81940	0.34933	-1353.47007	252.5	-26.8	22.3
**4fId**		-1353.81714	0.34907	-1353.46807	253.8	-26.5	22.8
**4fIIa**	**N(CH₂)**	**-1353.81585**	**0.34751**	**-1353.46834**	**253.6**	**-24.8**	**21.8**
**4fIIb**		-1353.81569	0.35010	-1353.46558	255.4	-27.1	24.0
**4fIIc**		-1353.81501	0.35029	-1353.46472	255.9	-26.5	24.0
**4fIIb'**		-1353.81622	0.34831	-1353.46791	253.9	-26.1	22.7
**4fIIc'**	**N(Fu)**	**-1353.81694**	**0.34777**	**-1353.46917**	**253.1**	**-26.8**	**22.6**
**4fIIIa**		-1353.81434	0.34886	-1353.46548	255.4	-25.4	23.1
**4fIIIa'**		-1353.81471	0.34997	-1353.46474	255.9	-25.9	23.6
**4fIIIb**		-1353.81403	0.34709	-1353.46694	254.5	-26.7	23.3
**4fIIIb'**		-1353.81204	0.34664	-1353.46540	255.5	-25.9	23.4
**4f**H^+		-1354.24601	0.36348	-1353.88253			

a) Electronic energies (E_{scf}), corrections to Gibbs energy (G_{corr}), and total Gibbs energies (G_{tot}) are given in a.u. while the gas-phase basicities (GBs) and the difference in solvation energies ($\Delta(\Delta G_{sol})$) are given in kcal mol^{-1}.

S6. Cartesian coordinates

a). Optimized geometries used in pK_a calculations

4alla

C	2.9834375172	-1.2307702386	-0.8629165303
C	2.097699095	0.0151824174	-0.6371842535
O	0.8249820935	-0.4800371261	-1.0801337272
C	0.8153931175	-1.6398744409	-0.2277956604
C	2.1863010379	-2.2639840314	-0.5948266436
C	1.8313732615	0.1102515994	0.8891033986
C	1.0130478569	-0.9219619321	1.1523985368
C	0.5036938439	-1.5216679396	2.4024534187
O	-0.1074214475	-0.6468031419	3.216602436
C	-0.6037566073	-1.2057900568	4.4420914114
C	2.5427863644	1.0899767484	1.7216546792
O	2.3516924111	0.9184327712	3.0471257221
C	-0.4294420044	-2.4901534869	-0.3859651209
N	-1.5695235036	-1.8071925797	0.1830175974
C	-2.7500733492	-2.2632664391	-0.0547999876
N	-3.8630131967	-1.5252960951	0.3283758882
C	-3.6451929481	-0.2817904916	1.0566852345
N	-3.0326216775	-3.4283114013	-0.7556824654
O	0.6271776186	-2.7091723734	2.6419822593
O	3.2558626461	1.9610396574	1.2536105065
H	-4.617121141	0.1891074336	1.2314823616
H	-3.0262738607	0.3820707416	0.450337273
H	-0.2266026987	-3.4505228077	0.1200409349
H	-0.5495681178	-2.7078702674	-1.4604935864
H	2.3766905176	0.9440784282	-1.130115225
H	-1.0585711759	-0.3721516285	4.9780820617
H	0.2121412223	-1.6395437918	5.0265423638
H	-1.3456782246	-1.9822150556	4.2360640284
H	2.4141810632	-3.3219421203	-0.552518245
H	4.0395628155	-1.2152951587	-1.1018673408
C	3.0294483551	1.8642342524	3.8870001963
H	2.7652929886	1.587671858	4.9082390691
H	4.1115315295	1.8052602032	3.740414909
H	2.6979109789	2.8826526802	3.666699619
C	-4.3705523628	-3.9230978285	-1.0204794937
H	-3.1269162781	-0.4274425572	2.0140942103
H	-4.3087255874	-4.7309065885	-1.755143915
H	-4.8873059063	-4.318620994	-0.1293977963
H	-4.9805722043	-3.1222921849	-1.4479121038
H	-2.2989193426	-4.1208522392	-0.7322942412
H	-4.6382716997	-2.0792197941	0.6706602205

4aIIc

C	3.0145930495	-1.097128434	-0.9327250412
C	2.1243280488	0.1252903109	-0.6184414043
O	0.8537246314	-0.3462543618	-1.1060707435
C	0.8532883444	-1.5724348741	-0.3389861732
C	2.2255904886	-2.1547959262	-0.7474881952
C	1.8437471706	0.1078644865	0.9063240589
C	1.0350826397	-0.9481341718	1.0907458961
C	0.5112331217	-1.6254224774	2.2964772913
O	-0.1886408252	-0.8146520316	3.1063737078
C	-0.7264205815	-1.4493959471	4.2803391751
C	2.5284076287	1.0348203153	1.8194125755
O	2.3105338225	0.7647813356	3.1229344026
C	-0.3952998236	-2.3870130222	-0.5814916664
N	-1.54319345	-1.6042630717	-0.1493445836
C	-2.8044616584	-2.1999323112	-0.1815475087
N	-3.8318186532	-1.3817297351	0.2733547194
C	-3.6603955615	0.0116953184	0.6449168577
N	-2.9240556655	-3.4305533897	-0.5341701909
O	0.6832022169	-2.8132387299	2.4908283517
O	3.2372351811	1.9449466904	1.4267910348
H	-4.7504123638	-1.6460121698	-0.0512214377
H	-4.5665476757	0.3549324902	1.1526229038
H	-3.4778780816	0.6847794141	-0.2097013464
H	-0.3612136555	-3.3086828511	0.0036023217
H	-0.4478649124	-2.6738361417	-1.6411142158
H	2.3998329352	1.0883814706	-1.0426281869
H	-1.2700201032	-0.6662987544	4.8092746648
H	0.0792151922	-1.8471341431	4.9028607288
H	-1.3979264121	-2.2646079049	3.9991518385
H	2.4574356078	-3.2114598735	-0.7894606171
H	4.0700492552	-1.0561142577	-1.1705176659
C	2.9628412941	1.6498289392	4.0471709473
H	2.6846786789	1.2908637327	5.0383504069
H	4.0474653105	1.6131981728	3.914671382
H	2.6220359161	2.6782873292	3.9005275035
C	-4.2264702861	-4.0496758093	-0.3895611817
H	-2.8224943876	0.1044120251	1.3417249637
H	-4.1358468421	-5.1257700634	-0.5693253727
H	-4.6626092125	-3.913055987	0.6150823151
H	-4.9661407264	-3.6702678133	-1.1189875914
H	-1.5083076564	-0.6489297779	-0.4890309246

4aH^+

C	-2.5538497999	-1.9199066655	-1.6132207414
C	-1.9318925037	-1.7132801615	-0.216689342
O	-0.5477832964	-1.4941405408	-0.5849254647
C	-0.8196537363	-0.4236073221	-1.5218854972

C	-1.867535493	-1.1184895824	-2.426557943
C	-2.2943314191	-0.2757496899	0.2465627065
C	-1.6135982905	0.5400579624	-0.5739407839
C	-1.5727521798	2.0210527249	-0.6497461101
O	-2.7606988319	2.5810652477	-0.4896596808
C	-2.7993890984	4.0281395758	-0.4581710563
C	-3.2038305227	0.0528637183	1.3715867472
O	-4.0327991991	-0.9824909493	1.6032722353
C	0.4635790621	0.0680493626	-2.1697582777
N	1.3941656682	0.6989399253	-1.2360132101
C	2.3115125636	0.0561273646	-0.4955074814
N	3.2712076207	0.7723439529	0.114059587
C	3.4854457557	2.2069573548	-0.0793774329
N	2.3131224993	-1.2849893672	-0.4172202789
O	-0.5391425317	2.6484116856	-0.8671875446
O	-3.1841169105	1.0866454519	2.0021988184
H	1.1041006596	1.6401987057	-0.9628088674
H	0.2268194295	0.8122863735	-2.934629868
H	0.962059507	-0.7707444148	-2.6637392594
H	-2.0359963323	-2.5065128881	0.5192463623
H	-3.850863674	4.2790784274	-0.3313400723
H	-2.4083727842	4.4351005738	-1.3928923269
H	-2.208606625	4.3949179122	0.3835122817
H	-2.0164818611	-0.9121557159	-3.4791300809
H	-3.4126450649	-2.5440698237	-1.8231856408
H	1.3981823298	-1.7258325268	-0.501469507
C	-4.9802204057	-0.7945210402	2.6787689024
H	-5.5591679961	-1.7161824708	2.7179261138
H	-4.4516062108	-0.6250914416	3.6195619072
H	-5.6248546596	0.0611202101	2.4660260632
H	2.7493252258	2.8099937398	0.4646375242
H	4.4824434602	2.4558556224	0.2867642428
H	3.4337894874	2.4462024299	-1.1444028561
C	3.4041497456	-2.0713669182	0.151945423
H	3.2628981953	-3.1124038212	-0.1423066319
H	4.3619490234	-1.7297052485	-0.2499900321
H	3.4282516461	-2.0255507595	1.2488684216
H	3.7815915474	0.3191070263	0.8589496516

4bIIa

C	0.8180321089	-1.0180177755	1.2451821073
C	0.6062018133	-2.3592277043	0.4612152543
O	0.5905486724	-1.8273323421	-0.8783457332
C	1.8673232224	-1.1731080591	-0.7879548774
C	1.6231790543	-0.2837778808	0.459798647
C	1.9749824502	-3.0845986445	0.4549078634
C	2.7581339777	-2.3517076236	-0.3356343392
C	-0.6405528342	-3.1598244489	0.789836947

N	-1.7738396777	-2.2701864794	0.8531478373
C	-2.9315129293	-2.6097401509	0.408083928
N	-3.3538659381	-3.8984344253	0.0601810154
C	-4.1292493079	-4.164463897	-1.1694427744
C	2.339792649	0.9868455875	0.6354155144
O	3.0473077424	1.4710442648	-0.2312911067
C	0.3332721685	-0.8627800628	2.6323840242
O	0.4858956077	-1.7381960526	3.4642408915
O	-0.2946490672	0.3002618745	2.8639324287
C	-0.7808964616	0.4676224748	4.2055786117
O	2.1593790524	1.5487469137	1.8493606064
C	2.8469188971	2.7921359071	2.0526655072
N	-3.9238514383	-1.6491438296	0.2645073588
C	-3.6547405455	-0.2433035329	0.6071973045
C	-4.9919450235	0.456547995	0.8605410305
C	-2.8600231685	0.4365846029	-0.5139246733
H	-0.4704736243	-3.6503415429	1.7595537951
H	-0.7179034427	-3.9546101951	0.0273271135
H	2.1303377617	-0.6480327181	-1.7037562146
H	-1.2715141267	1.4412642049	4.2164411897
H	0.0466718901	0.4458041729	4.9197408551
H	-1.4902800702	-0.3254065897	4.4562363153
H	2.212079386	-3.9601167503	1.0466119928
H	3.809532044	-2.4679205814	-0.5676610599
H	2.5933518425	3.1021341421	3.066886836
H	3.9271767133	2.6566870447	1.9504833473
H	2.5140292709	3.5397773625	1.3275745356
H	-3.0482791062	-0.2095725992	1.5216828534
C	-4.680425579	-5.5908465992	-1.0966996969
H	-4.9725281018	-3.4675390152	-1.1717631904
C	-3.3059117773	-3.9258853889	-2.4433771563
H	-4.8284254057	1.5090426562	1.1132457205
H	-5.6293466342	0.4203737107	-0.0320205312
H	-5.5379775789	-0.0061839418	1.6927824392
H	-2.629262911	1.4731704076	-0.2404481387
H	-1.917776693	-0.0890695458	-0.6874671035
H	-3.439102123	0.4441457114	-1.4453931716
H	-5.3017404471	-5.8101514126	-1.9716260963
H	-3.8646762762	-6.3260739927	-1.079889971
H	-5.2836401171	-5.732567274	-0.1942632175
H	-3.918783221	-4.0777963852	-3.3400287592
H	-2.9138684307	-2.904236299	-2.4640269567
H	-2.4565316081	-4.6190055419	-2.4943342537
H	-2.6238029008	-4.5875886198	0.2013379255
H	-4.8444777578	-1.9811771312	0.526551225

4bIIb'

C	-1.5154872547	-0.4485973415	-0.3451963099
C	-0.6132792591	-1.6896757092	-0.0132447619
O	-0.9306433151	-1.8173690484	1.3911174564
C	-2.3545380864	-1.9131814586	1.2089725202
C	-2.6093655117	-0.6035418204	0.4194594836
C	-1.3502134995	-2.9284733134	-0.5761297026
C	-2.4283059855	-3.0752581259	0.1928867162
C	0.8743073217	-1.5594114587	-0.2417238527
N	1.366257348	-0.4238901696	0.525032733
C	2.7089062393	-0.0650865536	0.3858724691
N	3.4110107819	-0.6147828603	-0.5394090298
C	4.7749915128	-0.1843680602	-0.806427639
C	-3.9077146203	0.0836631409	0.4941886046
O	-4.7844022995	-0.2499864067	1.2720693038
C	-1.1953974177	0.4508158918	-1.4741343054
O	-0.8539249237	0.0151604129	-2.5573050276
O	-1.2875189831	1.756336959	-1.1757406606
C	-1.0097055914	2.6505546231	-2.2688019611
O	-4.0364195418	1.095489419	-0.3879721146
C	-5.2930637181	1.7898189958	-0.3404111304
N	3.1412917193	0.8702978303	1.3293123521
C	2.2914501154	1.9459540747	1.8718707556
H	4.0866490135	1.1841127474	1.1461107761
C	1.8107143904	2.9330957207	0.798352988
C	3.0714191462	2.6465560241	2.9882215923
H	1.0760468501	-1.3869806502	-1.3006686084
H	1.3714614676	-2.5005078768	0.0346738577
H	-2.8876922892	-2.0162225073	2.1514942049
H	-1.0999893243	3.6533867148	-1.8513337353
H	-1.7327987373	2.5015741113	-3.0749537638
H	-0.0018712993	2.4815260964	-2.6558753761
H	-1.0417179169	-3.4803499183	-1.4549483845
H	-3.2422221317	-3.7847976368	0.1136874027
H	-5.2225942744	2.5698738331	-1.0988323957
H	-6.1179314357	1.1081384526	-0.564688533
H	-5.4532096439	2.2273585681	0.6485656051
H	1.4108726099	1.4768143568	2.3252695092
C	5.0679191753	-0.3981830663	-2.296730385
H	4.9143901556	0.896705616	-0.6021060851
C	5.7714569637	-0.9644731235	0.0700097887
H	1.0341071993	-0.4388350898	1.4833270394
H	1.1852456448	3.7158313434	1.2442977073
H	2.664688274	3.4178363501	0.3084735487
H	1.21689499	2.4142981297	0.0406768745
H	2.4569879337	3.420002141	3.4603984631
H	3.3842637935	1.9312021396	3.7554480192
H	3.9701576942	3.1365842037	2.5897955563

H	6.0860047488	-0.07967561	-2.5516293278
H	4.9587989067	-1.4583226564	-2.5517514361
H	4.3611445558	0.1684123036	-2.9120098246
H	6.8059803768	-0.6721268072	-0.1477968702
H	5.5827749104	-0.7936692152	1.1362907059
H	5.6682652217	-2.0391467152	-0.1175188126

4bH^+

C	2.4996095802	-1.9018887127	2.0793687414
C	2.0763580974	-1.9953363225	0.5998651451
O	0.6479552428	-1.8028046138	0.7253492201
C	0.7379593687	-0.5659255896	1.4708559323
C	1.6710900736	-1.0123253277	2.6243583614
C	2.4513203018	-0.6475738775	-0.07079223
C	1.6265306386	0.2617986542	0.4745353388
C	1.5281187875	1.7286373882	0.3024484649
O	2.7053024205	2.3159127508	0.1200725162
C	2.6838357795	3.7466738935	-0.0938910663
C	3.6178714479	-0.5793330222	-0.9796950934
O	3.5302535194	0.3825804823	-1.9042125794
C	-0.6415446723	-0.051726437	1.8449950085
N	-1.4627479213	0.3500826338	0.7071300616
C	-2.2642727355	-0.4602519304	-0.0098330305
N	-3.1549213557	0.0858657648	-0.8551757593
C	-3.6050702955	1.5007586964	-0.8854858587
N	-2.1892090444	-1.7907339297	0.1510148941
O	0.4630231001	2.3373346189	0.3531791354
O	4.5330908897	-1.3716423138	-0.8702610803
H	-1.2000064488	1.2572558066	0.325122905
H	-3.6075808902	-0.5425401911	-1.5040929357
C	-4.3035620006	1.7390921475	-2.2255764552
H	-2.7125794782	2.1364212259	-0.8543897454
H	-0.5392688428	0.8245857382	2.4900966188
H	-1.167588189	-0.8246403723	2.4125917805
H	2.3249263651	-2.8976156592	0.0463552971
H	3.7283154211	4.0318501215	-0.2081659574
H	2.234826212	4.2486190773	0.7656701688
H	2.114205048	3.9821687141	-0.995452366
H	1.6581555872	-0.6052051617	3.6277854014
H	3.3467137882	-2.417053133	2.5128901429
H	-1.276050231	-2.1416281031	0.4398846777
C	4.6614160416	0.4817482555	-2.7997802073
H	4.4087570273	1.2819767803	-3.4941968041
H	5.5660890822	0.7247247849	-2.2379994444
H	4.8071604928	-0.4619915434	-3.3299777048
C	-3.1591493843	-2.7905074426	-0.3437579306
C	-4.5046171622	1.8235785689	0.3123874689
C	-3.1983483105	-3.9589597565	0.6455592634

H	-4.1402687169	-2.3012003951	-0.3361642969
C	-2.819146234	-3.2535530179	-1.7680464681
H	-4.7848620336	2.8816451054	0.2930776006
H	-5.422452679	1.2264737426	0.279702551
H	-3.9953671653	1.6264511662	1.2605624478
H	-4.6187299121	2.7834022267	-2.2982383865
H	-3.6381665876	1.5258995667	-3.0686542893
H	-5.2024215475	1.1166030736	-2.3177766022
H	-3.934394611	-4.6997008965	0.3197399688
H	-2.2243176644	-4.4598429257	0.700449097
H	-3.471955946	-3.6189457733	1.648734538
H	-3.5690960481	-3.9675760928	-2.1228217647
H	-2.7826828382	-2.4207838939	-2.4801231385
H	-1.8426683673	-3.7499215501	-1.7889485522

4clIa

C	3.003698349	-1.8255701738	-0.7538936378
C	2.4125082881	-0.4128570426	-0.5378015345
O	1.1196284934	-0.5733738302	-1.1409608591
C	0.740878551	-1.7306022339	-0.3705254382
C	1.9628222337	-2.6487039126	-0.6371492486
C	2.0000173242	-0.3191798737	0.9581142307
C	0.9410680758	-1.1391260255	1.0622301639
C	0.1907796135	-1.647891983	2.2290839162
O	-0.2522243706	-0.6826907864	3.0443797199
C	-0.9309784237	-1.1421243248	4.2227001864
C	2.7479632596	0.3803039376	2.014896423
O	3.7306563147	1.1467006705	1.4679337511
C	-0.6368908753	-2.2550444821	-0.7193604942
N	-1.657315942	-1.3443960019	-0.2423117756
C	-2.8641282409	-1.5447746838	-0.6121321391
N	-3.9259413856	-0.7280007192	-0.2266760955
C	-5.1904575457	-1.3926594301	-0.5210551422
C	-4.7908241052	-2.240087686	-1.7415464338
N	-3.3999191981	-2.5588781828	-1.4145555749
O	0.0035955089	-2.8389290779	2.4001820369
O	2.5596318349	0.2980722146	3.2117381199
H	-5.981800713	-0.6740846939	-0.7539541083
H	-5.5330583545	-2.0400811982	0.3016849991
H	-0.7414010169	-3.2514763489	-0.262794523
H	-0.6489784883	-2.3964994327	-1.8149959569
H	-4.8773451889	-1.6501046787	-2.6661783307
H	-5.3974757685	-3.1449021774	-1.8420711191
H	2.95092772	0.4387971473	-0.9478451562
H	-1.2291902398	-0.2390062444	4.7556194372
H	-0.2595478208	-1.7456115121	4.8391283941
H	-1.8056596425	-1.7410859603	3.9540602955
H	1.9318770422	-3.7312734555	-0.6404742528

H	4.0543442466	-2.0550641516	-0.8820614224
H	-2.8052408806	-2.9148726816	-2.1521047696
C	4.5339410011	1.8678983784	2.4125611379
H	5.26558271	2.4157256233	1.8175651782
H	3.9165946747	2.5583923284	2.9937496076
H	5.0336226214	1.1788347295	3.0991475517
H	-3.8026096618	-0.2506010437	0.6566228634

4cIIb'

C	3.0468028174	-1.6339527085	-0.8706797868
C	2.405137914	-0.2630361495	-0.5556570345
O	1.1190132418	-0.4299101849	-1.1804965198
C	0.7854555549	-1.659393797	-0.4957720089
C	2.0399363171	-2.5049841186	-0.8215349761
C	1.9796072606	-0.2916355251	0.937573639
C	0.9540618457	-1.1585732977	0.9776171458
C	0.2217117019	-1.7777807171	2.1035353741
O	-0.2844360163	-0.8876948051	2.9651866424
C	-0.9554978436	-1.4518362079	4.1055449294
C	2.6951971109	0.3591879635	2.0491663894
O	3.6554879585	1.1906463432	1.5667122879
C	-0.5704000397	-2.1785426689	-0.9110718353
N	-1.5738718076	-1.1622441263	-0.6207829041
C	-2.9013934855	-1.4790852685	-0.826854118
N	-3.8338073147	-0.4274933325	-0.7661123576
C	-5.1447031352	-1.0927480263	-0.6526344705
C	-4.8090241603	-2.5217705446	-1.169083256
N	-3.3499432599	-2.6581961985	-1.0687707357
O	0.0912353471	-2.9844430834	2.1915827615
O	2.493436189	0.1873324363	3.2336233573
H	-1.3090579428	-0.2382015648	-0.9458857271
H	-5.902426663	-0.5824114074	-1.2551557878
H	-5.4913224516	-1.1152654855	0.390818364
H	-0.8128756042	-3.0797419128	-0.3432919519
H	-0.5464743851	-2.4520242167	-1.9756296512
H	-5.1168458085	-2.6424821339	-2.217538754
H	-5.3193266686	-3.3015362512	-0.5921948535
H	2.912284348	0.634587883	-0.9018357698
H	-1.3181979426	-0.5982531101	4.6780734058
H	-0.2562703019	-2.0436545856	4.701506872
H	-1.7853099196	-2.0877137784	3.7861422098
H	2.0479081452	-3.5837745228	-0.911771263
H	4.1050206965	-1.8110451058	-1.0159748229
C	4.4308092028	1.8697860625	2.5667085027
H	5.1497463674	2.4776806826	2.0167400618
H	3.7879068322	2.500152677	3.1868814875
H	4.945024122	1.149343824	3.2085058365
H	-3.6358242223	0.2713509631	-0.0566136826

4cH+

C	2.964961047	-1.5363685572	-0.7579142557
C	2.2809847816	-0.1847242501	-0.4681052737
O	0.9887856936	-0.4139902527	-1.0834056377
C	0.7102629759	-1.6395212393	-0.3632613283
C	1.9923878992	-2.4442699724	-0.691775712
C	1.8489055021	-0.1915392393	1.0240933555
C	0.8720362241	-1.1091851756	1.102425408
C	0.0794002772	-1.5887455664	2.2607641306
O	0.7974604748	-1.7194775659	3.3635771909
C	0.0833866881	-2.0971741106	4.5656979988
C	2.4348392641	0.6684289071	2.0820019456
O	3.6759210555	1.0469410026	1.7224570909
C	-0.6228010523	-2.2271768325	-0.7897939805
N	-1.7698386717	-1.3827800448	-0.4503649416
C	-2.1702096909	-0.3393967144	-1.1709889853
N	-3.2487647956	0.4090620516	-0.8656515
C	-3.5589563738	1.3301052943	-1.9745220095
C	-2.2248855949	1.3411157714	-2.7537290501
N	-1.58596473	0.0858812031	-2.3082937883
O	-1.1168851399	-1.8588390947	2.1811802608
O	1.8706293124	0.9997520028	3.1008146074
H	-2.0284918156	-1.4377653754	0.538750836
H	-3.980932852	0.040561229	-0.2717565536
H	-3.8364496514	2.3163098933	-1.5981212442
H	-4.3805475188	0.9329900787	-2.5809438276
H	-0.7829378493	-3.1883460023	-0.2962814732
H	-0.6141421392	-2.4046574935	-1.8692365973
H	-1.6012704605	2.1975345018	-2.4751465458
H	-2.3707686645	1.3427291264	-3.8352165463
H	2.751490461	0.7229863931	-0.837557974
H	0.8464834511	-2.1553602387	5.3395778571
H	-0.4068411515	-3.0624420322	4.4238872301
H	-0.6591974839	-1.3347283717	4.8089234716
H	2.0480652033	-3.5227538558	-0.7722652607
H	4.027845595	-1.6741694306	-0.9077813862
H	-0.5744220733	-0.0146505523	-2.3741653285
C	4.3710069363	1.8884643315	2.6704478085
H	5.3417350273	2.0902265847	2.2197475798
H	3.8139265344	2.8144655902	2.8295720723
H	4.4853433049	1.3658540067	3.6226873564

4dIa

O	4.1334627318	1.1756046945	-0.5853134398
O	0.7772591469	1.2859933546	2.5360335824
O	0.3260622535	-0.8841166537	3.0026032641
O	0.7305415169	-0.9038416627	-1.2965860341

N	-2.0417810379	0.0198181223	-1.7519486209
N	-3.7055527154	0.5765621094	-0.1250045627
N	-1.7736503519	-0.5053032348	0.5168332444
H	-2.2885077382	-0.4999105158	1.3890636489
C	0.7214765154	-0.0159743977	2.2435906112
O	3.5639673575	1.4399615786	1.591616132
C	1.1698720892	-0.3312983002	0.870420247
C	2.2322323028	0.0573771769	0.1451592327
H	-1.0304913152	-0.0344529557	-1.8088992456
C	-2.5773553113	0.0692968725	-0.4802587006
C	-2.746400295	0.6987966261	-2.838858595
H	-3.3967515029	-0.0146716174	-3.3657687886
H	-2.0041881628	1.0509585414	-3.562822393
C	0.4777210884	-1.4469351097	0.022955565
C	3.3438935443	0.9602356584	0.4997017865
C	-4.4485811305	1.3319949491	-1.1261712417
H	-4.9441708008	2.1735131079	-0.62400908
H	-5.2593051851	0.703471293	-1.5298345566
C	-0.9953663064	-1.719675298	0.2863333668
H	-1.3897201918	-2.3029513896	-0.5550579275
H	-1.0546761093	-2.3377537454	1.1857370997
C	-3.5857840174	1.8532051294	-2.2852085777
H	-4.2107798195	2.286223993	-3.0754170433
H	-2.9165525263	2.6422518509	-1.9187077629
C	2.1609717498	-0.8066470716	-1.1429125193
H	2.638616282	-0.3984843211	-2.0304692963
C	5.2604100674	2.0361645867	-0.3559834299
H	5.9125776005	1.6173322572	0.4150965024
H	4.9262180124	3.0269640171	-0.0370067893
H	5.7822017281	2.0956305804	-1.311519385
C	1.4491054356	-2.6538938141	-0.0030071279
H	1.2945762719	-3.5790019142	0.5380754814
C	2.4890970723	-2.2600430039	-0.7362475422
H	3.4145344952	-2.7771882698	-0.956607927
C	0.3803400935	1.6378367707	3.8725926865
H	0.4886031601	2.7206217546	3.9289370007
H	-0.656749744	1.3426962444	4.0527516493
H	1.0281237464	1.1471810063	4.6032404859

4dIIa

C	0.3991655423	-1.4111913452	-0.1567657933
C	1.0147041599	-0.3304956449	0.7900772518
C	2.0779555927	0.152887497	0.1264394711
C	2.0780450543	-0.6286323398	-1.217876025
C	2.4679982028	-2.0887859244	-0.8896232448
C	1.4251516453	-2.5731556392	-0.2172368969
H	2.5633525819	-0.1436127299	-2.062020671
H	3.4251804466	-2.5478975498	-1.1039968245

H	1.3003444702	-3.5329516158	0.2687453761
O	0.6641540734	-0.7722734733	-1.4202131679
C	-1.0623501023	-1.7554644312	0.049622209
H	-1.3894088767	-2.3239400217	-0.83782064
H	-1.1061804744	-2.4447958455	0.9125806042
N	-1.8317422605	-0.5513847152	0.2643602463
C	-3.1036765481	-0.6496866323	0.4475921199
N	-3.8374944478	-1.8285181735	0.4553083191
C	-5.1704650086	-1.9238694938	1.0520457112
C	-5.9563047433	-0.6428016362	0.7794014797
C	-5.1283837093	0.5601721717	1.2296464481
N	-3.8435980956	0.5198640281	0.5471508047
H	-3.2685596186	-2.6579012283	0.5537480787
H	-5.6807332356	-2.7907475421	0.6169982943
H	-5.1111327702	-2.0914148383	2.1405501345
H	-6.1661052749	-0.5578460197	-0.2926464184
H	-6.9109805477	-0.672977395	1.3155761389
H	-5.6305920987	1.4961615056	0.9627023455
H	-5.023902871	0.5464914804	2.3295283185
H	-3.2355052206	1.3233621578	0.6426815662
C	0.5424574785	-0.1516061531	2.1783039026
O	0.2936821042	-1.1012901129	2.8994798434
O	0.4129754625	1.1314829246	2.5393768724
C	0.011176971	1.3457348822	3.900275278
H	-0.9590156626	0.8790202847	4.0926957971
H	0.7518399777	0.9287510775	4.5876665317
H	-0.0507574969	2.4279146305	4.0171056123
C	3.1306380889	1.0715055655	0.588023031
O	3.2518085471	1.5327878292	1.7048868932
O	4.0005616741	1.3375037632	-0.4242492462
C	5.0789701376	2.2189397778	-0.0813518772
H	4.6941794676	3.1940277215	0.2299755584
H	5.6784570365	2.3152310826	-0.9873320139
H	5.6758993484	1.7993541212	0.7331805813

4dH^+

O	4.0391514664	1.2269776618	-0.3819833187
O	1.6347900805	0.4525564778	3.11223257
O	-0.5646565168	0.217588301	2.6766479819
O	0.5044636178	-0.7781930382	-1.2141749069
N	-2.2603725473	-0.3414148306	-1.8546098125
N	-3.6042081334	0.8348319608	-0.3641423331
H	-3.9866667622	0.8515470967	0.5724495941
N	-1.9994631193	-0.6089574577	0.4571870715
H	-1.9109648492	-0.0776840704	1.3264480183
C	0.6098195971	0.1843900395	2.3190494773
O	2.7673969523	2.3205149524	1.1501470861
C	1.0227262301	-0.1994920902	0.9469194302

C	1.9946799034	0.2835228506	0.1566159653
H	-1.294833238	-0.6386950748	-1.9867106439
C	-2.6073294476	-0.0343927555	-0.5973124816
C	-2.9994816007	0.2089384546	-3.0018203859
H	-3.8580921831	-0.4354110194	-3.2241766182
H	-2.3312287345	0.1812503252	-3.8644454885
C	0.3659917746	-1.3500958274	0.1095482162
C	2.9506340928	1.3928628802	0.3936216071
C	-4.2754507511	1.6391460232	-1.3984680757
H	-4.3992370057	2.6559727507	-1.0145097973
H	-5.2734416812	1.2215052423	-1.5776992508
C	-1.0762786756	-1.7392175332	0.3872022033
H	-1.4146578214	-2.4359686731	-0.3852003456
H	-1.1365044904	-2.2565077522	1.348321869
C	-3.4508879191	1.6361505242	-2.6851507775
H	-4.0567115457	2.036882485	-3.5028929916
H	-2.5718575072	2.2806282492	-2.5736781044
C	1.9374358637	-0.5807117563	-1.1325474706
H	2.3388432861	-0.13574834	-2.0395466686
C	5.0615713803	2.2396608509	-0.2470884381
H	5.4300343456	2.2665792119	0.7807560173
H	4.6576908614	3.2191248582	-0.5131027586
H	5.8527472845	1.9447237347	-0.9350540943
C	1.414478968	-2.48737144	0.020737082
H	1.3559442644	-3.4306147587	0.5495050396
C	2.3864688156	-2.0069712101	-0.7531143676
H	3.3343552999	-2.4518524198	-1.0265286871
C	1.3206226479	0.920644269	4.4457186558
H	2.285630334	1.0672749755	4.9273601563
H	0.7693954773	1.8612738465	4.3869476227
H	0.7262889861	0.1738490257	4.9761111533

4elc

C	2.7146879223	-2.3071910386	1.9022712106
C	2.1060026675	-2.1417978897	0.4912622656
O	0.7684862295	-1.7320611076	0.8395448945
C	1.1684270018	-0.6204075222	1.6844124638
C	2.1424467898	-1.3551921285	2.6379711504
C	2.6443185941	-0.8065027873	-0.089213631
C	2.0530378627	0.1460279761	0.6505026432
C	2.2731242904	1.606591998	0.7498396503
O	2.1417560224	2.2491242931	-0.415785636
C	2.3881779061	3.6644522228	-0.3619542952
C	3.7012229708	-0.6730449247	-1.1047269728
O	4.0008644406	-1.8884787122	-1.6363258301
C	-0.0170925394	0.1272446911	2.2718050764
N	-0.7122219331	0.9344517681	1.2900689392
C	-1.6822275243	0.4270268756	0.6074366272

N	-2.0295232976	-0.9306638415	0.6077894857
C	-3.2081977508	-1.5447832297	0.1506925116
N	-2.4612654363	1.2484066504	-0.1810654101
O	2.5397165367	2.1341685672	1.8127737657
O	4.2570661995	0.3546220227	-1.4340099921
H	0.3796869976	0.7996420409	3.0416372178
H	-0.6621767304	-0.61003149	2.7764988247
H	2.108416767	-3.0011283501	-0.1754620896
H	2.2406499845	4.0220101119	-1.3810509633
H	3.4113381182	3.8598777042	-0.0313329471
H	1.6873486396	4.1468060287	0.3243723519
H	2.3292781995	-1.065631904	3.6642899687
H	3.4959118788	-3.0087405118	2.1670674234
H	-2.8693232628	0.7989579483	-0.9921904948
C	5.0337771925	-1.8774488819	-2.6333402071
H	5.1512992581	-2.9158296829	-2.944923101
H	4.7417472075	-1.2517981296	-3.4810898822
H	5.9676751794	-1.4921439706	-2.2151320796
C	-2.0944705574	2.6490222239	-0.3584692066
H	-1.2213704394	-1.5407053966	0.6810367139
H	-1.9632792776	3.0838031826	0.6367204016
C	-3.1773222331	3.3969793055	-1.1379459387
H	-1.120328637	2.7408773516	-0.8644895916
C	-2.8253275058	4.8724001488	-1.3501747805
H	-3.3267734771	2.9146802607	-2.1155785707
H	-4.1302037882	3.3107581901	-0.6000007089
H	-3.6117023693	5.390191029	-1.9098141733
H	-2.7003813548	5.3911968144	-0.3921968137
H	-1.8895252774	4.981197941	-1.9115491045
C	-3.1203488872	-2.8448197857	-0.3784118799
C	-4.2651418603	-3.5226028031	-0.7893297882
C	-5.5202098829	-2.9176270021	-0.695759337
C	-5.6114410271	-1.6278729182	-0.1697895256
C	-4.4750746839	-0.944118026	0.2604525275
H	-2.1450175508	-3.3185328203	-0.4640227669
H	-4.171788545	-4.5278103326	-1.1923840543
H	-6.4119543996	-3.4442405926	-1.0229586494
H	-6.5817595613	-1.1464100221	-0.0772121098
H	-4.5628780672	0.0478184552	0.6882044175

4ellb'

C	3.395394904	-2.8708791135	-1.1704476682
C	3.2720941729	-1.4351928879	-1.727471228
O	1.8624379098	-1.3898126219	-2.0188017272
C	1.4316053265	-1.7898278684	-0.6985335366
C	2.2530439313	-3.0872097171	-0.5194347332
C	3.3219422395	-0.4688989304	-0.5159878971
C	2.163023977	-0.6742855035	0.1307094156

C	1.6360428344	-0.217717234	1.434514953
O	1.5969648848	1.1183193046	1.5640581769
C	1.1005789838	1.5892209114	2.8300447868
C	4.5318777999	0.3118419477	-0.2150421716
O	4.4820718867	0.9456817626	0.9739662831
C	-0.0742898532	-1.8537300219	-0.596020618
N	-0.6106490261	-0.5339016123	-0.8890053513
C	-1.959895031	-0.2833050181	-0.7013169784
N	-2.3767198527	0.9555858967	-1.1513232775
C	-1.4844346161	2.1051730368	-1.2985068036
N	-2.7082223472	-1.1895404724	-0.1630885691
O	1.2483332317	-1.0029661371	2.2781625177
O	5.4891489647	0.3586051004	-0.9672944463
H	-3.3299631286	1.1727897775	-0.8891906323
H	-0.8891464196	2.0025067708	-2.2183056304
C	-2.2917667376	3.4021990844	-1.380486942
H	-0.368461013	-2.1358407005	0.4170432107
H	-0.4568127504	-2.625517012	-1.2786949255
H	3.8818414514	-1.1589840932	-2.584853739
H	1.1163383224	2.6770363307	2.7605343162
H	1.7447431542	1.2460540067	3.6437179784
H	0.0842261616	1.2258393767	3.0022320285
H	1.9420619284	-3.9347438682	0.0780709789
H	4.2739300518	-3.4984148168	-1.2510342619
C	5.6463696391	1.7211407183	1.3016119851
H	5.4368332049	2.161523361	2.2766601
H	6.5324113467	1.082504102	1.3498065647
H	5.8112636433	2.5013275088	0.5537477972
C	-4.0257241623	-0.902476015	0.2198565005
H	-0.7693908206	2.1529947312	-0.463916478
H	-0.1920925726	-0.1022242542	-1.7048601153
C	-1.4053809612	4.632798125	-1.5903838634
H	-3.0152915068	3.3190507559	-2.2024370122
H	-2.8764157163	3.5188658804	-0.4572504004
H	-2.0076901938	5.546015589	-1.6388220953
H	-0.6857748689	4.7502326206	-0.771377864
H	-0.8370797121	4.5583255862	-2.5250384464
C	-5.0881634886	-1.6199085539	-0.3611513778
C	-6.4026060152	-1.4057440793	0.0475077577
C	-6.6942815853	-0.4755305583	1.0491681318
C	-5.6493840009	0.2352928858	1.6418535877
C	-4.330637694	0.0269357781	1.2358247832
H	-4.8562429292	-2.3475030655	-1.1335672212
H	-7.2059978522	-1.9716272345	-0.4182563986
H	-7.7201099766	-0.3146772022	1.3690626027
H	-5.8584860352	0.9518264765	2.433003326
H	-3.5169460837	0.5597681662	1.7220756274

4ellc'

C	2.9950620478	0.6933233795	-2.8494577135
C	2.463308591	1.6082551644	-1.7232320438
O	1.0561675663	1.3126782712	-1.791649957
C	1.2050730338	-0.1197020332	-1.671892427
C	2.21885641	-0.3888738905	-2.8090011976
C	2.8135283415	0.922916383	-0.3779352572
C	2.0190507318	-0.1593443488	-0.3296999791
C	1.9623529267	-1.3358152638	0.5647144189
O	1.8096555478	-1.0234942241	1.8591375672
C	1.7331907517	-2.148885518	2.751536259
C	3.9269238705	1.4102812022	0.4509540235
O	4.2243167458	0.6008389849	1.4868574985
C	-0.1298488753	-0.8248175261	-1.70712622
N	-0.9711880593	-0.2941864327	-0.648611484
C	-2.1991110654	-0.8773343829	-0.3958529329
N	-3.091904354	-0.1239377837	0.3841229826
C	-3.0023379667	1.2260816942	0.7778903076
N	-2.4443869789	-2.0634939015	-0.8236955093
O	2.0177964409	-2.4704266608	0.1289139819
O	4.5288856508	2.4404085763	0.2027240052
H	0.0097738149	-1.896250345	-1.5481411721
H	-0.5804741769	-0.6953224788	-2.7026619577
H	2.6667009112	2.6750163351	-1.7837845174
H	1.5766483886	-1.7217924314	3.7422230242
H	2.6622992284	-2.7242294682	2.7220878327
H	0.9003370991	-2.800001301	2.474015473
H	2.2795926432	-1.3112107003	-3.372591066
H	3.8649223794	0.8970572872	-3.4610073114
C	5.3047251091	1.0485664619	2.3209044902
H	5.4057201633	0.2896349567	3.0970585377
H	6.2282549723	1.1329221159	1.7418304691
H	5.071829505	2.0215479646	2.7616955306
C	-3.7329625753	-2.6656648367	-0.5403089307
C	-3.8930710326	-3.9770925104	-1.3151540021
H	-3.8460374361	-2.8894186377	0.539996268
H	-4.5680246285	-1.9888960396	-0.7989323322
H	-0.8777257352	0.6990204321	-0.4858162407
C	-3.3061509982	1.5780401023	2.1026493797
C	-3.2835297869	2.9121194541	2.5029080488
C	-2.9356318781	3.9173773442	1.5981526624
C	-2.6340840538	3.5724121471	0.2800309952
C	-2.6827759381	2.2427612994	-0.1381326289
H	-3.5519069938	0.796596234	2.818161461
H	-3.5254578659	3.1639244906	3.5321264142
H	-2.9065370094	4.955768498	1.9148563733
H	-2.3790390025	4.344458307	-0.4408927218
H	-2.505340522	1.9953085424	-1.1802546421

H	-3.059555881	-4.6395358474	-1.0502077243
C	-5.2307997602	-4.6717765332	-1.0488187231
H	-3.7843332897	-3.7598983786	-2.3853577767
H	-5.3146883827	-5.604306821	-1.6179549268
H	-5.3480354823	-4.9213495518	0.0136018494
H	-6.0772492441	-4.0334843377	-1.3325143772
H	-3.7192078979	-0.6918354435	0.9363969184

4eH^+

C	-3.4344775886	1.7470828023	1.3299734893
C	-2.8924431241	1.5071895477	-0.0942002723
O	-1.4799814582	1.7405711388	0.1281238291
C	-1.3516916713	0.7957630634	1.2186040679
C	-2.4817544033	1.3034706445	2.1485644653
C	-2.8551724918	-0.0282113977	-0.3259365497
C	-1.9024588766	-0.4847165085	0.5022477938
C	-1.4225533692	-1.8646267978	0.7583116849
O	-2.4036426253	-2.7511830462	0.8220324288
C	-2.0172087236	-4.1372807743	0.9789286663
C	-3.7147352442	-0.768747588	-1.2809465326
O	-4.8249571612	-0.0530756906	-1.5446897122
C	0.0659801088	0.7849989526	1.7655621174
N	1.0666524948	0.3196338198	0.8085049705
C	1.6994563286	1.085723836	-0.0951812973
N	1.2980945206	2.3622109437	-0.2976543511
C	2.0136920025	3.3333643927	-1.0727346564
N	2.7451610693	0.5843837961	-0.7604700917
O	-0.2363231516	-2.1367398646	0.9207911961
O	-3.4457241567	-1.8438000543	-1.7696442117
H	1.0576191582	-0.6934245988	0.6809755026
H	0.1187564819	0.1194271713	2.6311178569
H	0.3291552191	1.7915285978	2.1025672624
H	-3.2822378736	2.1164627774	-0.9057111703
H	-2.9548560963	-4.6885420136	1.020197804
H	-1.4449684751	-4.2664501768	1.899905728
H	-1.418798121	-4.4530779686	0.12205422
H	-2.4793727341	1.2300372647	3.2289683668
H	-4.4183166798	2.1336174076	1.5620708418
H	3.094276214	1.1367984165	-1.5347852541
C	-5.7566440193	-0.6654178445	-2.464178049
H	-6.5801246563	0.0416452124	-2.5548254995
H	-5.2761817204	-0.8314085727	-3.4310743965
H	-6.1060142373	-1.6201387591	-2.0648563034
C	3.4001885038	-0.7019402667	-0.4832882988
H	0.2969992405	2.5158933129	-0.174710672
H	3.4611108012	-0.8252708925	0.6040142875
C	4.7978871503	-0.7384655466	-1.1051120666
H	2.7896163182	-1.5250001144	-0.878941629

C	5.5035198209	-2.0726043916	-0.8439798245
H	4.7152461749	-0.5712989869	-2.1879629075
H	5.392775542	0.0886101917	-0.6975458054
H	6.4965146556	-2.0764867644	-1.3025078387
H	5.6314400091	-2.2534408776	0.2292264314
H	4.9390268246	-2.9125770375	-1.2641862075
C	1.3706694345	3.9441637525	-2.1540747054
C	2.0361500707	4.928962547	-2.8840888769
C	3.3426453131	5.2902067549	-2.5498343502
C	3.9805725979	4.6743856353	-1.4709244458
C	3.3171616237	3.7034221306	-0.7205360211
H	0.358859637	3.6494823093	-2.4189907772
H	1.5335946712	5.4077791455	-3.7189469198
H	3.8594817098	6.053422682	-3.1233147097
H	4.9908994659	4.9630550978	-1.197245387
H	3.7956744962	3.2522351893	0.1436127799

4fla

C	1.3786545746	-2.4737199135	1.6028179412
C	1.1715594171	-2.0297401566	0.1367573465
O	0.0733040751	-1.1181198599	0.2918649409
C	0.6955608086	-0.2976792074	1.306152965
C	1.0914411285	-1.395592954	2.3300960231
C	2.3009288236	-1.0153629763	-0.1972470698
C	2.0188127541	0.0673557717	0.5457759181
C	2.7575394315	1.3412537041	0.7090392324
O	4.0808241358	1.1607725677	0.793743509
C	4.8707188481	2.3629514747	0.8606769026
C	3.4172723016	-1.2621039953	-1.1305374909
O	3.5793480722	-2.5967790959	-1.3116274158
C	-0.2278571614	0.8184050419	1.7875821303
N	-0.6549204951	1.748456048	0.7706298921
C	-1.7997443697	1.6411824383	-0.0079318469
N	-2.2477527386	2.5571900664	-0.7925989661
C	-1.5731479118	3.8435142458	-0.8157297541
N	-2.5160213453	0.454664286	0.1695048354
O	2.2143886218	2.4306418285	0.7910819432
O	4.0882608256	-0.4182568248	-1.6851168914
H	0.0410483299	2.4234545345	0.4847478846
H	0.2918423101	1.3707934877	2.5799245496
H	-1.1125164043	0.355485145	2.2342359301
H	0.9651852806	-2.7936968994	-0.6093452153
H	5.9039530635	2.0237103007	0.9279259364
H	4.5962868472	2.9528732787	1.7388079329
H	4.7197627426	2.9623704671	-0.0401997627
H	1.1574777342	-1.2476062154	3.4011248117
H	1.7451628192	-3.4430701039	1.9162118517
H	-1.9290240704	-0.37536166	0.14676879

C	4.631318132	-2.9667689592	-2.2191129746
H	4.6236870532	-4.0565516515	-2.2462571688
H	4.4402448059	-2.5539131295	-3.2130788226
H	5.5946590712	-2.5961590534	-1.8592367238
H	-0.5768401433	3.7639982146	-1.2893622063
H	-1.3922497736	4.2266004643	0.205055176
C	-2.4036364542	4.8605458455	-1.5985154642
C	-3.741827475	0.3025336895	-0.6168689606
C	-4.4700253389	-0.942341964	-0.2386040273
H	-4.3808481685	1.1665160572	-0.423791347
H	-3.5300101885	0.3130906289	-1.6973505723
C	-5.6765411477	-1.1912731141	0.3451681502
C	-5.8109967356	-2.6176465194	0.4286088012
C	-4.6732377952	-3.133923349	-0.1078604589
O	-3.8450894989	-2.1314566739	-0.523911887
H	-4.3100028257	-4.1380109816	-0.266543568
H	-6.6433498444	-3.1763595139	0.8339258227
H	-6.38556087	-0.446136793	0.6787303837
H	-1.902067071	5.8350412711	-1.6398226138
H	-2.5692639104	4.5097450862	-2.6228523468
H	-3.3845632704	4.994220621	-1.1292180458

4fIIa

C	-3.5388178391	-2.2741919096	-1.2744428052
C	-3.388135756	-1.7418992728	0.1695853139
O	-2.0028249205	-2.0303199544	0.4135170599
C	-1.5034195901	-1.3469551439	-0.7531506937
C	-2.36684743	-2.0166313156	-1.8531621523
C	-3.3258048271	-0.1910222808	0.0774966457
C	-2.1356663339	0.0592831153	-0.4925915966
C	-1.5267937314	1.2971020796	-1.0228120471
O	-1.5051358727	2.306273555	-0.1427077333
C	-0.9706915393	3.5407356002	-0.6442200953
C	-4.4229406791	0.7360430133	0.3943250935
O	-5.4409524268	0.070745783	1.0061445167
C	0.0059424174	-1.4052219644	-0.8804792381
N	0.6126164324	-0.5570028411	0.1202633534
C	1.861184723	-0.6990005238	0.3974851579
N	2.4159941303	-0.0311955271	1.4817001208
C	1.5457468946	0.8281187332	2.2839222655
N	2.7278269982	-1.5582253527	-0.2749341897
O	-1.0887686174	1.3588549755	-2.1573947686
O	-4.4617027707	1.9245803434	0.1476051426
H	0.2544386815	-1.0726481318	-1.9028568575
H	0.3092507367	-2.4622713601	-0.797120141
H	-4.032911608	-2.1575377135	0.9409400476
H	-1.030137338	4.2413751493	0.1889032951
H	-1.5649950278	3.8989498785	-1.4888151919

H	0.0661498288	3.4071917874	-0.9649561386
H	-2.0586463651	-2.1527080485	-2.8824552076
H	-4.446197425	-2.6809110629	-1.7036960025
C	-6.5733845339	0.8803901728	1.3513958706
H	-7.2897569068	0.2006828547	1.8143668754
H	-6.2834100052	1.6682626902	2.0520991906
H	-7.0030299962	1.3436540899	0.4588285559
H	0.6968177016	0.2218926786	2.6105968348
H	1.1198178804	1.6424672521	1.6794305147
C	2.3116792717	1.3840250589	3.4814728105
C	4.1015042071	-1.8527344312	0.1165476676
C	5.1466065177	-0.9381350918	-0.4462430688
H	4.1332956651	-1.8433189534	1.210589591
H	4.3420765293	-2.8721056297	-0.2055822725
H	3.3269947456	0.3737461032	1.2896008486
C	6.1304946436	-1.0909996334	-1.3773898795
C	6.791976282	0.1773487499	-1.4956537124
C	6.1621003863	1.0140812326	-0.6298979118
O	5.1583912667	0.3534009553	0.02238626
H	6.290563571	2.051926126	-0.3631976492
H	7.6230908349	0.4263613158	-2.1405726015
H	6.3612464878	-1.9998116538	-1.9164305569
H	1.6560266961	2.0135191152	4.0924313046
H	2.6985136819	0.5754391737	4.1112627912
H	3.1616346829	2.0031055038	3.1652312966
H	2.4961776455	-1.7303332902	-1.242876913

4fIIc'

C	3.7055693943	1.5755261942	-2.1146424172
C	3.3479318439	1.9058532363	-0.6474520439
O	1.9670083255	2.2852973386	-0.7975623768
C	1.5596932272	1.0763295744	-1.4816866298
C	2.595465087	1.0487706698	-2.629918968
C	3.1702318322	0.5540199931	0.096152675
C	2.0427048946	0.0352223972	-0.4169023036
C	1.4113396314	-1.2955797663	-0.278229264
O	1.1403497579	-1.6270537872	0.9903264874
C	0.5649605295	-2.9336157022	1.1700184923
C	4.139331725	-0.063660966	1.0162906442
O	5.1401136952	0.8098341083	1.3039224001
C	0.0878408304	1.0885995007	-1.8190402072
N	-0.6649679889	1.2023385875	-0.5802323284
C	-2.0531162296	1.1120759341	-0.6190016418
N	-2.6884152201	1.4601134925	0.5700709305
C	-1.9678678724	1.5750899222	1.8400763849
N	-2.615850875	0.730892071	-1.7151015267
O	1.154210701	-1.9826309872	-1.2483771522
O	4.0892636773	-1.1923440971	1.4608008384

H	-0.2851387718	1.8980094092	0.0499646486
H	-0.1887882389	0.155179805	-2.3135108679
H	-0.1235176547	1.9080380472	-2.5211434496
H	3.927034981	2.6718726638	-0.1366444199
H	0.3943529688	-3.0283947942	2.2423779571
H	1.258901014	-3.7031842044	0.8229011896
H	-0.3742778363	-3.0171726449	0.6172844986
H	2.4195937863	0.6146370328	-3.6059256213
H	4.6851701052	1.6912627918	-2.5611866258
C	6.1537866479	0.3041273755	2.1861206356
H	6.8717145885	1.1164226856	2.3034260861
H	5.7198722543	0.0311421727	3.1518069678
H	6.635251915	-0.5769664911	1.7533185591
H	-1.32889186	2.4685813835	1.8168435263
H	-1.3052370794	0.7120433871	2.0052276721
C	-2.9581037754	1.7116835393	2.993876709
C	-4.0698256601	0.6235808282	-1.7761800255
C	-4.6438370557	-0.6396700227	-1.2054670602
H	-4.572684897	1.4834527139	-1.2998505806
H	-4.3512633822	0.6496292468	-2.8347685089
H	-3.5901206492	1.0113235694	0.6728934075
C	-5.010948476	-1.8383945289	-1.7398703953
C	-5.4519352352	-2.6661169986	-0.6534822417
C	-5.3230845001	-1.9159235905	0.4719754361
O	-4.8349465941	-0.676176558	0.1589342784
H	-5.5303692606	-2.0893042846	1.516976332
H	-5.8187685703	-3.6818180131	-0.7093474689
H	-4.9663880609	-2.1036326737	-2.7870420862
H	-2.4253288554	1.8270704413	3.943123155
H	-3.6043923112	2.584614984	2.8523158568
H	-3.595504503	0.8227390142	3.0723684427

4fH^+

C	-2.5619506231	-1.9272172082	-1.6114062221
C	-1.924236802	-1.7246824794	-0.2212328017
O	-0.5506987946	-1.4727167128	-0.6048771635
C	-0.8567823263	-0.3971998495	-1.5246282747
C	-1.9032702937	-1.101940909	-2.4236889634
C	-2.3107935197	-0.3009598789	0.2646580707
C	-1.657807837	0.5385601387	-0.5545064322
C	-1.6485194276	2.0206409502	-0.6130043888
O	-2.8448291611	2.5543901099	-0.4235326158
C	-2.9109975141	3.9994894334	-0.3717543477
C	-3.2143042936	-0.0059557491	1.403471066
O	-4.0220182944	-1.060232231	1.6275590889
C	0.4051165303	0.1302456234	-2.1868738393
N	1.3414289725	0.7641926857	-1.2625174206
C	2.2899507546	0.1259460623	-0.553212566

N	3.2429119705	0.8507201346	0.0512453614
C	3.4279435186	2.3087264223	-0.0994884647
N	2.3266271611	-1.2159762691	-0.508275728
O	-0.632619285	2.6719006676	-0.8419439377
O	-3.2084708117	1.0180907577	2.0499375929
H	1.0358063276	1.6914367229	-0.9624010974
H	0.1371620045	0.8809023301	-2.9350861841
H	0.9098100473	-0.6905144845	-2.7042453682
H	-2.0019837043	-2.5286759553	0.5063119112
H	-3.9639252152	4.2280542602	-0.2172995011
H	-2.5501196034	4.4264392873	-1.3097538519
H	-2.3078397647	4.3674325422	0.460623886
H	-2.0711771952	-0.8854202796	-3.4713838584
H	-3.4112274379	-2.5656409208	-1.8172321742
H	1.421427414	-1.6787889808	-0.582103021
C	-4.9616093478	-0.9055399312	2.7146916725
H	-5.525105798	-1.8371353783	2.7442700493
H	-4.4268779976	-0.7427590694	3.6532348018
H	-5.622263763	-0.0572710737	2.522402733
H	2.6446633537	2.8372128557	0.4622951529
C	4.7801585795	2.7050159312	0.3753661742
H	3.3238462321	2.5684516234	-1.1564275371
C	3.4414901669	-1.9982639757	0.0426807303
C	3.3301069982	-3.4571026185	-0.390844257
H	4.3753901237	-1.5617492073	-0.3272386762
H	3.4490215381	-1.9320947116	1.1405736834
H	3.7892425016	0.4092728756	0.7793283685
C	5.8530453786	3.3070179256	-0.2087491382
C	6.85647415	3.4214674253	0.809673667
C	6.3234049175	2.8714990962	1.9328661775
O	5.0518175238	2.428927291	1.6916107071
H	6.6869453338	2.7221692535	2.9379448801
H	7.8409053165	3.8559426973	0.7102024521
H	5.9253874369	3.636522119	-1.2361649339
H	4.1621452646	-4.0304749721	0.0273046825
H	2.3995881243	-3.9095377479	-0.0301278828
H	3.3633811704	-3.5469616284	-1.4808392618

b). Optimized structures used for calculations of $\Delta_r G$

4aH$^+$

C -2.5456031779 -1.9012602851 -1.554280261
C -1.9301618344 -1.6948202187 -0.1544594405
O -0.5482573871 -1.4675634087 -0.5103564611
C -0.8138543225 -0.400247076 -1.4528151188
C -1.8575678211 -1.0948830489 -2.3614948379
C -2.3045909384 -0.2583199213 0.3046616523
C -1.6212194229 0.5587690385 -0.5130961269
C -1.6148473813 2.0368862226 -0.6171410123
O -2.8302463511 2.5669891811 -0.4962339426
C -2.9044045105 4.0119417204 -0.5359032287
C -3.2375599172 0.0650946122 1.4043650583
O -4.1089918671 -0.9449232187 1.5820096821
C 0.4605051647 0.0995289941 -2.10956304
N 1.4196677545 0.6923127724 -1.1857990724
C 2.3840869577 0.0321115212 -0.519938112
N 3.3678697218 0.7266924091 0.0633487472
C 3.5465730642 2.1698984299 -0.0559406227
N 2.3960247344 -1.3099356845 -0.4939745285
O -0.6009843236 2.6925830273 -0.8204590555
O -3.2132608082 1.0796826093 2.0738352557
H 1.2177765661 1.6513433503 -0.9210689857
H 0.2017704708 0.8595826249 -2.8502240113
H 0.9330294001 -0.7304513367 -2.6411296008
H -2.0388572006 -2.4885427734 0.5803117072
H -3.9619224775 4.2484943447 -0.4262480163
H -2.5255161895 4.3859596378 -1.4893480596
H -2.3290097208 4.442787725 0.2863207992
H -1.9913883945 -0.8821779752 -3.4142917553
H -3.3999214766 -2.5305693644 -1.7667375466
H 1.4946468634 -1.7659921022 -0.5934809634
C -5.0733586339 -0.7680545479 2.6429401062
H -5.6817798389 -1.6716908493 2.6371853456
H -4.5660966852 -0.6560581241 3.6040179523
H -5.6944732184 0.110014917 2.4519154783
H 2.7960545918 2.7245056926 0.5187021254
H 4.5355947495 2.4174639482 0.3294999257
H 3.4902163534 2.472880728 -1.1046744308
C 3.4852209327 -2.1013430678 0.0687354646
H 3.3140642651 -3.1439834821 -0.1997294371
H 4.4422602445 -1.784637399 -0.3538035796
H 3.5316501492 -2.024306417 1.1618828663
H 3.9412659149 0.241960794 0.7405900817

4bH$^+$

C -1.9533776987 -2.8788096105 -1.5156082219
C -1.6506648487 -2.3649550969 -0.0929846187
O -0.2906593395 -1.9116265077 -0.2623081623
C -0.5616054862 -1.0577526948 -1.4013327716
C -1.2812352667 -2.0662761863 -2.3299988654
C -2.3500392092 -0.9869574274 0.055042383
C -1.6852913347 -0.163591361 -0.772096203
C -1.9354027797 1.2475795679 -1.1384482397
O -3.2343864962 1.5461093486 -1.1871286748
C -3.5615876928 2.9226976506 -1.491559519
C -3.5744900773 -0.825630944 0.8643698569
O -3.6539993833 0.3613284971 1.4750801851
C 0.703908251 -0.4078363135 -1.9323825167
N 1.3511025613 0.5022631582 -0.9961446515
C 2.3064005972 0.171953784 -0.1046409859
N 3.0544870432 1.1354377828 0.4493206543
C 3.1404855301 2.5486780462 0.0296456787
N 2.5229995908 -1.1195453481 0.1868364587
O -1.0434377754 2.0472770825 -1.3927041447
O -4.3954873416 -1.7207916402 0.9690746132
H 3.5766305113 0.8897728208 1.2810719387
H 2.8321963722 2.5855207383 -1.0192130481
C 2.2085920376 3.4303582238 0.8677855335
H 0.4569840909 0.1614427438 -2.8313546171
H 1.40628058 -1.1933109669 -2.2225085016
H -1.7559304067 -3.0561821715 0.7400154705
H -4.6495567145 2.9693185309 -1.4775676382
H -3.1799746275 3.194283953 -2.4781385334
H -3.1402074039 3.5890595758 -0.7358320181
H -1.2542359166 -2.0291449369 -3.4114104021
H -2.6301500534 -3.6906817657 -1.7472750455
H 1.7371098569 -1.747626448 0.0394100304
C -4.8442784792 0.5831339536 2.265222612
H -4.7257244 1.576986624 2.695227722
H -5.734073086 0.5453553032 1.6327021317
H -4.9230256942 -0.1669169338 3.0554227193
C 3.6825683188 -1.6840233514 0.9044927722
C 4.6005648599 2.9940125843 0.1301413027
C 4.0279310807 -3.0331298902 0.2706079654
H 4.5202973319 -0.9981975603 0.7449804596
C 3.4028981955 -1.809355757 2.4068190715
H 2.277872795 4.4709238115 0.5354012887
H 1.1659175796 3.1107853873 0.7738868985
H 2.4876877058 3.3891430922 1.926432629
H 4.6957128228 4.0341038686 -0.1947585078
H 4.9566405695 2.9294362793 1.1649307933
H 5.2461125006 2.3737210821 -0.4987152532

H 4.8988876513 -3.4678146118 0.7695901066
H 3.1931413483 -3.7360411772 0.3736619591
H 4.2575858161 -2.9208715226 -0.7931466732
H 4.280896219 -2.2148195827 2.9193978969
H 3.1654746412 -0.8383020191 2.8536039308
H 2.5589281036 -2.484533688 2.5859062184
H 0.9359579498 1.4264040228 -0.9479794673

4cH^{+}
C 2.950812899 -1.4932306228 -0.718614771
C 2.2832140933 -0.1332706843 -0.4244745369
O 0.9902415047 -0.3410339315 -1.0337050672
C 0.6931102253 -1.5678825955 -0.3251586759
C 1.9652435416 -2.3875634698 -0.654416297
C 1.8568477026 -0.1449334604 1.0699643895
C 0.8707591145 -1.0535505743 1.1439244238
C 0.1125868038 -1.5672640412 2.308071391
O 0.8735564817 -1.7244129149 3.3900956402
C 0.1967486928 -2.1749383471 4.5872578172
C 2.4675102084 0.6851120313 2.1301225892
O 3.7423658642 0.9745765742 1.8109389284
C -0.6417116277 -2.1483089841 -0.7529067004
N -1.7835245123 -1.2903258333 -0.4520930566
C -2.222059379 -0.302562972 -1.227383254
N -3.3028454496 0.4444829302 -0.9493377054
C -3.6421541318 1.2915315471 -2.1050604543
C -2.3161384027 1.2929002766 -2.8900473961
N -1.660527171 0.0662541027 -2.3951098742
O -1.0795774476 -1.8426109037 2.2662526309
O 1.9019545823 1.0775644345 3.1319928391
H -2.1580401775 -1.3671992253 0.4890659926
H -4.0167240574 0.105943424 -0.3150181267
H -3.9423371198 2.2899733837 -1.7851381493
H -4.4544725674 0.837007926 -2.6813732544
H -0.8041323786 -3.0999787394 -0.2441343882
H -0.6115391149 -2.3506866164 -1.8267185811
H -1.7033763152 2.1670452242 -2.6483670736
H -2.4679622626 1.2468108978 -3.9689174508
H 2.7673916191 0.7696646901 -0.7880485428
H 0.9729791754 -2.241230768 5.3483327984
H -0.2603758816 -3.1527136835 4.420831842
H -0.5685028023 -1.4542486667 4.8835437581
H 1.9950847008 -3.4661386053 -0.7395209794
H 4.0120957107 -1.6407223664 -0.8702396665
H -0.6576260439 -0.0441443723 -2.5076763513
C 4.4579987195 1.801045622 2.7555531925
H 5.4574238712 1.9207099467 2.3388246977
H 3.9688840283 2.772573148 2.8583186415

H 4.5083673032 1.311102219 3.7306957813

4dH+
O 4.1189528025 1.2476470847 -0.3188146324
O 1.7466232255 0.343661891 3.1771820052
O -0.4687661123 0.3413535199 2.7459757928
O 0.5079481387 -0.6216081417 -1.1849397916
N -2.3695721992 -0.400515771 -1.8820803732
N -3.6378126737 0.9160127912 -0.4497544628
H -3.9423967188 1.0722849502 0.5032791325
N -1.9904357931 -0.4491007455 0.4228230341
H -2.0117710356 0.0929069694 1.280806806
C 0.6910755087 0.2219939609 2.3741980538
O 2.8888112844 2.3419909963 1.240778753
C 1.0650743582 -0.1157025701 0.9805857929
C 2.0413043387 0.3613289105 0.1919117707
H -1.4330887986 -0.7588973187 -2.0343927793
C -2.659750112 0.0251486829 -0.6448432334
C -3.136735569 0.0563127316 -3.05052938
H -3.9974708493 -0.6045274604 -3.2030122976
H -2.4840059508 -0.0320620576 -3.9198682731
C 0.3641403857 -1.2239240612 0.1238490441
C 3.0329149054 1.4236676503 0.4566104916
C -4.3680982463 1.6000310585 -1.5256818337
H -4.5047226253 2.6429567337 -1.2289250056
H -5.3591803887 1.1434339371 -1.6275364487
C -1.0804618435 -1.5860411127 0.4204004547
H -1.4182564925 -2.3226142711 -0.3131829487
H -1.134216172 -2.0578985004 1.4040200161
C -3.5945645885 1.4974683963 -2.8365303767
H -4.2381058073 1.8150745618 -3.6608363559
H -2.7213718714 2.1579030639 -2.8128250749
C 1.9435164303 -0.4702473714 -1.1174092106
H 2.3503009735 -0.0168020628 -2.0177263542
C 5.1671368319 2.2301371694 -0.1698280569
H 5.5479594088 2.2302025338 0.8541380061
H 4.7950094056 3.2257536775 -0.4225897193
H 5.9494970668 1.9289694521 -0.8655923087
C 1.3820518905 -2.384527321 0.003341165
H 1.2929477474 -3.3337703805 0.5157285569
C 2.3588132637 -1.91580149 -0.7723160325
H 3.2911940538 -2.3784098338 -1.0686386556
C 1.4647886605 0.7059737831 4.5499161774
H 2.435734075 0.7472177164 5.0415256984
H 0.9738394983 1.6806272957 4.5908839475
H 0.8299865946 -0.0485120483 5.0194679067

4eH+

C	-3.4040052126	1.7722213553	1.1645201332
C	-2.8850081042	1.4582043419	-0.2539796313
O	-1.4678643457	1.6733800746	-0.0630402645
C	-1.3372542504	0.7825343711	1.072498346
C	-2.4474478679	1.349261451	1.9899894708
C	-2.8779670203	-0.0876627214	-0.4103531324
C	-1.9216425847	-0.5196184737	0.4274318873
C	-1.4824598273	-1.8921581513	0.7706584375
O	-2.4980227077	-2.7422942707	0.9030852392
C	-2.1473453016	-4.1172327064	1.1895943678
C	-3.7766226711	-0.8544958784	-1.2990213542
O	-4.9142740799	-0.1673356084	-1.5084331893
C	0.0813146154	0.7741927624	1.6124615779
N	1.0738761186	0.2964724194	0.6551045291
C	1.7564384316	1.0529926099	-0.2170733863
N	1.3507285195	2.3285045299	-0.4361826023
C	2.0619538233	3.3653392614	-1.101665488
N	2.8298067684	0.5551176665	-0.8316230158
O	-0.3087124206	-2.1973234661	0.9400785212
O	-3.5234672087	-1.9358718521	-1.7933290377
H	1.1341947595	-0.7143970979	0.5740293751
H	0.1270867302	0.1231097312	2.4879336442
H	0.3446180245	1.7839855024	1.9351804329
H	-3.2783444521	2.0334623218	-1.0881317006
H	-3.0964029446	-4.6466987908	1.2598843766
H	-1.602370509	-4.1814466087	2.1337501052
H	-1.5386871005	-4.5286625376	0.3815395762
H	-2.4200503014	1.324097602	3.0716612789
H	-4.3776406367	2.1885418135	1.3872014167
H	3.1728843679	1.0584280769	-1.6416657859
C	-5.8841467895	-0.8007801895	-2.3719625042
H	-6.7213454797	-0.1060773483	-2.428821031
H	-5.4588459829	-0.9677476846	-3.3642084807
H	-6.2083178209	-1.7535625406	-1.946942962
C	3.4564025603	-0.7392326349	-0.5367235974
H	0.3482551028	2.4787386828	-0.3349424595
H	3.4260772216	-0.8941593531	0.5465615596
C	4.9010640747	-0.7580710724	-1.0353050791
H	2.8817926306	-1.5480714436	-1.0063555099
C	5.5749969988	-2.1043794854	-0.7661209309
H	4.9112238394	-0.5459877675	-2.1121821551
H	5.4587793249	0.0483856642	-0.5446837116
H	6.6057541865	-2.1009159209	-1.1337252146
H	5.603078818	-2.3280268536	0.3062278663
H	5.0441740186	-2.9224117806	-1.2658345173
C	1.3323290049	4.2228502995	-1.9356103414
C	1.970477164	5.2912900931	-2.5635823047
C	3.3381741616	5.5052657651	-2.3770304918

C 4.0603876718 4.6493084268 -1.5430057088
C 3.4304681098 3.5863831235 -0.8943237414
H 0.2706676979 4.0478839496 -2.0852524892
H 1.3955950451 5.9523274181 -3.2052183987
H 3.835146738 6.3335540246 -2.8725431727
H 5.1214579201 4.8133010076 -1.3802955511
H 3.9972801719 2.9470898921 -0.2269472003

4fH+
C -2.504602955 -1.9056298587 -1.5890834659
C -1.942369614 -1.706051605 -0.1661963218
O -0.5603111695 -1.4148519757 -0.4723606983
C -0.8405152064 -0.338313398 -1.3999965501
C -1.8258344914 -1.0535680797 -2.3561917442
C -2.3897869242 -0.2966307207 0.3116804575
C -1.7149399959 0.5654507586 -0.4657640712
C -1.7583783426 2.0444460534 -0.5368967155
O -2.9945624154 2.5310575796 -0.4503553238
C -3.11591694 3.9734438055 -0.4606053771
C -3.3692274978 -0.0351162922 1.3870998133
O -4.2007266332 -1.0850355263 1.519164652
C 0.4312631081 0.2271689514 -2.0064284806
N 1.3518927755 0.8062686277 -1.0349489729
C 2.3365497072 0.1439375999 -0.4011183894
N 3.3104623148 0.8349944678 0.2081659785
C 3.4990303074 2.2930639319 0.105834122
N 2.3806905779 -1.195566568 -0.4341221541
O -0.7600310802 2.7368715256 -0.6882024441
O -3.4105921253 0.9670388631 2.0742839968
H 1.1095158264 1.7405515852 -0.7202160956
H 0.1667296189 1.0095331363 -2.7211578298
H 0.9421256889 -0.5654428572 -2.5583486542
H -2.0407449067 -2.5198257096 0.5477242814
H -4.1841616897 4.1721265749 -0.3875461552
H -2.7132079797 4.3827676541 -1.3894908009
H -2.5879605958 4.4034326968 0.3933078415
H -1.9352288565 -0.8224031387 -3.4079126024
H -3.3247198183 -2.56460222 -1.8425488986
H 1.4877018668 -1.669379889 -0.5410900536
C -5.2072669646 -0.9678667573 2.5487346046
H -5.7784565916 -1.8945019749 2.5045188776
H -4.7379752302 -0.8559194067 3.528933499
H -5.8561159594 -0.1111342701 2.3527332731
H 2.8114302778 2.8098283728 0.7869305649
C 4.9054075284 2.6765002133 0.4044655639
H 3.2616062729 2.6023531384 -0.9144589391
C 3.511497806 -2.0054350572 0.0351692518
C 3.3951716846 -3.4252199748 -0.5044004381

H 4.435717761 -1.5369554385 -0.3162830098
H 3.5394471976 -2.0169139025 1.1327636113
H 3.8662284908 0.34520075 0.8980123682
C 5.9543868264 3.0764166135 -0.3696089958
C 7.057052363 3.2993202264 0.520337748
C 6.5992452216 3.0139145989 1.7697761231
O 5.2884811632 2.6350321795 1.7213684856
H 7.05002422 3.0320167253 2.7508729431
H 8.0533516127 3.6288694148 0.2610748806
H 5.9411571197 3.198987538 -1.4437268103
H 4.2364132153 -4.024291953 -0.1449898231
H 2.4701453972 -3.9020620387 -0.1628701433
H 3.4066780331 -3.4320199699 -1.5986199795

5aH$^+$
C 0.4398427502 9.4259102796 10.5758343226
C 0.5243965175 8.0610446345 9.9126884231
O 1.743565447 8.1739562221 9.1500156608
C 2.5558670287 8.6753042199 10.2190094711
C 1.7043689979 9.8026281093 10.778904238
C 0.976813164 7.0024268882 10.9746752184
C 2.4758776257 7.430869031 11.2281898037
C 3.9619460196 8.979347531 9.7783836979
N 4.6029806204 7.7519367592 9.329532408
C 4.3602966269 6.5476610521 9.8597170919
N 3.4104116787 6.3727628746 10.8062052157
C 3.3110861847 5.0740729858 11.4908862219
C 2.788918315 7.8780561568 12.6668297246
O 4.0914316476 7.7624754869 12.9457113117
C 4.50249429 8.2248909823 14.2555318128
C 0.0290643143 6.8742685459 12.1544893219
O -0.9952792882 7.5123460415 12.2876976375
N 5.0909412821 5.497381241 9.4576231869
C 6.1657043065 5.5667148976 8.4719263665
O 1.9633372659 8.3366440652 13.4287727154
O 0.4066878873 5.8823044404 12.9783512714
C -0.4279930314 5.6674177502 14.1373474657
H 4.7848076426 4.5761804865 9.7338373809
H 0.9994608222 6.0248803945 10.4860959235
H 2.9398542105 4.2934041702 10.8178515424
H 2.6066009613 5.165467483 12.315175101
H 6.6250545651 4.5803014247 8.4169429271
H 6.9284389577 6.2884748618 8.7789781091
H 3.946328731 9.681639173 8.9432949165
H 4.5176259277 9.4368472143 10.604443581
H -0.3050337108 7.7419619751 9.2842878577
H -1.4427987896 5.4017497555 13.8333353614
H -0.4525724682 6.5653339259 14.7585813651

H 0.0326868969 4.8430041517 14.6807135906
H 5.574155281 8.0369442045 14.3044573911
H 3.9791429472 7.6664015197 15.034037226
H 4.2952313605 9.2917259373 14.3620563098
H 2.0906689648 10.6690541741 11.3000302184
H -0.4724617098 9.9154741955 10.8869100714
H 5.7908406452 5.8354380384 7.4776921195
H 4.2828489893 4.7799740159 11.8956912276
H 5.2580491253 7.8169717034 8.5631861927

5bH^+
C -2.6003240345 2.2121568658 -0.1565615396
C -1.7364272682 1.6891904686 -1.2935649658
O -0.4366158467 2.2089159172 -0.9471890805
C -0.4459230529 1.7675250752 0.4159434065
C -1.7982590639 2.2573642399 0.9102132276
C -1.4696483779 0.1626251193 -1.0794535022
C -0.5511115418 0.1818757583 0.2082841622
C -1.1867472338 -0.4557608926 1.4591128731
O -0.3185446761 -0.4804673926 2.4775524273
C -0.8236965452 -1.0021457775 3.7321372113
C -2.7345289932 -0.6794317208 -1.1051382633
O -2.4491619254 -1.9915738905 -1.160384434
C 0.7998016539 2.1733716485 1.1521499428
N 1.9460400381 1.5330648188 0.5249532623
C 1.9263564792 0.296069539 0.0063671163
N 3.0751795178 -0.2285193955 -0.4433617156
C 4.4299564658 0.3504093373 -0.311870434
N 0.7789404582 -0.4277820324 -0.0417872956
O -2.336504167 -0.8340374631 1.5363726309
O -3.8592461895 -0.2283126597 -1.1861017342
H 2.7465104755 2.1153310883 0.3270703237
H 3.0147963858 -1.0545203013 -1.0201188181
C 4.7762331878 1.2347109299 -1.5152203053
C 5.4256014577 -0.7979482041 -0.1389305154
H 0.7183735472 1.8942132783 2.2069491413
H 0.9385022175 3.2535301666 1.08850525
H -2.0105795487 1.9592746223 -2.3118172454
H 0.014350894 -0.9469484114 4.4252446887
H -1.150102701 -2.0361218392 3.6058014847
H -1.6558743741 -0.3904504979 4.0861603985
H -2.0328465645 2.495425293 1.9401652066
H -3.6607035036 2.4134023447 -0.2180296833
H -0.849184656 -0.1785447038 -1.9094210004
C -3.5832702343 -2.8839408487 -1.1976942484
H -3.1637690791 -3.8888489329 -1.2354027037
H -4.1933671008 -2.760818306 -0.3000988344
H -4.19139165 -2.6932391788 -2.0847068561

C 0.7575933203 -1.9169798595 -0.2932541547
H -0.2936171514 -2.1697834814 -0.1526237808
C 1.0929507749 -2.3319958324 -1.7371437984
C 1.552955164 -2.7135308645 0.748092408
H 4.4368515348 0.9515789547 0.6026864866
H 0.6911768837 -3.3362897201 -1.9020305971
H 0.6419292274 -1.663104485 -2.4737944789
H 2.1671394741 -2.3939954247 -1.941824711
H 1.3392556391 -3.7768332681 0.6022386367
H 2.632402672 -2.5764567554 0.6521250319
H 1.255754421 -2.4404292672 1.7630835876
H 6.4371097848 -0.3957802755 -0.0333903533
H 5.1953184106 -1.39122203 0.7506847506
H 5.4145245143 -1.4585525822 -1.0135033073
H 5.7741449716 1.6651108932 -1.3858974978
H 4.7732247549 0.6446198903 -2.4380210018
H 4.0641851535 2.0576710456 -1.6325437986

5cH^+
C 0.4440156775 9.5778534404 10.7326343748
C 0.4452381469 8.3029279518 9.907001686
O 1.6485620356 8.4526305192 9.1238364031
C 2.513370683 8.7925377663 10.2119283905
C 1.7295381433 9.8801411474 10.9292922635
C 0.8760812486 7.1019242839 10.8142628479
C 2.3870681805 7.4392125575 11.0887609122
C 3.9179957189 9.1139086906 9.7597395823
N 4.5833861331 7.9189781054 9.2407716519
C 4.3057225664 6.7040499054 9.694288533
N 3.2581426481 6.4325646717 10.495461575
N 5.0103711929 5.5944853911 9.4260437723
C 4.2774370036 4.4074115336 9.8936627774
C 3.3224014024 5.0210730203 10.9327765785
C 2.7493217702 7.7013183197 12.5596630623
O 4.0634330283 7.5549597862 12.7654783357
C 4.5336475116 7.8437983625 14.105278233
C -0.0425899297 6.8360770681 11.9913231983
O -1.04014437 7.4779417082 12.2505945496
O 1.9574334379 8.0560045558 13.4068539337
O 0.3328221285 5.7254669892 12.646807473
C -0.4730106462 5.3616025744 13.7881315727
H -0.4352708832 10.058799355 11.1381633171
H 2.1691413018 10.6605566687 11.5369585732
H 5.6570779537 5.5583103932 8.6473653742
H 5.3878644629 8.0356310921 8.6365996259
H 0.8591094583 6.2002628917 10.1967840165
H 3.8759971908 9.8582279075 8.9624897381
H 4.4857172781 9.5348233689 10.5965115274

H -0.4149384736 8.1010907966 9.2715772054
H -1.5047509987 5.1712667438 13.4838825644
H -0.451208481 6.1561356867 14.5373681037
H -0.0226741449 4.4530234228 14.1868384189
H 5.6074187291 7.6645215179 14.0791034506
H 4.0513100882 7.1790547116 14.8246076312
H 4.3241827378 8.8840305839 14.362489997
H 2.3339908944 4.562515643 10.9162996416
H 3.7299981159 4.9674036765 11.9476278747
H 4.9588579977 3.6793189149 10.3343654132
H 3.7323180612 3.9422742762 9.0659068203

5dH^+
C 0.4764741878 9.6388918747 10.6913623492
C 0.4423235018 8.328348646 9.9233592329
O 1.6264673408 8.4276668902 9.1060653046
C 2.5208133697 8.7956409825 10.1627982352
C 1.7706564189 9.9249626434 10.8506120637
C 0.8805638905 7.1630166004 10.8725005471
C 2.4121007821 7.4849993739 11.0858474186
C 3.9177766515 9.054198549 9.6695372182
N 4.4685903084 7.825304636 9.1225119609
C 4.2098868115 6.6028716028 9.6104291325
N 3.2668689609 6.4064478795 10.5555098781
N 4.9134640163 5.5723674092 9.121826705
C 4.7821651725 4.1854229164 9.571728495
C 4.2984244223 4.1910629779 11.0134099639
C 3.0478003794 5.0513573862 11.1208237753
C 2.8068572098 7.8120460859 12.5376085858
O 4.1179141891 7.6434961544 12.7397355314
C 4.6055233191 7.9915391436 14.0587635496
C -0.019425464 6.9844173442 12.0820038566
O -0.9987431846 7.6636064324 12.3156883703
O 2.0345086633 8.2333372907 13.3730341829
O 0.3349313673 5.9056516209 12.7991523156
C -0.4687471081 5.631750108 13.9670552949
H 5.6244810484 5.7860307362 8.4334921229
H 5.2118522165 7.9040884705 8.4402675796
H 0.8181712637 6.2339261273 10.3015240798
H 2.214456112 4.5746663927 10.5941821226
H 2.7540227235 5.1740364921 12.1641840158
H 5.7628686636 3.7153759689 9.4772321339
H 4.0793997021 3.6458763208 8.9260184211
H 3.8961359522 9.8050612283 8.8774053963
H 4.5337864119 9.4369213314 10.4915998402
H 4.0573879131 3.1746783325 11.3341768394
H 5.0824280193 4.5810747621 11.6704673697
H -0.4361072552 8.1089954688 9.3191705685

H -1.5068340006 5.4457943486 13.6823297061
H -0.4237401471 6.4707300945 14.6649321485
H -0.0348090411 4.7398910392 14.4183058476
H 5.6742671639 7.7830741388 14.0356460177
H 4.1118459656 7.3799073738 14.8164894138
H 4.4251840524 9.0491378491 14.2614424946
H 2.2366526296 10.7236694167 11.4132466046
H -0.3866766004 10.1541275596 11.08909931

5eH^{+}
C 0.4180147806 9.4335571103 10.7098157853
C 0.43163416 8.0659831493 10.0468259582
O 1.601407264 8.1495141536 9.2052714665
C 2.4904375041 8.6369851352 10.2156119727
C 1.7003913101 9.7848664841 10.8267040949
C 0.9286201317 7.0019119921 11.0809112293
C 2.4422393475 7.4036887006 11.2453835321
C 3.8666920285 8.9350430515 9.6904386124
N 4.5340275612 7.7024935884 9.2991254105
C 4.3302267191 6.5086015262 9.8614680537
N 3.3471249029 6.3176871549 10.7856299921
C 3.2117962837 5.0091406123 11.3996875588
C 2.8444579453 7.8877395117 12.6493198764
O 4.1734098305 8.0271699331 12.7451098228
C 4.6694879416 8.5559400338 14.0004702588
C 0.0432760514 6.8829992593 12.3106196776
O -0.9300912707 7.5782235052 12.5250771235
N 5.1059671505 5.4809159087 9.4987120985
C 6.2170682626 5.5632759913 8.5424429516
O 2.063403012 8.1810005679 13.5286453586
O 0.398658643 5.8331503168 13.0644704274
C -0.3833547859 5.6178468103 14.2572894117
H 4.8829761469 4.569571602 9.8768920907
H 0.9020033083 6.0284284401 10.5903744336
C 6.9701806133 4.2344649392 8.4999171984
H 6.8962324652 6.3675792007 8.8501942851
H 3.7897009817 9.5767051375 8.8105704044
H 4.4456629645 9.4641731856 10.4543567208
H -0.4436353629 7.7632355625 9.4752322224
H -1.4285256844 5.4263933374 14.0029858282
H -0.3197920124 6.4862650966 14.9169599875
H 0.0543988455 4.7439967716 14.7391147016
H 5.7519373381 8.5956906736 13.8884610542
H 4.3930206998 7.8944688051 14.823759386
H 4.2640831879 9.5545172859 14.1745722947
H 2.1393474341 10.6449314547 11.3163659776
H -0.4621031007 9.9421320429 11.0773817199
H 5.8310218196 5.8098022706 7.5447972918

H 5.2545683502 7.7736370676 8.5940439548
C 2.4844349042 4.0088259963 10.7430604244
C 2.342870719 2.7544617377 11.3384858275
C 2.923617404 2.4995611825 12.5824435094
C 3.6588340067 3.4965615748 13.227553882
C 3.8110813787 4.751832841 12.6372976657
H 2.0414802563 4.211271502 9.7730811903
H 1.7780345951 1.9802655397 10.8286383431
H 2.8096406436 1.5238192212 13.0449732899
H 4.1223319397 3.2977976025 14.1888926497
H 4.3980843716 5.5206679028 13.126127685
C 8.1346583947 4.2732730291 7.5096820976
H 7.3393019705 4.0025510495 9.506220186
H 6.2700745934 3.4366282638 8.2224263342
H 8.6597503638 3.3132984441 7.4944928768
H 7.7853078589 4.4797568902 6.4919220099
H 8.8611368316 5.0478488529 7.7795548034

5fH^+
C 3.0270992554 -0.9763623614 -1.6941249513
C 1.9176395653 0.0195476349 -1.9872481921
O 0.8021753625 -0.8499752786 -2.2767739047
C 0.9423912868 -1.6797052682 -1.1177153007
C 2.4224236258 -2.0282346652 -1.1378970075
C 1.4210195843 0.6433195862 -0.6393368552
C 0.6962468726 -0.5866080521 0.0310853417
C 1.3116028484 -1.0680746981 1.3575282772
O 0.440329861 -1.798869328 2.0619962008
C 0.9384384625 -2.362415861 3.3009169169
C 2.4981719406 1.3841702562 0.1322529139
O 1.9867955929 1.9904595173 1.2174231007
C -0.0520663709 -2.8066850015 -1.0801626271
N -1.3993794435 -2.2697577879 -0.9588980486
C -1.7122984743 -1.1501084417 -0.3003271455
N -3.0056803694 -0.8474993566 -0.1125553406
C -4.1269920829 -1.6899014991 -0.552814977
N -0.7518456074 -0.3290993239 0.1980994334
O 2.4610087361 -0.8654666805 1.6878615574
O 3.6488827356 1.5087174796 -0.2340234206
H -2.1408858777 -2.7606490788 -1.4384966918
H -3.2309004102 0.1157307706 0.1097805718
H -4.16887579 -1.7217361303 -1.6492635781
C -5.4332181438 -1.1447777598 0.0089164538
H 0.1795498029 -3.4757787136 -0.2444047515
H 0.0035849428 -3.3812090325 -2.0063002314
H 2.0714938547 0.7412962071 -2.787129359
H 0.0996682509 -2.9128681926 3.7244556155
H 1.25409913 -1.5650412491 3.976683961

H 1.7764199227 -3.0329605382 3.1004496373
H 2.8520075578 -2.9217408725 -0.7035702935
H 4.0838486028 -0.79655053 -1.834287334
H 0.6507288844 1.379644721 -0.8768916996
C 2.9186988614 2.7448436422 2.0227815052
H 2.3299831896 3.1581325778 2.8411650996
H 3.701597098 2.0897609452 2.4109838977
H 3.3681223204 3.5480513315 1.4347535968
C -1.1276674726 0.7381614964 1.1624330054
H -0.1963780536 1.0868678762 1.6135409605
C -1.8675040476 1.9032754599 0.5853380206
H -1.7384995995 0.3087948299 1.9613558335
H -3.9574649783 -2.7094066203 -0.1914767178
H -6.2624198023 -1.7775985341 -0.3184062111
H -5.4167302017 -1.1344937482 1.1030177489
H -5.6219616306 -0.1269040946 -0.348131821
C -3.0402262988 2.5103526247 0.9352936913
C -3.1890574868 3.6443828365 0.0686708023
C -2.0999295503 3.6446169535 -0.7451683202
O -1.2803511491 2.5930959458 -0.442051626
H -1.772099635 4.2796815497 -1.5545391155
H -4.0013315014 4.3572331845 0.0556808482
H -3.7111001705 2.1902802716 1.7207495301

c) Optimized structures used in NMR calculations

9c_c1

C -0.1697057838 -0.5455852607 0.1508831642
N 1.2012430853 -0.6244273779 0.1113064355
N -1.1041671316 -1.4087300268 0.2552436233
N -0.5146702334 0.8014734049 0.1114520494
C -1.9453296625 0.9088017963 0.3659564644
C 1.7714148514 0.6650235415 0.1189916125
C 0.5823007768 1.6270432129 0.1964304822
C -2.3510274609 -0.6086880489 0.2809983508
H -2.1309846392 1.3455096128 1.3560538684
H -2.4415086107 1.5287132237 -0.3871233446
H -2.9659127041 -0.907593724 1.1356222181
H -2.9309872661 -0.8104716205 -0.6273254113
C 0.5162280324 2.976415552 0.3089616178
C 1.9728205062 -1.8672327546 0.0252119404
C 1.9035033605 -2.5289418575 -1.3100089269
H 3.002558424 -1.5916425 0.2671280141
H 1.5954352329 -2.5516106541 0.7897479969
H -0.4715742835 3.427643848 0.3060332322
C 1.6281825529 3.9275288393 0.4448708031
O 2.9558771702 0.8889699035 0.0750969646
O 1.1168946696 5.196256902 0.4107853235
O 2.8110253165 3.7000765509 0.5837419556
C 2.090388893 6.237635944 0.5477611306
H 1.5294932182 7.1723477626 0.5031430276
H 2.618492713 6.1526771595 1.5020279098
H 2.824109369 6.1920901747 -0.26223497
O 2.7462906124 -2.0560451757 -2.2807534137
C 2.5005886181 -2.7791347264 -3.4083079921
C 1.5242076033 -3.7008459961 -3.1826223497
C 1.1333877306 -3.5357810293 -1.81385726
H 0.3686138946 -4.0778227086 -1.2754631595
H 1.1308924334 -4.4109807716 -3.8965792299
H 3.1008967114 -2.5223031961 -4.2678631271

9c_c2

C -0.1894041328 -0.5214305897 0.2341060417
N 1.1749297149 -0.3311276902 0.1634702876
N -0.9106142811 -1.5490656382 0.4495397749
N -0.8152938845 0.714807386 0.0582557028
C -2.2503174687 0.5194070233 0.3040596081
C 1.4533935761 1.0259748217 -0.0062431221
C 0.102725619 1.7243141183 -0.0493910897
C -2.3040863017 -1.0444220998 0.4145765774
H -2.5480061265 1.0215385829 1.228887083
H -2.8458495088 0.9280548015 -0.510730892

H -2.8339974655 -1.3664680942 1.3167373636
H -2.8228645012 -1.4874938618 -0.4438987502
C 0.0231983406 3.0706915105 -0.1776152206
C 2.1779392729 -1.3980069827 0.231172213
C 2.2694633907 -2.2182175064 -1.0114099909
H 3.1288759509 -0.8988823567 0.4347629697
H 1.9231279431 -2.0430737413 1.0760590821
H 0.9658260728 3.6021834864 -0.2344517161
C -1.2107148759 3.8468758732 -0.2644721059
O 2.5543956211 1.5274441775 -0.0844884226
O -0.9315703566 5.1721129272 -0.3558418195
O -2.3529919751 3.4125354146 -0.2678197884
C -2.0733098477 6.0319199739 -0.4618517503
H -1.6720516259 7.043879405 -0.5276884877
H -2.6571878219 5.7920703279 -1.3550506779
H -2.7170210809 5.9312391857 0.4166744756
O 3.0479934225 -1.7227745382 -2.0240065123
C 2.9682124743 -2.607550482 -3.0563268346
C 2.1604123247 -3.6535670335 -2.7290041482
C 1.7031501495 -3.3981894056 -1.3952540319
H 1.0266935064 -3.9975447708 -0.8022580943
H 1.9195855533 -4.5019568347 -3.3542406874
H 3.541938322 -2.35352239 -3.9346550368

9c_c2b

C -0.3752470491 -0.6133226374 0.4540908687
N 0.9828695244 -0.5287084519 0.2225161589
N -1.1317595892 -1.5566128268 0.851303537
N -0.9368805415 0.6437852855 0.2143728088
C -2.3454276462 0.5809389766 0.626741855
C 1.3214608856 0.7761110356 -0.1274257738
C 0.0239331085 1.5671351233 -0.098249098
C -2.4853038691 -0.956063216 0.9074090986
H -2.5066340235 1.1926972215 1.5187852075
H -3.0002427258 0.9503676632 -0.1608832152
H -2.9311022003 -1.1474026727 1.8887312341
H -3.122773664 -1.4424493392 0.1594650019
C 0.0158251905 2.896774498 -0.359318136
C 1.9384420402 -1.6401608078 0.2777128827
C 2.2740124495 -2.2113933621 -1.0591898965
H 2.8476636753 -1.2545646042 0.7451518183
H 1.4977289104 -2.4025536161 0.9246773586
H 0.9774610104 3.3474383968 -0.574851239
C -1.1671356785 3.7520858543 -0.3922384741
O 2.4384118465 1.1760430341 -0.3849565233
O -0.8158604233 5.0368544651 -0.6550165201
O -2.3274891933 3.4081002669 -0.2229264269
C -1.9044695563 5.9663873884 -0.7251799191

H -1.4495046068 6.9336115285 -0.9417987235
H -2.6034182587 5.685290352 -1.5181014519
H -2.4457847562 6.0015418108 0.2245806174
O 1.4450672995 -3.1884045956 -1.5440469068
C 1.9180906469 -3.5372215022 -2.7728216456
C 3.0251787012 -2.8084408548 -3.0844039128
C 3.2562214445 -1.9422218018 -1.9667585082
H 4.0366446802 -1.2028101938 -1.8537906002
H 3.6076347759 -2.8773045406 -3.9925102452
H 1.3664035922 -4.3121578777 -3.2829912312

9c_c3
C -0.2774305405 -0.5732146449 0.3543534059
N 1.0831740263 -0.808295047 0.2894182901
N -1.2818978684 -1.3303705725 0.5647391042
N -0.511299189 0.7795846619 0.1335975879
C -1.9461652837 1.0255778558 0.1973583836
C 1.7294585838 0.3863695317 -0.0217282896
C 0.6478778756 1.460116675 -0.1387056758
C -2.4595475564 -0.434824528 0.5026552763
H -2.1864022738 1.7398475643 0.9904354092
H -2.3155842331 1.4221735982 -0.7530910313
H -3.0008477807 -0.4759142062 1.4543210307
H -3.1447976278 -0.7860631887 -0.2765295951
O 0.7937501584 2.637714168 -0.4000393799
C 1.5853008022 -2.1978923816 0.2290479635
C 1.5205451549 -2.7810843116 -1.1419535965
H 2.6078169136 -2.1902996765 0.6052593061
H 0.9458893071 -2.77913905 0.8963494326
C 3.0194928394 0.745288845 -0.2112982166
H 3.1538679238 1.7827658899 -0.4992877293
C 4.2431753362 -0.0448016879 -0.0555179736
O 5.2961030571 0.6357072247 -0.5724728207
O 4.37123338 -1.1335961806 0.4832209604
C 6.5677769679 -0.0144581577 -0.4450338676
H 7.2888707328 0.6637124634 -0.9025946486
H 6.8123371835 -0.182165431 0.6076610598
H 6.5626481806 -0.977192256 -0.9635795447
O 2.5255400356 -2.4417427644 -2.0112300862
C 2.2548907799 -3.0590975035 -3.1951942568
C 1.1062762593 -3.7841646397 -3.1053395452
C 0.6258139182 -3.5993537543 -1.7678492338
H -0.2717150772 -4.0088145845 -1.3256788612
H 0.6564834336 -4.3779598079 -3.8888796384
H 2.9694625805 -2.8879611032 -3.9859812195

9c_c4
C -0.0351069869 -0.5436372156 0.0263100359

N 1.3379762212 -0.5761302862 0.0904603014
N -0.9309423346 -1.4495711622 -0.058642931
N -0.4363193813 0.7829835271 0.0544979435
C -1.880824727 0.8328698922 -0.1337369474
C 1.8454165531 0.7300482963 0.1244456055
C 0.6292529066 1.6626562519 0.0386138032
C -2.2143431141 -0.7070488739 -0.0753168584
H -2.3624293868 1.4102241152 0.6598006008
H -2.1308379152 1.2887068795 -1.0978130642
H -2.7893011116 -0.9522148318 0.8255954893
H -2.811452137 -1.0208274867 -0.9377232849
O 0.5783002929 2.866787651 -0.0675322556
C 2.0853583628 -1.8340573437 0.1707269974
C 2.6608210336 -2.3055045836 -1.1241323958
H 2.8776387651 -1.7129386247 0.9172587003
H 1.3760591334 -2.5782793654 0.5403838368
C 3.1627133707 1.0293560234 0.1473572734
H 3.8804600043 0.2258118548 0.0228023409
C 3.7577121954 2.3685699186 0.3225246491
O 5.0432409724 2.3385714442 -0.135135293
O 3.2594945375 3.3512595281 0.8264851868
C 5.762344264 3.5690718993 0.0143795472
H 6.7593416877 3.3770656054 -0.3846312597
H 5.2741141457 4.3729047318 -0.5438757172
H 5.8195602439 3.8592968544 1.0675395683
O 3.9226146304 -1.8714251195 -1.4398238479
C 4.234140329 -2.3981064666 -2.6582710414
C 3.2056259117 -3.157425925 -3.1251521761
C 2.1807344032 -3.0974171919 -2.1251986639
H 1.2096834581 -3.5726805323 -2.1458959335
H 3.1780706612 -3.6978027535 -4.0610083369
H 5.2107850104 -2.1445897107 -3.0412508728

9c_c5
N -5.8644942889 1.254711356 -0.9249177857
C -6.4268714482 0.1249739547 -0.6938118126
N -7.7042012009 0.1571051561 -0.1354248382
N -5.8660969044 -1.1184968917 -0.9615855242
C -8.0771818022 1.5599959096 0.0675072993
C -6.8277630505 2.2914642304 -0.5162570832
H -8.2431044417 1.7497245247 1.13257876
H -9.0071490425 1.783705479 -0.4631460211
H -6.3661984157 2.9567281253 0.2224583699
H -7.093175818 2.9088825368 -1.3827771743
C -8.4336839619 -0.933447376 0.3138012946
C -7.7664807351 -2.2070877013 0.0375336856
C -4.4417269501 -1.1049816888 -1.3912467443
O -9.5168837355 -0.8033576508 0.8700113605

C -6.551630567 -2.2603814692 -0.568135442
C -5.9908137215 -3.6032746488 -0.9550848624
O -5.3593539811 -3.8263712964 -1.9671997858
O -6.3301449166 -4.5452331618 -0.0613992562
C -5.9135013339 -5.8830176974 -0.3942765334
H -4.8254350755 -5.9279176844 -0.4822915912
H -6.3643853781 -6.1999636274 -1.3380547268
H -6.2621503878 -6.5054616622 0.4293558602
O -3.2751729148 -2.364125672 0.3408427415
C -3.4907912411 -1.1404990854 -0.2451953661
C -2.774129701 -0.1832790171 0.4098678223
H -2.7686044121 0.8708122359 0.1714719878
C -2.0651628812 -0.8502385679 1.4623850514
H -1.3920234678 -0.4078801991 2.1835174963
C -2.4087653877 -2.1639459649 1.3753315235
H -2.1368065972 -3.0455580424 1.9356472716
H -4.3093815846 -0.1729668012 -1.9392555227
H -4.2905403805 -1.9484583489 -2.0615620645
H -8.284064275 -3.1127392534 0.3176016102

9c_c6

N -5.7110658574 1.6298630157 -0.2866158824
C -5.9796649392 0.3921508647 -0.4890171147
N -7.234821757 -0.0636930832 -0.0567627118
N -5.1345897869 -0.5144807012 -1.1015976641
C -7.9394932943 1.0843028887 0.549859833
C -6.880557195 2.2154726523 0.3779155112
H -8.1839528809 0.8738299241 1.5927411555
H -8.8742133083 1.2807755772 0.0205595882
H -6.586641405 2.6351826788 1.3473827509
H -7.2751156049 3.0436180978 -0.223164974
C -7.636864152 -1.3597700731 -0.2563734701
C -5.4920667292 -1.8552656591 -1.359491021
C -3.7988937601 -0.0357183831 -1.514564539
O -4.7293321922 -2.6121375537 -1.9441544942
C -6.8154788327 -2.2440563419 -0.883908876
C -8.9948041784 -1.7571413887 0.2444288684
O -9.7569231272 -1.0150762875 0.830930665
O -9.2695058764 -3.0411469951 -0.0341180186
C -10.5534224689 -3.5093591194 0.4170481591
H -10.6302293612 -3.4213040187 1.5035986917
H -11.3546877446 -2.9298395175 -0.0481137327
H -10.6027372761 -4.5534505965 0.1094291706
O -2.14291727 -1.1255648052 -0.0909900403
C -2.8136209064 0.0309786632 -0.3960350626
C -2.4121896312 1.0314694259 0.4405273665
H -2.7923537651 2.0426863346 0.4347813353
C -1.4309772749 0.4630343325 1.3163979031

H -0.8898027697 0.9614253364 2.1087861159
C -1.3114980552 -0.8428474568 0.9491694589
H -0.7064825069 -1.6649748246 1.3005593068
H -3.9289142978 0.9593166171 -1.9432407121
H -3.4570305448 -0.7341926132 -2.2792474598
H -7.1164412501 -3.2671969904 -1.0490701067

S7. References

1 Aoyagi, N.; Endo, T. Synthesis of five- and six-membered cyclic guanidines by guanylation with isothiouronium iodides and amines under mild conditions. *Synth. Commun*. **2017**, *47*, 442–448. DOI: 10.1080/00397911.2016.1269927.

2 Acheson, R.M.; Wallis, J.D. Addition reactions of heterocyclic compounds. Part 74. Products from dimethyl acetylenedicarboxylate with thiourea, thioamide, and guanidine derivatives. *J. Chem. Soc. Perkin Trans. 1*, **1981**, 415–422. DOI: 10.1039/P19810000415.

3 Frisch, M.J.; Trucks, G.W.; Schlegel, H.B.; Scuseria, G.E.; Robb, M.A.; Cheeseman, J.R.; Scalmani, G.; Barone, V.; Mennucci, B.; Petersson, G.A.; et al. Gaussian09. Rev D. 01; Gaussian, Inc.: Wallingford, CT, USA, 2009.

4 Frisch, M.J.; Trucks, G.W.; Schlegel, H.B.; Scuseria, G.E.; Robb, M.A.; Cheeseman, J.R.; Scalmani, G.; Barone, V.; Petersson, G.A.; Nakatsuji, H.; et al. Gaussian16. Rev C. 01; Gaussian, Inc.: Wallingford, CT, USA, 2019.

5 Glasovac, Z.; Eckert-Maksić, M.; Maksić, Z. B. Basicity of organic bases and superbases in acetonitrile by the polarized continuum model and DFT calculations, *New J. Chem*. **2009**, *33*, 588–597. DOI: 10.1039/b814812k

6 Glasovac, Z.; Kovačević, B. Modeling pK_a of the Brønsted Bases as an Approach to the Gibbs Energy of the Proton in Acetonitrile, *Int. J. Mol. Sci.* **2022**, *23*, 10576, DOI: 10.3390/ijms231810576.

7 Marenich, A.V.; Cramer, C.J.; Truhlar, D.G. Universal Solvation Model Based on Solute Electron Density and on a Continuum Model of the Solvent Defined by the Bulk Dielectric Constant and Atomic Surface Tensions. *J. Phys. Chem. B* **2009**, *113*, 6378–6396.
